# On-Chip Raman Spectroscopy for Rapid Antimicrobial Susceptibility Testing from Blood Cultures

**DOI:** 10.1021/acs.analchem.6c01690

**Published:** 2026-06-22

**Authors:** Richard Grohs, Franziska Hornung, Marie-Luise Enghardt, Anja Silge, Uwe Glaser, Uwe Hübner, Stefanie Deinhardt-Emmer, Oleg Ryabchykov, Karina Weber, Thomas Bocklitz, Bettina Löffler, Jürgen Popp

**Affiliations:** † 40096 Leibniz Institute of Photonic Technology, Member of Leibniz Health Technologies, Member of the Leibniz Research Alliance INFECTIONS, Member of the Leibniz Centre for Photonics in Infection Research (LPI), Albert-Einstein-Straße 9, 07745 Jena, Germany; ‡ InfectoGnostics Research Campus Jena, Center of Applied Research, Philosophenweg 7, 07743 Jena, Germany; § Institute of Physical Chemistry and Abbe Center of Photonics, 9378 Friedrich Schiller University Jena, Member of the Leibniz Centre for Photonics in Infection Research (LPI), Helmholtzweg 4, 07743, Jena, Germany; ∥ Institute of Medical Microbiology, 39065 Jena University Hospital, Am Klinikum 1, 07747 Jena, Germany; ⊥ Biophotonics Diagnostics GmbH, Am Wiesenbach 30, 07751, Jena, Germany

## Abstract

Bloodstream infections are associated with considerable morbidity and mortality, necessitating timely and accurate antimicrobial susceptibility testing (AST) to guide appropriate therapy. Current diagnostic methods primarily rely on culture-based AST, which is time-consuming, or on genotypic approaches that lack phenotypic relevance. We present the RamanBioAssay (RBA) platform, a novel diagnostic tool integrating dielectrophoretic on-chip bacterial enrichment with label-free Raman spectroscopy, to enable rapid phenotypic AST and simultaneous bacterial identification (ID). The RBA platform delivers AST results within 3.5 h from a positive blood culture, substantially reducing the diagnostic turnaround time compared to standard culture-based techniques. The RBA platform demonstrated high concordance with conventional AST using quality control strains: 94.4% and 100% for *E. coli* treated with ciprofloxacin and *S. aureus* treated with oxacillin in medium controls, 91.7% and 97.2% in artificial blood cultures, respectively. For proof-of-concept evaluation, six patient blood cultures were analyzed, yielding concordance rates of 91.7% and 83.3% for *E. coli* treated with ciprofloxacin and *S. aureus* treated with oxacillin, respectively (1/6 samples showed a S/R mismatch, 1/6 samples was nonconclusive). The mean diagnostic turnaround time for clinical samples was 3 h and 6 min (±24 min). Additionally, the family level classification of *E. coli* and *S. aureus*, shown exemplary in medium controls, was achieved with 97.2% accuracy. These findings highlight the RBA platform as a promising tool for rapid phenotypic AST combined with bacterial ID, providing comprehensive and clinically actionable results significantly faster than conventional methods.

## Graphical abstract

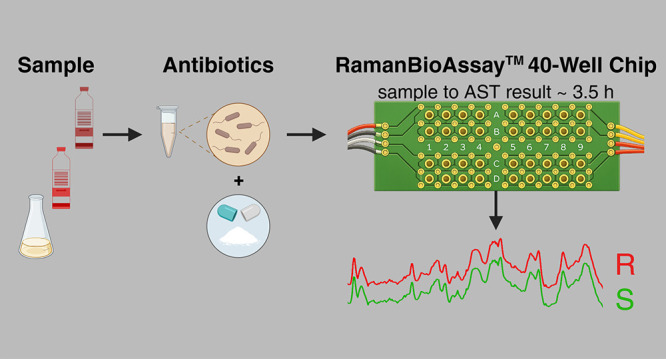

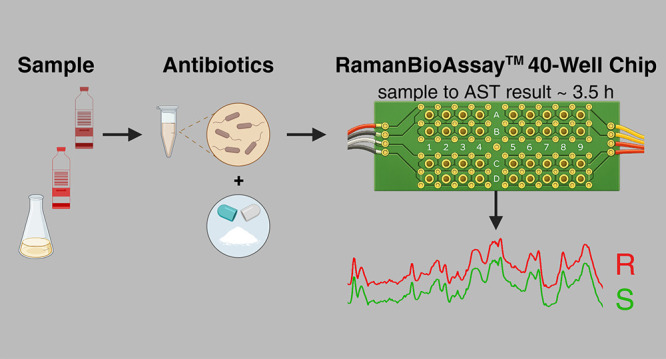

## Introduction

Bloodstream infections (BSIs) are a critical clinical condition associated with substantial morbidity and mortality, particularly among hospitalized and immunocompromised patients.^1^ Rapid and accurate identification of the causative pathogen is essential for the effective clinical management of BSIs and for improving patient outcomes.^2,3^

Blood culture remains the gold standard for BSI diagnosis, although it is time-consuming and can delay the initiation of targeted antimicrobial therapy.^4^ The increasing prevalence of multidrug-resistant organisms further complicates clinical management and underscores the urgent need for improved diagnostic approaches.^5^

Rapid and cost-effective methods for antimicrobial susceptibility testing (AST) are currently unavailable. Conventional phenotypic AST methods, including disk diffusion, broth microdilution (BMD), and automated platforms such as VITEK 2, require bacterial cultivation, inherently limiting the diagnostic speed due to the time needed for microbial growth.

In contrast, genotypic approaches, such as polymerase chain reaction (PCR) and next-generation sequencing (NGS), have emerged as faster and more sensitive alternatives for detecting antimicrobial resistance. Although genotypic assays can identify known resistance genes within hours, they cannot assess phenotypic susceptibility or detect novel or noncanonical resistance mechanisms.^6,7^

Consequently, there is a strong clinical demand for diagnostic platforms that enable both rapid pathogen identification (ID) and phenotypic AST, with the potential to significantly improve patient outcomes by facilitating the early initiation of targeted therapy and reducing unnecessary use of broad-spectrum antibiotics, which contribute to antimicrobial resistance.^8^ Technologies capable of performing rapid phenotypic AST on early bacterial micropopulations would represent a significant advancement. However, balancing speed, accuracy, and cost remains a key hurdle for the widespread implementation of these advanced diagnostics in clinical settings.^9^

Raman spectroscopy is a promising diagnostic technique that offers rapid, label-free, and molecularly specific analysis of small quantities of biological materials. A laser focused on a micrometer-scale spot enables the detailed analysis of bacterial cell composition.^10^ Employing this concept of Raman microspectroscopy, we present the RamanBioAssay (RBA) platform for rapid AST. Spectroscopic cell signatures reveal whether bacteria are susceptible or resistant to treatment after only 90 min of antibiotic exposure. This approach does not rely on visible growth inhibition and is broadly applicable to various bacterial species and antibiotic classes.^11–13^ Additionally, minimum inhibitory concentrations (MICs) can be determined when multiple antibiotic concentrations are tested.^11,14^

Previous studies have demonstrated that Raman spectroscopy can successfully identify bacteria at different taxonomic levels, from the Gram category to even species level.^15–17^ These methods typically require washing and deposition of bacteria onto Raman-compatible substrates, such as calcium fluoride or nickel foil, which immobilizes cells in a two-dimensional focal plane but compromises bacterial viability.

In this study, we demonstrated the capability of the RBA platform to perform spectroscopic analysis of fully viable bacteria in suspension, enabling a simple and robust sample-to-result workflow. The focus is on rapid, phenotypic AST of bacteria while simultaneously enabling bacterial ID within the same platform. Samples of increasing complexity were compatible with the RBA, from the characterization of bacteria in a culture medium and laboratory environment, to the testing of artificial blood cultures, and finally the application to real patient blood cultures.

We evaluated the RBA platform using *Escherichia coli* and *Staphylococcus aureus*, two of the most frequently isolated pathogens from positive blood cultures. Bacteria were exposed to ciprofloxacin (fluoroquinolone) and oxacillin (β-lactam), respectively, two antibiotic classes against which clinically relevant resistance is present.^18^ Spectral data were analyzed using Partial Least Squares analysis to generate spectroscopic resistograms. The results demonstrate that a 90 min antibiotic exposure is sufficient to detect molecular signatures of antibiotic-susceptible strains with high accuracy. Furthermore, taxonomic ID at the family level was achieved in parallel without the need for additional instrumentation, measurements, or hands-on time. Integrating the RBA concept into clinical workflows has the potential to improve patient outcomes by enabling earlier, more targeted therapy, and strengthening rapid antibiotic stewardship.

## Methods

### Bacteria and Antibiotics

Two reference strains were used for AST: Gram-negative (*Escherichia coli*) ATCC 25922 and Gram-positive (*Staphylococcus. aureus*) ATCC 29213. Additionally, one susceptible (bk032337) and one resistant (bk032021) clinical *E. coli* isolate were analyzed. All strains were propagated in brain-heart infusion medium (BHI, brain heart infusion CM135, Oxoid) until the exponential phase, harvested, and then stored at −80 °C in BHI supplemented with 20% glycerol until use.

Ciprofloxacin (CIP; ciprofloxacin hydrochloride monohydrate, Sigma-Aldrich) and oxacillin (OXA; oxacillin Sodium Salt, Sigma-Aldrich) were used for treatment. To validate the RBA-AST concept, antibiotic concentrations were selected according to the MICs of the tested strains determined in the medium control (see Supporting Information 1). For each AST, four concentrations were used: two below the MIC_range_ (growth control and MIC–1) and two above the MIC_range_ (MIC+1 and MIC+5).

### Sample Matrices

The experimental setup was designed to demonstrate the feasibility of the platform for different sample origins, whereas downstream sample preparation, internal reference testing, and rapid AST were identical for all samples. Samples of increasing complexity included bacteria from medium controls, artificial blood cultures, and patient blood cultures. The generation of artificial blood culture flasks with donor blood and the use of patient blood cultures were approved by the ethics committee of the Jena University Hospital, Germany (UKJ-5292–10/17).

Medium controls were generated by cultivating stored bacteria and cultivating them in *antibiotic testmedium* 2 50% (AT2 50%, see Supporting Information 2) for 2–3 h.

Artificial blood cultures were prepared using Lytic Plus Anaerobic Flasks (Cat. No. 442265, Becton Dickinson) filled with approximately 5 mL of healthy donor blood under sterile conditions. The flasks were subsequently inoculated with 1 mL of *E. coli* or *S. aureus* (McFarland 0.5) under sterile conditions and placed in a BD BACTEC FM incubation system (Becton Dickinson GmbH, Germany). The addition of 1 mL of Dulbecco’s phosphate-buffered saline (PBS, ref. 14190–094, Gibco) served as a negative control. The flasks showed positive growth signals after approximately 3 h of incubation.

Patient blood culture flasks from the Institute of Medical Microbiology, Jena University Hospital, were obtained after positive growth signals were detected in the incubation system and standard procedures (ID, plating on appropriate media, VITEK 2 analysis) were initiated.

### Isolation of Bacteria from Blood Cultures

Blood culture samples were obtained from both artificial and patient blood cultures under sterile conditions. Blood culture material (3 mL) was mixed with 6 mL of BLINK erythrocyte lysis buffer (BEL buffer; BLINK AG, Germany)^19^ and incubated at room temperature for 5 min. Subsequently, the samples were centrifuged at 400 × *g* for 5 min, and the pellet was washed with one volume of the same buffer. After a second centrifugation step, the resulting colorless bacterial pellet was resuspended in AT2 50% medium. The bacterial load was determined both before and after lysis by plating on blood agar plates (Columbia agar with sheep blood plus, ref. PB5039A; Thermo Fisher Scientific). Colony-forming units per mL were quantified after overnight incubation at 37 °C.

### Bacteria–Antibiotic Interaction

Bacteria originating from all three sample matrices, medium control, and bacteria isolated from artificial and patient blood cultures were incubated in flasks with the corresponding antibiotic concentrations. Antibiotic concentrations were selected according to their respective MICs, which were determined by BMD within the medium control (see Supporting Information 1). Here, the experimental conditions used for the RBA differed from standard EUCAST AST conditions in several aspects. First, instead of cation-adjusted Mueller–Hinton broth recommended by EUCAST, AT2 50% medium was used (medium composition see Supporting Information 2), as it provided low fluorescence background and was therefore compatible with Raman spectroscopic measurements while simultaneously supporting bacteria–antibiotic interaction. Second, the interaction time was reduced to 90 min for all bacteria–antibiotic combinations, compared to the 18 ± 2 h incubation specified by EUCAST. Third, a higher bacterial inoculum of 2 × 10^8^ colony forming units (CFU)/mL was required for the RBA measurements compared to the standardized EUCAST inoculum of 5 × 10^5^ CFU/mL, in order to obtain sufficient bacterial enrichment and Raman signal intensity. Due to these deviations from standard EUCAST conditions, direct comparison between RBA-derived antibiotic concentrations and clinical breakpoint concentrations was not readily possible, requiring an adaptation of the tested RBA concentrations to enable direct comparison for patient blood cultures (see Supporting Information 10).

### Broth Microdilution

BMD was performed to validate the RBA results as an internal reference method for AST. For this a 96-well plate (Item No. 655161, Greiner Bio-One) was filled with bacteria–antibiotic suspensions from interaction flasks immediately before incubation for 90 min in a shaking incubator. For each antibiotic concentration, 3 × 200 μL was pipetted and a sterile control containing AT2 50% medium was added. Plates were incubated at 37 °C in ambient air, and optical density at 600 nm (OD_600_) was measured after 18 ± 2 h (EUCAST 2024). Bacterial growth was considered present if OD_600_ increased over time and absent if it remained below 50% of the growth control.

### RamanBioAssay-Platform for Rapid Antimicrobial Susceptibility Testing

The RBA chip was fabricated in-house at the Leibniz Institute of Photonic Technology’s cleanroom facility in Jena, Germany. The lithographic manufacture of RBA chips was carried out on carefully precleaned fused silica wafers (Nano Quarz Wafer GmbH, Germany) with a diameter of 100 mm and a thickness of 200 μm. These wafers served as substrates for the subsequent process steps. With a chip size of 25 × 55 mm^2^, three chips were manufactured in parallel per wafer (Supporting Information 3, Figure 1). The wafers were coated with AZ5214E JP photoresist (MicroChemicals GmbH, Germany) using a spin coating process, exposed using a mask aligner process, and developed in a resist image reversal process. This lift-off-capable resist mask was evaporated with 3 nm titanium +100 nm gold and then removed in a solvent bath (acetone and isopropanol rinse). The titanium served as an adhesive layer for the gold on the quartz. For separation, the manufactured RBA wafers were first coated with a protective AZ resist and then cut into individual chips using a wafer saw. In a final step, the chips were carefully stripped of the protective resist.

**Figure 1:**
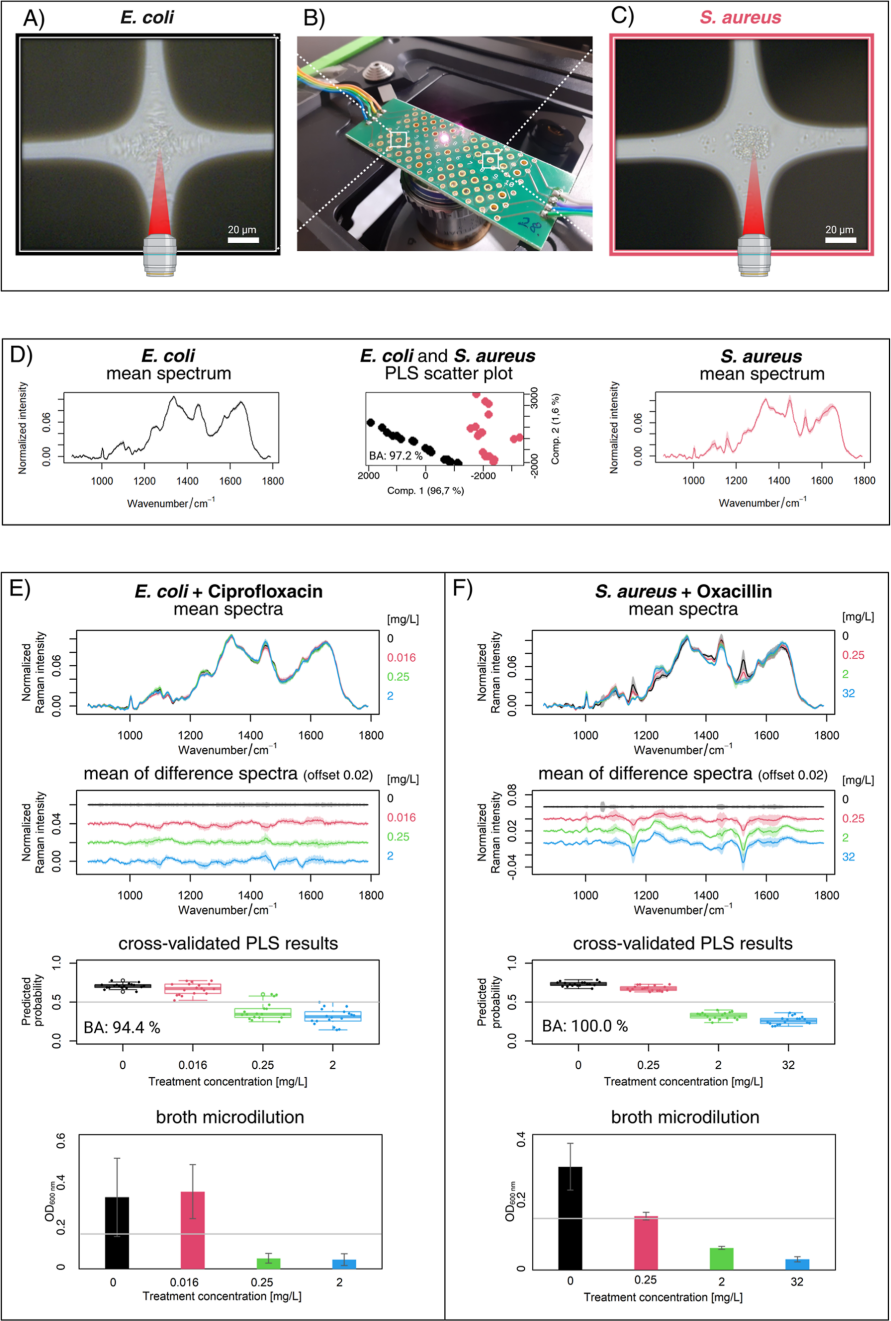
Concept of the RamanBioAssay^TM^ platform for rapid antimicrobial susceptibility testing with integrated bacterial identification capability. (A–C) Gram-negative *E. coli* (A) and Gram-positive *S. aureus* (C) are trapped within the dielectric field inside the RBA chip (B), as seen under the microscope. (D) Preprocessed Raman mean spectra of untreated *E. coli* (black) and *S. aureus* (red). Partial Least Squares (PLS) scatter plot shows clustering of *E. coli* (black dots) and *S. aureus* (red dots). Data from six technical replicates, three biological replicates. (E) *E. coli* is treated with ciprofloxacin at increasing concentrations. Difference spectra are generated by subtracting the growth control from treatment spectra, offset by 0.02 for visualization. A batch-wise cross-validated PLS model predicts growth with balanced accuracy (BA) of 94.4%. Broth microdilution (BMD) serves as internal reference. The gray line represents the 50% OD_600_ cutoff of the growth control used to distinguish growth from no growth. Four ciprofloxacin concentrations, six technical replicates, three biological replicates. (F) *S. aureus* is treated with oxacillin at increasing concentrations. Difference spectra generated as in (E), offset by 0.02. A batch-wise cross-validated PLS model predicts growth with BA of 100%. BMD serves as internal reference. The gray line represents the 50% OD_600_ cutoff of the growth control used to distinguish growth from no growth. Four oxacillin concentrations, six technical replicates, three biological replicates. Created in BioRender. Grohs, R. (2026) https://BioRender.com/rmwrgpb.

The RBA chips were assembled with a printed FR-4 circuit board (PCB) using precisely laser-cut double-sided adhesive films (120 μm thickness, BiFlow Systems GmbH, Germany) to mount the chip onto the PCB. The circuit board comprises 40 through-holes serving as sample cavities, 72 electrical vias, and eight solder pads. The vias were filled twice with electrically conductive silver paste to establish electrical contact between the PCB and the RBA chip. Two 4-pole ribbon cables with pin headers were soldered onto the PCB to enable connection to the DEP frequency generator (Supporting Information 3, Figure 2).

**Figure 2:**
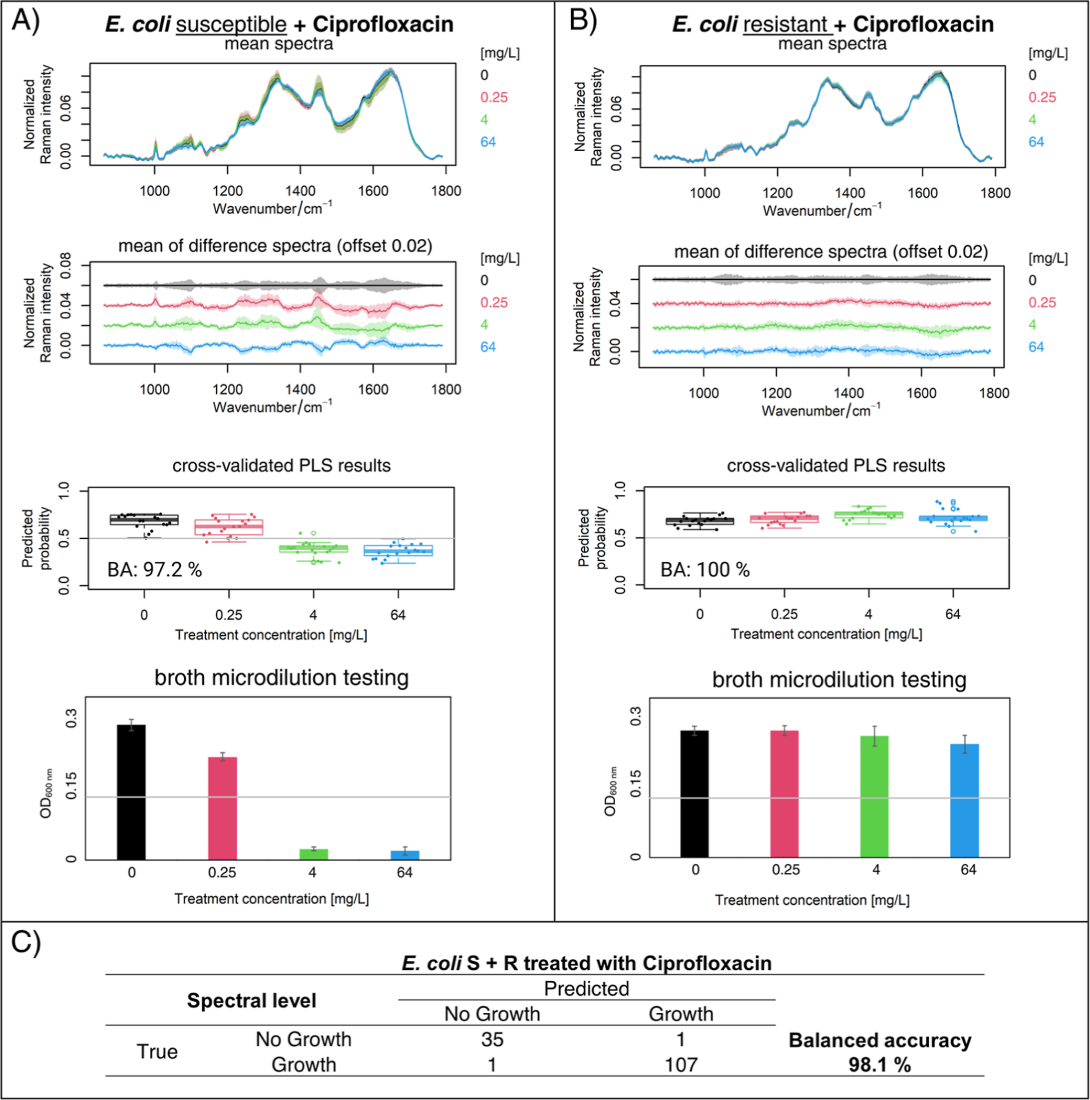
RamanBioAssay rapid antimicrobial susceptibility testing of susceptible and resistant clinical isolates. (A) *E. coli* (S) is treated with ciprofloxacin at increasing concentrations. Difference spectra are generated by subtracting the growth control from treatment spectra, offset by 0.02 for visualization. A batch-wise cross-validated PLS model predicts growth with balanced accuracy (BA) of 97.2%. Broth microdilution (BMD) serves as internal reference. The gray line represents the 50% OD_600_ cutoff of the growth control used to distinguish growth from no growth. Four ciprofloxacin concentrations, six technical replicates, three biological replicates. (B) *E. coli* (R) is treated with ciprofloxacin at increasing concentrations. Difference spectra generated as in (A), offset by 0.02. A batch-wise cross-validated PLS model predicts growth with BA of 100%. BMD serves as internal reference. The gray line represents the 50% OD_600_ cutoff of the growth control used to distinguish growth from no growth. Four ciprofloxacin concentrations, six technical replicates, three biological replicates. (C) Batch-wise cross-validated PLS model predicts bacterial growth from combined spectra of susceptible and resistant clinical *E. coli* isolates, achieving a BA of 98.1%. Created in BioRender. Grohs, R. (2026) https://BioRender.com/rmwrgpb.

The platform features a multiwell design with 40 cavities, where on-chip bacterial enrichment is achieved by negative dielectrophoresis (DEP). Viable bacteria were concentrated in each well, forming bacterial clouds with several hundred individual cells. Using DEP, only a small sample volume per well (4 μL of bacterial suspension, concentration c ≈ 2 × 10^8^ CFU/mL) was required to run the RBA. This reduced the required precultivation time and thereby shortened the total turnaround time. Each well can be loaded independently with a different sample, allowing for high flexibility and throughput for clinical applications. For example, one bacterium can be tested against several antibiotics at breakpoint concentrations for S/R classification, against fewer antibiotics at multiple concentrations for MIC determination, or multiple bacteria can be tested against a single antibiotic on the same chip. This allows AST panels to be adapted for operator requirements and updated according to the latest antimicrobial resistance (AMR) surveillance data. Owing to the scalability of the platform, the panel size is flexible. The RBA represents a hygienic, closed platform with no cross-contamination between wells, no leakage, and no direct contact between the measurement device (e.g., objective) and the infectious sample.

To validate the RBA-based AST concept, bacteria treated with various concentrations of antibiotics were used for ID and AST. After bacteria–antibiotic interaction in flasks, the bacterial suspensions were removed from the incubator, and the OD_600_ was measured for each flask. The optical densities were then adjusted to match a concentration of 2 × 10^8^ CFU/mL (OD_600_ ≈ 0.08). At least two RBA-wells per concentration were filled with 4 μL of OD-adjusted bacterial suspensions. The filled wells were sealed with a microplate seal (ThermalSeal RT, Excel Scientific Inc., USA), and the RBA was transported to the measurement device, placed inside the sample holder, and connected to the electrical interface. The DEP field was activated, and the voltage was adjusted to 5 V_peak‑to‑peak_ (AC, 1 MHz). To visually confirm the DEP-enrichment of bacteria, a filled well was inspected under the microscope. The DEP effect was observed by gradually trapping the bacteria inside the quadrupole structure (see Supporting Information 4). After 5–15 min, the bacterial clouds had enlarged to a diameter of ≥ 10 μm and were ready for measurement.

### Raman Spectroscopic Measurement

Raman spectroscopic measurements were performed using a confocal Raman microscope (inVia, Renishaw Plc, UK) with an inverted microscope setup (DMi8M, Leica, Germany). The 785 nm laser was focused onto the sample using a 63×/1.20 water-immersion objective (HC PL APO 63×/1.20 W CORR CS2 50634, Leica, Germany). The 180° backscattered light was collected by the same objective and passed through a 600 L/mm diffraction grating before reaching the detector (Centrus 2UM153; Renishaw, UK). The spectral resolution was ≤ 2 cm^–1^. Medium controls and artificial blood cultures were measured as biological replicates (batches) on three separate days, whereas patient blood cultures were measured once per patient. Two wells per concentration were measured as technical replicates, and three spectra per well were recorded. For additional measurement details and total number of spectra acquired, refer to Supporting Information 5.

### Data Analysis

Prior to analysis, the spectra were preprocessed by despiking based on the one-dimensional Laplacian,^20^ 4-acetamidophen wavenumber calibration,^21^ sensitive nonlinear iterative peak (SNIP) clipping baseline correction^22^ with smoothing at the first iteration, and vector normalization.

To differentiate the bacterial strains, Partial Least Squares (PLS) discriminant analysis was selected because of its robustness in handling collinear and high-dimensional spectral data, which is typical in Raman spectroscopy. PLS effectively reduces dimensionality while preserving the variance relevant to class separation, making it particularly suitable for data sets where the number of variables exceeds the number of samples. Its interpretability and compatibility with cross-validation strategies further support its use, making it one of the most widely applied methods in biomedical Raman spectral data analysis.^23^ To employ PLS regression for the classification task, two dummy response variables (0 and 1) corresponding to the respective strains were used as model responses. Model validation was performed using a leave-one-batch-out cross-validation approach,^24^ optimizing the number of latent variables (LV) in the range of 1–15. The preprocessed spectra of the two bacterial strains, together with the PLS plot visualizing intra- and interstrain variations, are shown in Figure 1D.

For AMR detection, batch-to-batch and strain-to-strain variation was minimized by centering the data relative to the untreated control of each biological replicate (batch). This was achieved by subtracting the mean of the preprocessed Raman spectra of the untreated growth control from the spectra collected for each batch.

The calculated difference spectra reflect how the spectral properties change as bacteria respond to antibiotic treatment. For antibiotic-resistant bacteria, the spectral difference from the control remains small, while significant changes occur for antibiotic-susceptible bacteria. Although this effect was visually observable, a PLS model was used to quantify the observed effect. The previously described leave-one-batch-out cross-validation strategy was applied to optimize the number of LV in the range up to 15. For AMR detection the “dummy” response values were set to indicate “growth” or “no growth” expected. Model performance was assessed by calculating the balanced accuracy as the average of the sensitivity values for both classes at the spectral level. The softmax function was applied to constrain the PLS model predictions to a range between 0 and 1. The resulting values are referred to in this paper as PLS-predicted probabilities.

## Results

### On-Chip Raman Spectroscopy for ID and AST

Initial validation of the on-chip Raman Spectroscopy platform’s dual functionality for simultaneous bacterial ID and AST was performed with quality control strains *E. coli* ATCC 25922 and *S. aureus* ATCC 29213 originating from medium controls, representing the simplest and most straightforward sample type. Bacteria were loaded into the RBA chip (Figure 1B) for bacterial enrichment using DEP. After 5–15 min, sufficiently sized bacterial clouds had formed (Figure 1A and C), which were measured by Raman spectroscopy using 785 nm laser excitation.

For RBA-ID Raman spectra of untreated growth controls of Gram-negative *E. coli* and Gram-positive *S. aureus* are used, mean spectra are shown in Figure 1D (left and right panels). In the scatterplot (middle), each data point represents one spectrum, with *E. coli* shown in black and *S. aureus* in red. Data were derived from three independent biological replicates and analyzed using PLS. The model shows a clear differentiation of both bacteria, confirming the RBA’s integrated bacterial ID capability, as both species form distinct clusters (Figure 1D, middle). No additional instrumentation or turnaround time was required, since the growth control Raman spectra already contained the relevant ID information. For the complete documentation of ID results, including those from artificial and patient blood cultures, see Supporting Information 6.

For RBA-AST, *E. coli* was treated with CIP and *S. aureus* with OXA in increasing concentrations. The bacterial Raman spectra of the growth control and treatment concentrations were recorded (mean spectra, Figure 1E,F, top). Difference spectra were calculated by subtracting the growth control Raman signal from the treatment spectra after a 90 min incubation period. Spectra that are closer to the growth control baseline correspond to subinhibitory concentrations, where the molecular signatures indicate expected bacterial growth. In contrast, more pronounced spectral differences indicate inhibitory concentrations, where bacteria are no longer expected to grow (Figure 1E,F, second from top). For the data analysis, supervised PLS models were trained, which required prior class assignments. In our study, the assignments were ‘growth’ and ‘no growth’ expected, according to internal BMD. The models were built and validated by batch-out cross-validation with three independent biological replicates, allowing for high internal validity. Difference spectra were used as the input for the PLS model for the differentiation of expected growth. The cross-validated PLS model results demonstrated that bacterial growth could be predicted with balanced accuracies of 94.4% and 100%, respectively (Figure 1E,F, second from bottom).

Treatment concentrations were selected based on the respective MICs to include two concentrations that allowed bacterial growth (growth control and subinhibitory) and two that inhibited growth (inhibitory and inhibition control) in BMD (Figure 1E,F, bottom). Spectroscopic results were concordant with this internal BMD, while RBA results were available up to 14.5 h earlier than those of BMD.

To exemplify the feasibility of RBA-AST to differentiate between antibiotic susceptible (S) and resistant (R) strains, two clinical *E. coli* isolates, one susceptible (bk032337) and one resistant (bk032021), were analyzed. Treatment concentrations were selected based on the MIC of the susceptible isolate to include two concentrations that allowed bacterial growth (growth control and subinhibitory) and two that inhibited growth (inhibitory and inhibition control). Resistant isolates were exposed to the same concentrations and exhibited sustained growth across all four concentrations. Spectroscopic results per isolate were concordant with internal BMD (Figure 2A,B). When combining the respective spectra of susceptible and resistant *E. coli* isolates, the cross-validated PLS model results demonstrated S/R differentiation with 98.1%.

### RamanBioAssay Platform Integration into Diagnostic Workflow for Positive Blood Cultures

Next, artificial blood cultures were examined to integrate the RBA platform into a sample-to-answer workflow for positive blood cultures, ensuring both matrix removal and immediate test readiness. Sterile blood cultures were inoculated with bacteria and incubated using the BD BACTEC FM system (Becton Dickson GmbH, Germany). Bacterial metabolism-induced increases in CO_2_ levels were detected via pH-dependent fluorescence, which initiated a positive flag. Positive artificial blood cultures were mixed with lysis buffer^19^ and centrifuged for bacterial isolation. High isolation efficiency was confirmed by plating, and CFU counting before and after isolation was used as a quality control for the sample preparation protocol (see Figure 3A and Supporting Information 7). Subsequently, the isolated bacteria were exposed to antibiotics for 90 min, followed by an RBA-based AST (Figure 3A).

**Figure 3:**
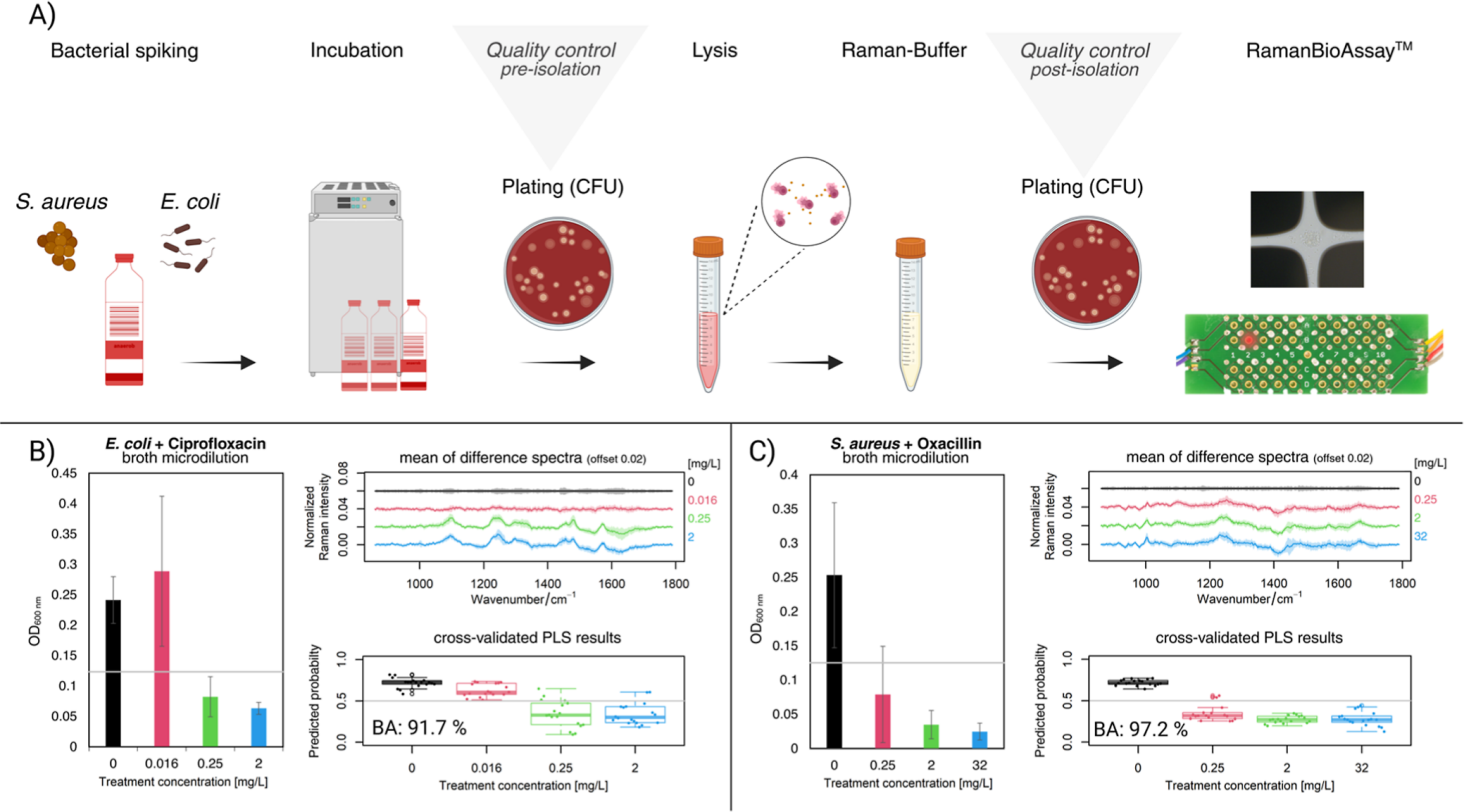
RamanBioAssay antimicrobial susceptibility testing from artificial blood cultures. (A) Experimental design outline. Artificial blood cultures are incubated until flagged positive, removed from the incubator and processed by lysis and centrifugation. High isolation efficiency is confirmed by CFU plating before and after bacterial isolation. Retrieved viable bacteria are incubated with antibiotics for 90 min prior to RamanBioAssay testing. (B) *E. coli* is treated with increasing ciprofloxacin concentrations. Raman difference spectra are generated and displayed with an offset of 0.02 for visualization. Difference spectra are analyzed using a Partial Least Squares (PLS) model to differentiate between the classes “growth” and “no growth” expected, achieving high balanced accuracy (BA). Broth microdilution (BMD) serves as internal reference. The gray line represents the 50% OD_600_ cutoff of the growth control used to distinguish growth from no growth. Six technical replicates, three biological replicates. (C) *S. aureus* is treated with increasing oxacillin concentrations. Raman difference spectra are generated as in (B) and analyzed using a PLS model, achieving high BA. BMD serves as internal reference. The gray line represents the 50% OD_600_ cutoff of the growth control used to distinguish growth from no growth. Six technical replicates, three biological replicates. Created in BioRender. Grohs, R. (2026) https://BioRender.com/rmwrgpb.

Using this bacterial isolation protocol, downstream bacteria–antibiotic interactions and subsequent spectroscopic ASTs were successfully performed for *E. coli* treated with CIP (Figure 3B) and *S. aureus* treated with OXA (Figure 3C), achieving high balanced accuracies of 91.7% and 97.2%, respectively. For the complete documentation of AST results and internal BMD from artificial blood cultures, see Supporting Information 8.

Compared to bacteria from medium controls, slight shifts in the MIC were observed in *S. aureus* isolated from blood cultures and treated with OXA, most likely due to isolation-related bacterial stress. Therefore, the subinhibitory assignment of OXA concentration 0.25 mg/L changed to an inhibitory, in accordance with BMD results. This effect was not observed in *E. coli* isolated from blood cultures treated with CIP (for further information, see Supporting Information 1).

### Validation of RamanBioAssay Using Real Blood Cultures

Building on the established workflow for artificial blood cultures, the adaptability of the RamanBioAssay platform for direct antimicrobial susceptibility testing of real patient blood cultures was evaluated, emphasizing immediate test readiness in a clinical context (Figure 4A). Therefore, positive blood cultures were divided into two parts. One portion was processed using the standard clinical ID and AST methods, while the other was retained for subsequent analysis. After confirming the presence of *E. coli* and *S. aureus* in the sample, the RamanBioAssay workflow was used for AST. This sequential approach ensured the workflow’s compatibility with real-world clinical scenarios while adhering to established validation protocols. Six monoinfected blood cultures from six different patients containing *E. coli* or *S. aureus* were processed. After isolation of the bacteria from the blood culture matrix, bacteria–antibiotic interactions occurred for 90 min, and RBA measurements were performed. The spectroscopic results were compared with those of routine clinical testing. PLS model predictions predominantly aligned with internal BMD results for four out of six blood cultures, while the other two samples (bk023067 and bk023014) required further investigation. A detailed look into the results of bk023067 (*E. coli* + CIP) revealed that for this specific strain the prediction of the two subinhibitory concentrations (CIP 0.002 mg/L and 0.03 mg/L) as “growth” expected was in accordance with the BMD. Whereas the spectra of bk023067, treated with the highest concentration (CIP 0.25 mg/L), were consistently mispredicted as “growth” expected in the Raman data, while this concentration showed no growth in BMD. Therefore, this strain was incorrectly classified as resistant. For bk023014 (*S. aureus* + OXA), the lowest concentration (OXA 0.5 mg/L) was consistently mispredicted as “no growth” expected, whereas signals of the higher concentrations (OXA 4 mg/L and 64 mg/L) yielded mixed predictions of “growth” and “no growth” expected, rendering the spectroscopic results nonconclusive. The mean turnaround time, measured from the completion of clinical ID until the resistogram result was available, was 3 h 6 min ± 24 min for the RBA (Figure 4B), compared to 13 h 54 min ± 4 h 31 min for the routine clinical AST. The total turnaround time, including blood culture incubation and clinical ID, was 14 h 34 min ± 6 h 38 min for the RBA and 51 h 26 min ± 10 h 06 min for the routine clinical workflow.

**Figure 4:**
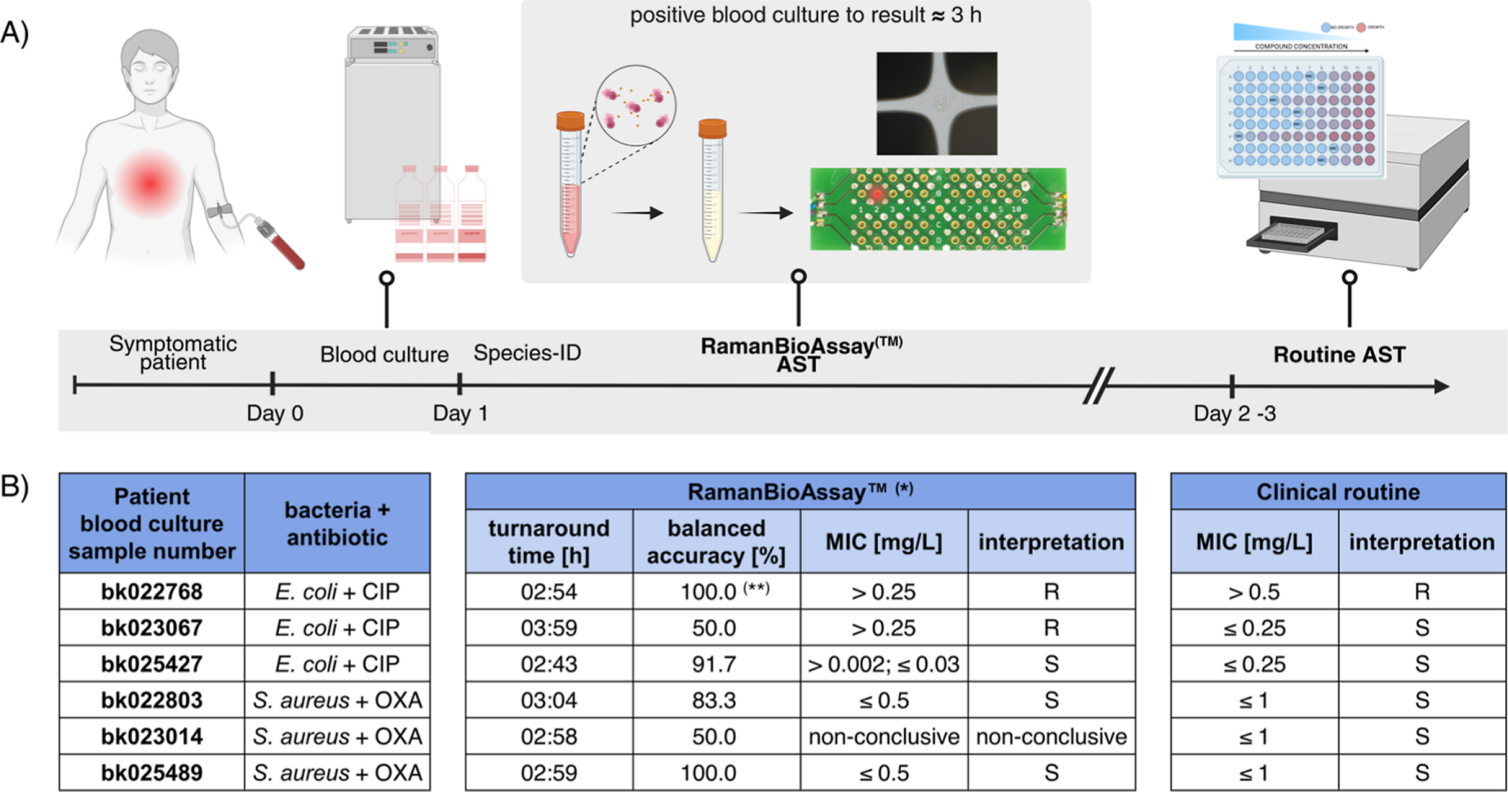
RamanBioAssay antimicrobial susceptibility testing from patient blood cultures. (A) Timeline from symptomatic patient with bloodstream infection to result. A patient blood sample is taken, blood culture bottles are inoculated, and incubated until flagged positive. A retention sample is stored and rapid species identification (ID) is performed by clinical routine (using loop-mediated isothermal amplification or mass spectrometry). Retention samples testing positive for *E. coli* or *S. aureus* are released and the RBA-AST workflow begins in parallel with routine culture-based AST. Spectroscopic results are therefore available hours to days before the clinical routine results. B) Minimal inhibitory concentrations (MICs) are determined according to internal broth microdilution (BMD) and RBA results. These MICs are compared to clinical routine testing, demonstrating the ability of the RBA to correctly classify antibiotic-resistant and -susceptible bacteria. The RBA predicts additional concentration ranges for MIC estimation when the MIC lies within its concentration range. Compared to internal BMD, susceptibility classification matches in 4/6 patient samples, mismatches in 1/6 samples, and is nonconclusive in 1/6 samples. (*) RBA-MICs and antibiotic concentrations are adjusted to match the EUCAST standards for direct comparability with routine clinical testing (see Supporting Information 10 for details). (**) bk022768 was resistant to ciprofloxacin at all RamanBioAssay concentrations. S/R···susceptible/resistant: susceptibility categories^25^ Created in BioRender. Grohs, R. (2026) https://BioRender.com/rmwrgpb.

For documentation of patient blood culture results, including AST, internal BMD, and turnaround times, see Supporting Information 9.

## Discussion

Evaluation of the RamanBioAssay platform focused on *E. coli* and *S. aureus*, which are the two most commonly isolated pathogens in positive blood cultures, demonstrating its potential for rapid phenotypic AST. These bacteria were chosen for their clinical relevance and technical challenges involved in developing a unified AST workflow applicable to both Gram-negative and Gram-positive pathogens. Bacteria were exposed to CIP (a fluoroquinolone) and OXA (a β-lactam antibiotic), for which resistance is increasingly prevalent and predicted to rise in the future.^26^ Susceptible bacterial strains exhibited distinct spectral changes compared to resistant strains, enabling the prediction of bacterial growth dynamics for up to 14.5 h before conventional growth-dependent assays (e.g., optical density measurements) could detect changes. Our results demonstrate that a mere 90 min interaction between bacteria and antibiotics is adequate to detect antibiotic-induced effects in susceptible strains via Raman spectroscopy and therefore differentiate susceptible from resistant bacteria. By integrating Raman spectroscopy with DEP trapping in a closed chip system, optimized sample preparation, and machine learning algorithms, we developed a holistic framework for Raman-based phenotypic AST of positive blood cultures. This multimodal approach enables the rapid and clinically relevant identification of antibiotic efficacy, overcoming the limitations of traditional methods that rely on prolonged bacterial growth. The combination of DEP trapping for cell enrichment in the laser focus, Raman spectroscopy for molecular fingerprinting, and machine learning for pattern recognition not only enhances the speed and accuracy of AST, but also supports the monitoring of microbial responses to therapeutic interventions. Previous DEP-Raman concepts^11,12^ were incompatible for use in clinical settings because the microscope objective was submerged in a bacteria-containing solution, raising safety concerns. To address this limitation, Raman measurements using the RBA were conducted for the first time inside a closed DEP-chip system. This innovation allowed the examination of bacteria without contamination risk by utilizing a laser through a quartz glass window. A workflow was developed to translate the critical steps of this unique technology into practical usability (for graphical representation of workflow and beam path, see Supporting Information 11).

The modular architecture of the RBA platform further supports scalability toward a broader range of clinically relevant pathogens and antibiotics. Because AST is performed in independently addressable wells, antibiotic panels can be flexibly adapted to different clinical requirements, resistance epidemiology, or organism-specific testing schemes. Future studies will therefore focus on expanding the spectral database and validating additional pathogens, as well as antibiotics with different mechanisms of action. Here, optimizations of bacteria–antibiotic interaction times, Raman spectroscopic measurement parameters and MIC-shifts compared to the EUCAST standard have to be considered. Since the spectroscopic workflow itself is independent of visible bacterial growth, the approach is expected to be transferable to a broad range of bacteria–antibiotic combinations after dedicated model training and validation.

In this study, we demonstrate for the first time that lysis of the blood culture matrix and isolation of viable bacteria (Gram-positive and Gram-negative) can be achieved in a way that enables downstream bacteria–antibiotic interactions and subsequent rapid Raman-based AST. The isolation of viable bacteria from a complex matrix is a critical prerequisite for the success of accelerated phenotypic AST, as it ensures the preservation of microbial viability and accuracy of molecular interactions. By successfully isolating viable bacterial cells from a complex environment, our approach not only facilitates the rapid detection of antibiotic effects, but also establishes a foundation for scalable, high-throughput clinical diagnostics. The RBA-AST protocol, when applied to artificial blood cultures, achieved high balanced accuracies of 91.7% for *E. coli* and 97.2% for *S. aureus*, validated against BMD as the reference standard. These results demonstrate the potential of the protocol to replicate the performance of established antimicrobial susceptibility testing methods. However, the application of this protocol provides critical insights into the behavior of bacteria with distinct morphochemical characteristics. Specifically, the Gram-positive *S. aureus* exhibited a MIC shift against OXA: a concentration of 0.25 mg/L was classified as inhibitory in both BMD and Raman-based AST after blood culture isolation (Figure 3C), yet it was subinhibitory in the medium control experiments (Figure 1F). In contrast, Gram-negative *E. coli* showed no such deviation, highlighting the differential impact of isolation-related stress on bacterial phenotypes (Figure 3B and Figure 1E). This observation underscores the necessity of minimizing isolation-induced stress to preserve full bacterial viability and ensure an accurate MIC classification. Careful documentation of these discrepancies not only strengthens the reliability of the RBA-AST protocol but also emphasizes the importance of tailoring isolation techniques to account for the diverse responses of bacteria with varying properties. Future work will focus on optimizing isolation strategies to enhance the precision of phenotypic AST outcomes across a broader range of pathogens.

The gold standard and routine clinical AST procedures are conducted under strictly standardized conditions, ensuring reproducibility, reliability, and regulatory compliance across laboratories. MIC breakpoints are governed by multinational organizations, such as the EUCAST.^25^ While the RBA platform can provide rapid, phenotypic AST, it is not yet fully compatible with EUCAST standard conditions, differing in medium composition and bacterial inoculum (see Supporting Information 10). Therefore, RBA-MICs and breakpoints are not directly transferable from the gold standard or routine clinical tests. Operating procedures were required to account for the bacteria–antibiotic-dependent MIC shifts. Therefore, BMD was performed for each experiment using RBA conditions to internally validate the spectroscopic results. Further technological improvements will aim to harmonize the RBA conditions with the EUCAST conditions so that AST results are easily and directly transferable, without the need to adjust for MIC shifts. This includes switching to Mueller-Hinton medium for bacteria–antibiotic interactions and adjusting the spectroscopic readout to be compatible with this medium. While the required volume of bacterial suspension for the RBA is already very small, the enrichment efficiency must be further increased to be compatible with a low inoculum of 5 × 10^5^ CFU/mL.

A key highlight of this study is the evaluation of the RBA-AST protocol using real patient blood cultures, which introduced an unprecedented level of complexity compared with standardized artificial models. Although the number of patient blood cultures analyzed was limited, the successful measurements demonstrated the RBA’s direct readiness for AST from real-world samples and should therefore be considered as a first feasibility study, laying the foundation for larger and more diverse clinical AST trials. Unlike controlled environments, clinical samples inherently reflect the heterogeneity and variability of human biology, including individual differences, such as unknown disease states, comedications, and prior antibiotic exposure. These factors can significantly influence AST outcomes, making method validation particularly challenging in real-world settings. To investigate the impact of such variables on our workflow and technological implementations, we again focused on model organisms, *E. coli* under CIP treatment and *S. aureus* under OXA treatment, which were previously characterized in controlled experiments. In contrast to the artificial blood cultures and medium control groups, which were analyzed using three biological replicates and validated using batch-out cross-validation with three independent biological replicates, allowing for high internal validity, patient samples were measured only once. Directly after isolating bacteria from positive blood cultures and without subcultivation, we observed greater variability owing to the unpredictable nature of clinical contexts. RBA-AST results for *E. coli* and *S. aureus* isolates from patient blood cultures were predicted with high accuracy using Raman data, achieving correct classification for two out of three *E. coli* and two out of three *S. aureus* isolates (Figure 4B). Despite these challenges, the RBA-AST approach still yields meaningful results, demonstrating its potential for translational applications. For implementation in routine clinical diagnostics, not only the DEP-chip platform itself but also cost-effective and robust Raman microspectrometers with automated measurement workflows and integrated data analysis will be required. Recent advances in optical engineering and device miniaturization have already enabled the development of compact and comparatively low-cost Raman systems while retaining high analytical performance.^27^ These developments support the long-term feasibility of translating Raman-based AST platforms into broader clinical use.

While this study primarily demonstrates the technical integration of the RBA into a sample-to-answer workflow, future clinical studies will include substantially larger and more diverse patient cohorts to improve the robustness and generalizability of the underlying spectral database. In particular, expanded data sets comprising susceptible and resistant clinical isolates across different bacteria–antibiotic combinations will be required to better capture biological variability encountered in routine diagnostics. Future validation strategies will include strict separation of independent training and test data sets to reduce the risk of overfitting and to further strengthen the clinical validity of the spectroscopic models. In addition, increasing attention will be paid to preanalytical variables, including sample handling, gentler bacterial isolation, and prior antibiotic exposure, which may influence Raman-based phenotypic responses. These measures are expected to reduce mismatches and nonconclusive classifications observed in this feasibility study and support the development of robust, clinically adaptable Raman-based AST workflows. Future model implementations may additionally incorporate confidence-based classification thresholds to identify uncertain predictions and minimize false susceptibility categorization.

Our findings emphasize the need to develop robust, adaptable Raman-based diagnostics capable of handling the inherent variability of clinical samples.

## Conclusion and Summary

The aim of this study was to systematically evaluate all technological parameters by leveraging a limited panel of bacteria and isolates with manageable biological variance to identify which aspects of our workflow can be controlled through a deeper understanding of the method and which sources of variation are inherently biological. Based on these insights, the technological processes in the protocol can be refined so that limitations due to biological variations become increasingly easier to handle.

The successful AST of the model bacteria with different morphologies and antibiotics with different modes of action underscores the RBA concept’s transferability to diverse bacterial species and antibiotic classes. The RBA-AST panel will be expanded to include additional bacteria–antibiotic combinations in the future. Stepwise evaluation across samples of increasing complexity, starting with medium controls, progressing to artificial blood cultures, and finally real patient samples, demonstrated the system’s potential for reaching Technology Readiness Level (TRL) 6 (prototype demonstration in a relevant application environment), representing a significant milestone toward clinical implementation. The demonstrated capability of the RBA platform to analyze real-world patient samples paves the way for future clinical trials. The insights gained from these trials will facilitate further technological advancements toward system prototyping and qualification.

Future developments of RBA will focus on achieving additional time savings by enabling the earliest possible harvesting of bacteria directly from blood cultures. Because the RBA DEP-chip requires only minimal bacterial quantities for analysis, far fewer than those found in fully incubated cultures or needed for accelerated conventional testing, this approach should allow sampling at a much earlier stage. As a result, the overall diagnostic turnaround time can be significantly reduced by shortening the incubation period of the preceding blood culture incubation period. The long-term goal is to match the accuracy of routine diagnostics while maintaining a significant advantage in turnaround time, ultimately enabling rapid, reliable, and resource-efficient antimicrobial stewardship in clinical settings.

## Supplementary Material



## Data Availability

Spectroscopic data, including associated metadata files, are available from the corresponding author upon reasonable request. Detailed information on the methodology, data analysis results, and clinical reference testing is provided in Supporting Information S1–S11.

## Notes

RamanBioAssay antibiotic concentrations used for on-chip spectroscopic AST (PDF) SI 2 Characteristics of Raman compatible culture medium AT2 50% (PDF) SI 3 Details of RamanBioAssay platform (PDF) SI 4 Enrichment of bacteria inside the RamanBioAssay by negative DEP (MP4) SI 5 Raman spectroscopic measurement parameters, number of Raman spectra and measurement overview (PDF) SI 6 Comprehensive spectroscopic data analysis: bacterial identification (PDF) SI 7 CFU counting pre and post bacterial isolation from blood cultures (PDF) SI 8 Comprehensive spectroscopic data analysis: AST from artificial blood cultures (PDF) SI 9 Comprehensive spectroscopic data analysis: AST from patient blood cultures (PDF) SI 10 Adaptation of RBA concentrations to be directly comparable to standard EUCAST concentrations (PDF) SI 11 Graphical representation of workflow and beam path in DEP-Chip (PDF)

## Notes

The generation of artificial blood culture flasks with donor blood and the use of patient blood cultures were approved by the ethics committee of Jena University Hospital, Germany (UKJ-5292–10/17).
