# Supplementary material for: On-Chip Raman Spectroscopy for Rapid Antimicrobial Susceptibility Testing from Blood Cultures
Source: Anal Chem. 2026 Jun 22;98(30):22188–200. doi: 10.1021/acs.analchem.6c01690 (PMC13449216; doi:10.1021/acs.analchem.6c01690)
Supplement: Supplementary file 1 [file ac6c01690_si_001.pdf]

Supporting Information

On-Chip Raman spectroscopy for Rapid Antimicrobial Susceptibility Testing from Blood Cultures

Richard Grohs[1,2,3], Franziska Hornung[4], Marie-Luise Enghardt[1,2,3], Anja Silge[1,2,3], Uwe Glaser[1], Uwe Hübner[1], Stefanie Deinhardt-Emmer[4], Oleg Ryabchykov[1,3,5], Karina Weber[1], Thomas Bocklitz[1,3], Bettina Löffler[4], Jürgen Popp * [1,2,3]

[1] Leibniz Institute of Photonic Technology, Member of Leibniz Health Technologies, Member of the Leibniz Research Alliance INFECTIONS, Member of the Leibniz Centre for Photonics in Infection Research (LPI), Albert-Einstein-Straße 9, 07745 Jena, Germany

[2] InfectoGnostics Research Campus Jena, Center of Applied Research, Philosophenweg 7, 07743 Jena, Germany

[3] Institute of Physical Chemistry and Abbe Center of Photonics, Friedrich Schiller University Jena, Member of the Leibniz Centre for Photonics in Infection Research (LPI), Helmholtzweg 4, 07743 Jena, Germany

[4] Institute of Medical Microbiology, Jena University Hospital, Am Klinikum 1, 07747 Jena, Germany

[5] Biophotonics Diagnostics GmbH, Am Wiesenbach 30, 07751 Jena, Germany

Correspondence:

Prof. Dr. Jürgen Popp

Institute of Physical Chemistry, Friedrich Schiller University Jena

Helmholtzweg 4, 07743 Jena, Germany

E-Mail: juergen.popp@uni-jena.de

Table of contents

SI 1 RamanBioAssay™ antibiotic concentrations used for on-chip spectroscopic AST (PDF)

SI 2 Characteristics of Raman compatible culture medium AT2 50% (PDF)

SI 3 Details of RamanBioAssay platform (PDF)

SI 4 Bacterial enrichment of bacteria inside the RamanBioAssay™ by negative DEP (MP4)

SI 5 Raman spectroscopic measurement parameters, number of Raman spectra and measurement overview (PDF)

SI 6 Comprehensive spectroscopic data analysis: bacterial identification (PDF)

SI 7 CFU counting pre and post bacterial isolation from blood cultures (PDF)

SI 8 Comprehensive spectroscopic data analysis: AST from artificial blood cultures (PDF)

SI 9 Comprehensive spectroscopic data analysis: AST from patient blood cultures (PDF)

SI 10 Adaptation of RBA concentrations to be directly comparable to standard EUCAST concentrations (PDF)

SI 11 Graphical representation of workflow and beam path in DEP-Chip (PDF)

SI 1: RamanBioAssay™ (RBA) concentrations

To determine MIC for bacteria-antibiotic combinations broth microdilution testing was conducted. Conditions were those of the medium control group within the RBA.

- medium: AT2 50 %
- inoculum: CFU of $2 * 10^8\ ml^{-1}$ ($OD_{600\ nm}$ ≈ 0.08)
- t = 22 ± 2 h
- antibiotic concentrations: 0 – 16 (521) mg/L
- 96-well plate, 160 rpm, 37 °C, sealed with lid, ambient air

MIC_{ranges}, as normally stated for standard AST results, were derived from BMD under RBA conditions as follows:

- *E. coli* + CIP: uneven range (e.g. 0.004-0.016 mg/L)[1] → MIC is in the middle value
    - RBA-MIC as by BMD = 0.06 mg/L
        - **RBA-MIC_{range} = 0.03 – 0.125 mg/L CIP**
- *S. aurues* + OXA: even range (e.g. ≤ 0.25 – 0.5 mg/L)[2] → MIC is the lower value
    - RBA-MIC as by BMD = 0.5 mg/L
        - **RBA-MIC_{range} = 0.5 -1 mg/L OXA**

1. E. coli ATCC 25922 + Ciprofloxacin

1.1. broth microdilution testing (medium control)

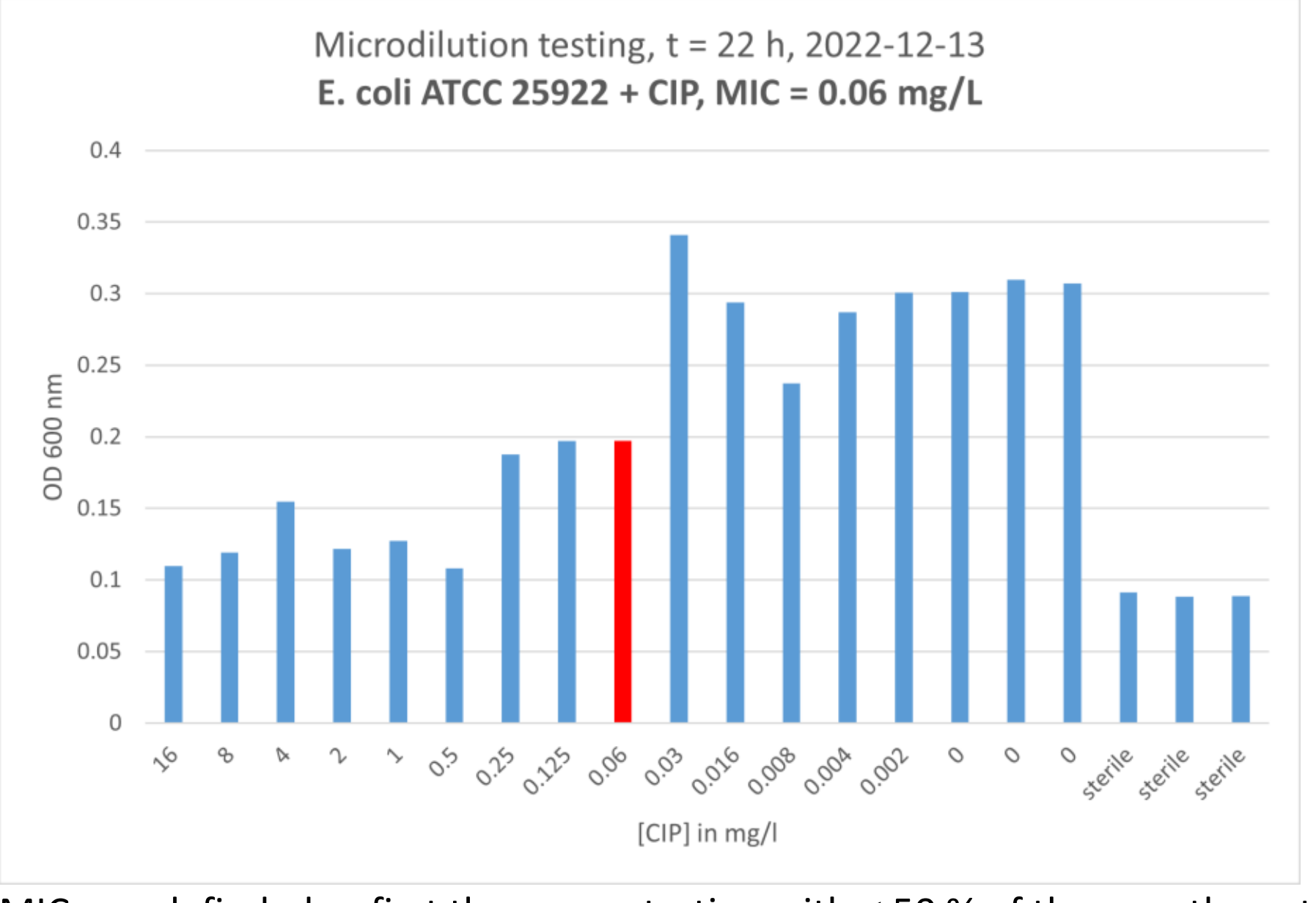

MIC was definded as first the concentration with < 50 % of the growth control's value (sterile subtracted).

1.2. RBA-concentrations for AST (medium control, blood cultures: artificial and patient)

RBA-concentrations in $\frac{mg}{L}$			
GC	$\mathrm{MIC}_{range} - 1$	$\mathrm{MIC}_{range} + 1$	$\mathrm{MIC} + 5$
0	0.016	0.25	2

growth: yes	growth: no

GC … growth control

MIC_{range} -1 … subinhibitory

MIC_{range} +1 … inhibitory

MIC + 5 … inhibition control

MICrange = 0.03 – 0.125 mg/L

2. E. coli bk032337 (S) + Ciprofloxacin

2.1. broth microdilution testing (medium control)

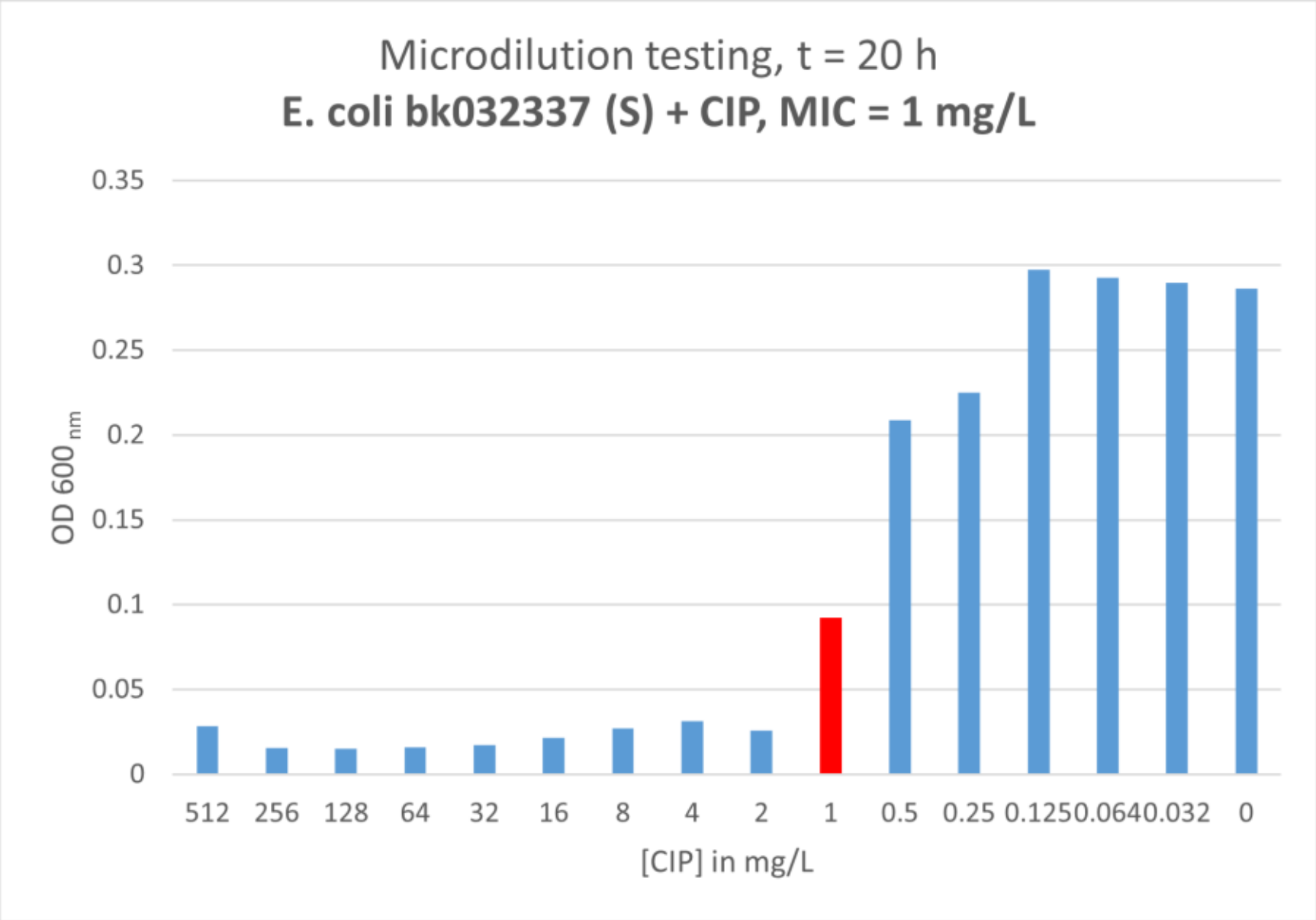

MIC was definded as first the concentration with < 50 % of the growth control's value (sterile subtracted).

2.2. RBA-concentrations for AST (medium control)

RBA-concentrations in $\frac{mg}{L}$			
GC	$\mathrm{MIC}_{range} - 1$	$\mathrm{MIC}_{range} + 1$	$\mathrm{MIC} + 5$
0	0.25	4	64

growth: yes	growth: no

GC … growth control

MIC_{range} -1 … subinhibitory

MIC_{range} +1 … inhibitory

MIC + 5 … inhibition control

MICrange = 0.5 – 2 mg/L

3. E. coli bk032021 (R)+ Ciprofloxacin

3.1. broth microdilution testing (medium control)

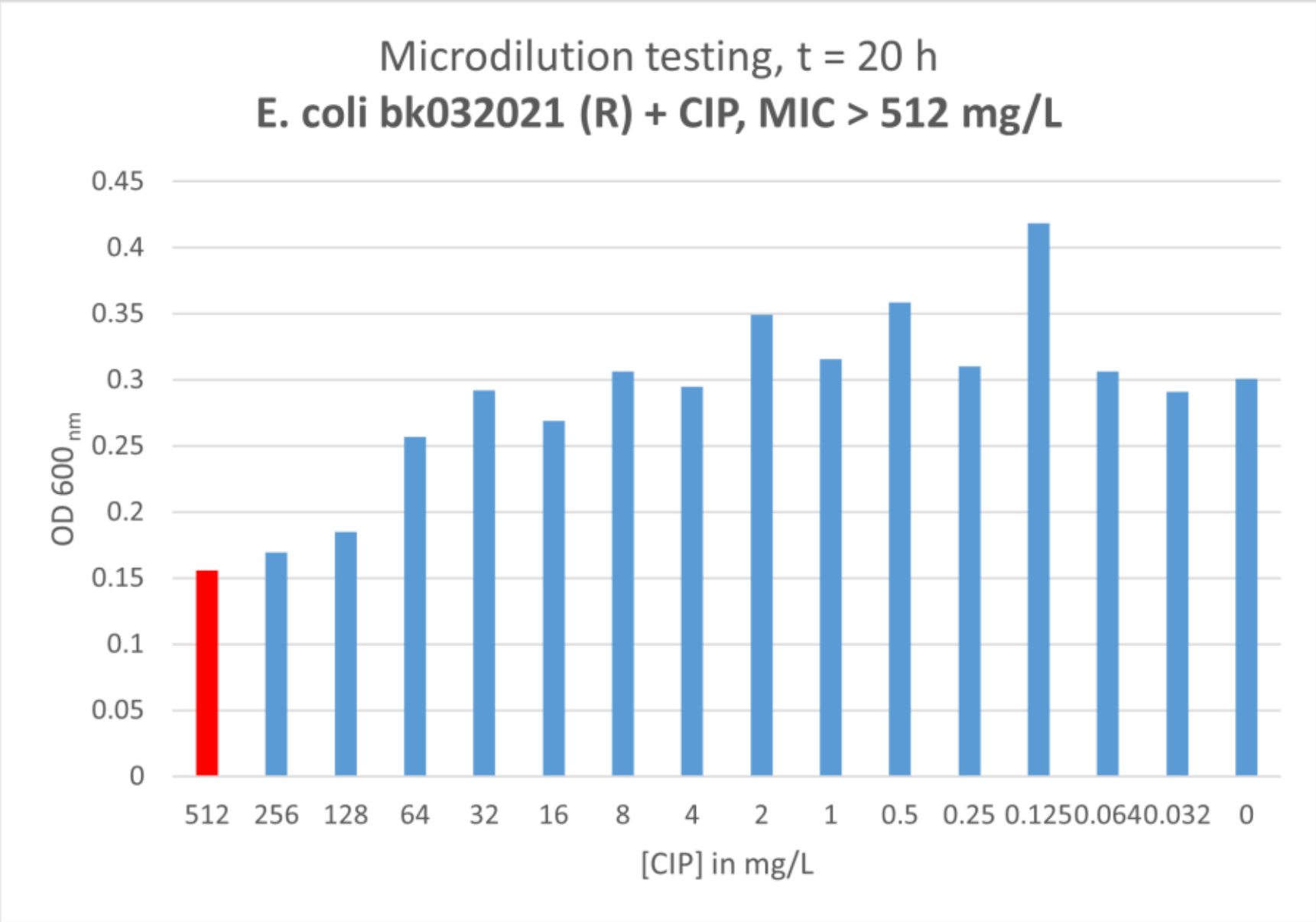

MIC was definded as first the concentration with < 50 % of the growth control's value (sterile subtracted).

3.2. RBA-concentrations for AST (medium control)

RBA-concentrations in $\frac{mg}{L}$			
GC	$MIC_{range} - 1$	$MIC_{range} + 1$	$MIC + 5$
0	0.016	4	64

growth: yes

GC ... growth control

MIC_{range} -1 ... subinhibitory

MIC_{range} +1 ... inhibitory

MIC + 5 ... inhibition control

MICrange > 512 mg/L

4. S. aureus ATCC 29213 + Oxacillin

4.1. broth microdilution testing (medium control)

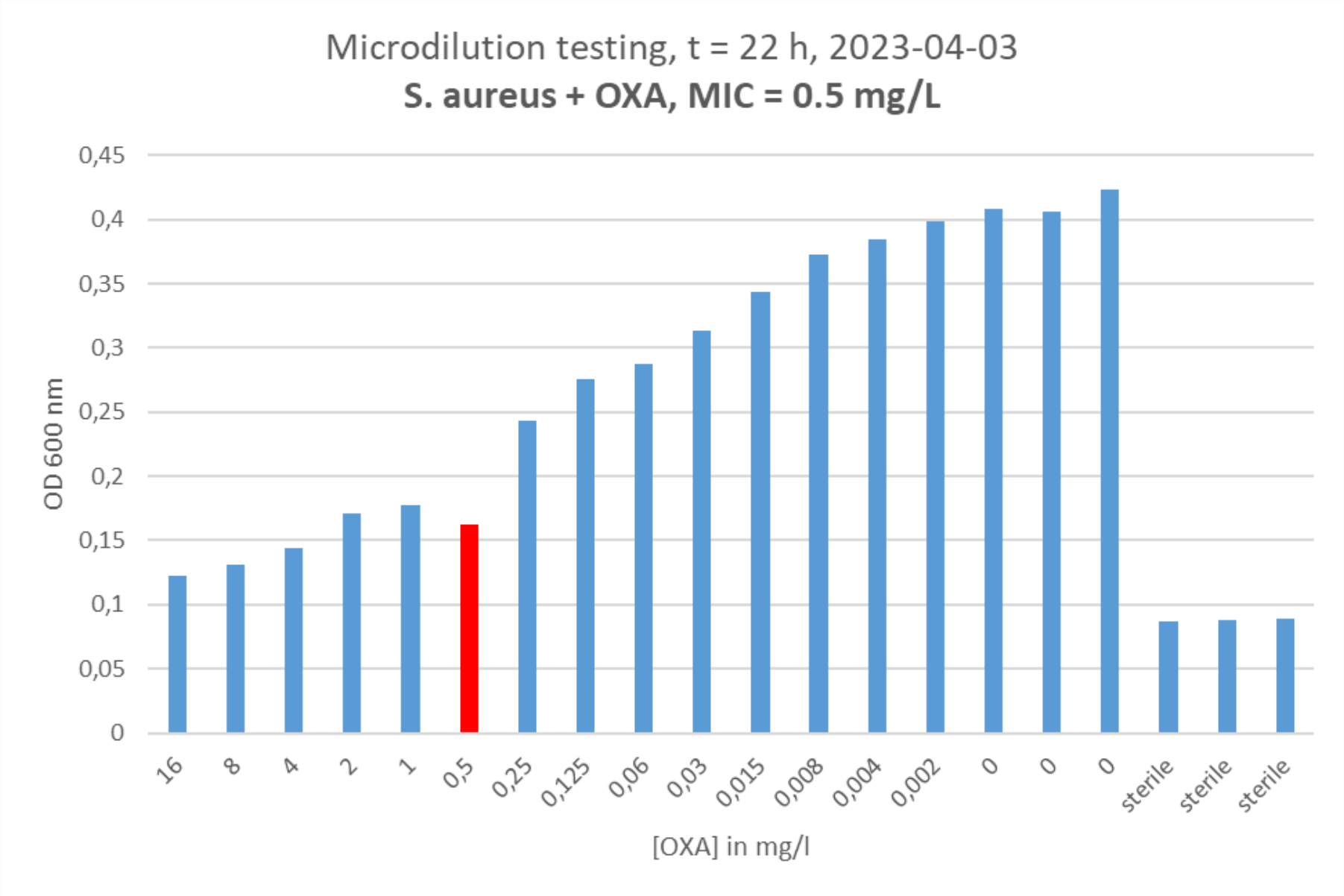

MIC was definded as first the concentration with < 50 % of the growth control's value (sterile subtracted).

4.2. RBA-concentrations for AST (medium control)

RBA-concentrations in $\frac{mg}{L}$			
GC	$\mathrm{MIC}_{range} - 1$	$\mathrm{MIC}_{range} + 1$	$\mathrm{MIC} + 5$
0	0.25	2	32

growth: yes	growth:no

GC ... growth control

MICrange -1 ... subinhibitory

MIC_{range} +1 ... inhibitory

MIC + 5 ... inhibition control

MICrange = 0.5 - 1 mg/L

4.3. RBA-concentrations for AST: S. aureus (artificial and patient blood culture) → MIC-shift post isolation

For isolation of *S. aureus* form blood cultures, a shift in RBA-MIC for oxacillin occurred, most likely due to centrifugation-induced bacterial stress. The MIC shifted by two 2-fold dilution steps resulting in a MIC_{range} of ≤ 0.125 – 0.25 mg/L. Since the same RBA-concentrations were used for medium control and blood culture experiments the former subinhibitory $MIC_{range} - 1$ was now inhibitory, too. This blood culture isolation-dependent MIC shift effect did not occur for *E. coli* + ciprofloxacin.

RBA-concentrations in $\frac{mg}{L}$			
GC	$MIC_{range} - 1$	$MIC_{range} + 1$	$MIC + 5$
0	0.25	2	32

growth: yes	growth: no

GC … growth control

MIC_{range} -1 … subinhibitory

MIC_{range} +1 … inhibitory

MIC + 5 … inhibition control

MICrange ≤ 0.125 – 0.25 mg/L

References

1. EUCAST. Routine and extended internal quality control for MIC determination and disk diffusion as recommended by EUCAST. Accessed November 11, 2024. https://www.eucast.org/fileadmin/src/media/PDFs/EUCAST_files/QC/v_14.0_EUCAST_QC_tables_routine_and_extended_QC.pdf
2. Institute of Medical Microbiology, Jena University Hospital. *QC Laboratory Report: Antibiotic Susceptibility Testing of ATCC 29213*; 2023. Accessed June 25, 2025.

SI 2: AT2 50% medium

- Low fluorescence background
- Low intrinsic Raman signal
- Enables fast bacterial growth

compound
Glucose
NaCl
Tryptone-Peptone
$MgSO_4$
KCl
KH_2PO_4
$NaHPO_4$-H_2O

SI 3: RamanBioAssay platform

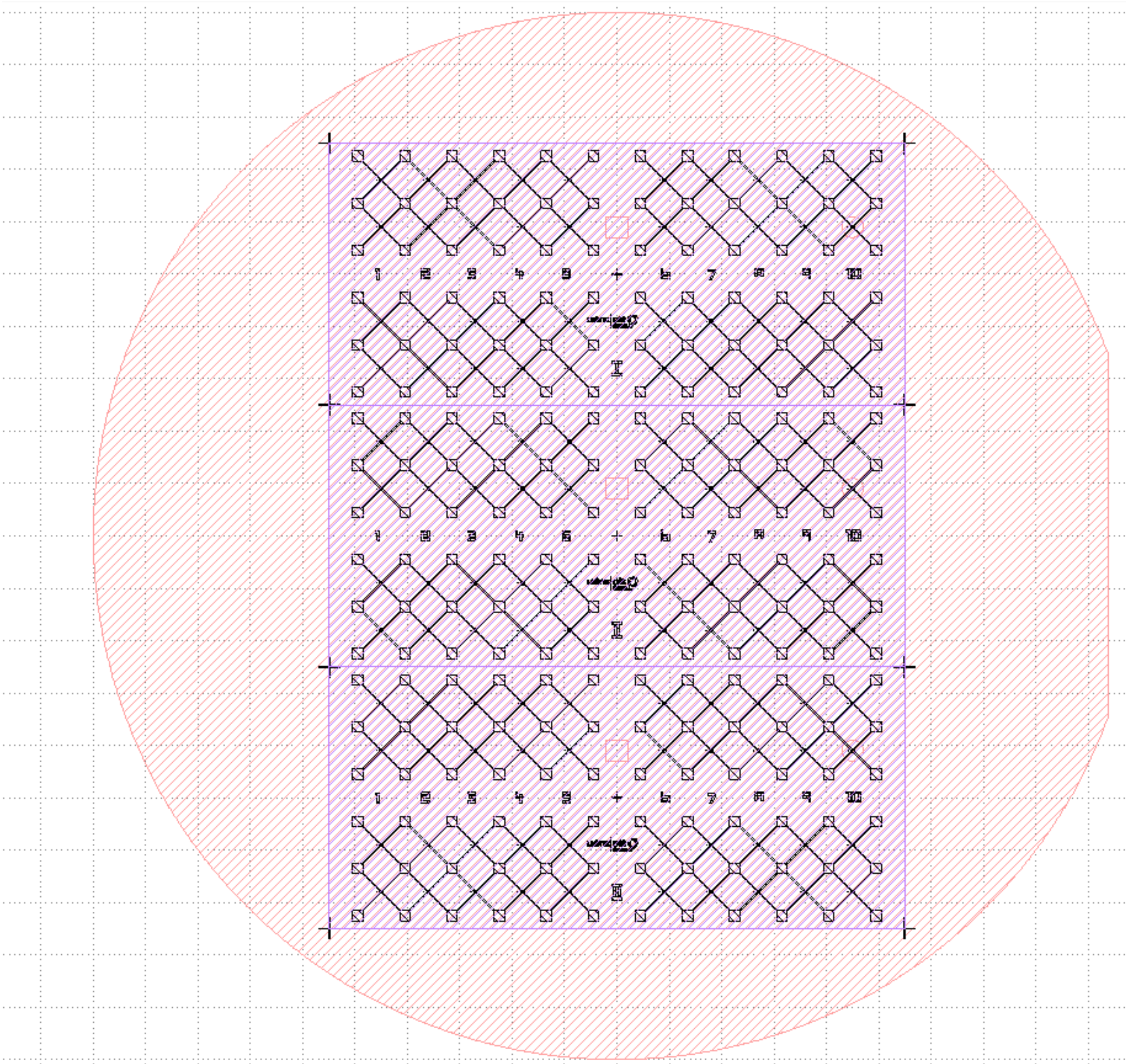

Figure 1: Layout of 100 mm wafer with 3 RBA chips measuring 25 x 55 mm^2

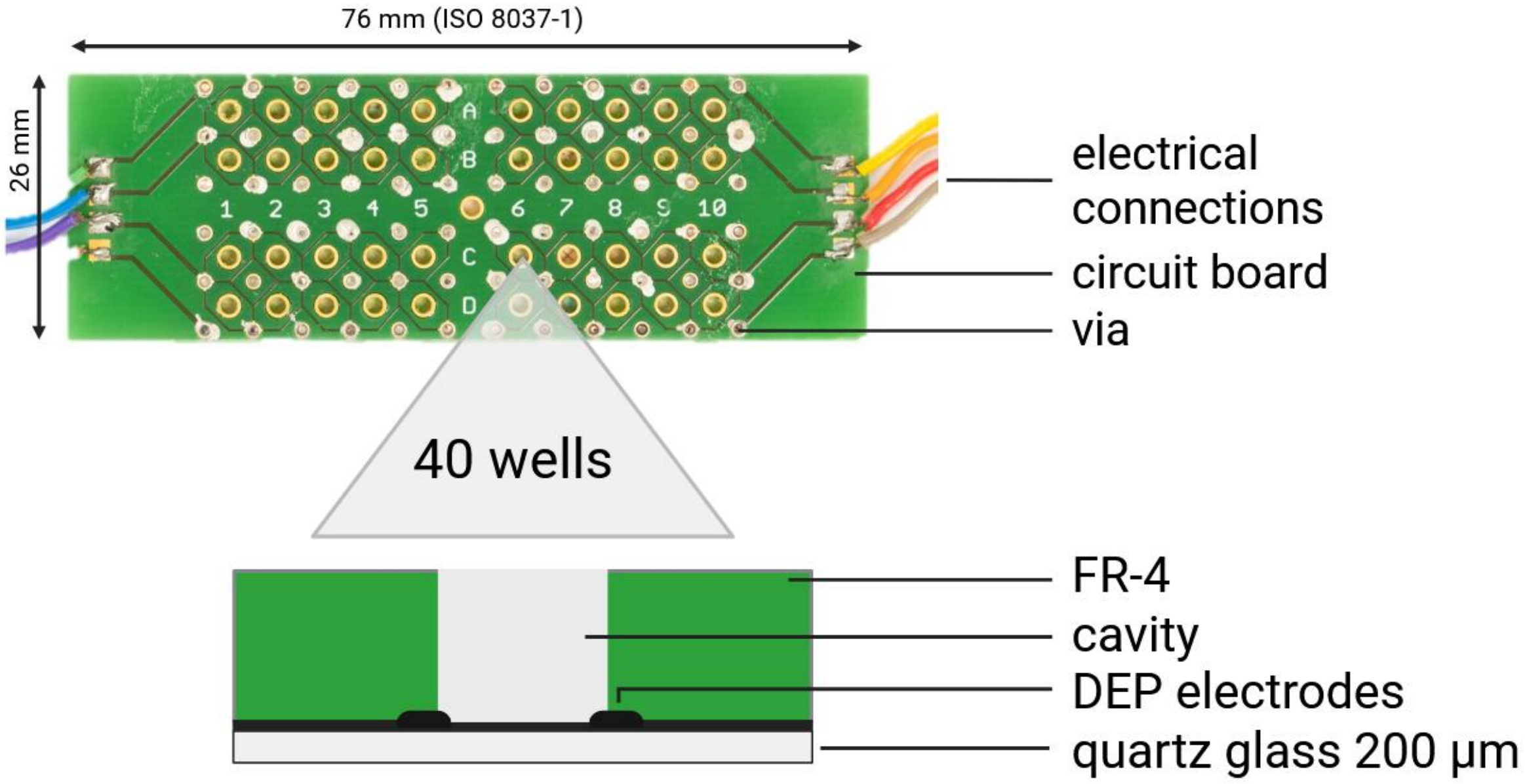

Figure 2: Details RamanBioAssay™ chip, Top view (photo) and shematic cross-section of a single well

SI 5: RBA Measurement parameters

**wavelength**	**laser, max. power source**	**laser power, after objective**	**grating**	**measurement parameters** integration time per single spectrum, laser power (source), spectral range, number of spectra per well, preburning time	
				E. coli	S. aureus
785 nm	diode laser, 300 mW	≈ 60 mW	600 l/mm	20 s, 300 mW, 500-2400 cm^{-1}, 3, no	20 s, 300 mW, 500-2400 cm^{-1}, 3, 20 s

- Pinhole out
- Normal confocality

Measurement overview and number of Raman spectra

Sample origin	Bacteria	Antibiotic concentrations tested	Technical replicates	Biological replicates	Single spectra
Medium control	*E. coli*<br>ATCC 25922<br>= 1	Ciprofloxacin<br>0/ 0.016/ 0.25/ 2 mg/L<br>= 4	2 wells * 3 spectra<br>= 6	3	72
	S. aureus<br>ATCC 29213<br>= 1	Oxacillin<br>0/ 0.25/ 2/ 32<br>= 4	2 wells * 3 spectra<br>= 6	3	72
	E. coli isolate (S)<br>bk032337<br>= 1	Ciprofloxacin<br>0/ 0.25/ 4/ 64 mg/L<br>= 4	2 wells * 3 spectra<br>= 6	3	72
	E. coli isolate (R)<br>bk032021<br>=1	Ciprofloxacin<br>0/ 0.25/ 4/ 64 mg/L<br>= 4	2 wells * 3 spectra<br>= 6	3	72
Artificial blood culture	*E. coli*<br>ATCC 25922<br>= 1	Ciprofloxacin<br>0/ 0.016/ 0.25/ 2 mg/L<br>= 4	2 wells * 3 spectra<br>= 6	3	72
	S. aureus<br>ATCC 29213<br>= 1	Oxacillin<br>0/ 0.25/ 2/ 32<br>= 4	2 wells * 3 spectra<br>= 6	3	72
Patient<br>blood culture	*E. coli*<br>bk022768<br>bk023067<br>bk025427<br>= 3	Ciprofloxacin<br>0/ 0.016/ 0.25/ 2 mg/L<br>= 4	2 wells * 3 spectra<br>= 6	1	72
	S. aureus<br>bk022803<br>bk023014<br>bk025489<br>= 3	Oxacillin<br>0/ 0.25/ 2/ 32 mg/L<br>= 4	2 wells * 3 spectra<br>= 6	1	72
					576

Infecto_Xplore

```
## Culture: short culture
## Laser: 785
```

```
## short culture 785 2023-01-11_MP_Ec+CIP_spectra_RG
```

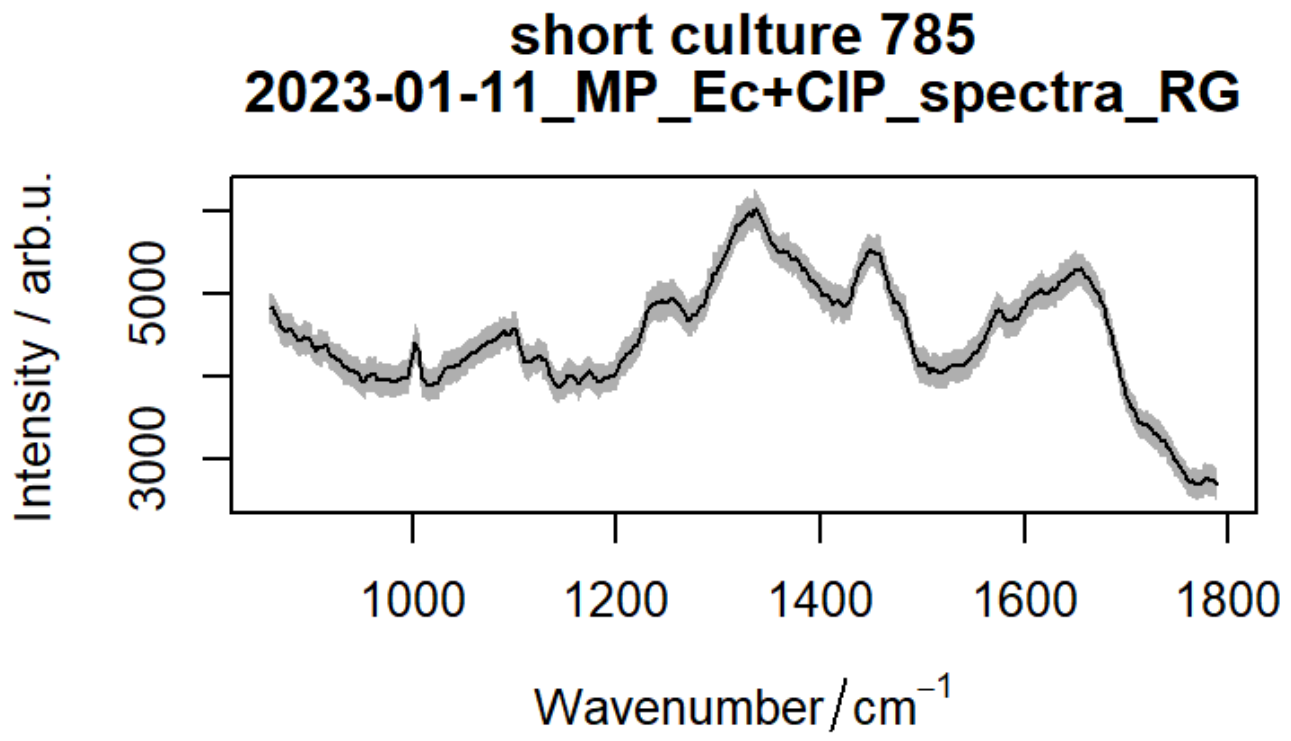

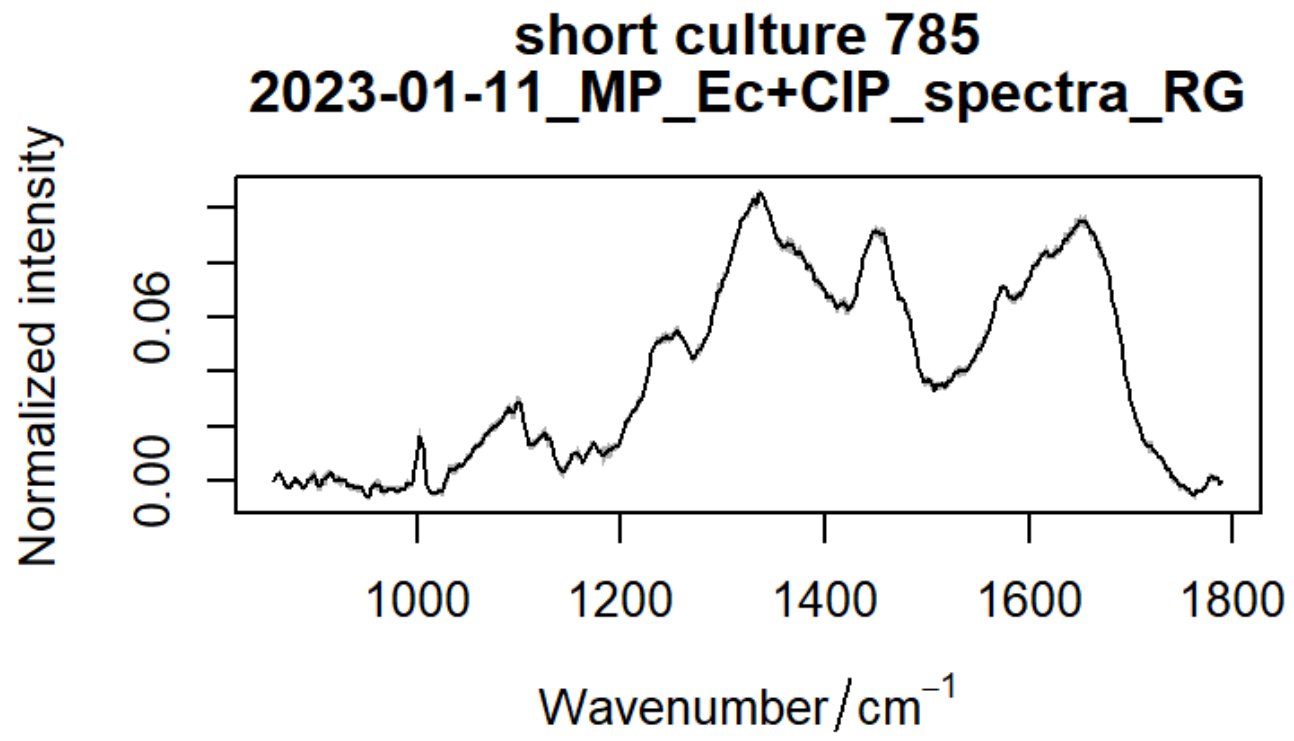

short culture 785
2023-01-11_MP_Ec+CIP_spectra_RG

E. coli_ATCC_25922 MP - CIP: 0.0000mg/l

```
## short culture 785 2023-01-23_MP_Ec+CIP_spectra_RG
```

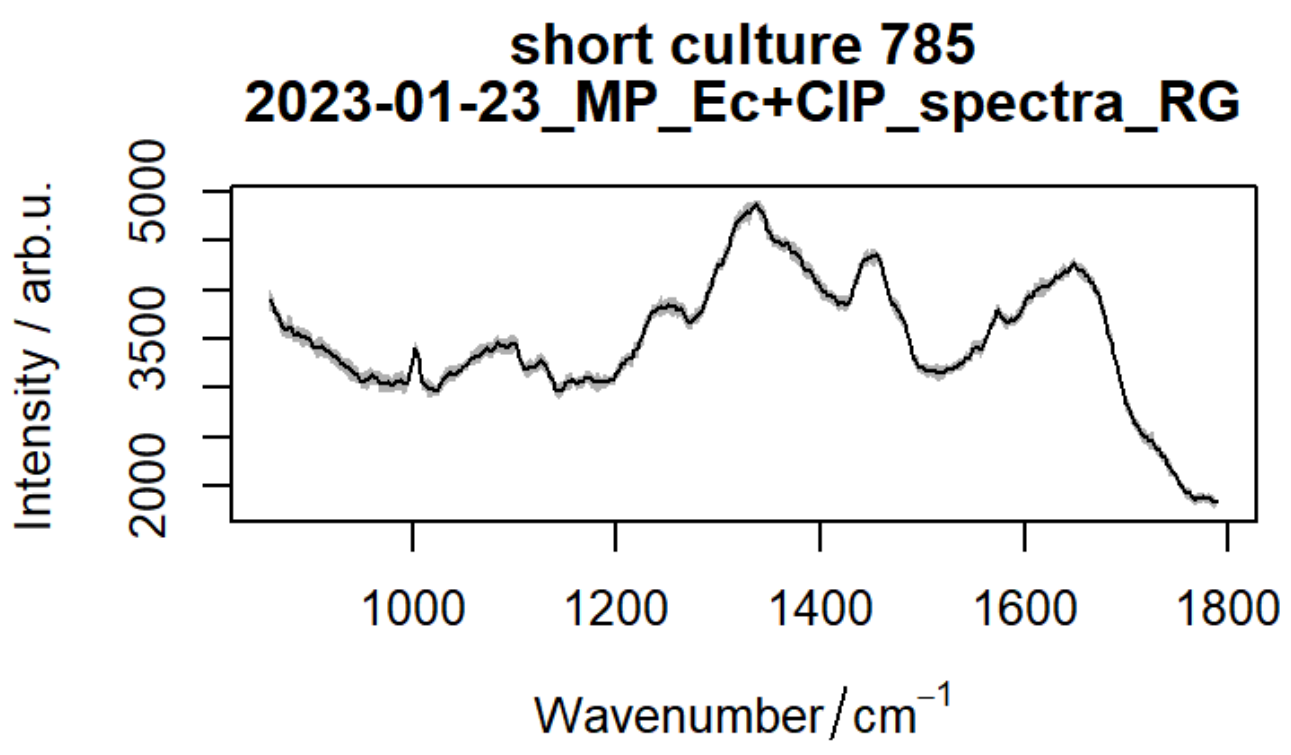

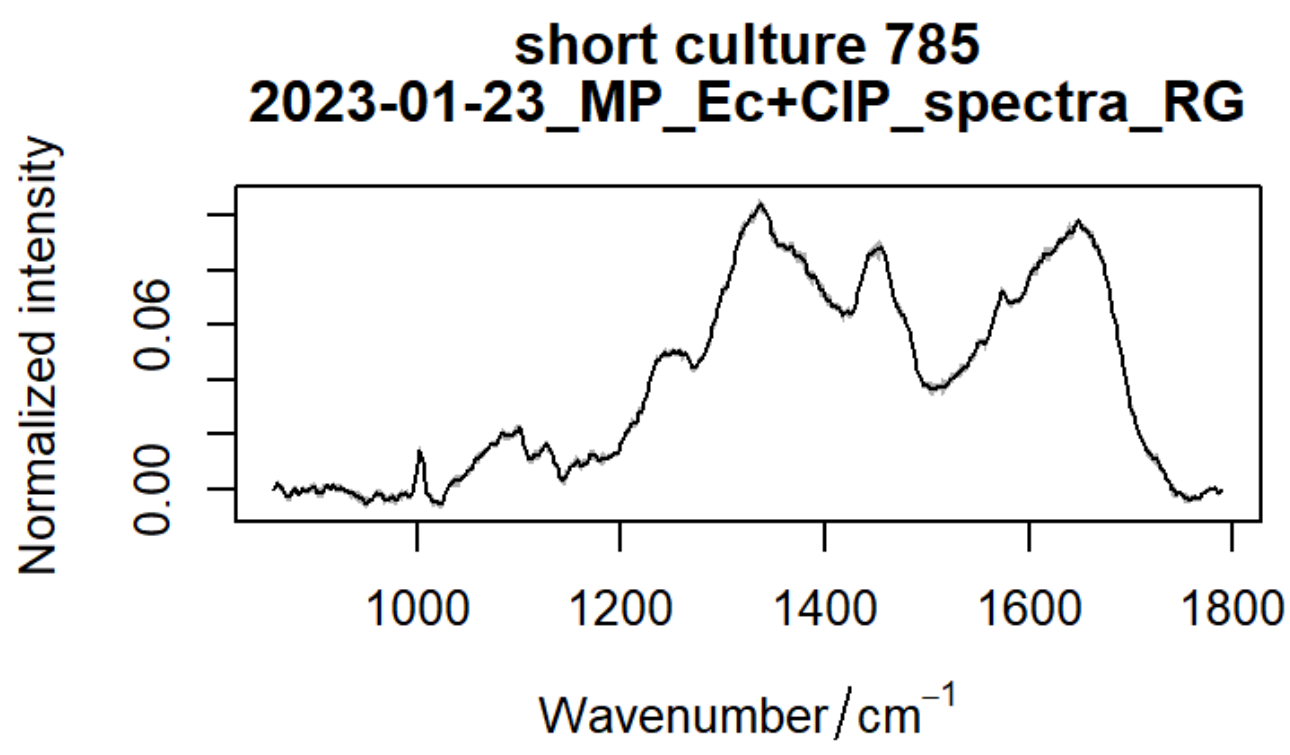

short culture 785
2023-01-23_MP_Ec+CIP_spectra_RG

E. coli_ATCC_25922 MP - CIP: 0.0000mg/l

```
## short culture 785 2023-05-03_MP_Sa+OXA_spectra_RG
```

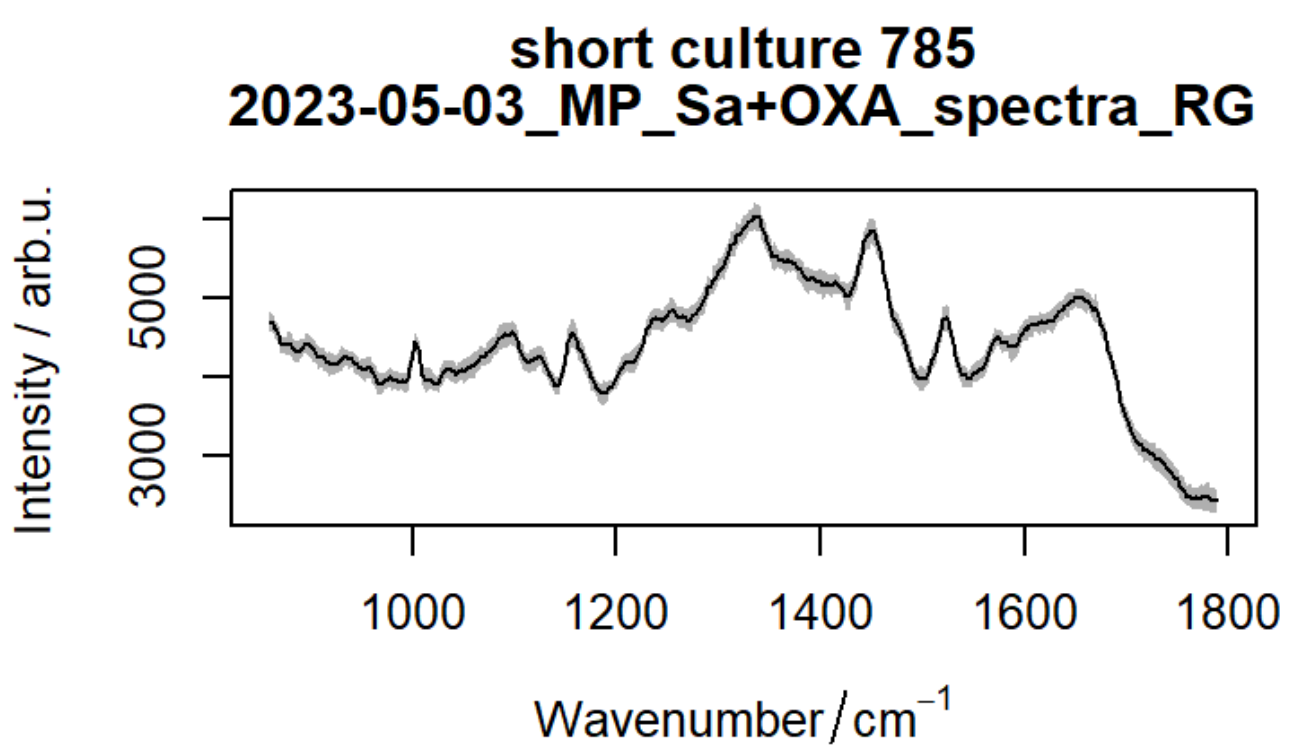

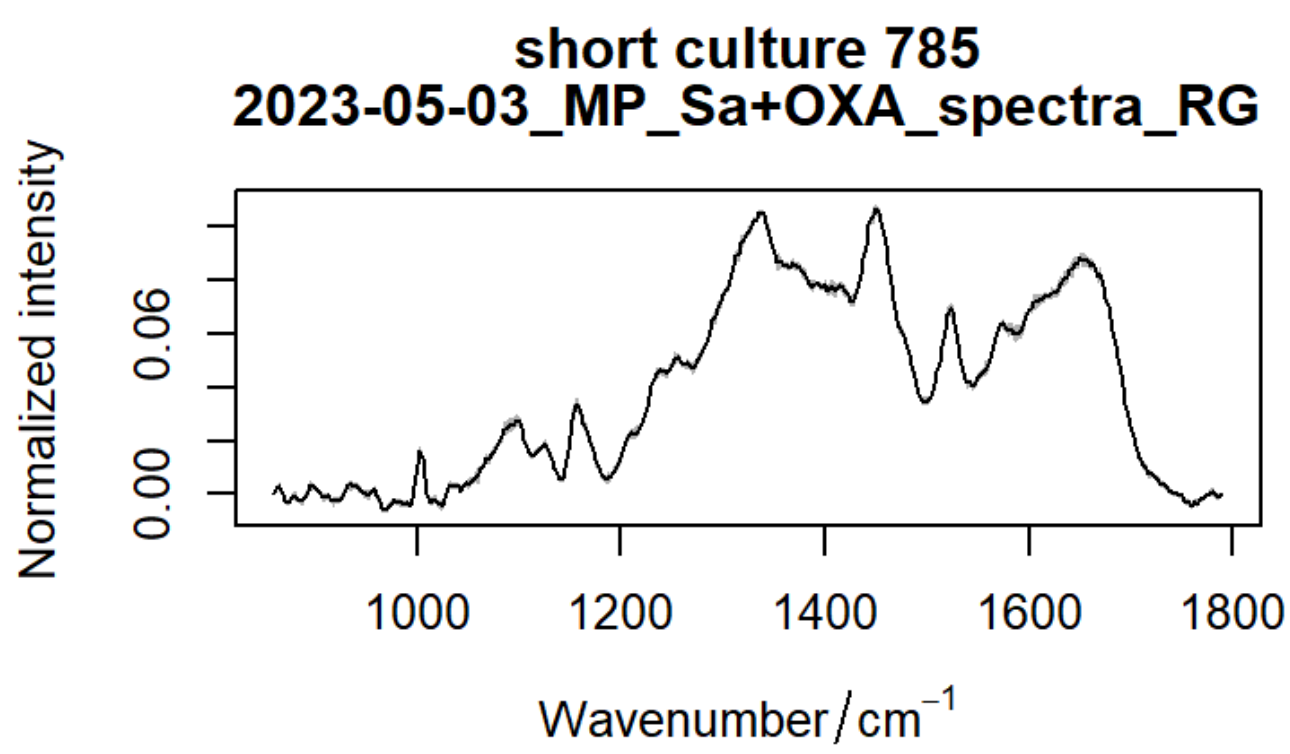

short culture 785
2023-05-03_MP_Sa+OXA_spectra_RG

S. aureus_ATCC_29213 MP - OXA: 0.0000mg/l

```
## short culture 785 2023-06-15_MP_Ec+CIP_spectra_RG
```

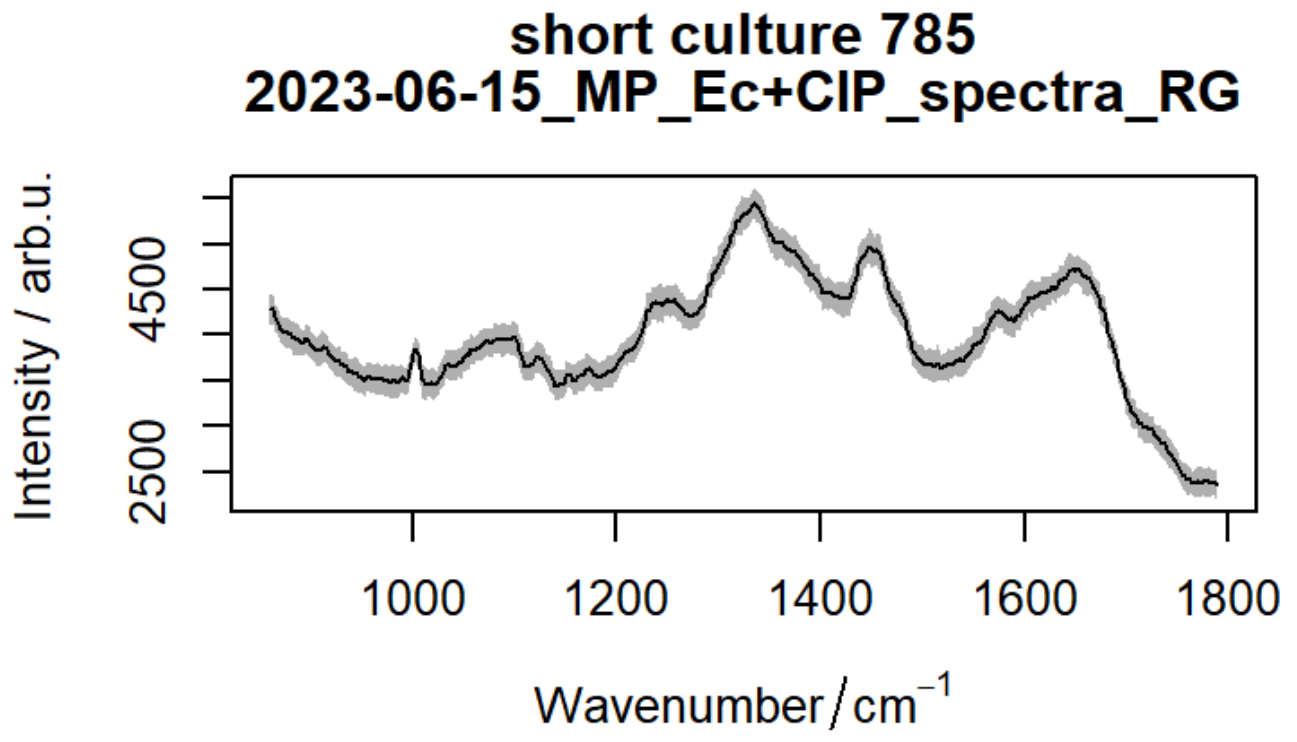

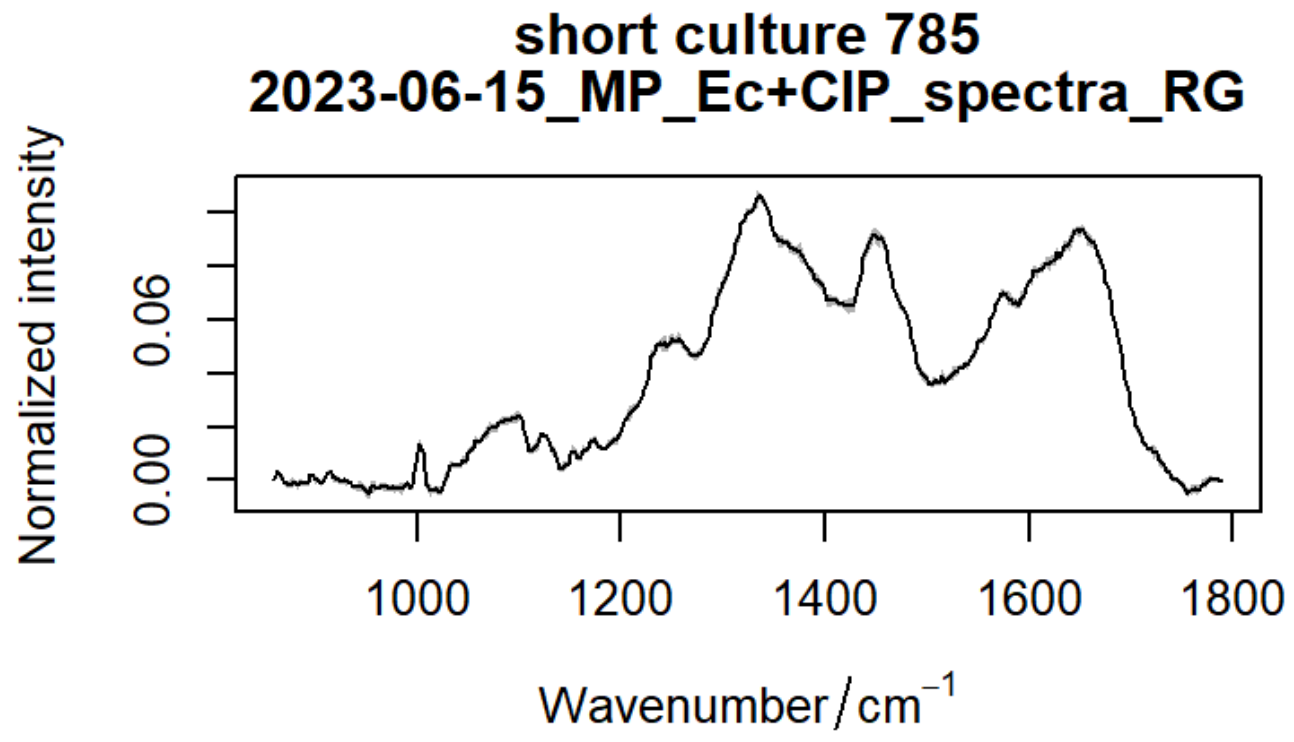

short culture 785
2023-06-15_MP_Ec+CIP_spectra_RG

E. coli_ATCC_25922 MP - CIP: 0.0000mg/l

```
## short culture 785 2023-06-20_MP_Sa+OXA_532 BK missing_spectra_RG
```

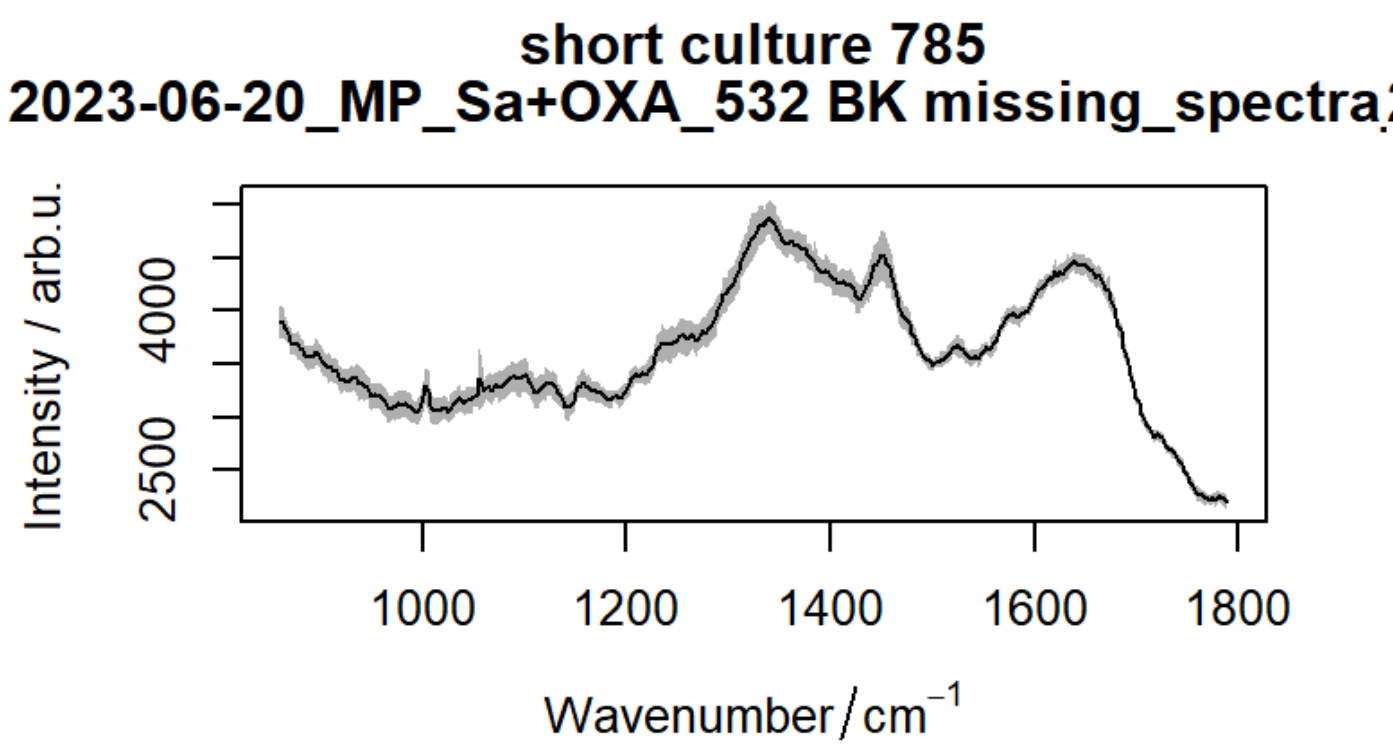

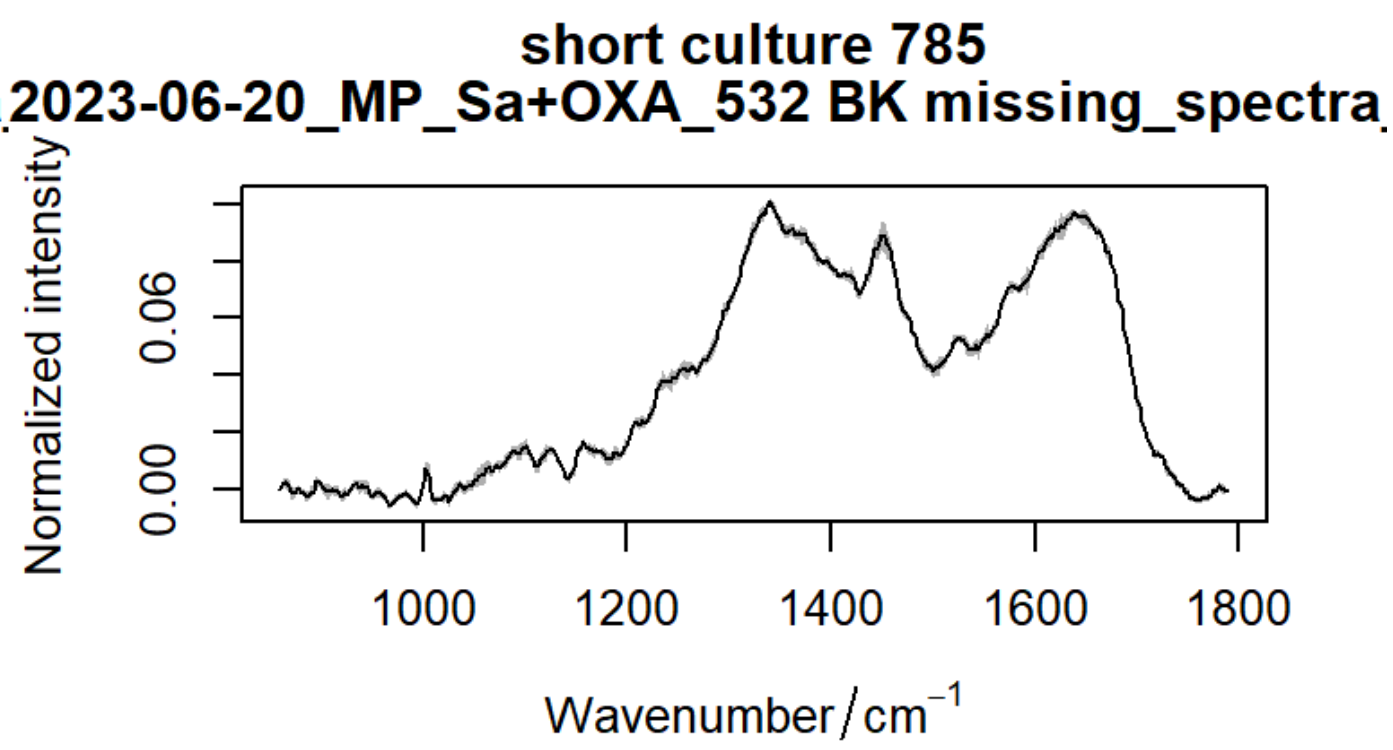

short culture 785
2023-06-20_MP_Sa+OXA_532 BK missing_spectra

S. aureus_ATCC_29213 MP - OXA: 0.0000mg/l

```
## short culture 785 2023-11-12_MP_Sa+OXA_spectra_RG
```

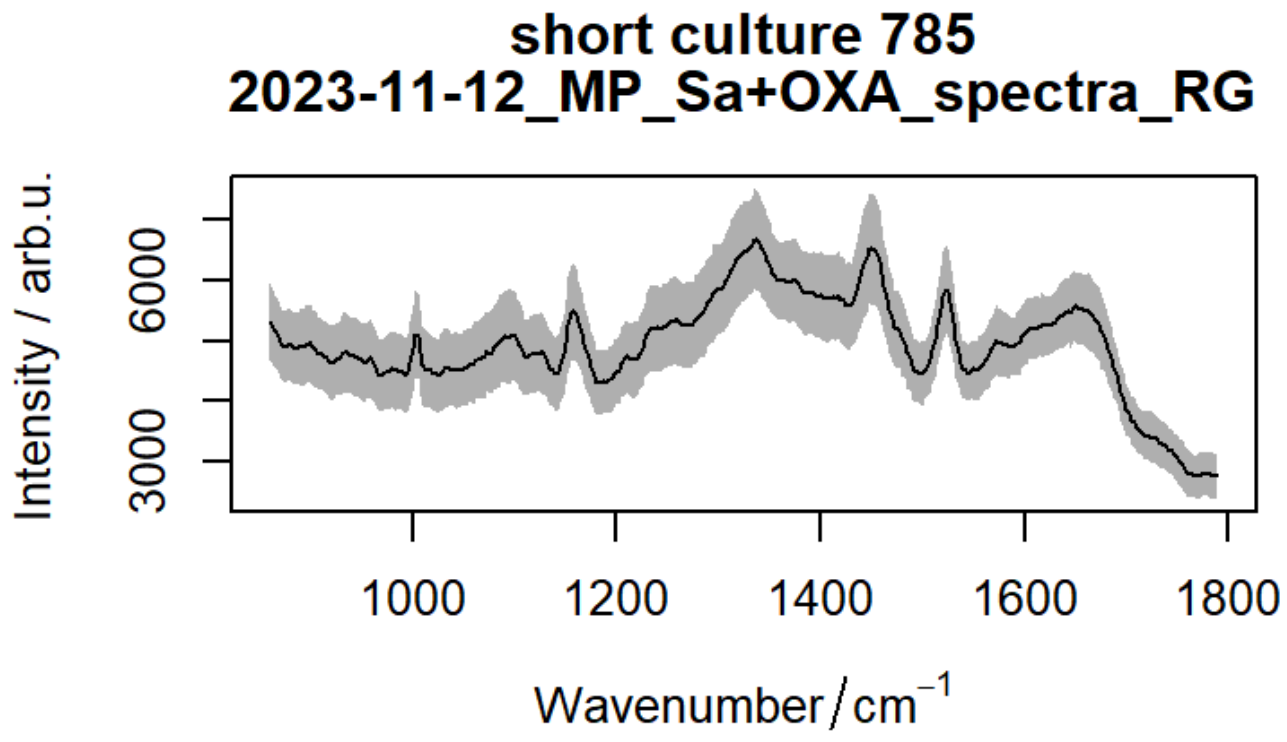

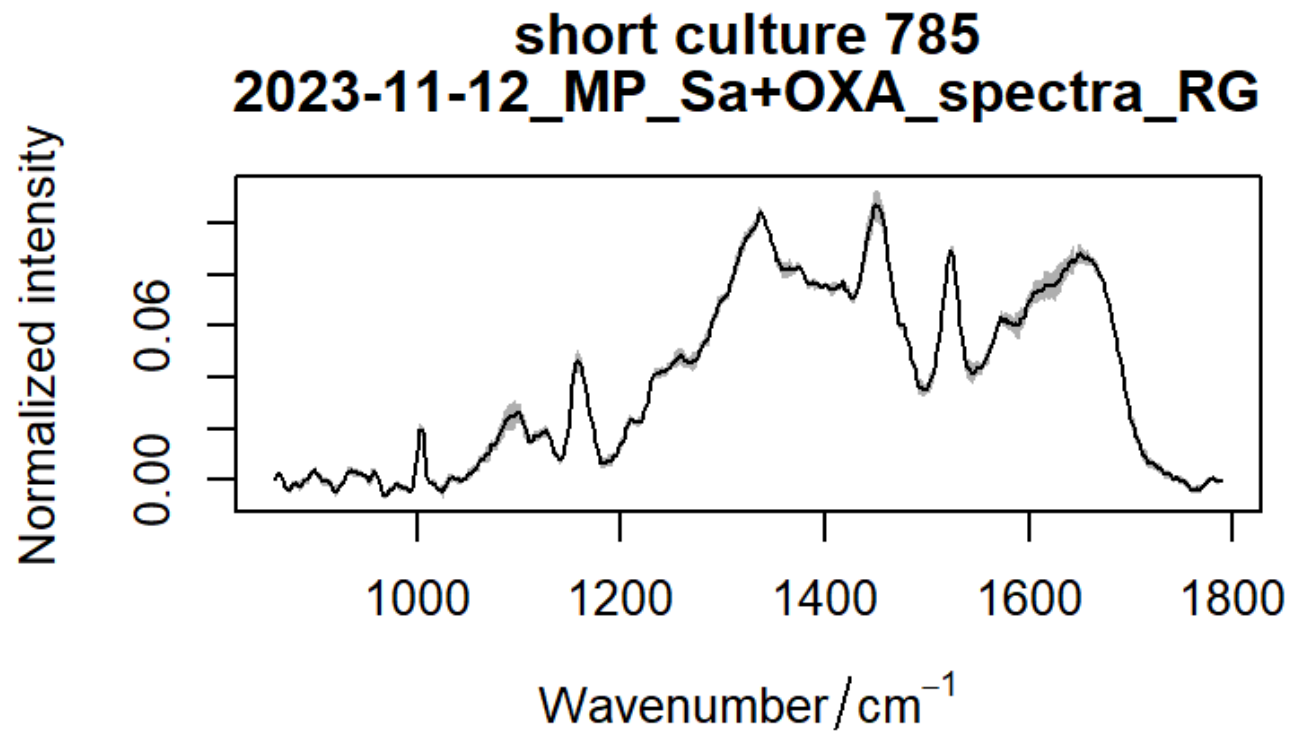

short culture 785
2023-11-12_MP_Sa+OXA_spectra_RG

S. aureus_ATCC_29213 MP - OXA: 0.0000mg/l

```
##            pred
## true         E. coli S. aureus       acc sensitivity specificity
##   E. coli        18         0 0.9722222   1.0000000   0.9444444
##   S. aureus       1        17 0.9722222   0.9444444   1.0000000
```

```
##
## Balanced accuracy: 97.2 %
```

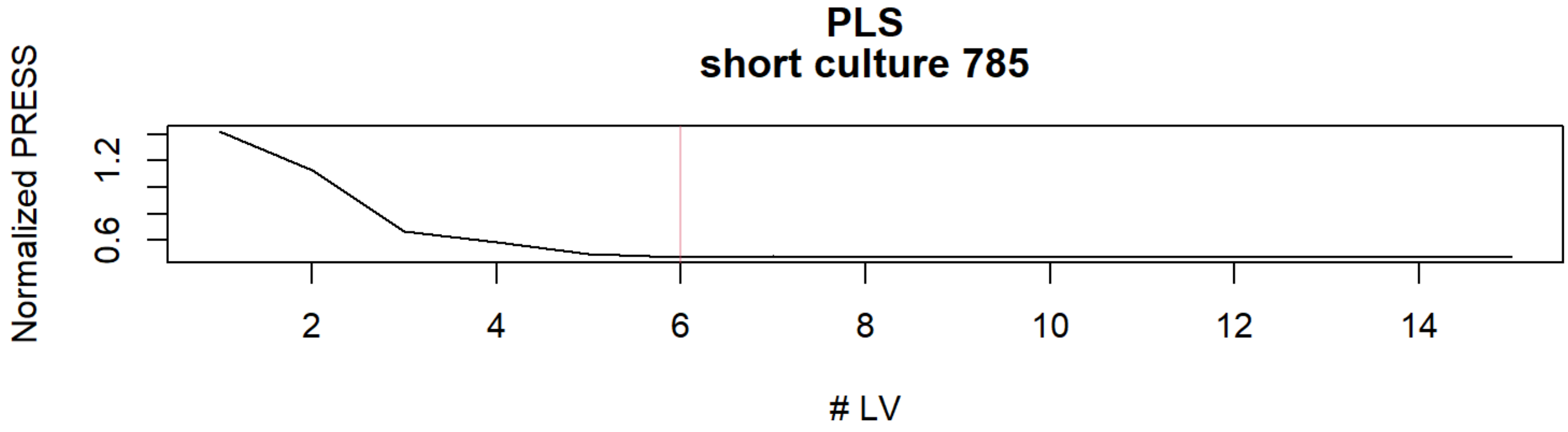

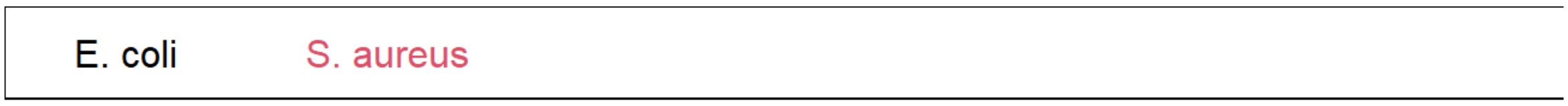

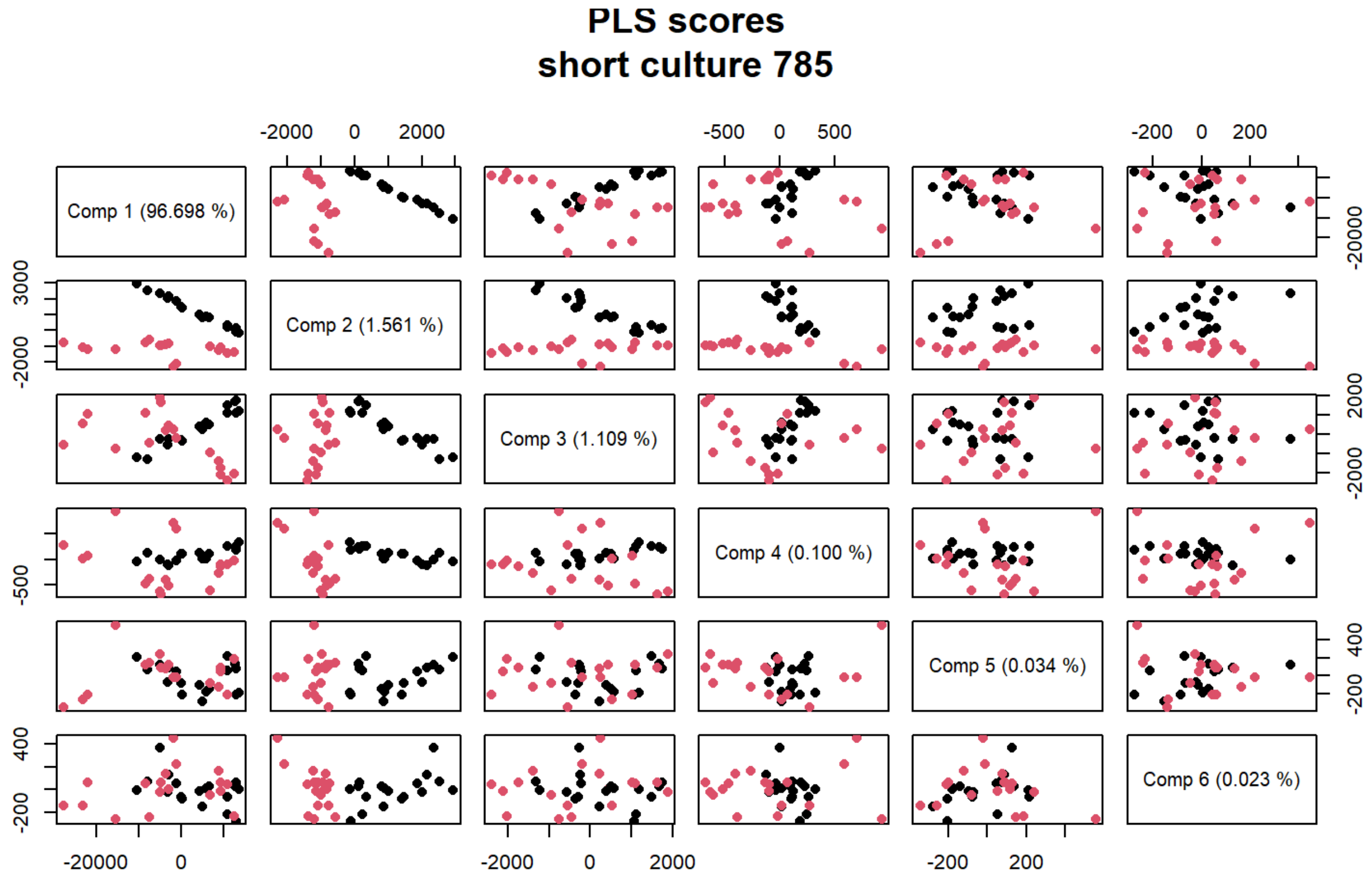
PLS scores
short culture 785
Comp 1 (96.698 %)
Comp 2 (1.561 %)
Comp 3 (1.109 %)
Comp 4 (0.100 %)
Comp 5 (0.034 %)
Comp 6 (0.023 %)
-20000
0
-2000
2000
-500
500
-200
200
3000
400

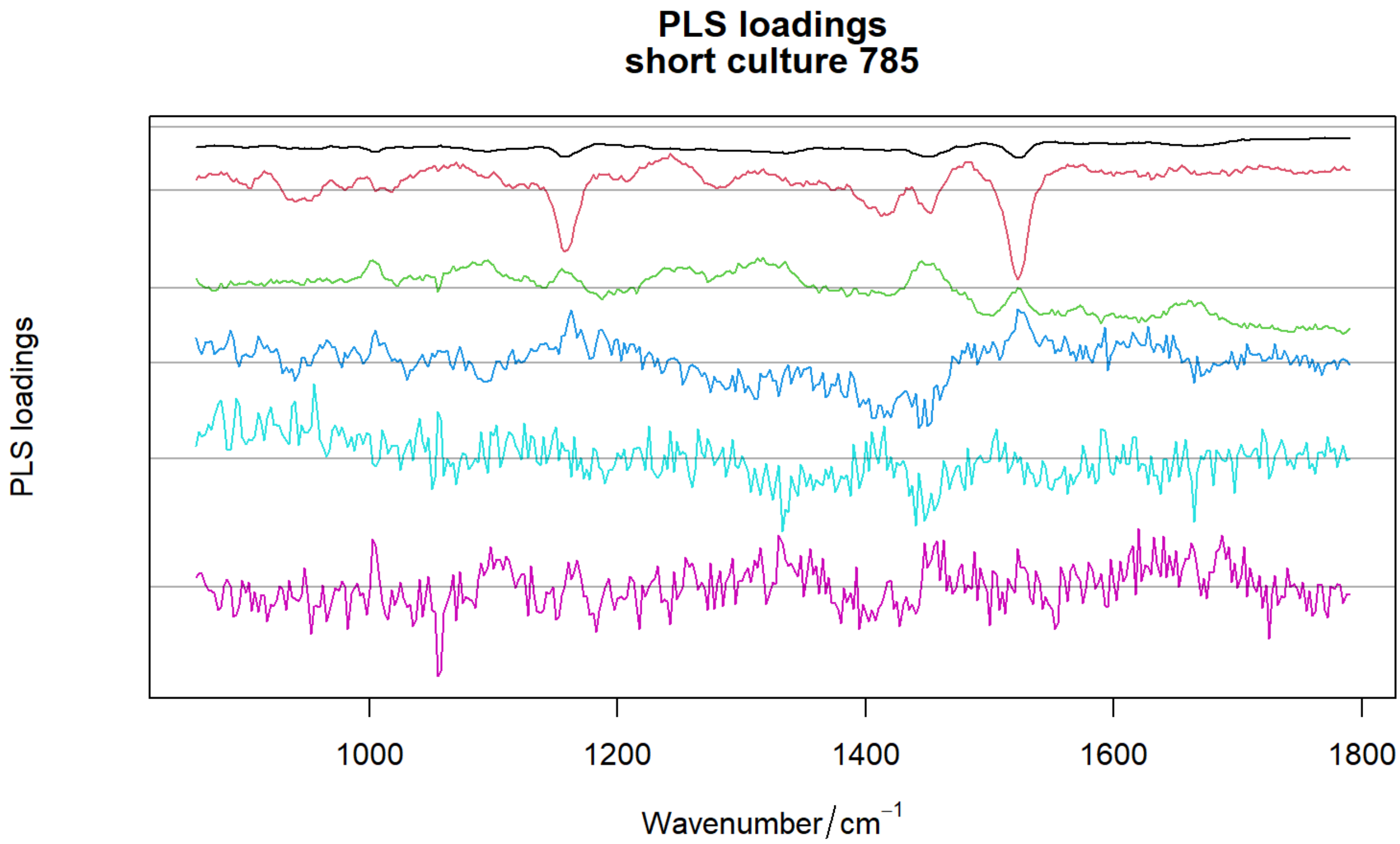
PLS loadings
short culture 785
PLS loadings
1000
1200
1400
1600
1800
Wavenumber/cm⁻¹

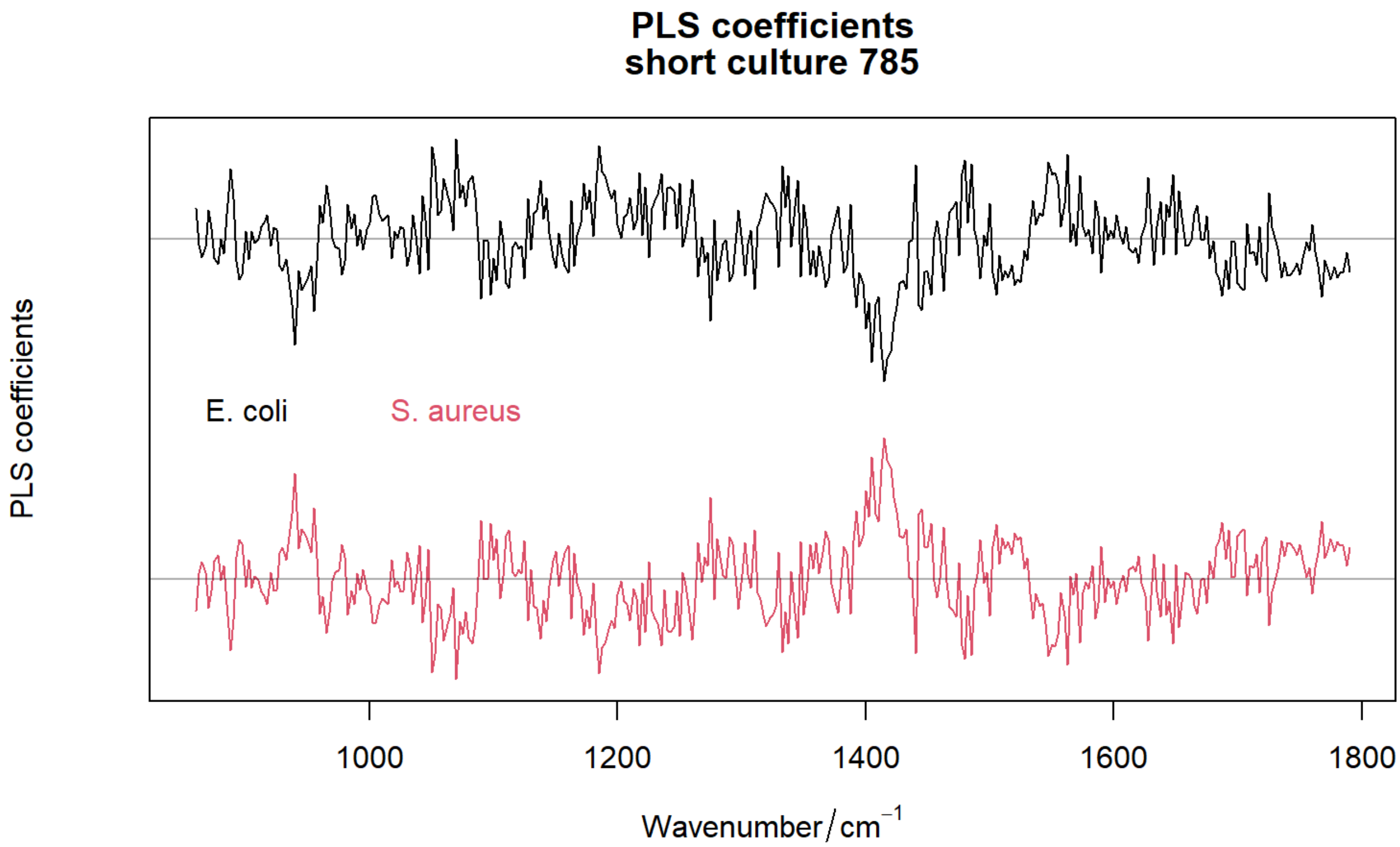
PLS coefficients
short culture 785
PLS coefficients
E. coli
S. aureus
1000
1200
1400
1600
1800
Wavenumber/cm^{-1}

Infecto_Xplore

```
## Culture: blood culture
## Laser: 785
```

```
## blood culture 785 2023-01-11_MP_Ec+CIP_spectra_RG
```

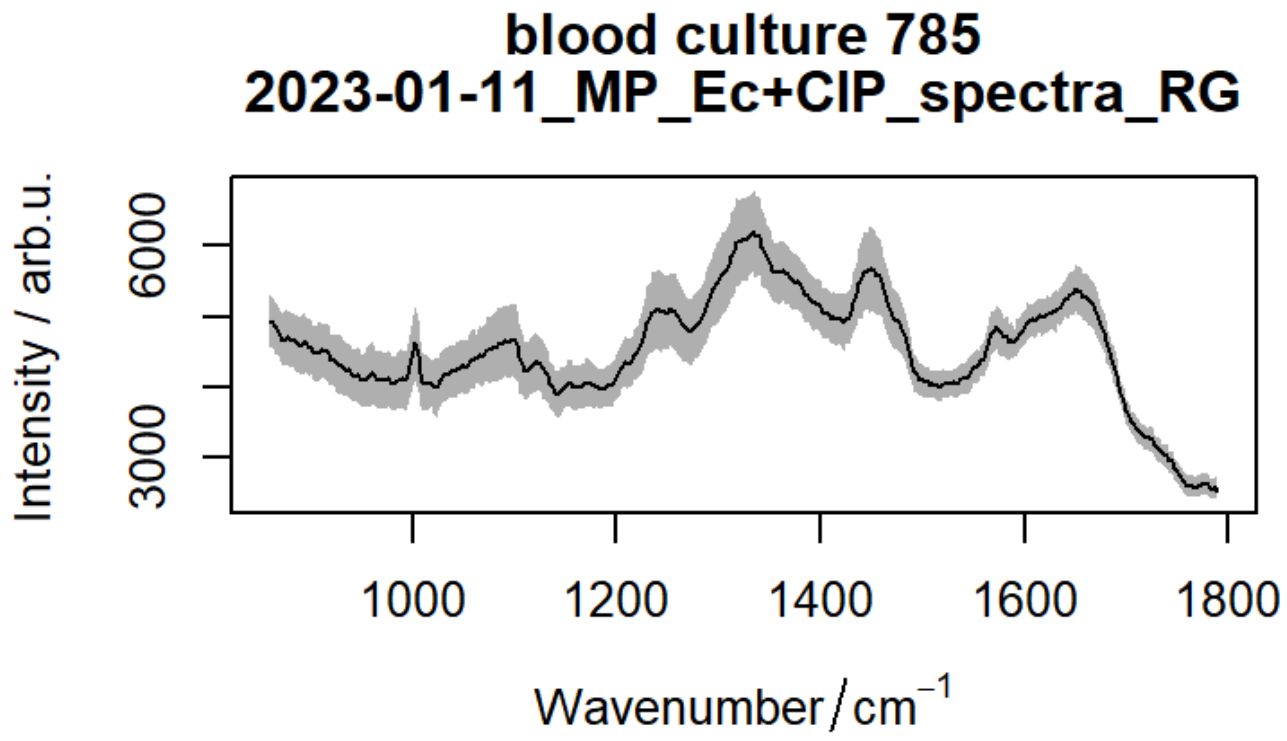

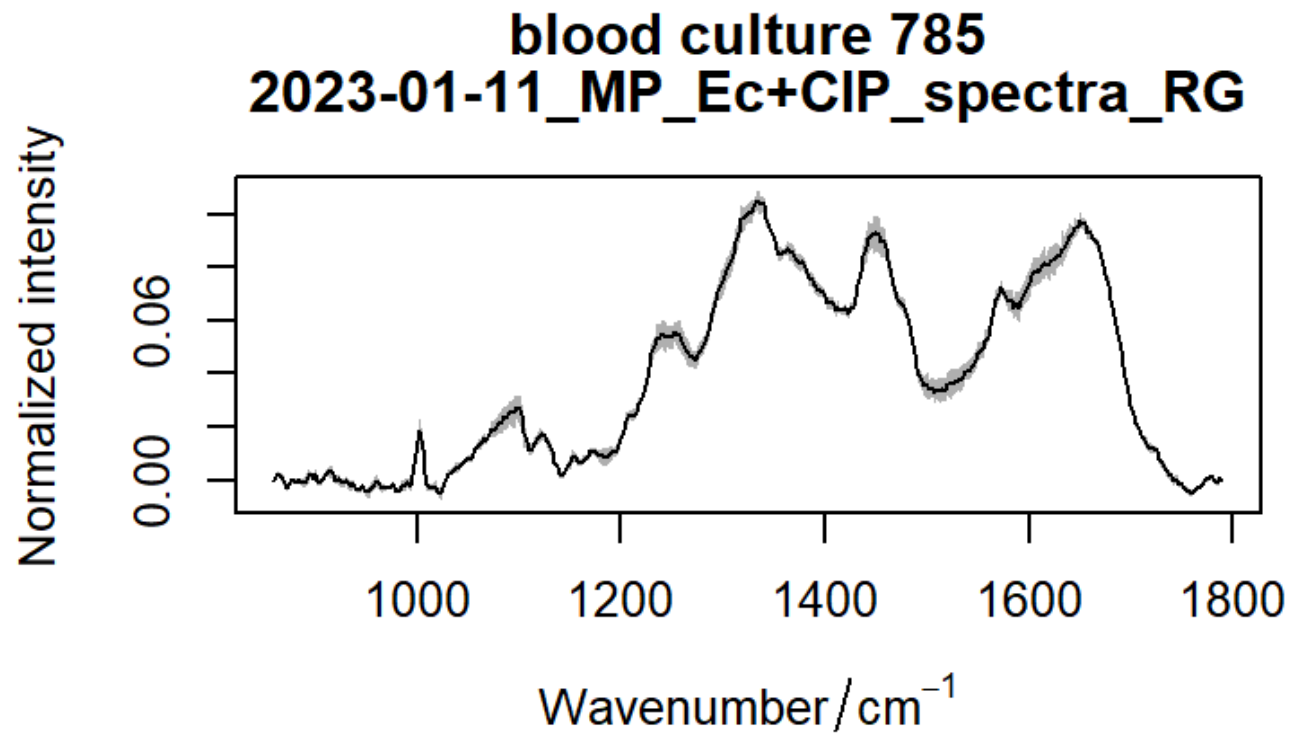

blood culture 785
2023-01-11_MP_Ec+CIP_spectra_RG

E. coli_ATCC_25922 MP - CIP: 0.0000mg/l

```
## blood culture 785 2023-01-23_MP_Ec+CIP_spectra_RG
```

**blood culture 785
2023-01-23_MP_Ec+CIP_spectra_RG**

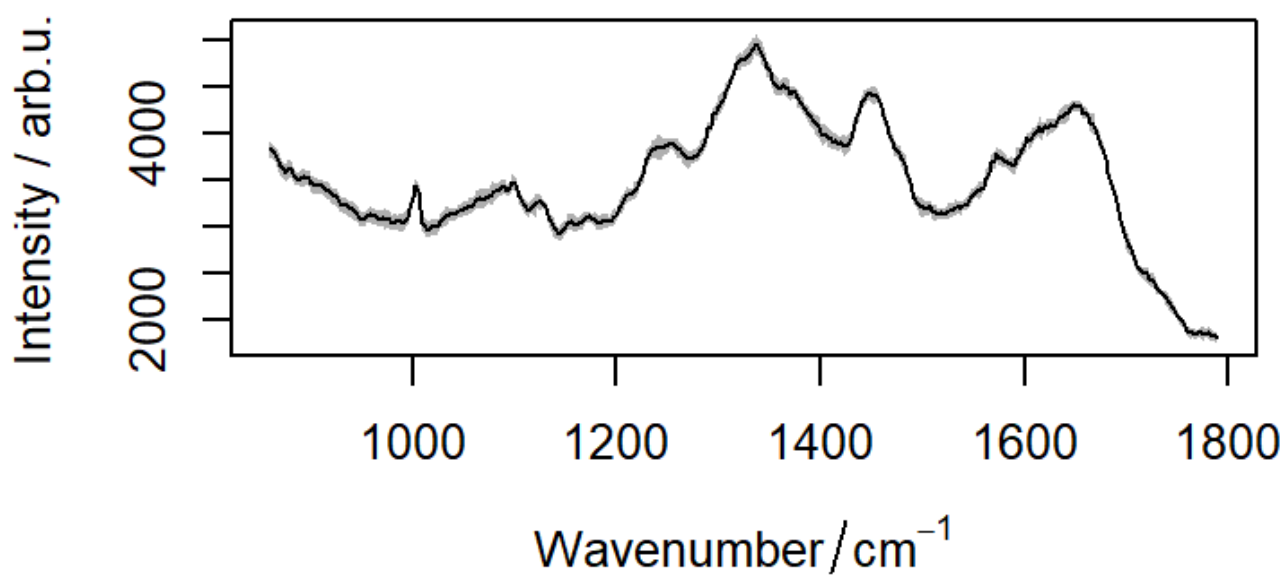

**blood culture 785
2023-01-23_MP_Ec+CIP_spectra_RG**

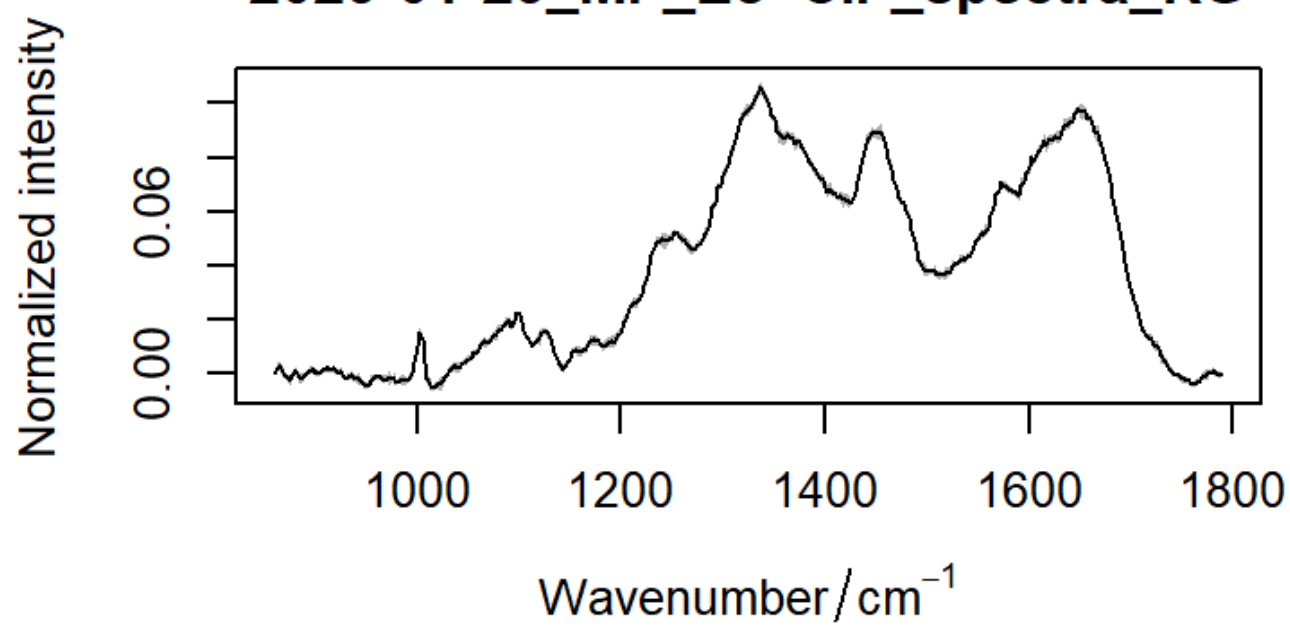

**blood culture 785
2023-01-23_MP_Ec+CIP_spectra_RG**

E. coli_ATCC_25922 MP - CIP: 0.0000mg/l

```
## blood culture 785 2023-05-03_MP_Sa+OXA_spectra_RG
```

**blood culture 785
2023-05-03_MP_Sa+OXA_spectra_RG**

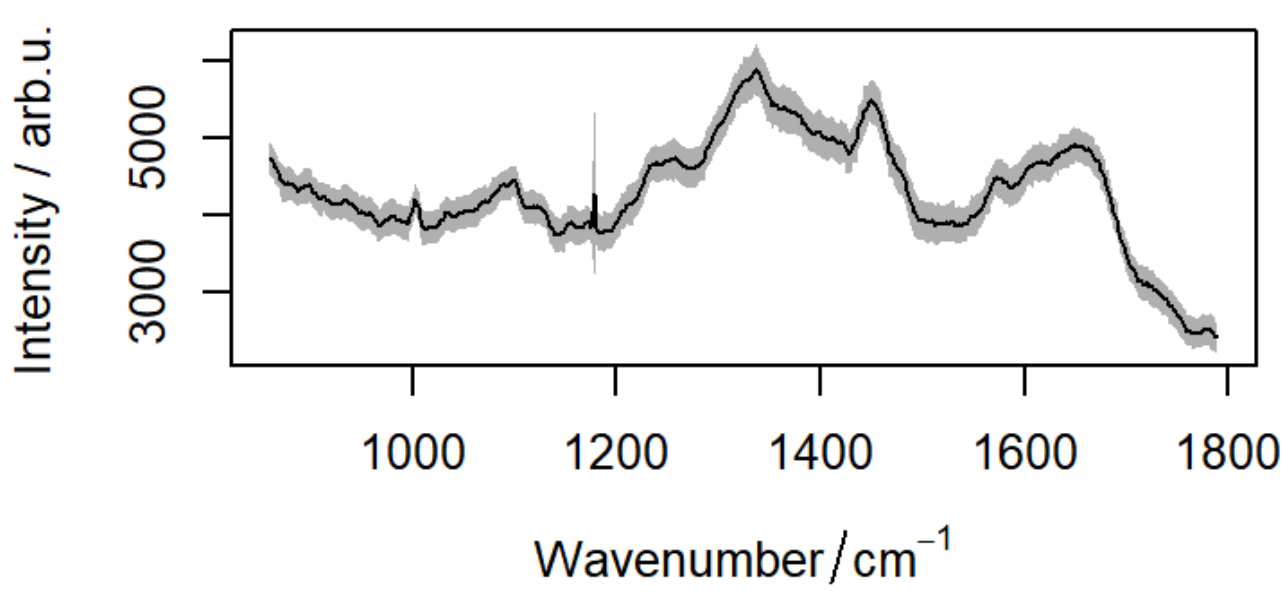

**blood culture 785
2023-05-03_MP_Sa+OXA_spectra_RG**

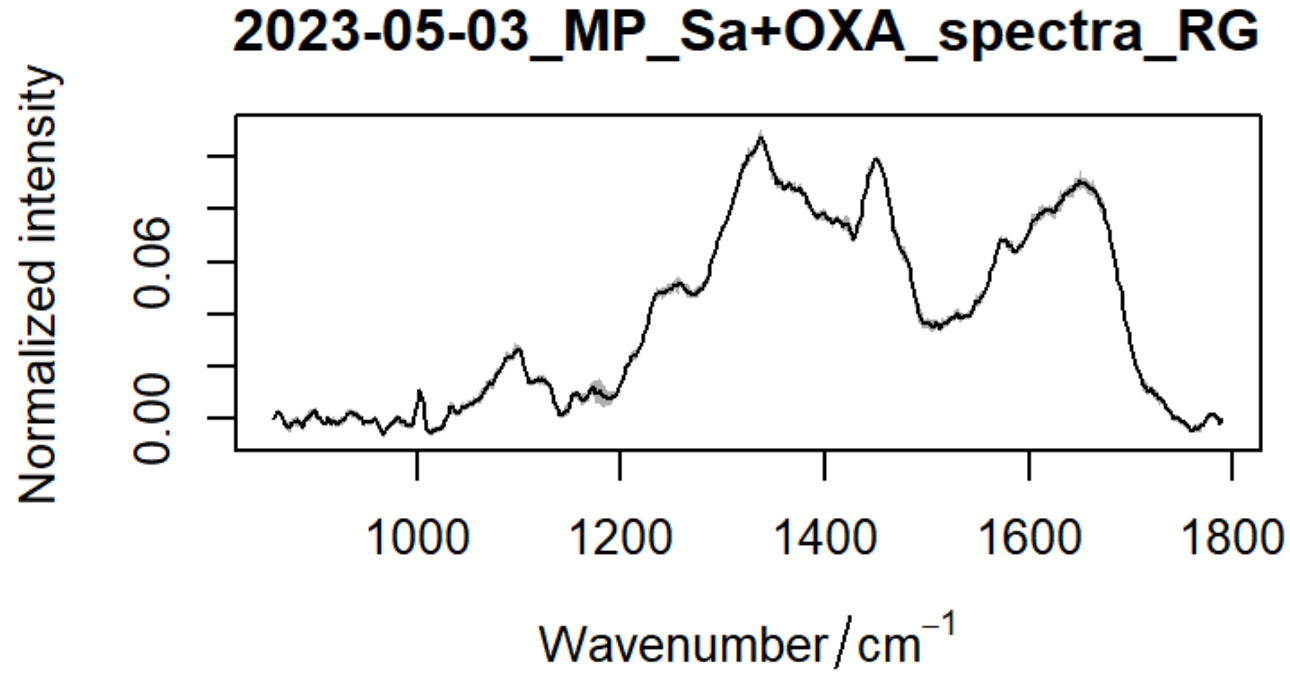

**blood culture 785
2023-05-03_MP_Sa+OXA_spectra_RG**

S. aureus_ATCC_29213 MP - OXA: 0.0000mg/l

```
## blood culture 785 2023-06-15_MP_Ec+CIP_spectra_RG
```

blood culture 785
2023-06-15_MP_Ec+CIP_spectra_RG

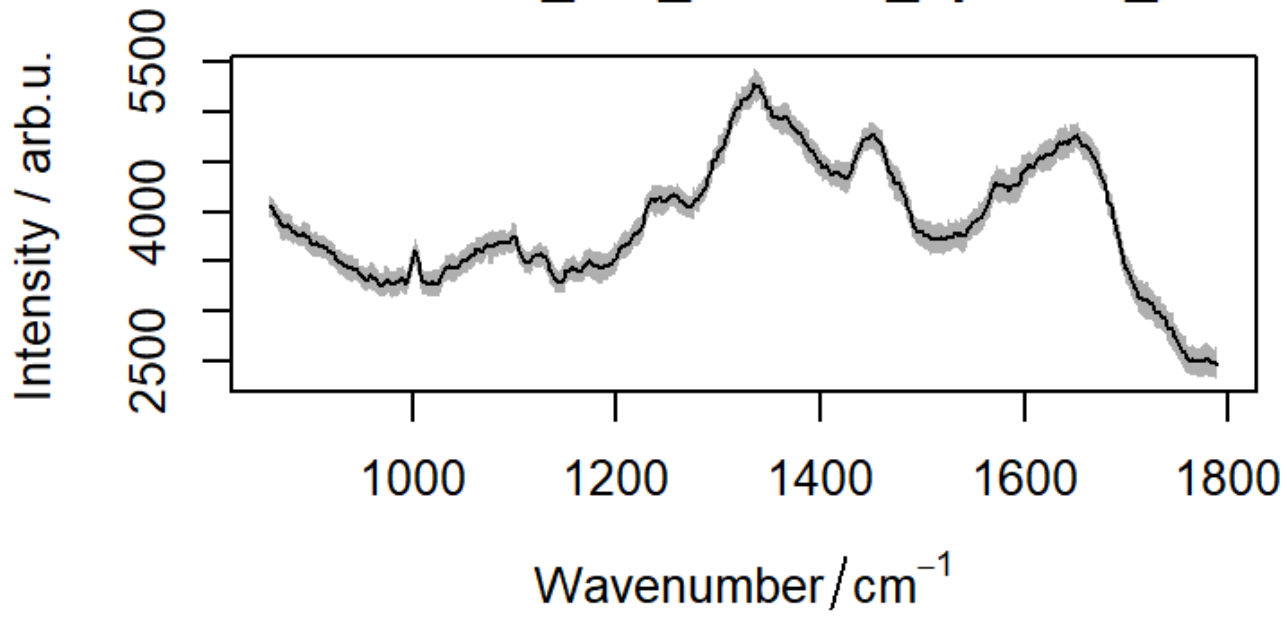

blood culture 785
2023-06-15_MP_Ec+CIP_spectra_RG

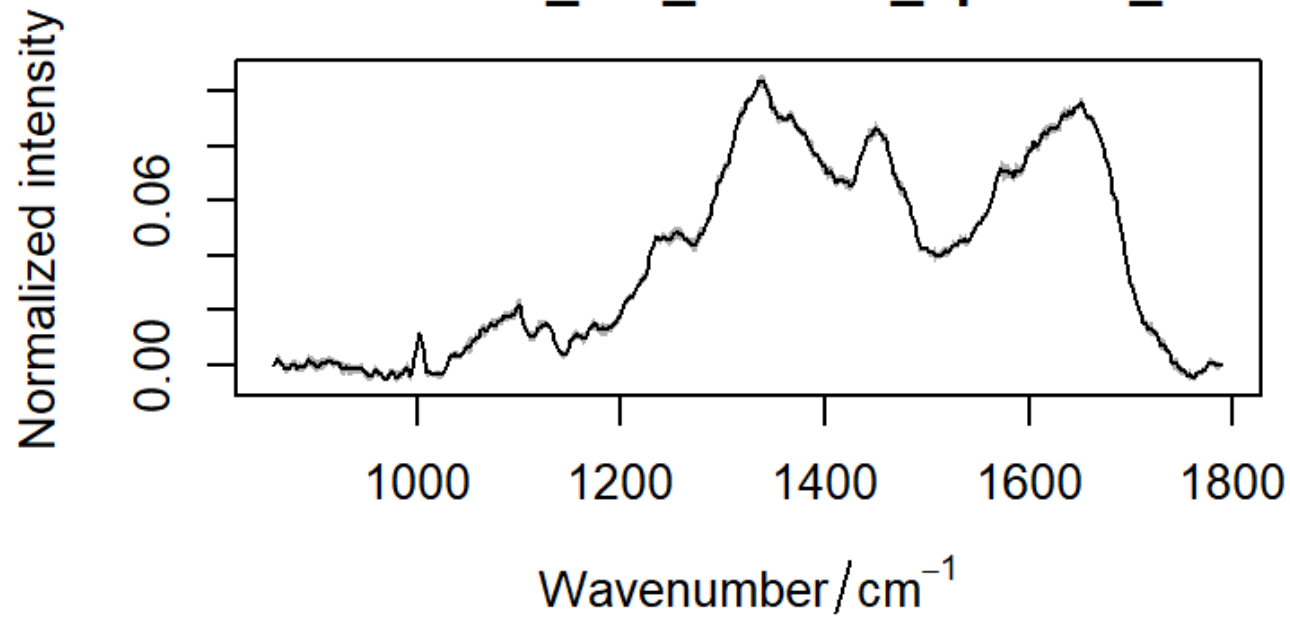

blood culture 785
2023-06-15_MP_Ec+CIP_spectra_RG

E. coli_ATCC_25922 MP - CIP: 0.0000mg/l

```
## blood culture 785 2023-06-20_MP_Sa+OXA_532 BK missing_spectra_RG
```

blood culture 785
2023-06-20_MP_Sa+OXA_532 BK missing_spectra

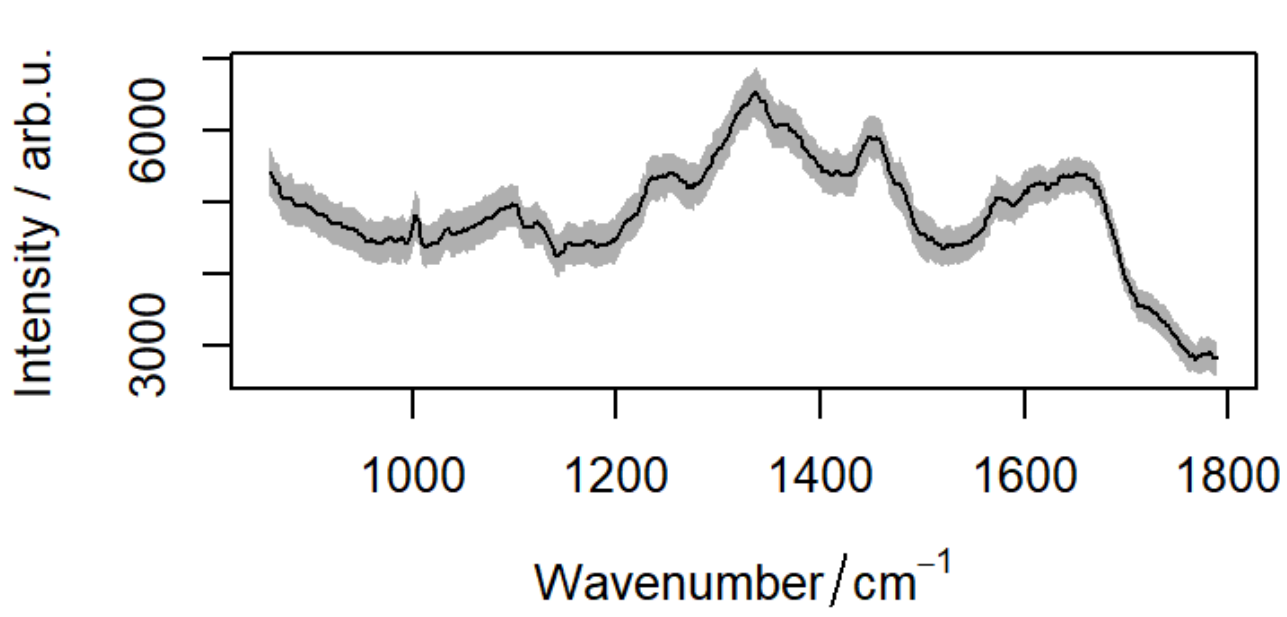

blood culture 785
2023-06-20_MP_Sa+OXA_532 BK missing_spectra

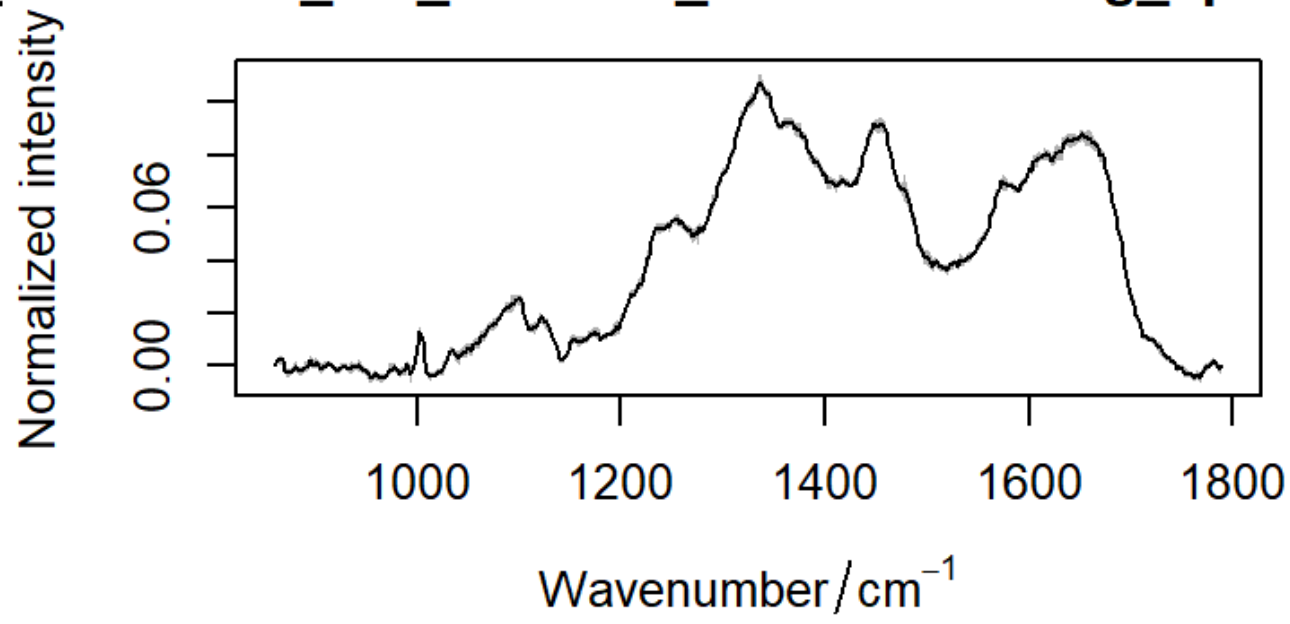

blood culture 785
2023-06-20_MP_Sa+OXA_532 BK missing_spectra

S. aureus_ATCC_29213 MP - OXA: 0.0000mg/l

```
## blood culture 785 2023-11-12_MP_Sa+OXA_spectra_RG
```

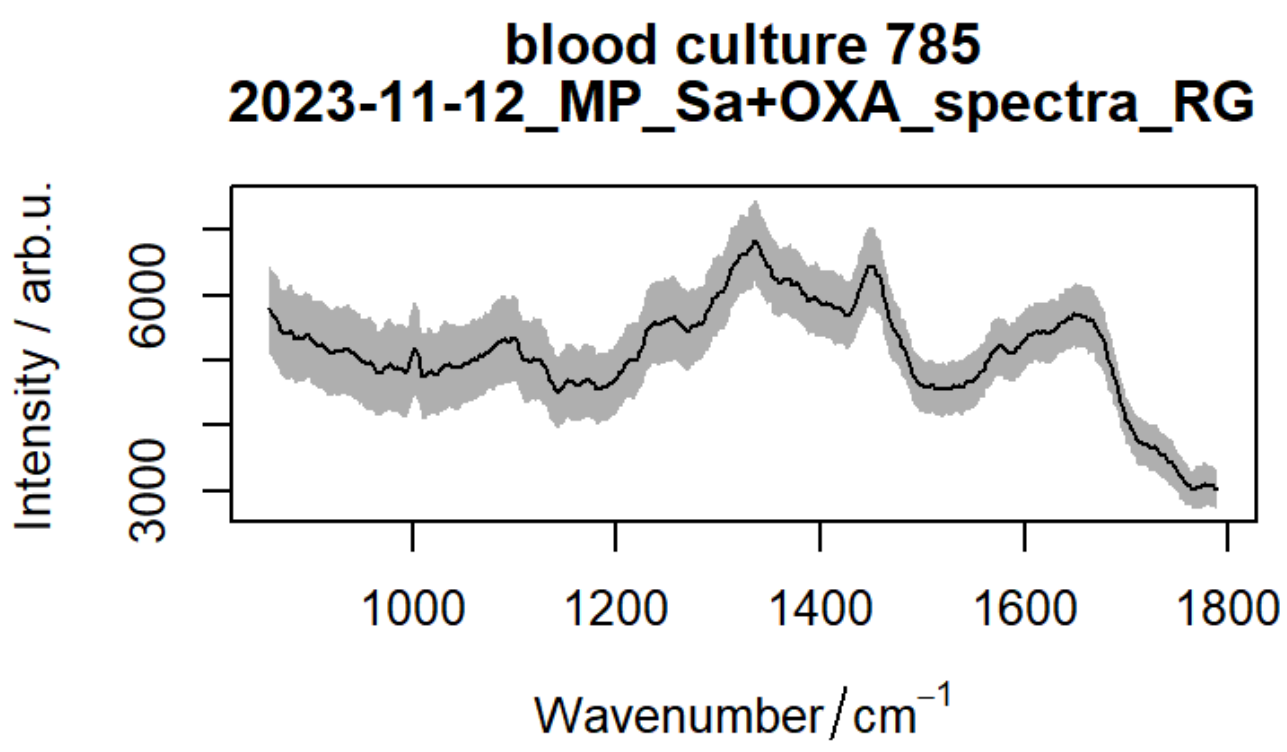

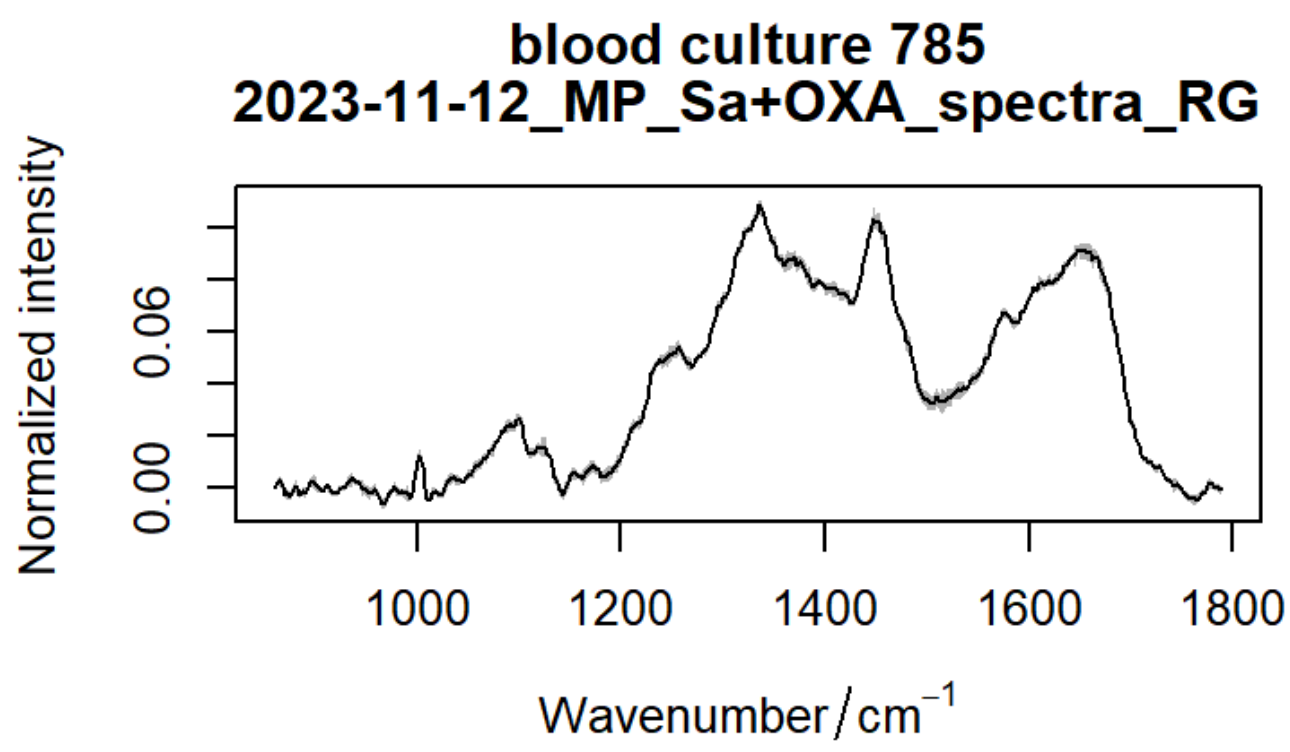

blood culture 785
2023-11-12_MP_Sa+OXA_spectra_RG

S. aureus_ATCC_29213 MP - OXA: 0.0000mg/l

```
##              pred
## true          E. coli S. aureus       acc sensitivity specificity
##    E. coli         15         3 0.8888889   0.8333333   0.9444444
##    S. aureus        1        17 0.8888889   0.9444444   0.8333333
```

```
##
## Balanced accuracy: 88.9 %
```

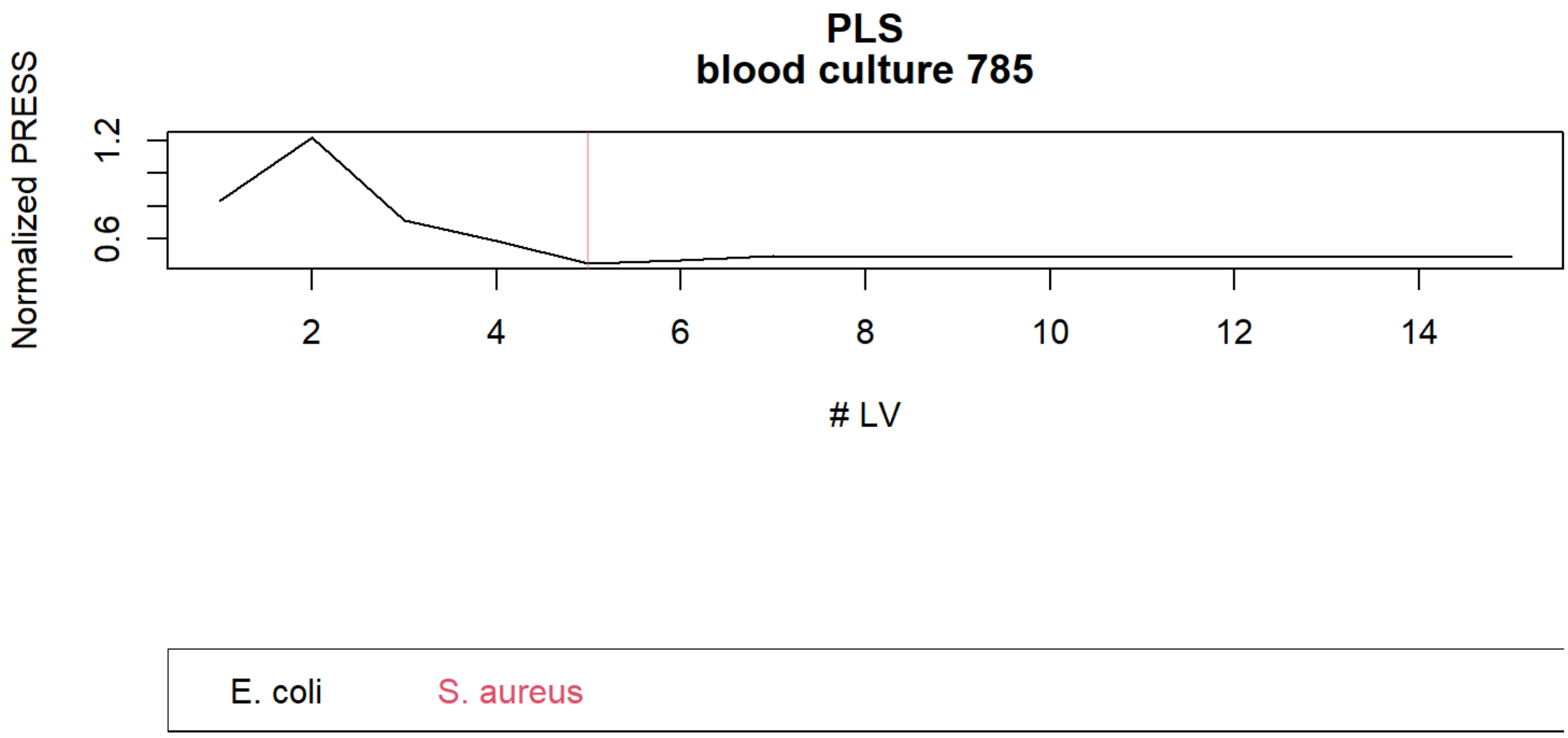

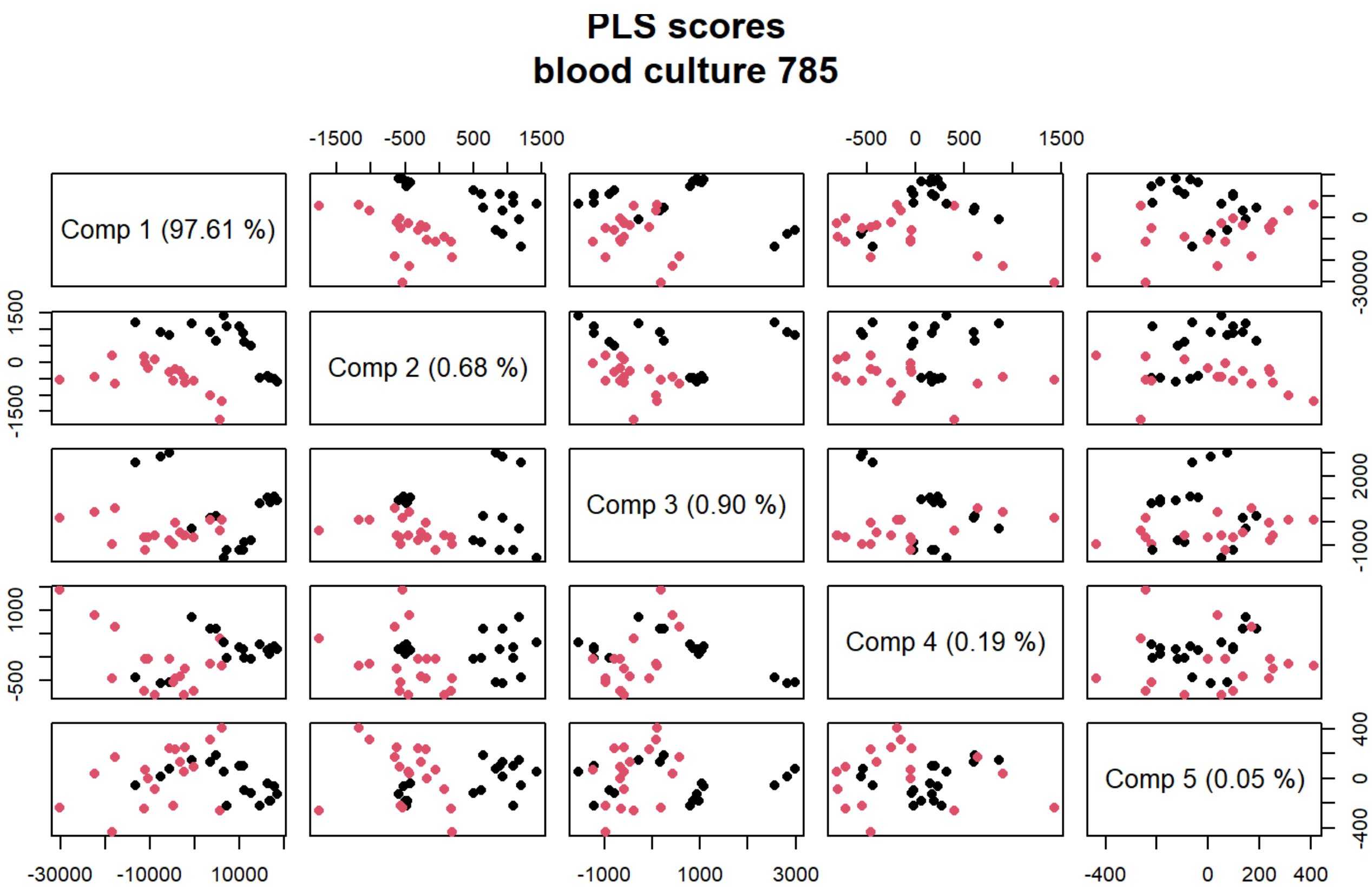
PLS scores
blood culture 785
Comp 1 (97.61 %)
Comp 2 (0.68 %)
Comp 3 (0.90 %)
Comp 4 (0.19 %)
Comp 5 (0.05 %)
-30000
-10000
10000
-1500
-500
500
1500
-1000
1000
3000
-500
0
500
1500
-400
0
200
400
0
-30000
1500
0
-1500
2000
-1000
1000
-500
400
0
-400

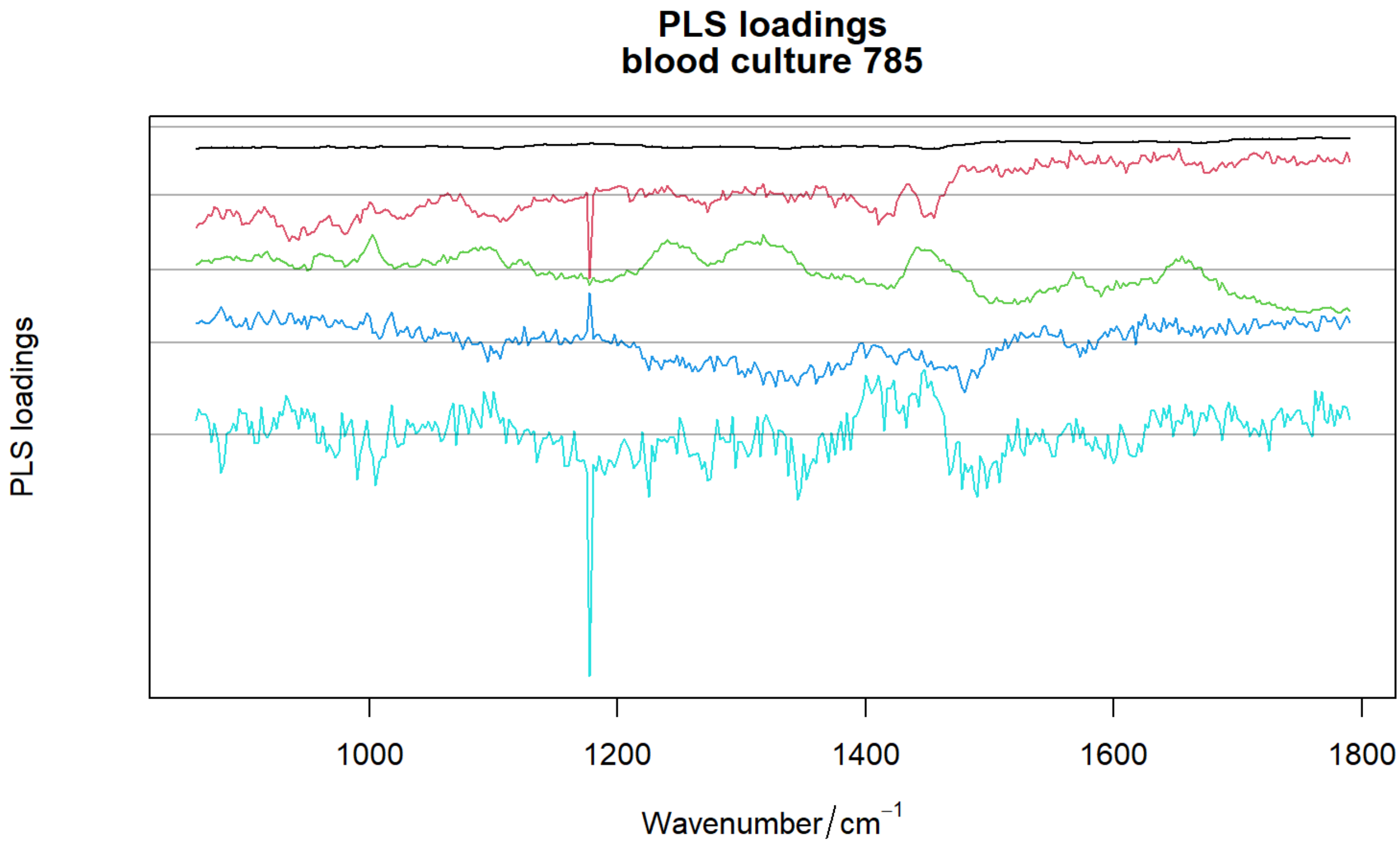
PLS loadings
blood culture 785
PLS loadings
1000
1200
1400
1600
1800
Wavenumber / cm⁻¹

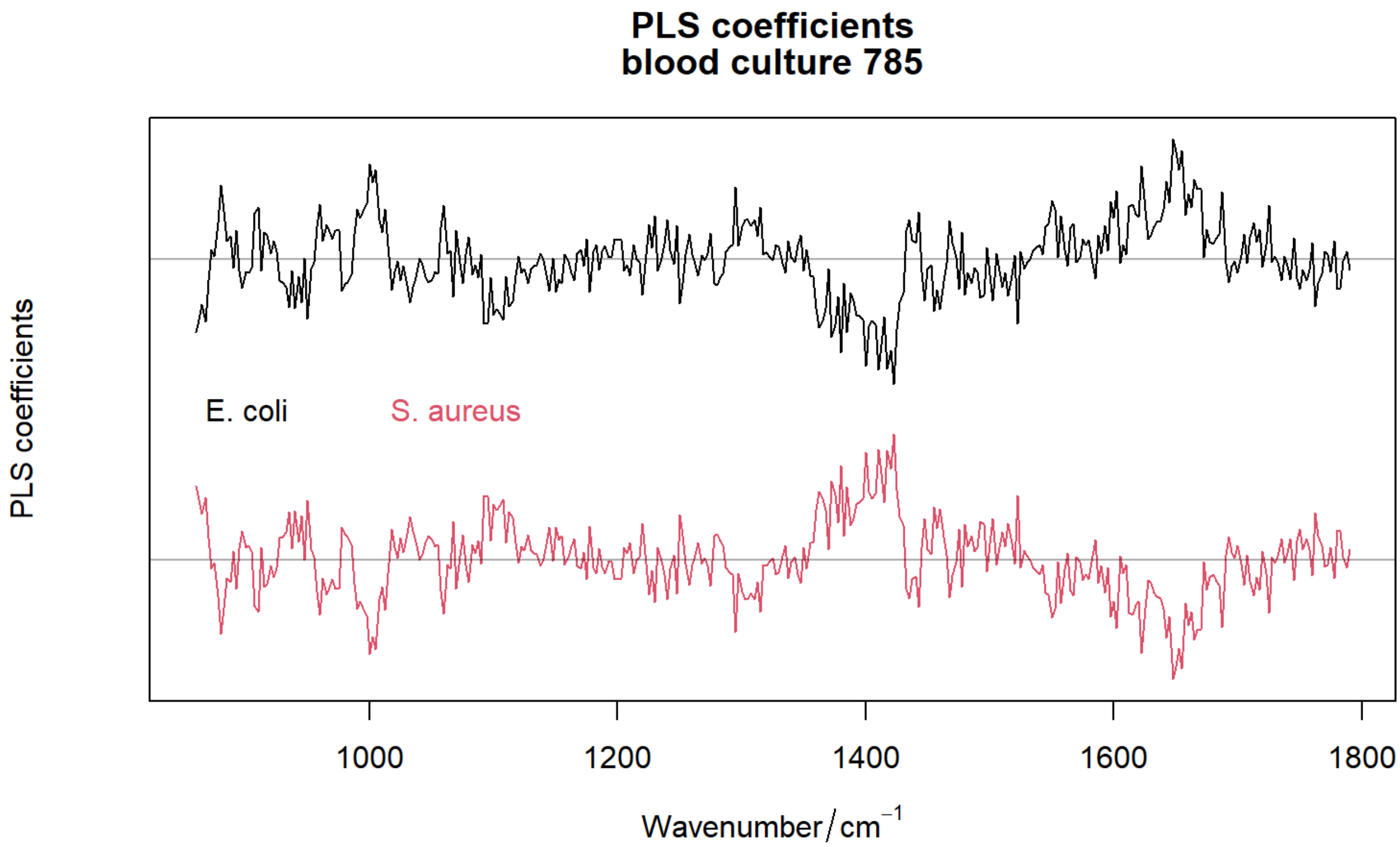
PLS coefficients
blood culture 785
PLS coefficients
E. coli
S. aureus
1000
1200
1400
1600
1800
Wavenumber/cm−1

Infecto_Xplore

```
## Culture: real blood culture
## Laser: 785
```

```
## real blood culture 785 e.c. 1
```

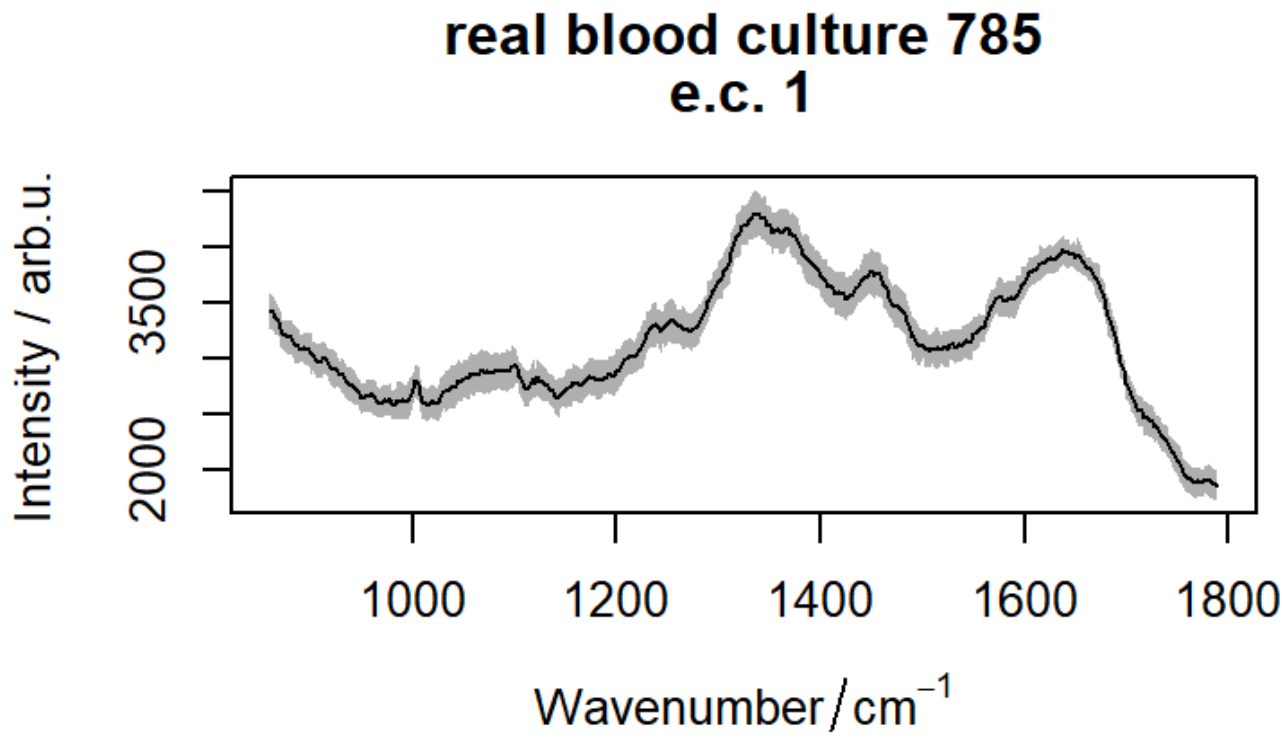

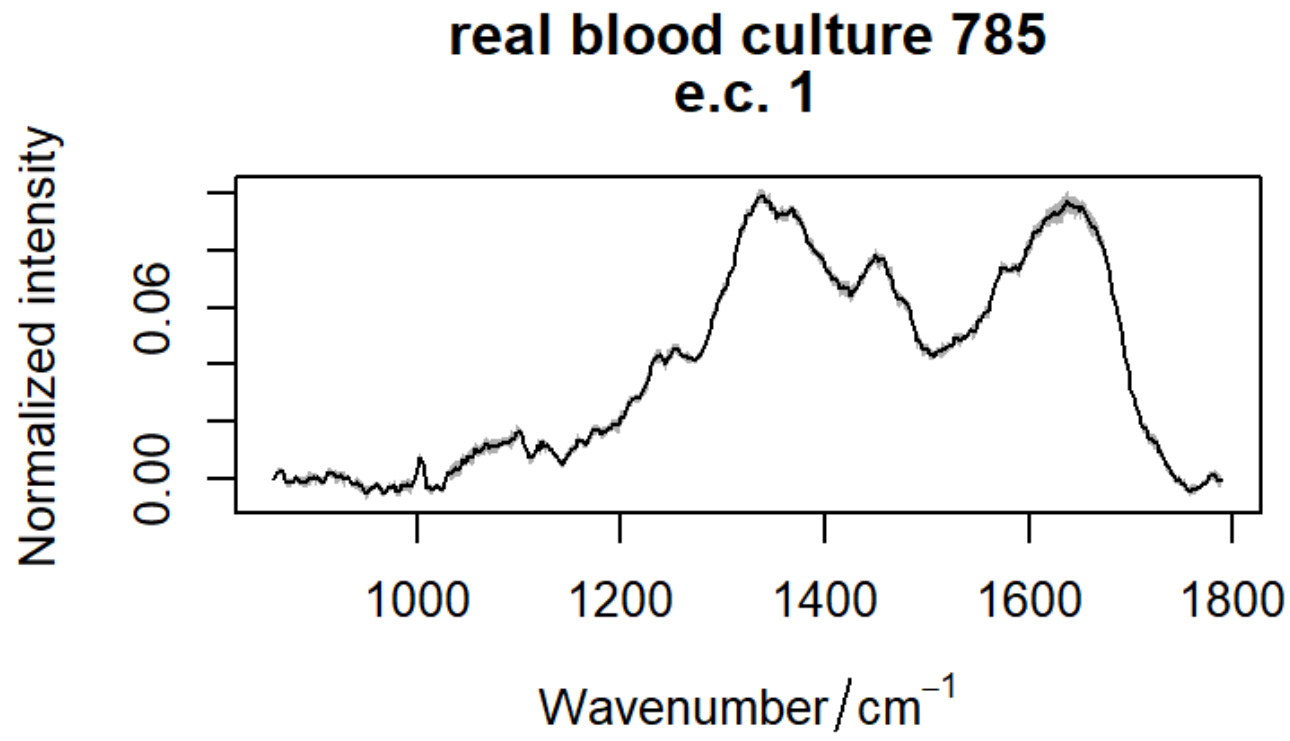

real blood culture 785
e.c. 1

E. coli 1 - CIP: 0.0000mg/l

```
## real blood culture 785 e.c. 4
```

real blood culture 785
e.c. 4

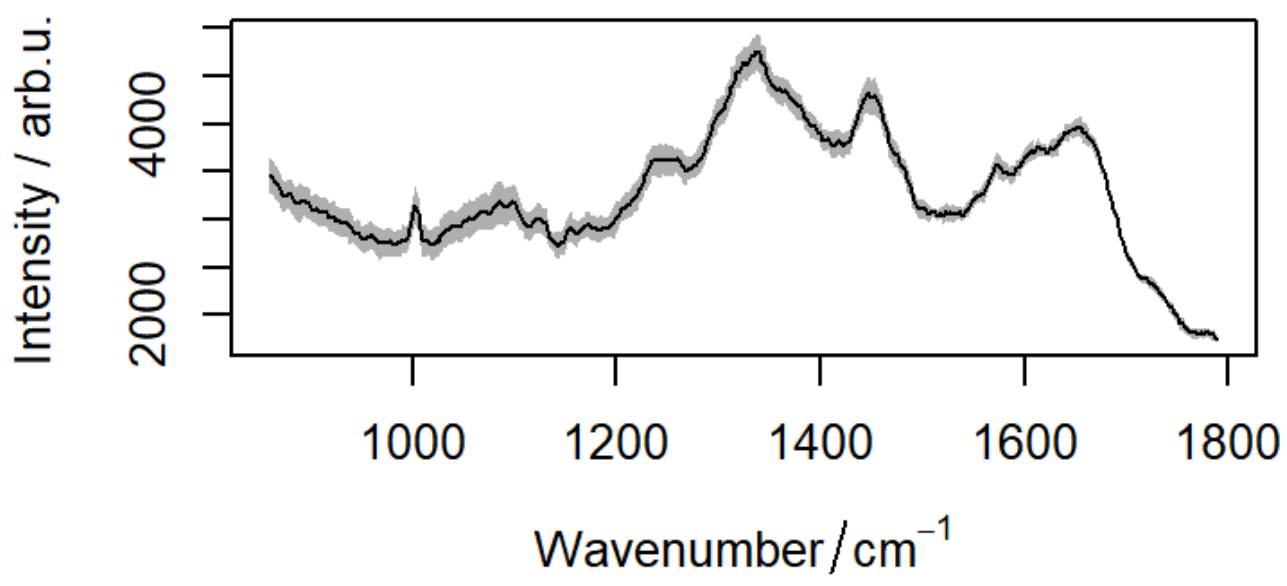

real blood culture 785
e.c. 4

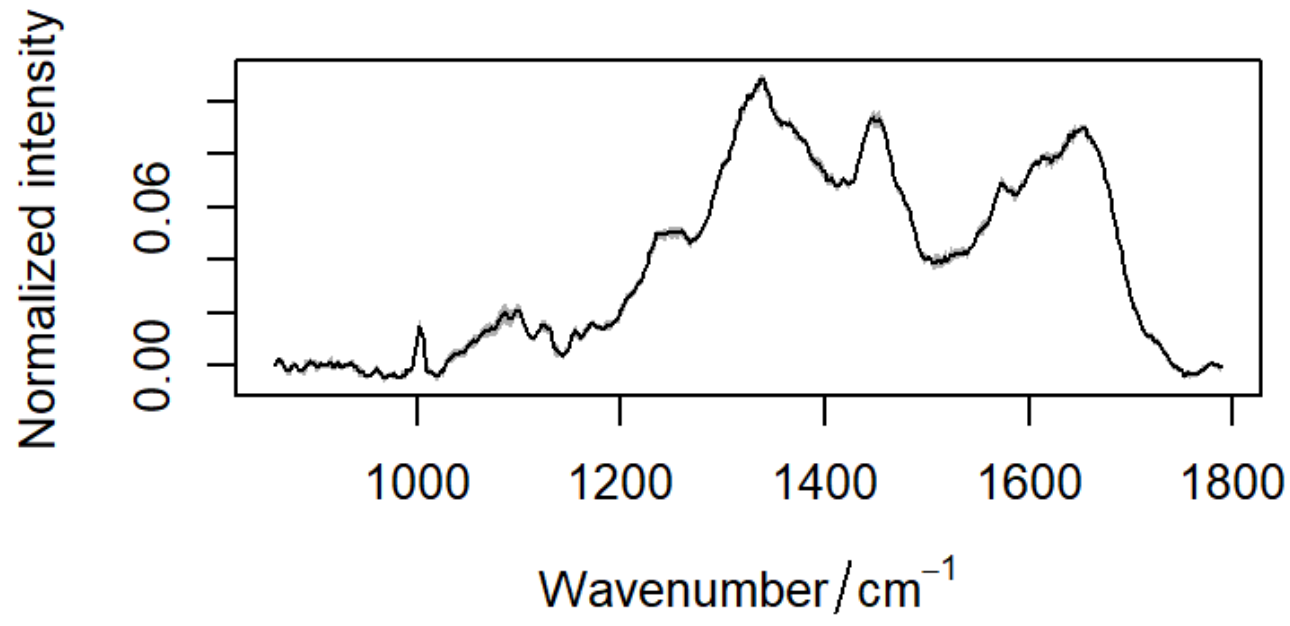

real blood culture 785
e.c. 4

E. coli 4 - CIP: 0.0000mg/l

```
## real blood culture 785 e.c. 6
```

real blood culture 785
e.c. 6

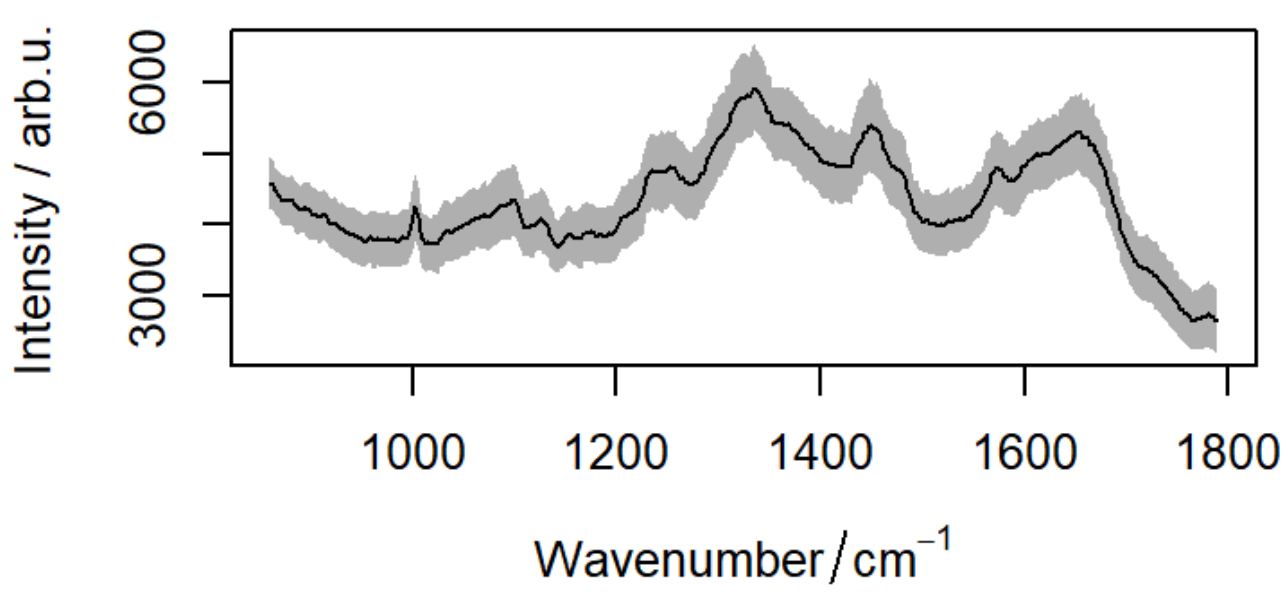

real blood culture 785
e.c. 6

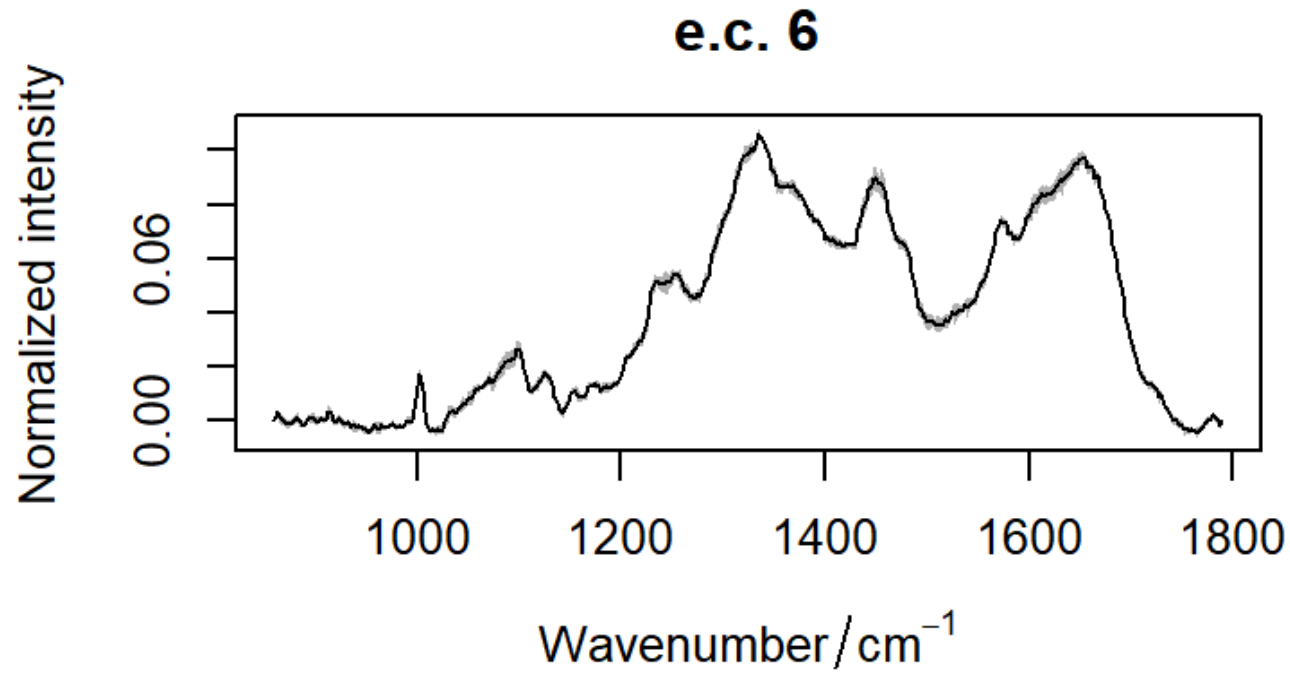

real blood culture 785
e.c. 6

E. coli 6 - CIP: 0.0000mg/l

```
## real blood culture 785 s.a. 1
```

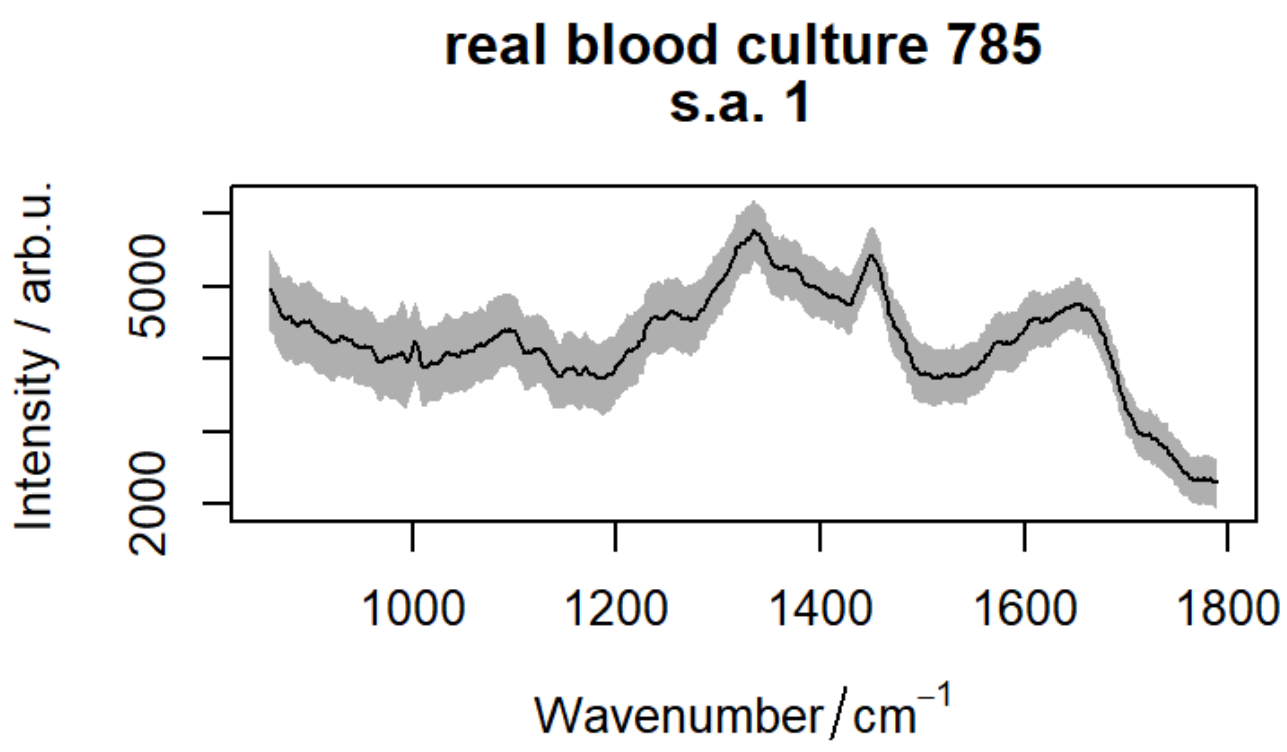

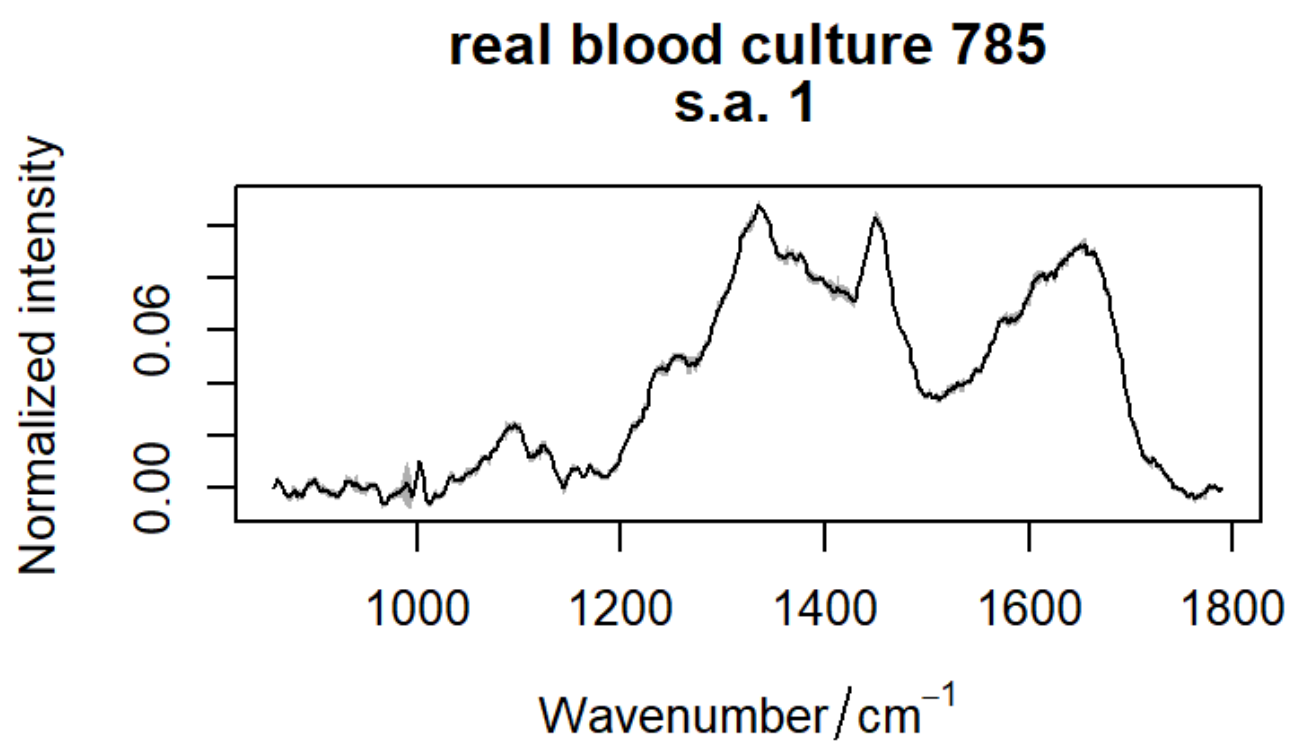

real blood culture 785
s.a. 1

S. aureus 1 - OXA: 0.0000mg/l

```
## real blood culture 785 s.a. 2
```

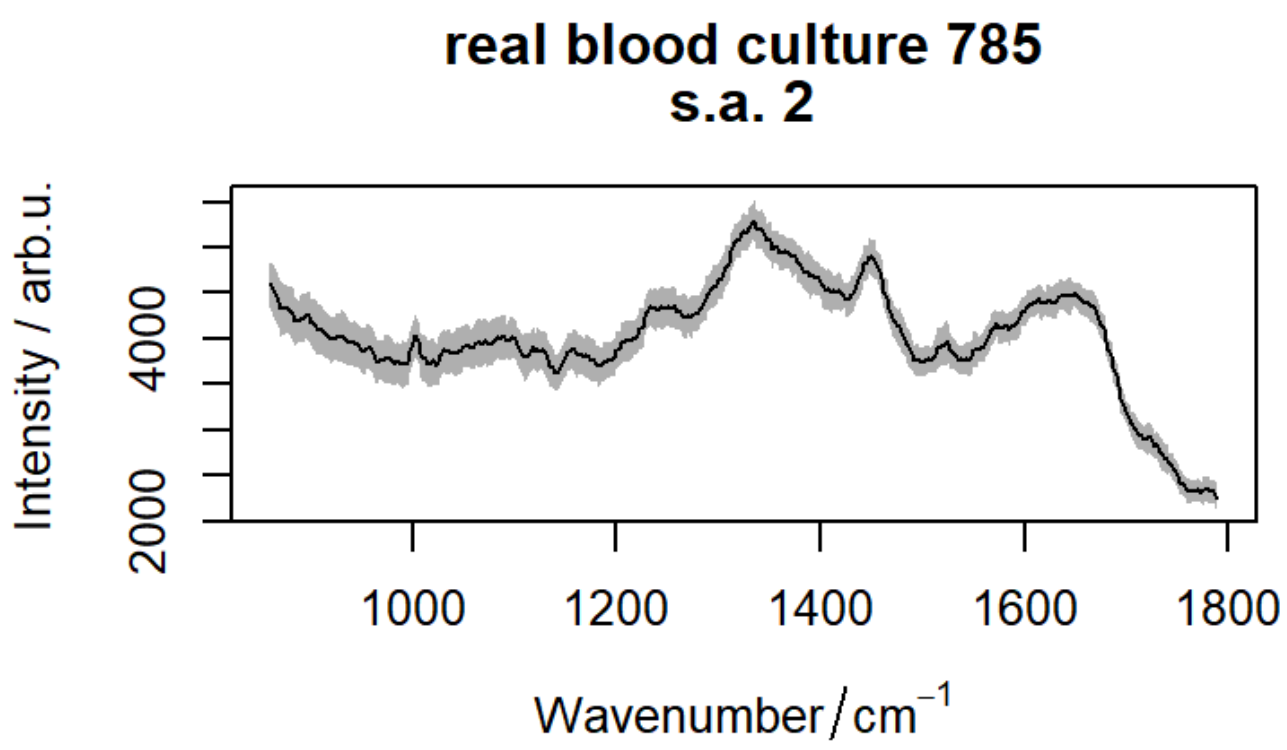

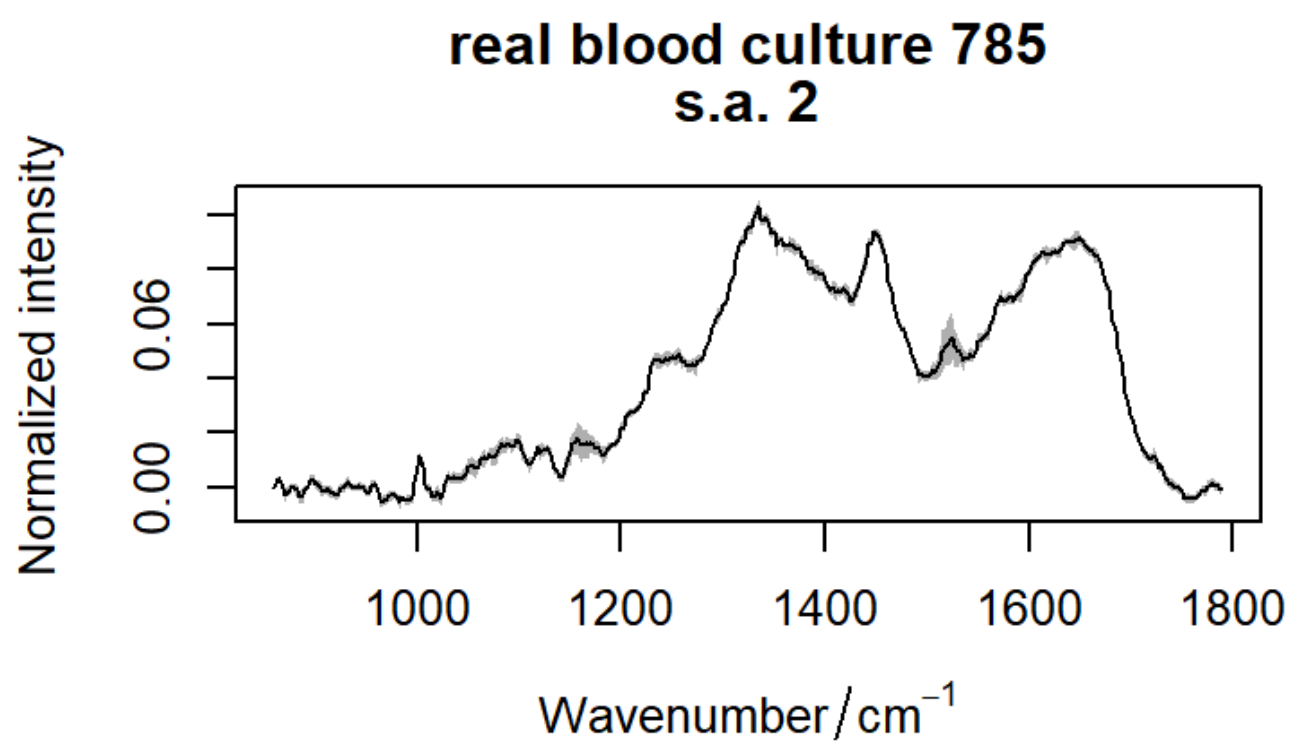

real blood culture 785
s.a. 2

S. aureus 2 - OXA: 0.0000mg/l

```
## real blood culture 785 s.a. 3
```

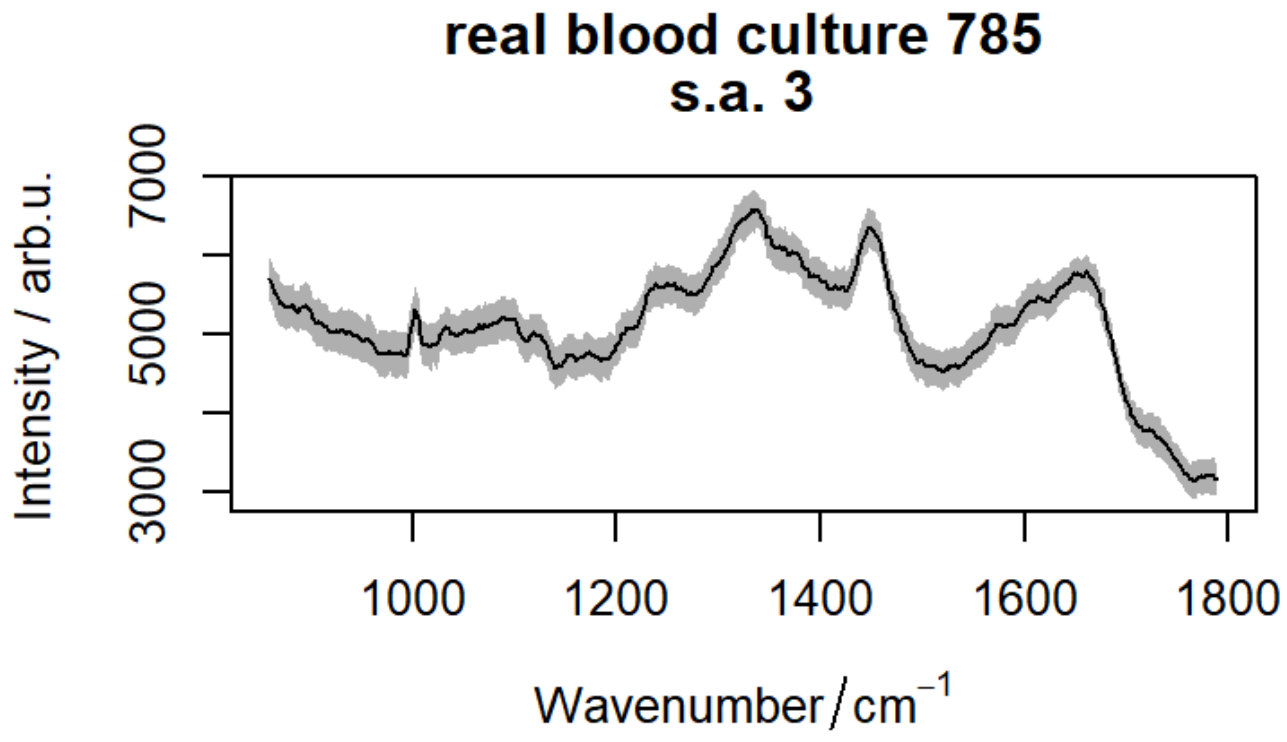

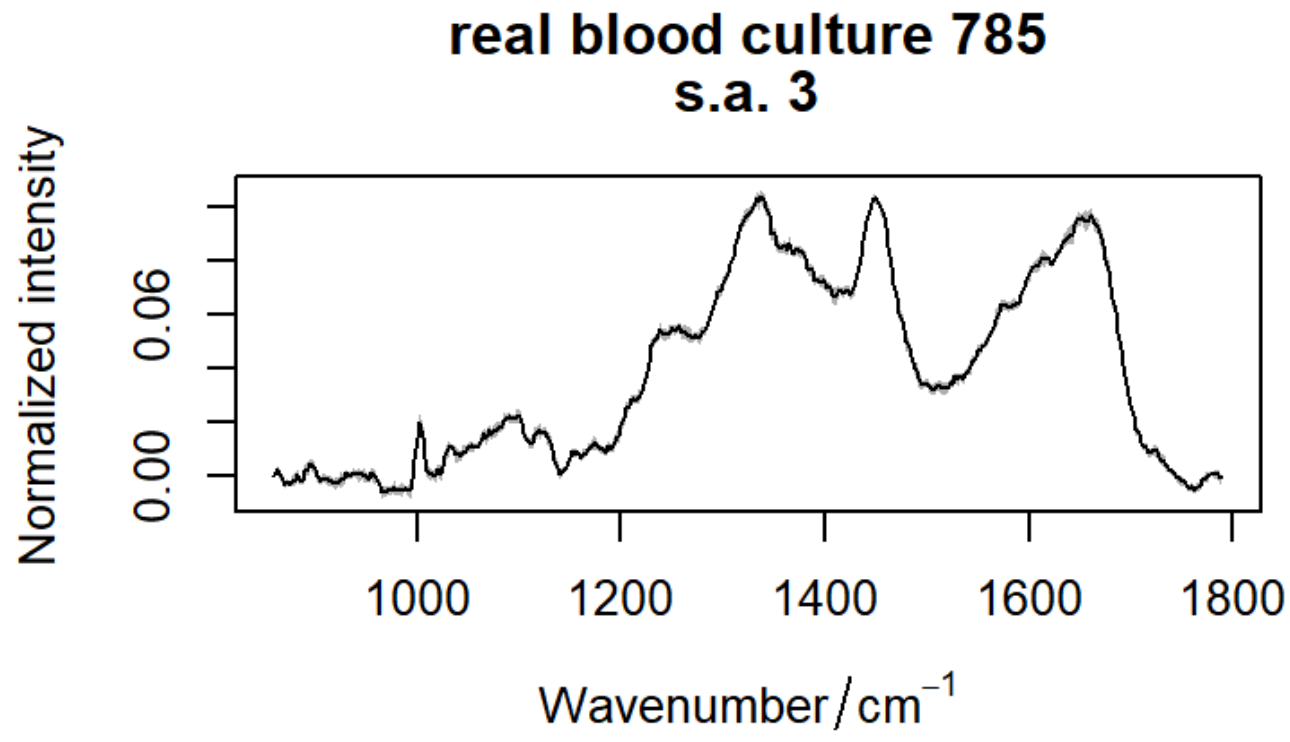

real blood culture 785
s.a. 3

S. aureus 3 - OXA: 0.0000mg/l

```
##              pred
## true          E. coli S. aureus acc sensitivity specificity
##    E. coli        18         0   1           1           1
##    S. aureus       0        18   1           1           1
```

```
##
## Balanced accuracy: 100 %
```

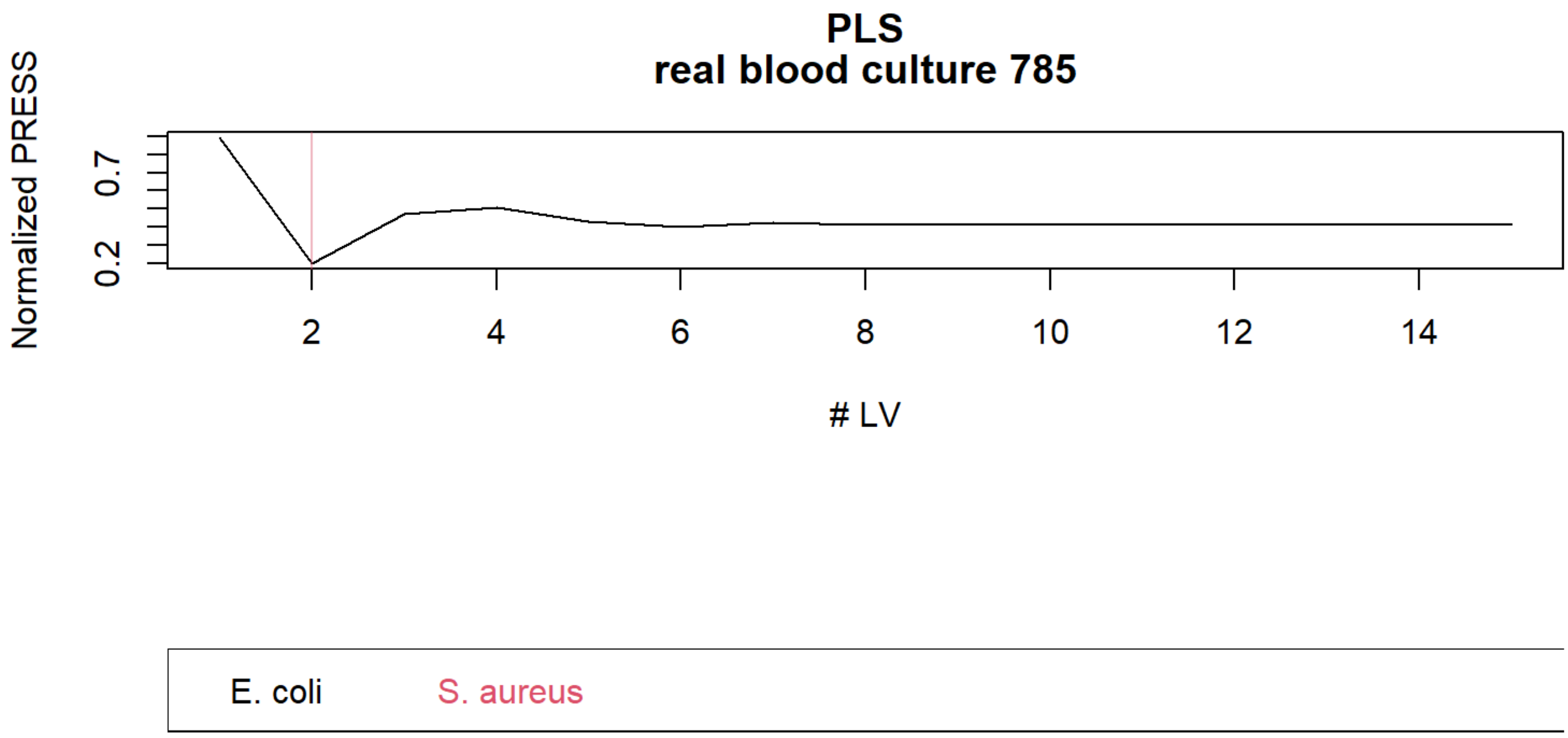

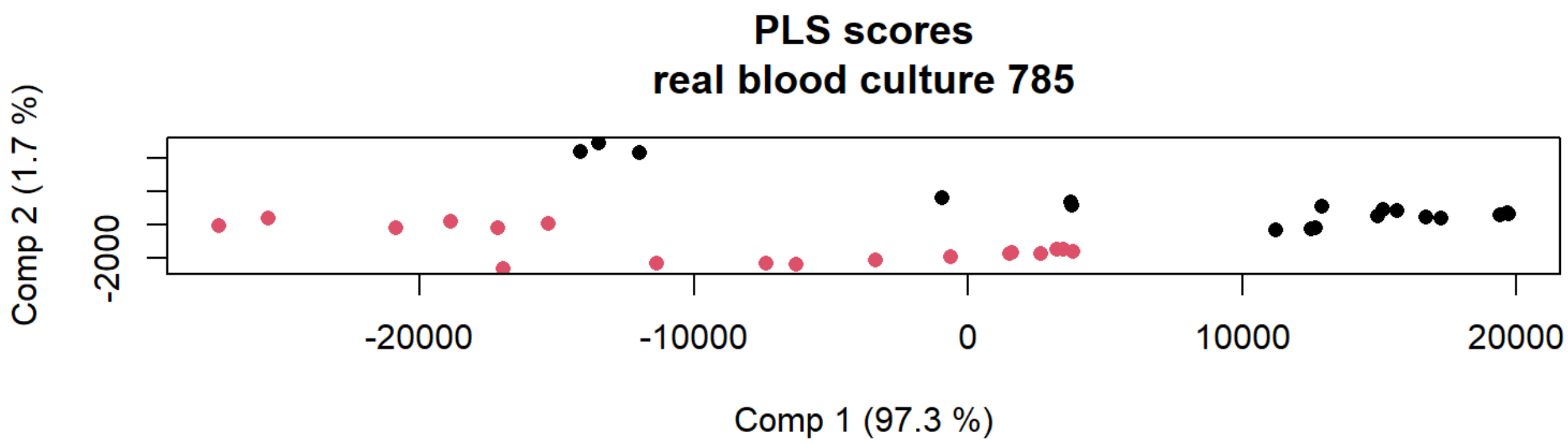
PLS scores
real blood culture 785
Comp 2 (1.7 %)
-2000
-20000
-10000
0
10000
20000
Comp 1 (97.3 %)

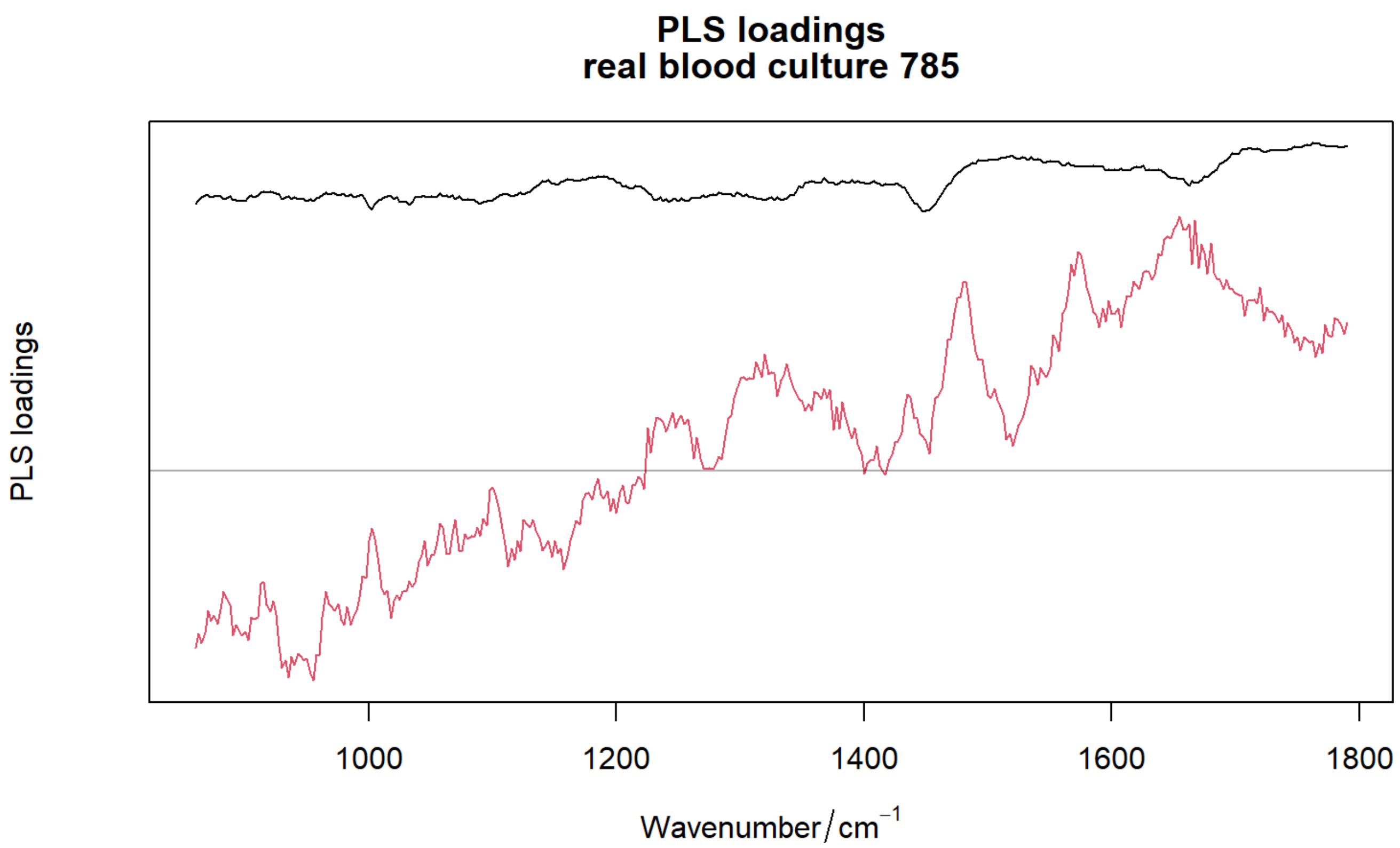
PLS loadings
real blood culture 785
PLS loadings
1000
1200
1400
1600
1800
Wavenumber / cm⁻¹

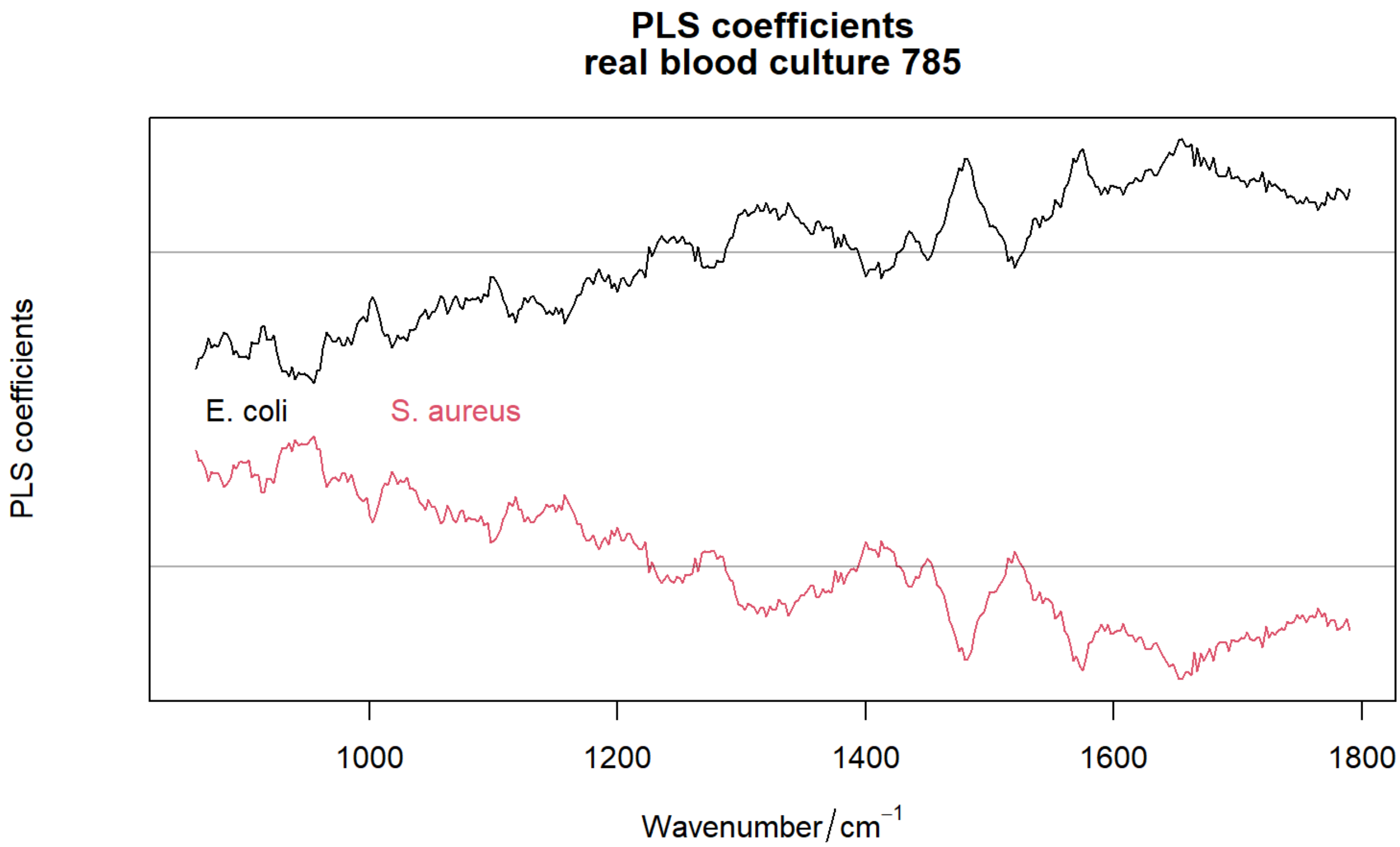
PLS coefficients
real blood culture 785
PLS coefficients
E. coli
S. aureus
1000
1200
1400
1600
1800
Wavenumber / cm⁻¹

SI 7: CFU pre and post isolation from artificial blood culture

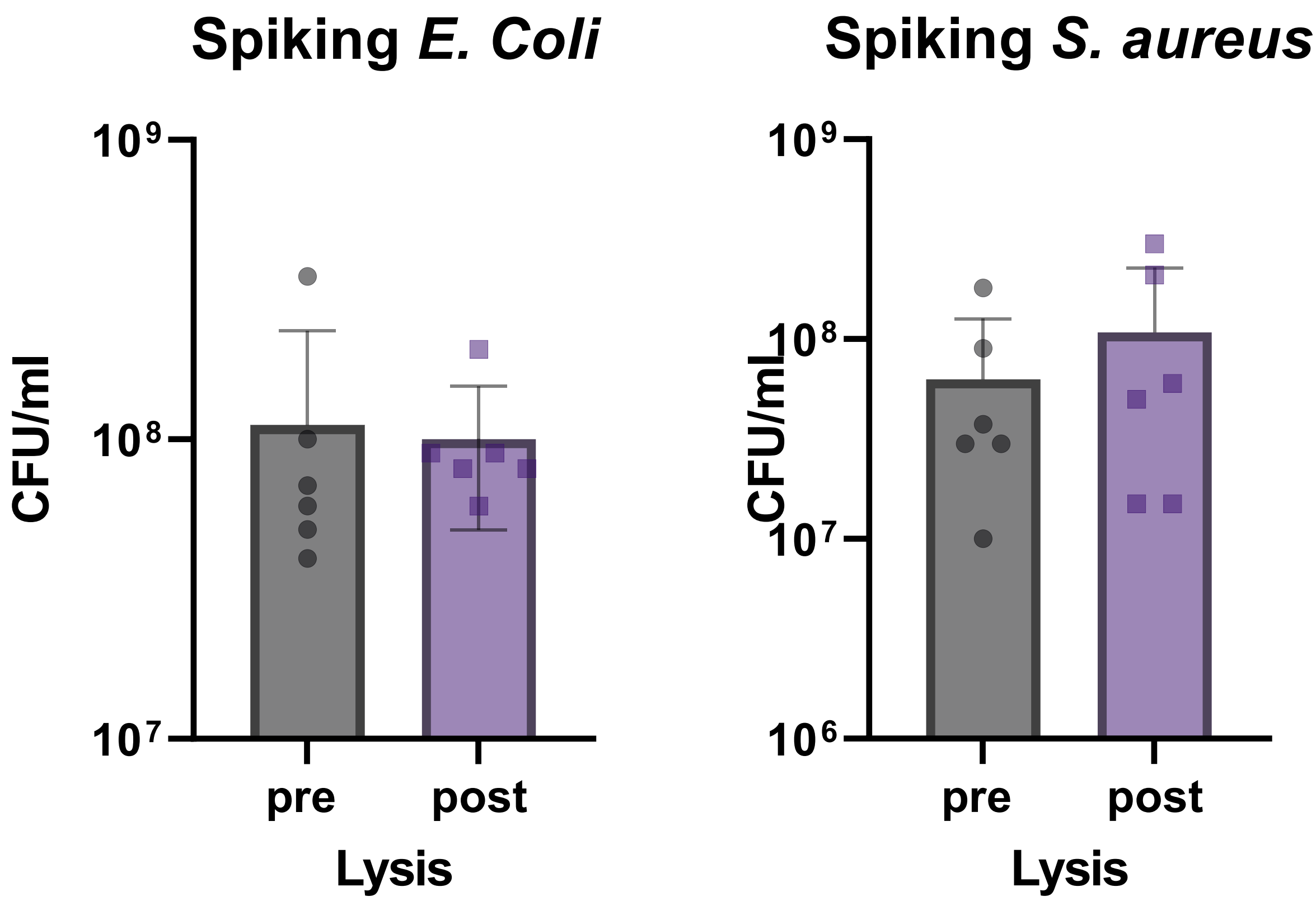

Infecto_Xplore

```
## Laser: 785
```

```
## blood culture 785 2023-01-11_MP_Ec+CIP_spectra_RG
```

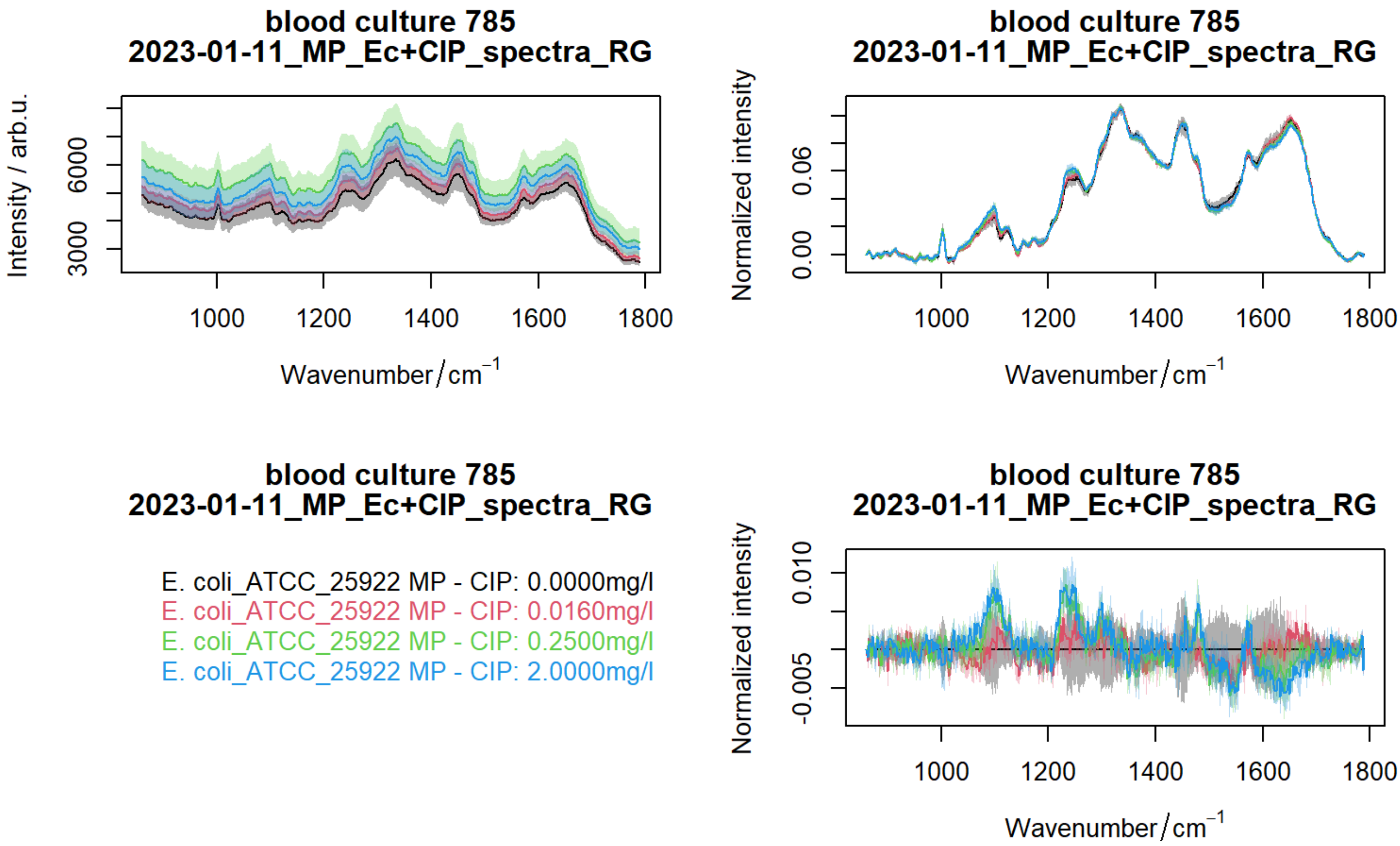

```
## blood culture 785 2023-01-23_MP_Ec+CIP_spectra_RG
```

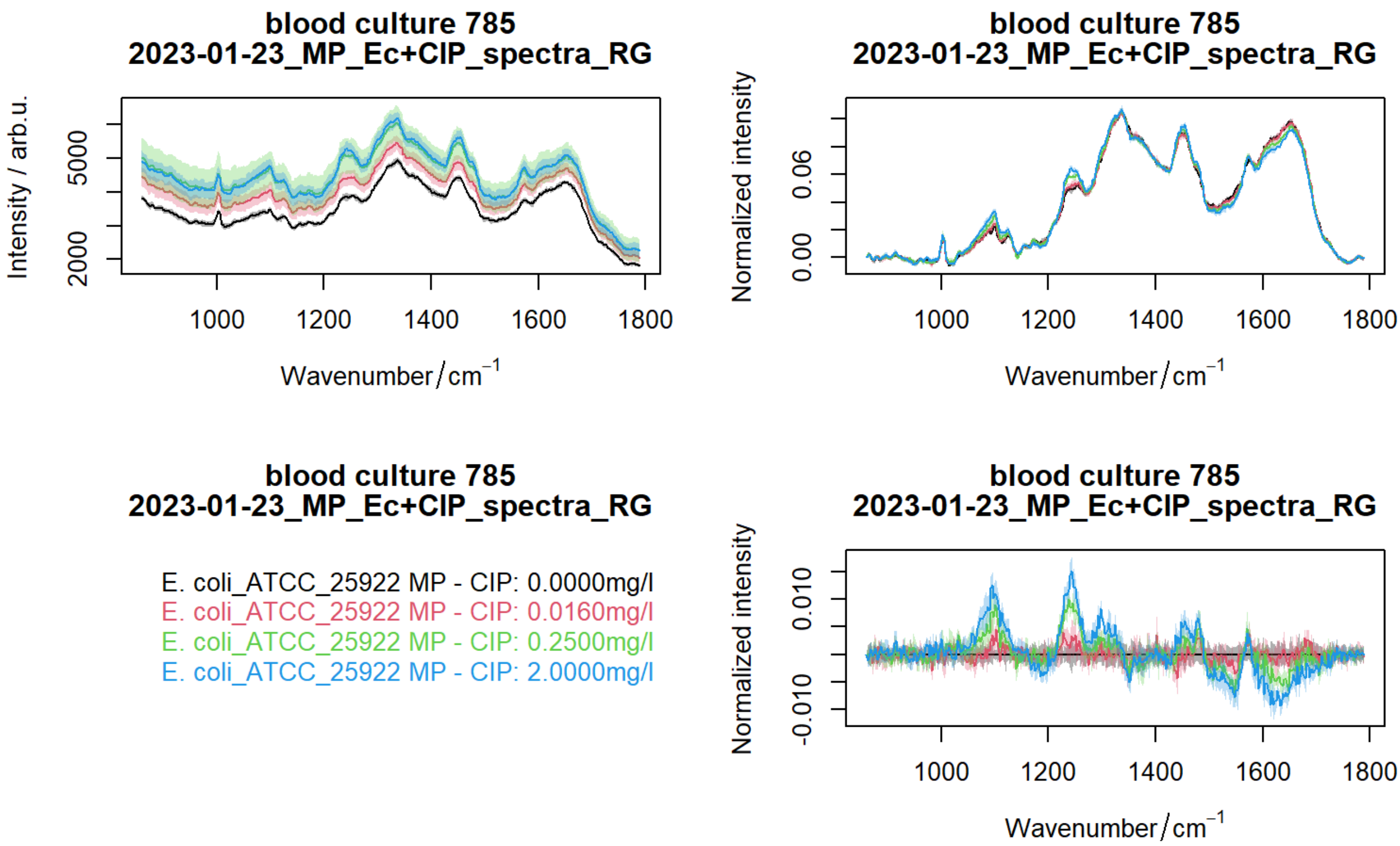


```
## blood culture 785 2023-05-03_MP_Sa+OXA_spectra_RG
```

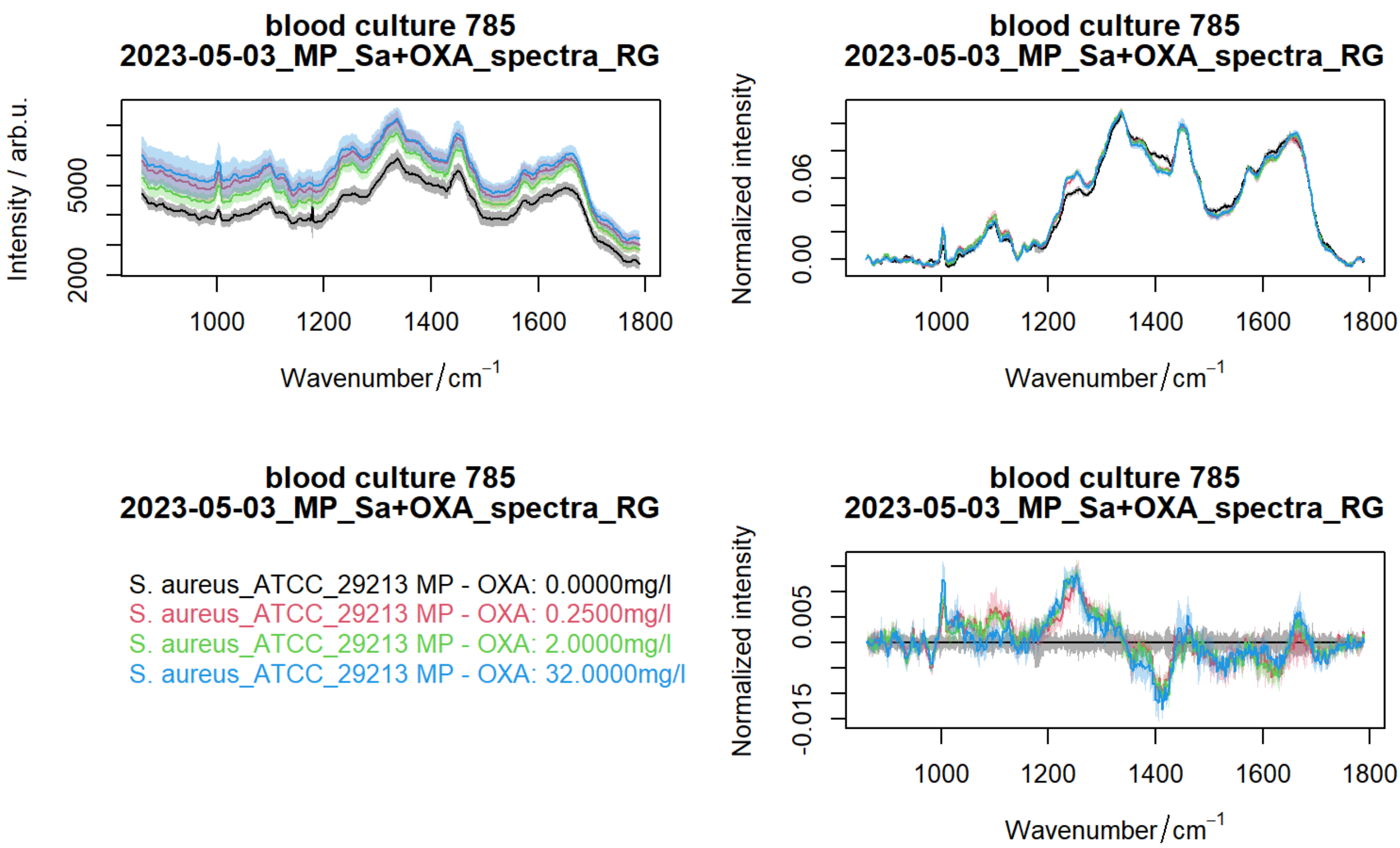

```
## blood culture 785 2023-06-15_MP_Ec+CIP_spectra_RG
```

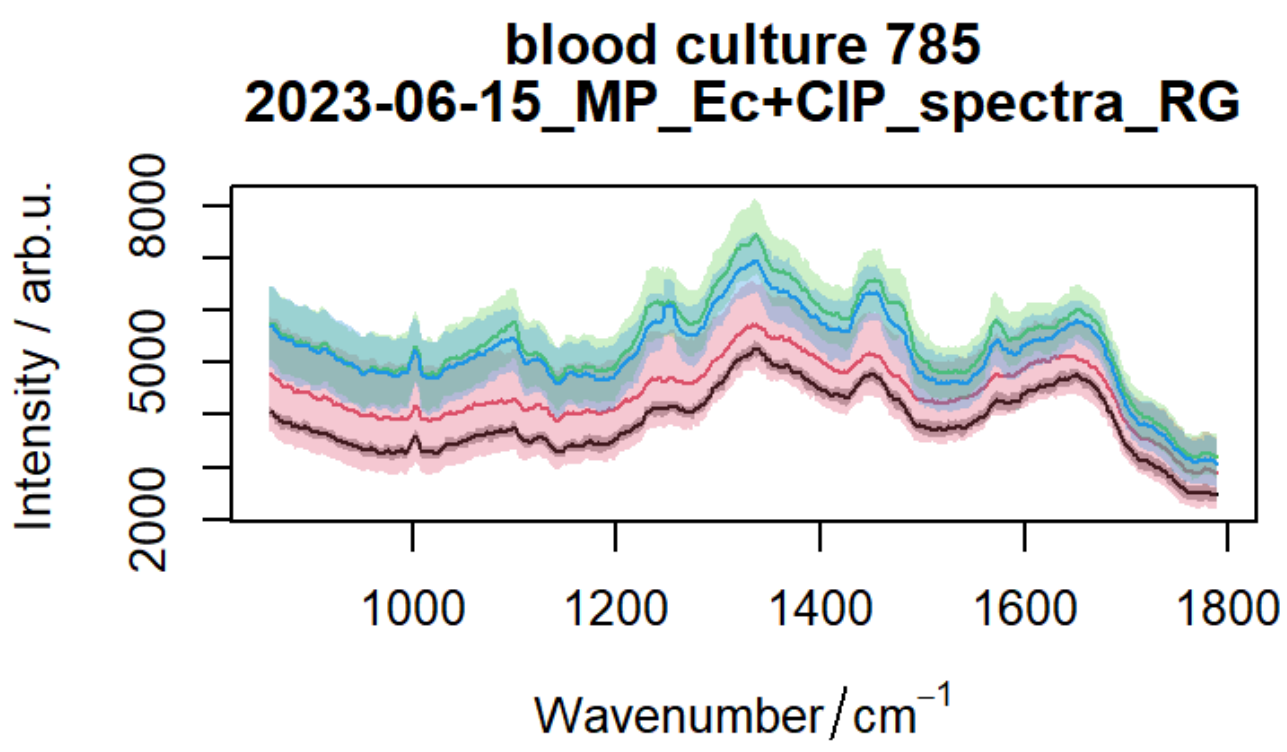

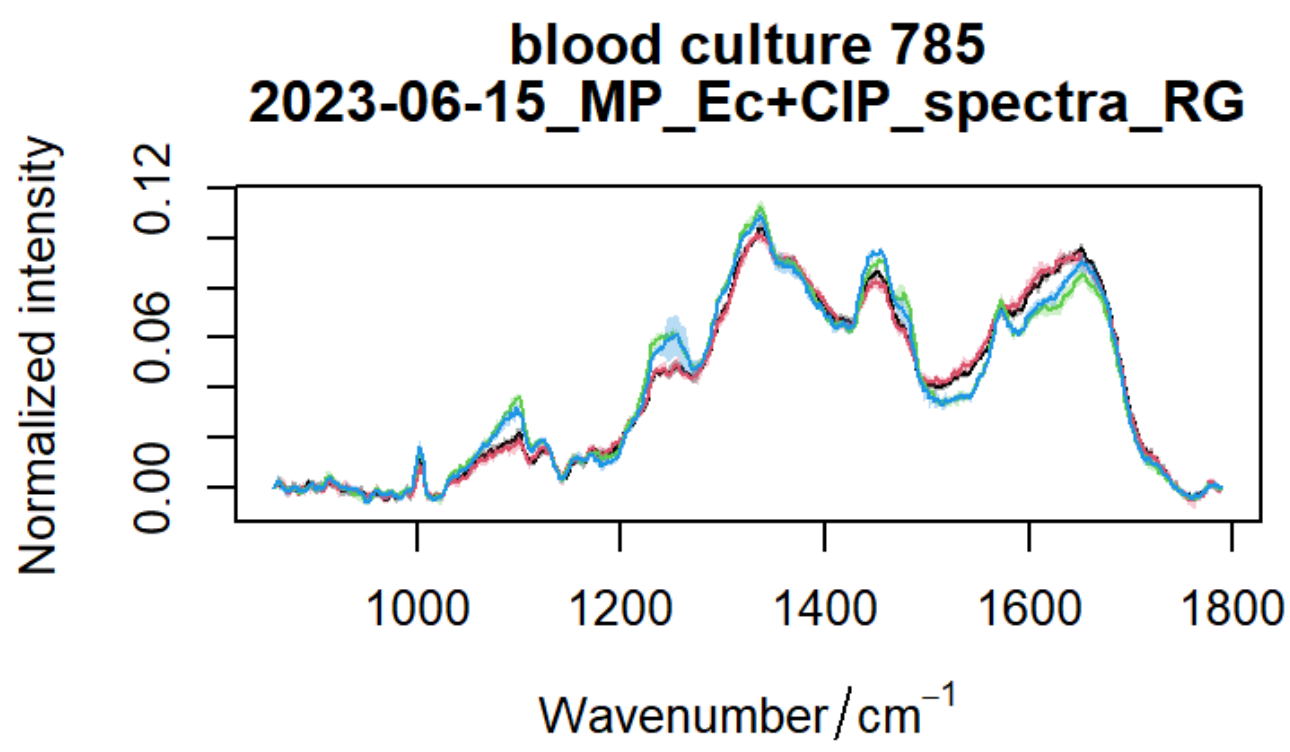

blood culture 785
2023-06-15_MP_Ec+CIP_spectra_RG

E. coli_ATCC_25922 MP - CIP: 0.0000mg/l
E. coli_ATCC_25922 MP - CIP: 0.0160mg/l
E. coli_ATCC_25922 MP - CIP: 0.2500mg/l
E. coli_ATCC_25922 MP - CIP: 2.0000mg/l

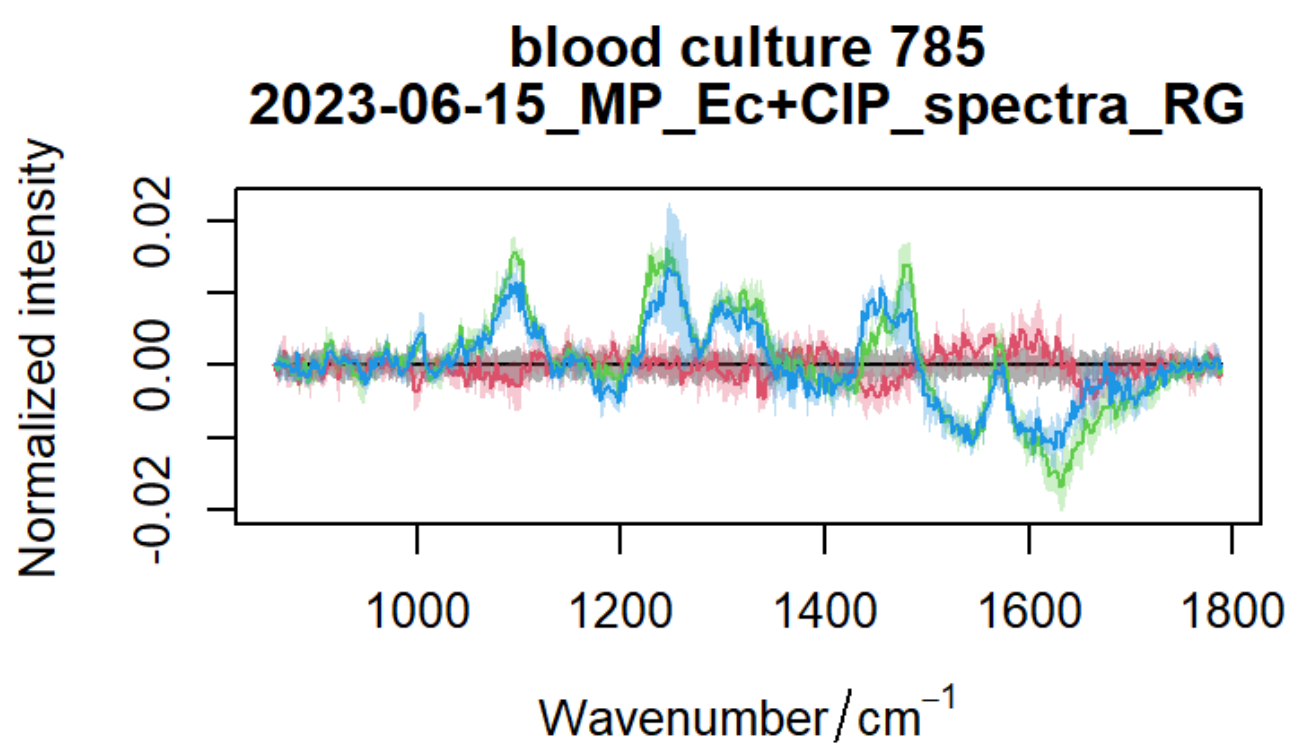


```
## blood culture 785 2023-06-20_MP_Sa+OXA_532 BK missing_spectra_RG
```

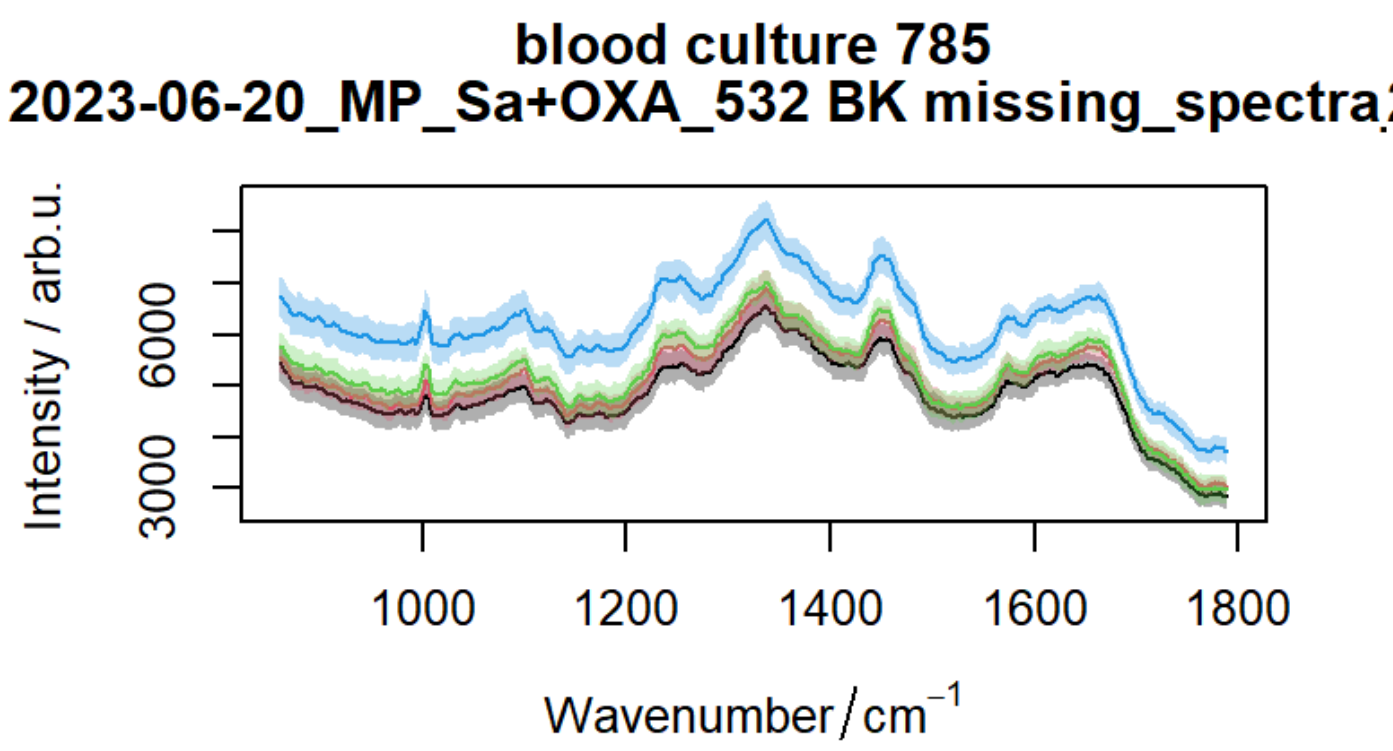

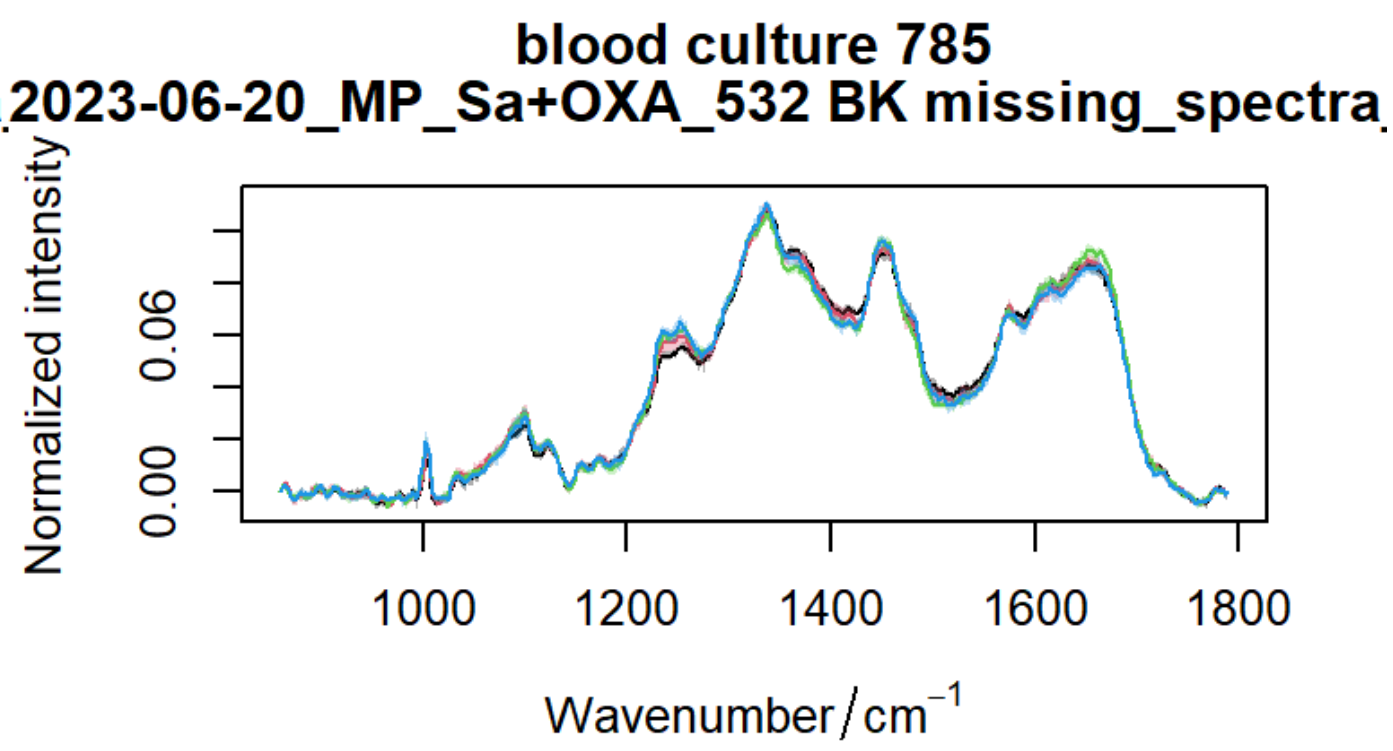

blood culture 785
2023-06-20_MP_Sa+OXA_532 BK missing_spectra

S. aureus_ATCC_29213 MP - OXA: 0.0000mg/l
S. aureus_ATCC_29213 MP - OXA: 0.2500mg/l
S. aureus_ATCC_29213 MP - OXA: 2.0000mg/l
S. aureus_ATCC_29213 MP - OXA: 32.0000mg/l

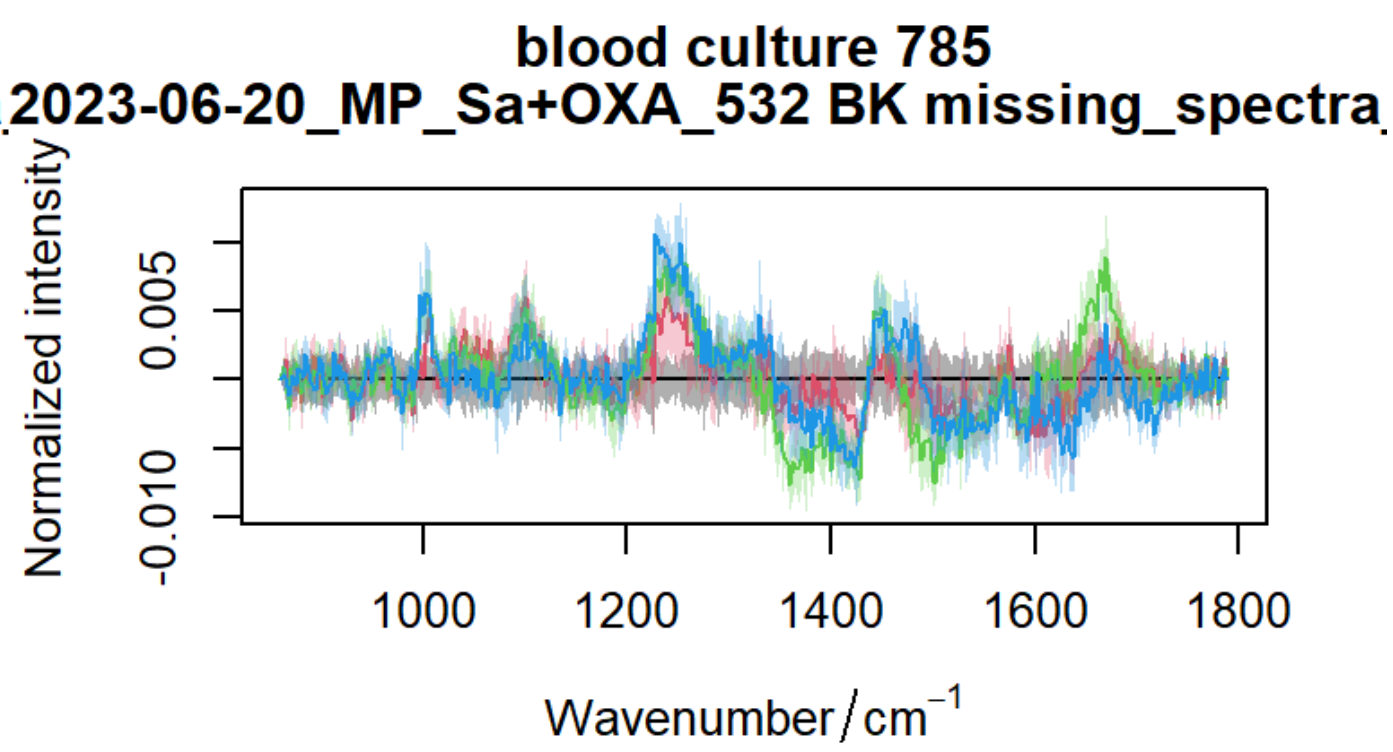

```
## blood culture 785 2023-11-12_MP_Sa+OXA_spectra_RG
```

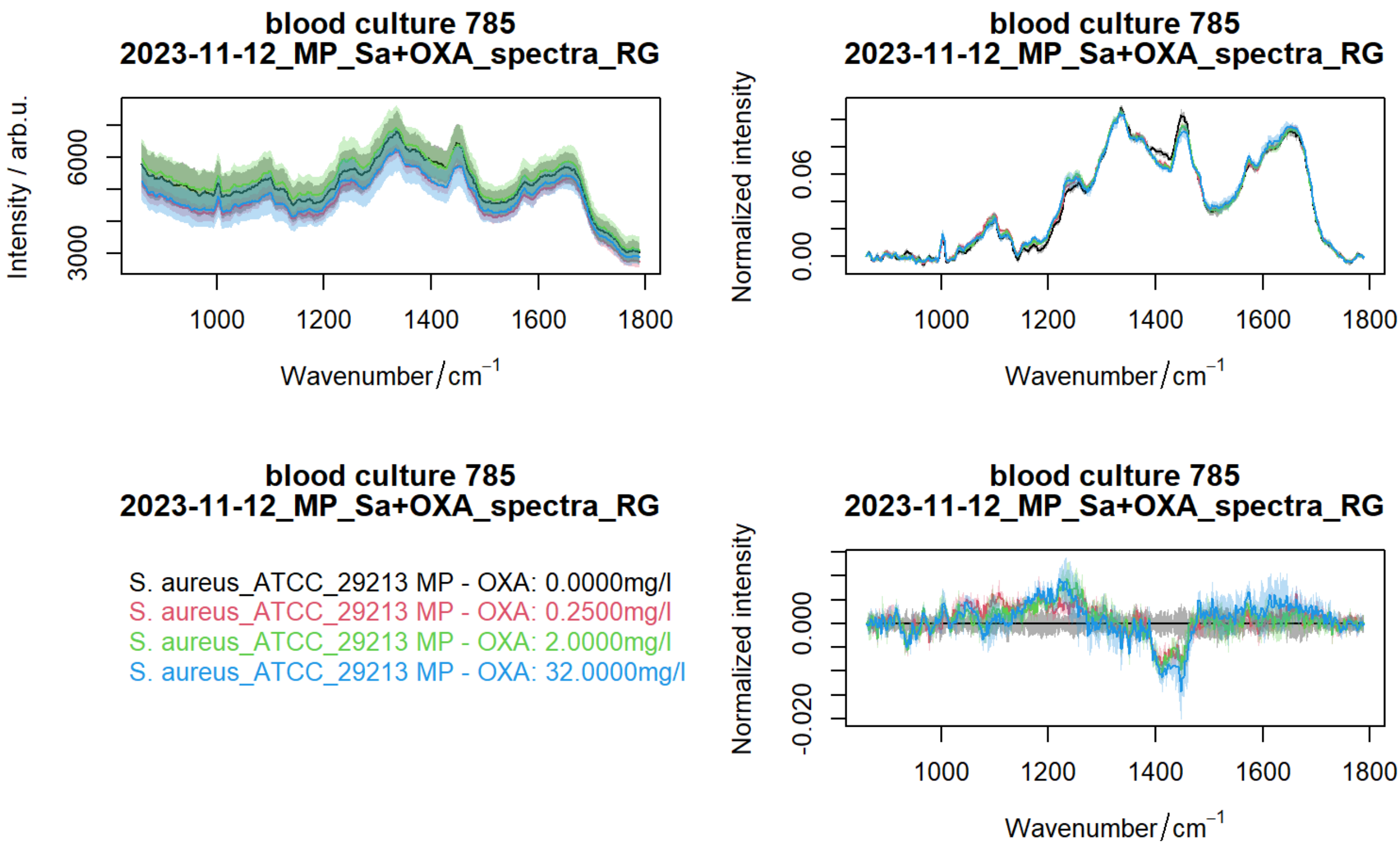

```
##
## growth expected:
```

```
##
##              no yes
##   CIP 0       0  18
##   CIP 0.016   0  18
##   CIP 0.25   18   0
##   CIP 2      18   0
##   OXA 0       0  18
##   OXA 0.25   18   0
##   OXA 2      18   0
##   OXA 32     18   0
```

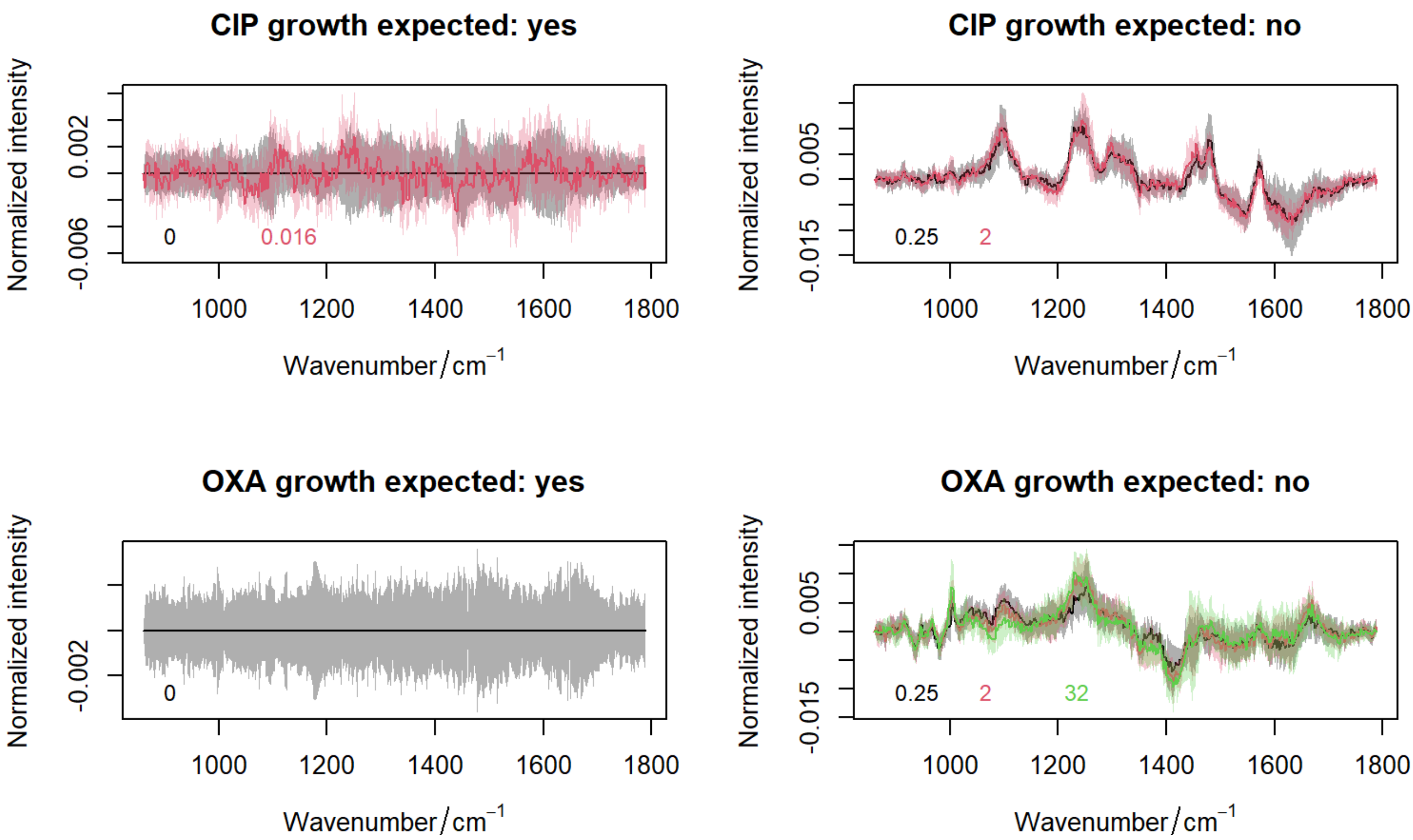

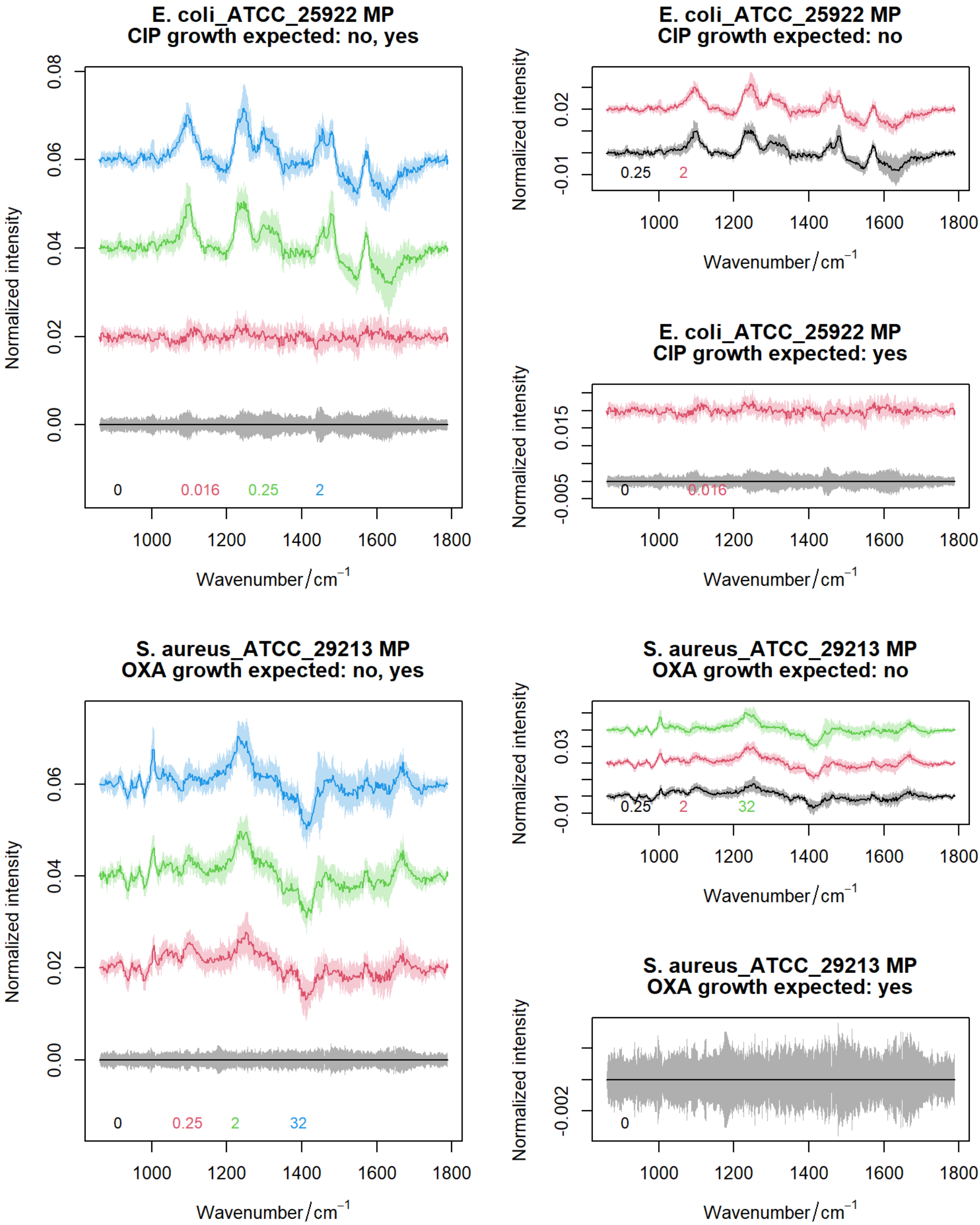
E. coli_ATCC_25922 MP
CIP growth expected: no, yes
Normalized intensity
0.00
0.02
0.04
0.06
0.08
0
0.016
0.25
2
1000
1200
1400
1600
1800
Wavenumber/cm^{-1}
E. coli_ATCC_25922 MP
CIP growth expected: no
Normalized intensity
-0.01
0.02
0.25
2
Wavenumber/cm^{-1}
E. coli_ATCC_25922 MP
CIP growth expected: yes
Normalized intensity
-0.005
0.015
0
0.016
Wavenumber/cm^{-1}
S. aureus_ATCC_29213 MP
OXA growth expected: no, yes
Normalized intensity
0.00
0.02
0.04
0.06
0
0.25
2
32
Wavenumber/cm^{-1}
S. aureus_ATCC_29213 MP
OXA growth expected: no
Normalized intensity
-0.01
0.03
0.25
2
32
Wavenumber/cm^{-1}
S. aureus_ATCC_29213 MP
OXA growth expected: yes
Normalized intensity
-0.002
0
Wavenumber/cm^{-1}

```
##
## ===============
##  CIP E. coli
##
##      pred
## true  no yes       acc sensitivity specificity
##   no  30   6 0.9166667   0.8333333   1.0000000
##   yes  0  36 0.9166667   1.0000000   0.8333333
##
## Balanced accuracy: 91.7 %
```

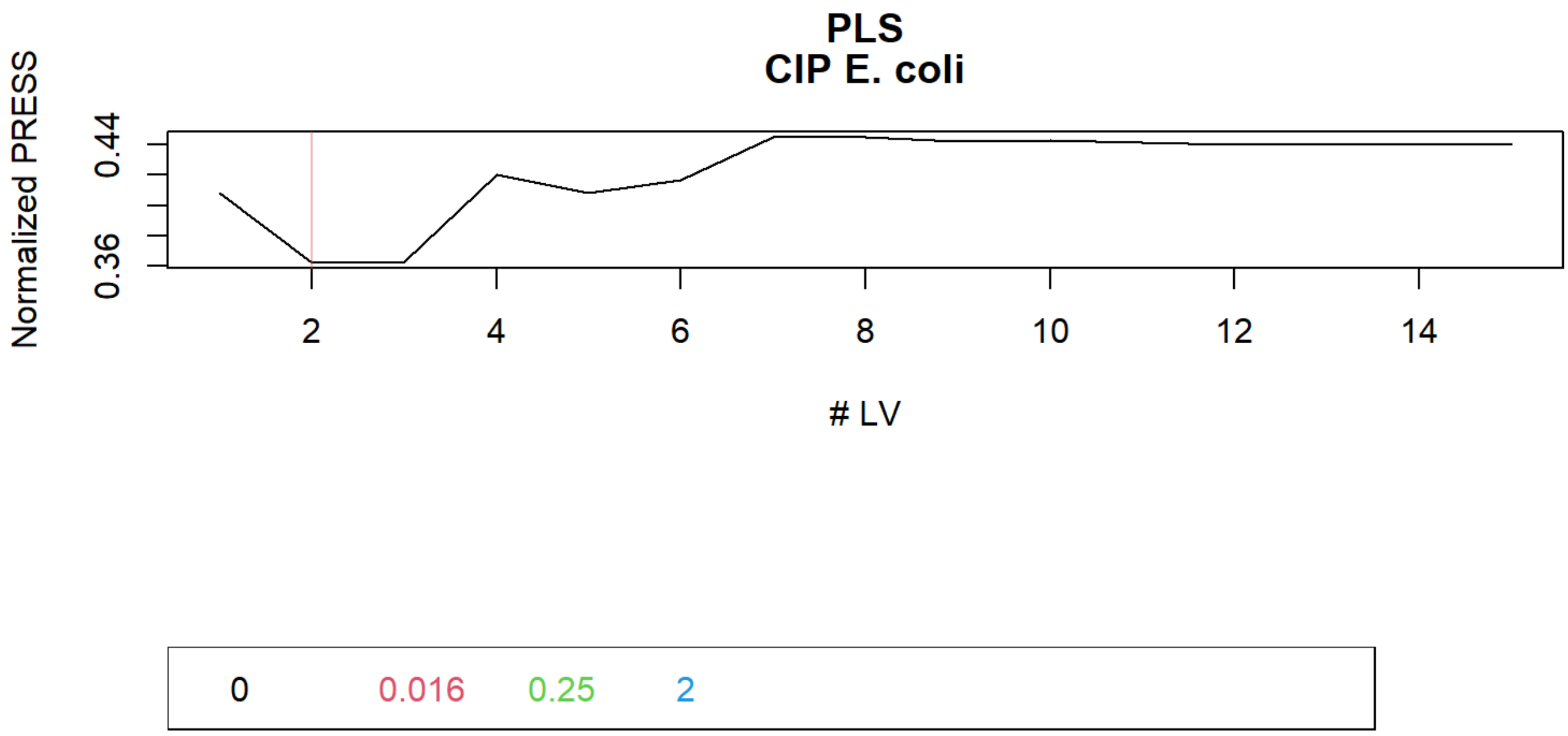

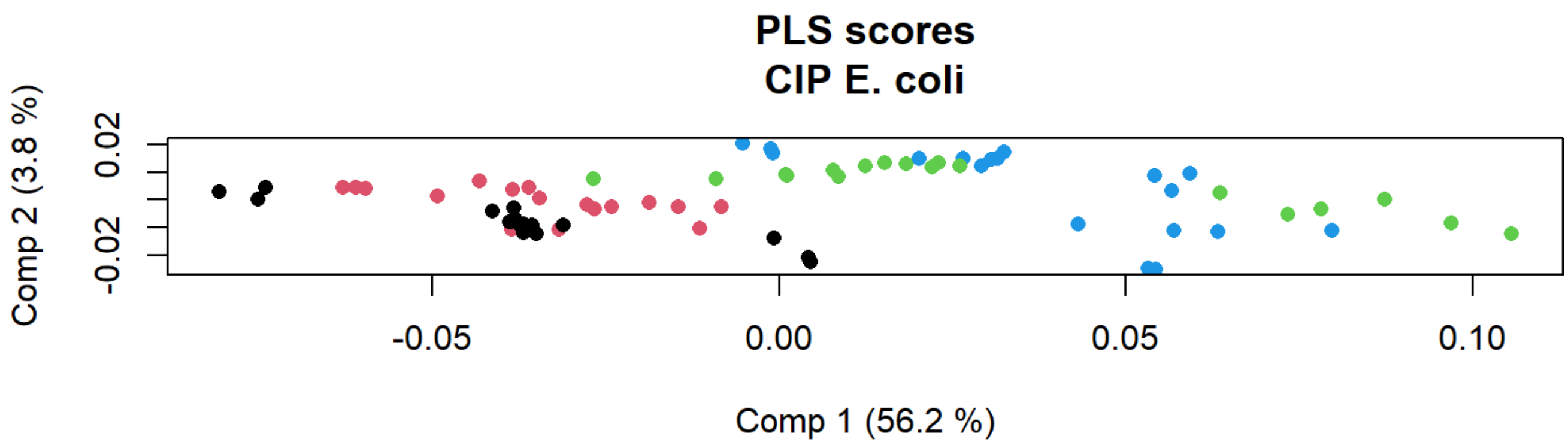
PLS scores
CIP E. coli
Comp 2 (3.8 %)
0.02
-0.02
-0.05
0.00
0.05
0.10
Comp 1 (56.2 %)

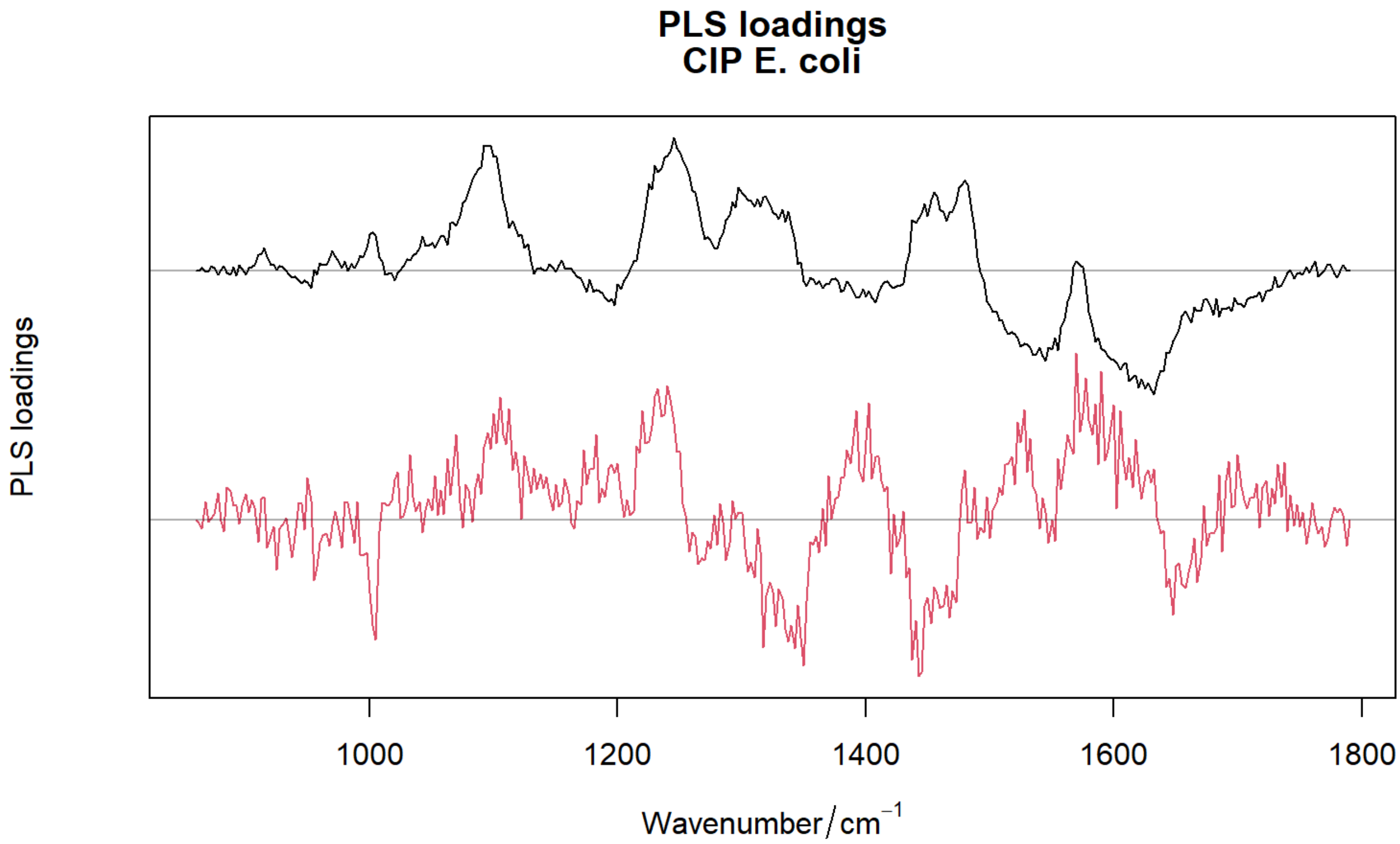
PLS loadings
CIP E. coli
PLS loadings
1000
1200
1400
1600
1800
Wavenumber / cm⁻¹

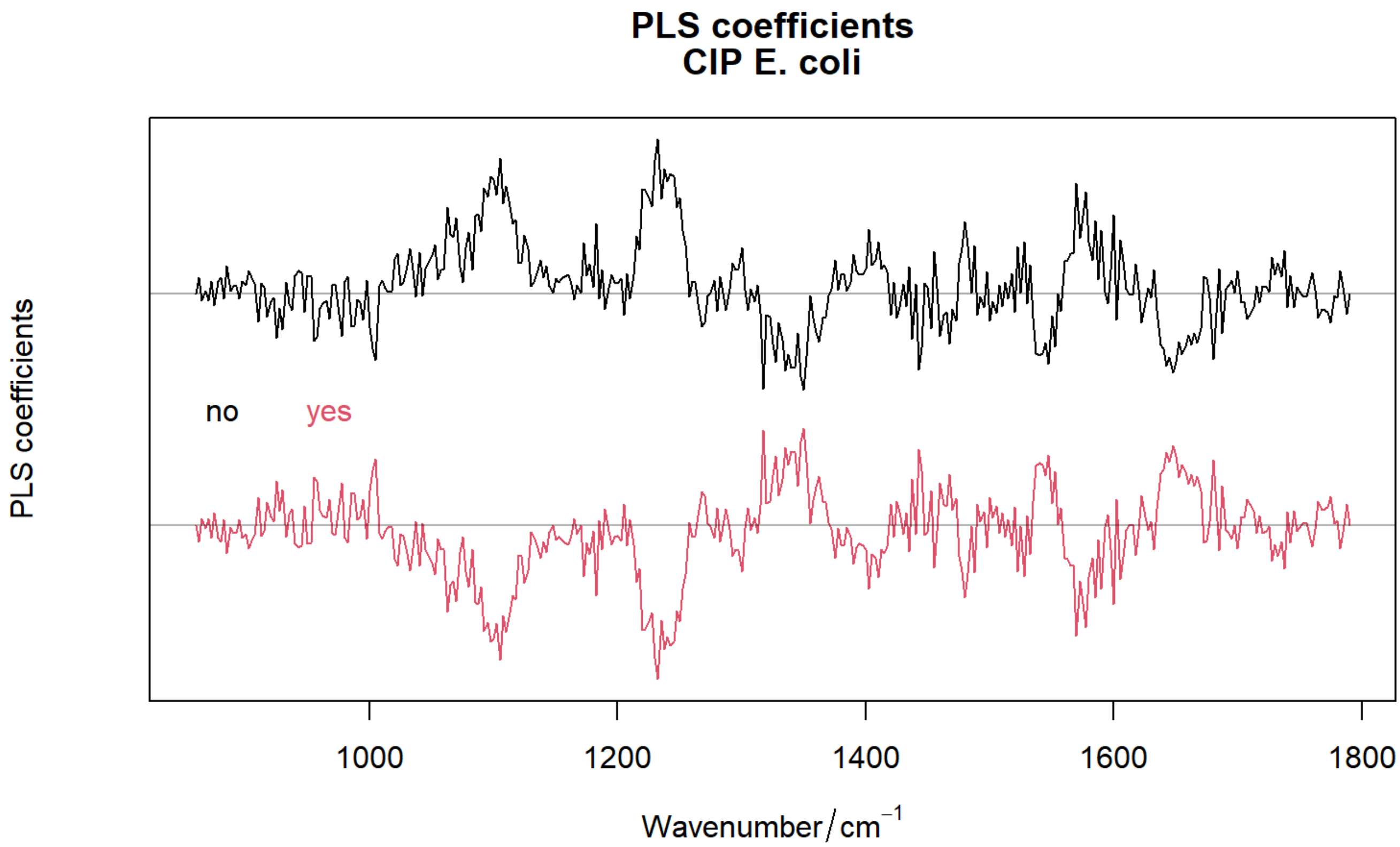
PLS coefficients
CIP E. coli
PLS coefficients
no
yes
1000
1200
1400
1600
1800
Wavenumber / cm^{-1}

```
##
## ===============
##  OXA S. aureus
##
##      pred
## true  no yes       acc sensitivity specificity
##   no  51   3 0.9583333   0.9444444   1.0000000
##   yes  0  18 0.9583333   1.0000000   0.9444444
##
## Balanced accuracy: 97.2 %
```

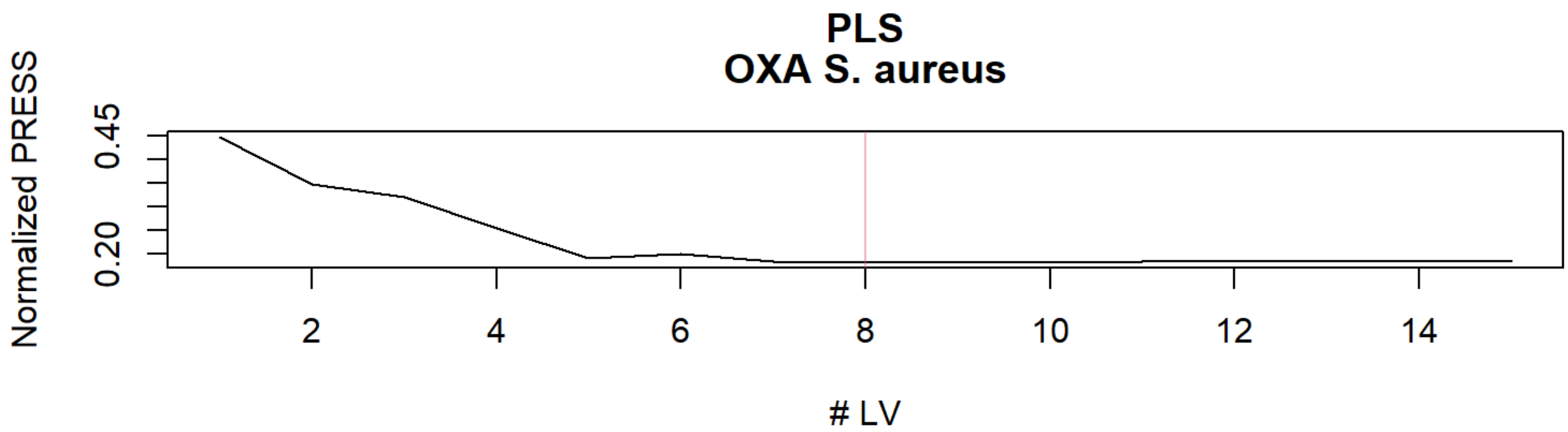

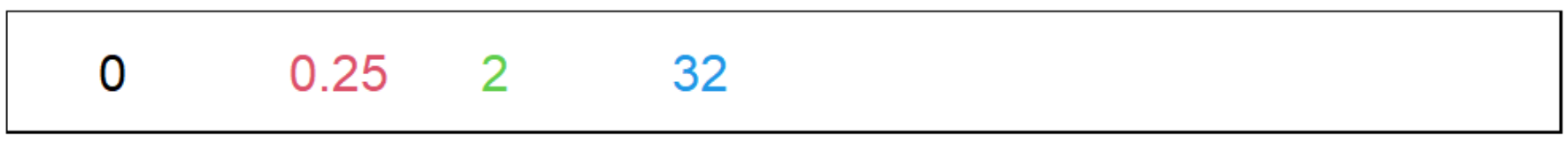

PLS scores
OXA S. aureus

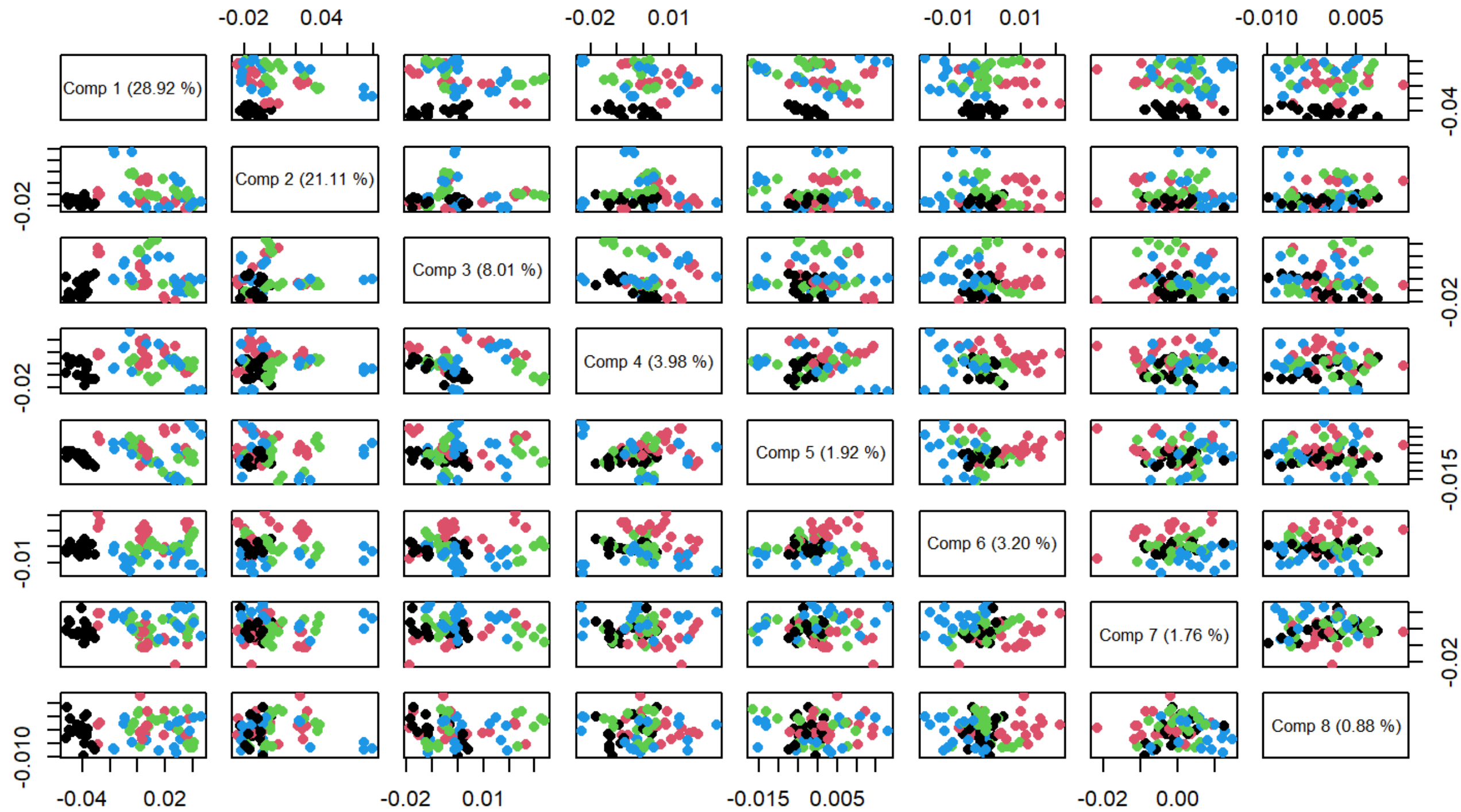

PLS loadings
OXA S. aureus

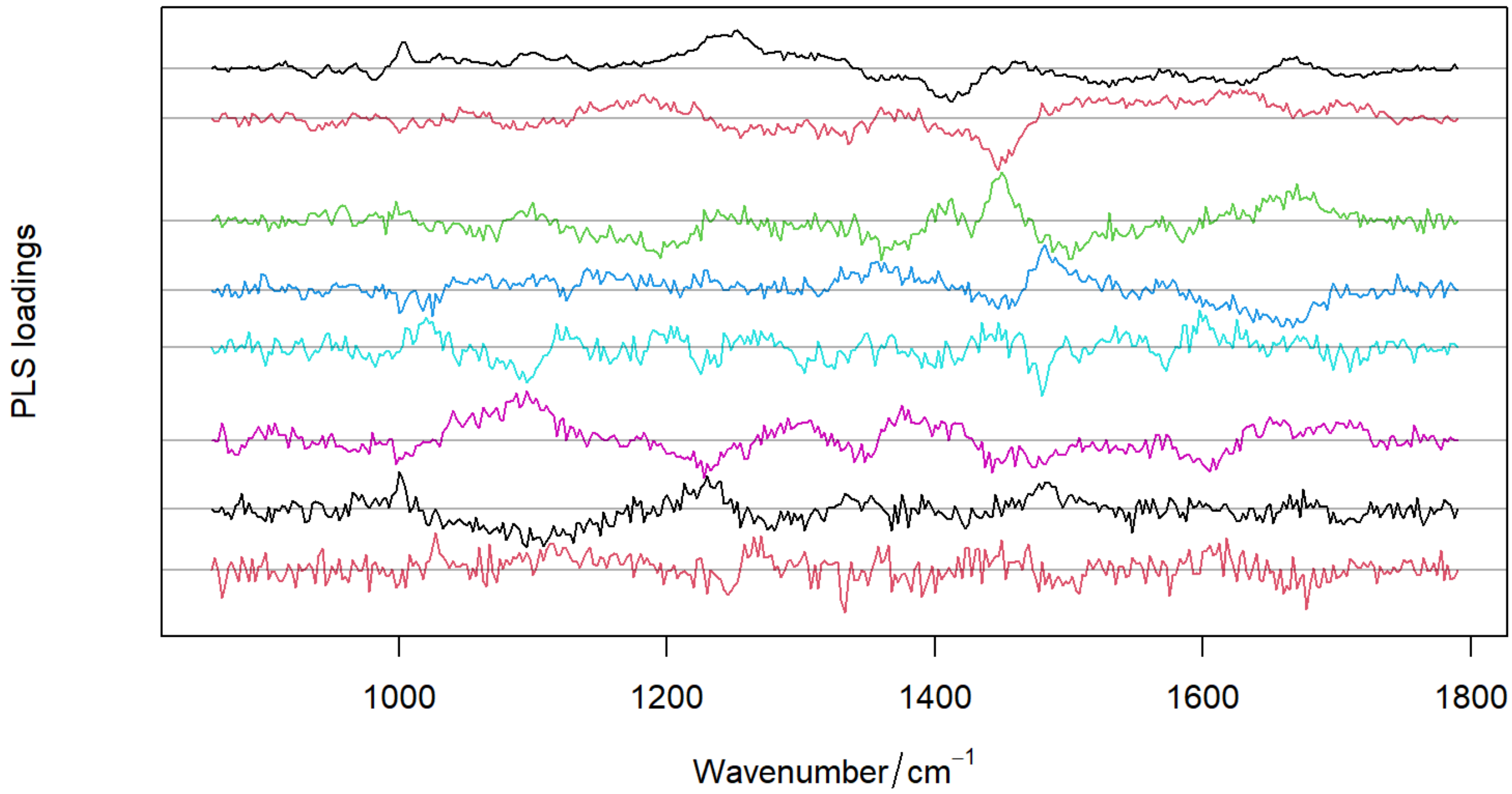

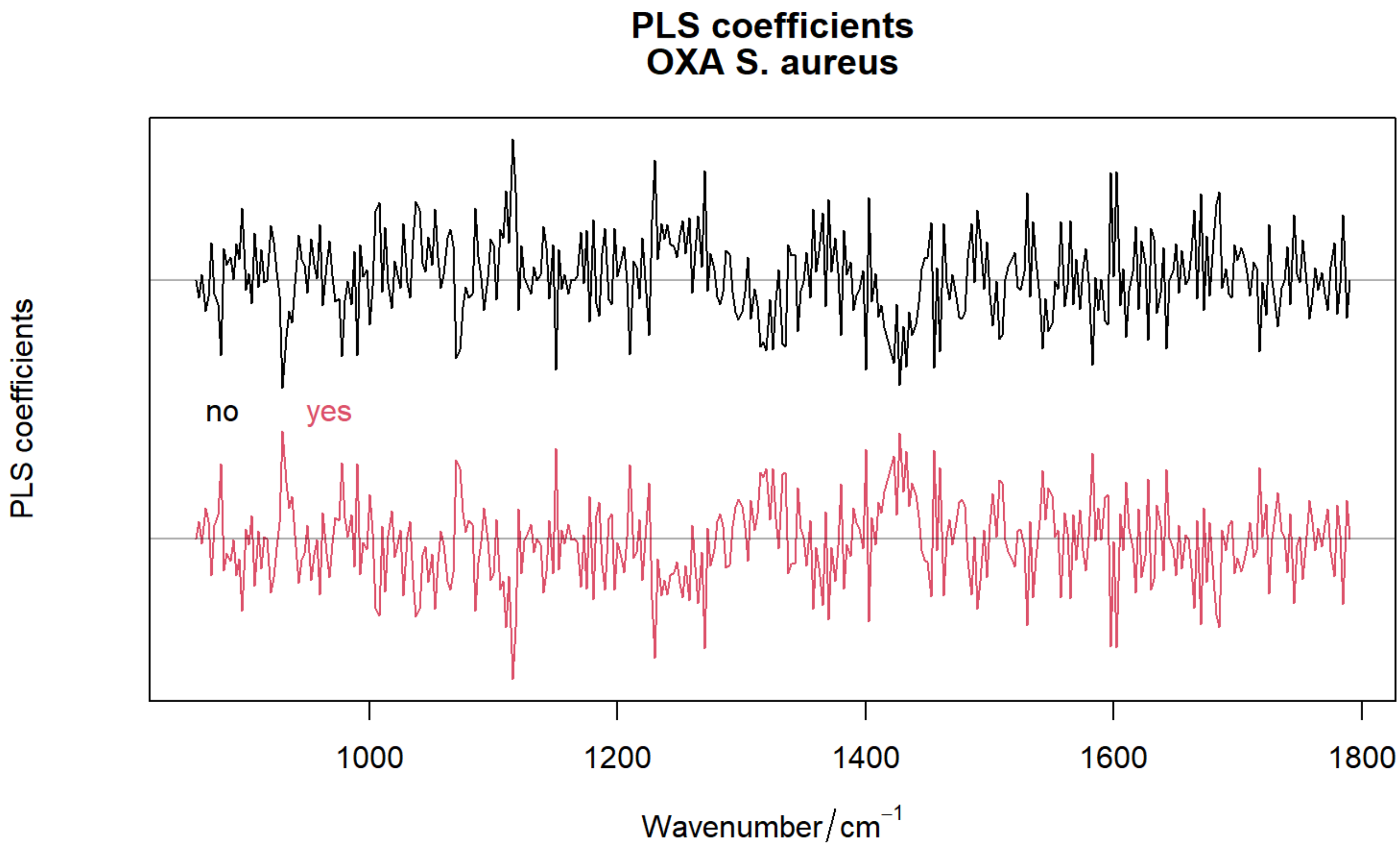
PLS coefficients
OXA S. aureus
PLS coefficients
no
yes
1000
1200
1400
1600
1800
Wavenumber/cm^{-1}

SI 8: Broth microdilution of bacteria isolated from artificial blood cultures

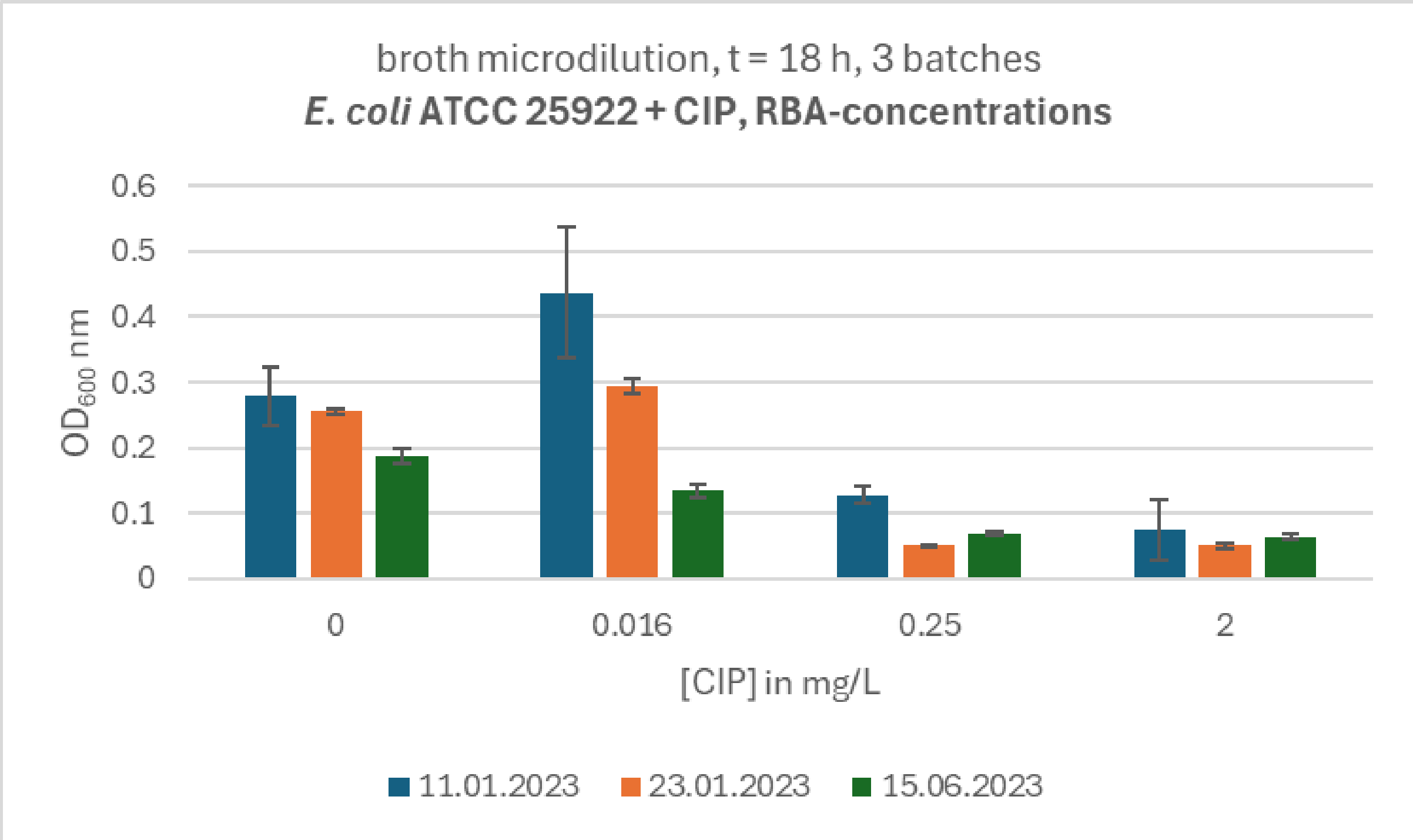

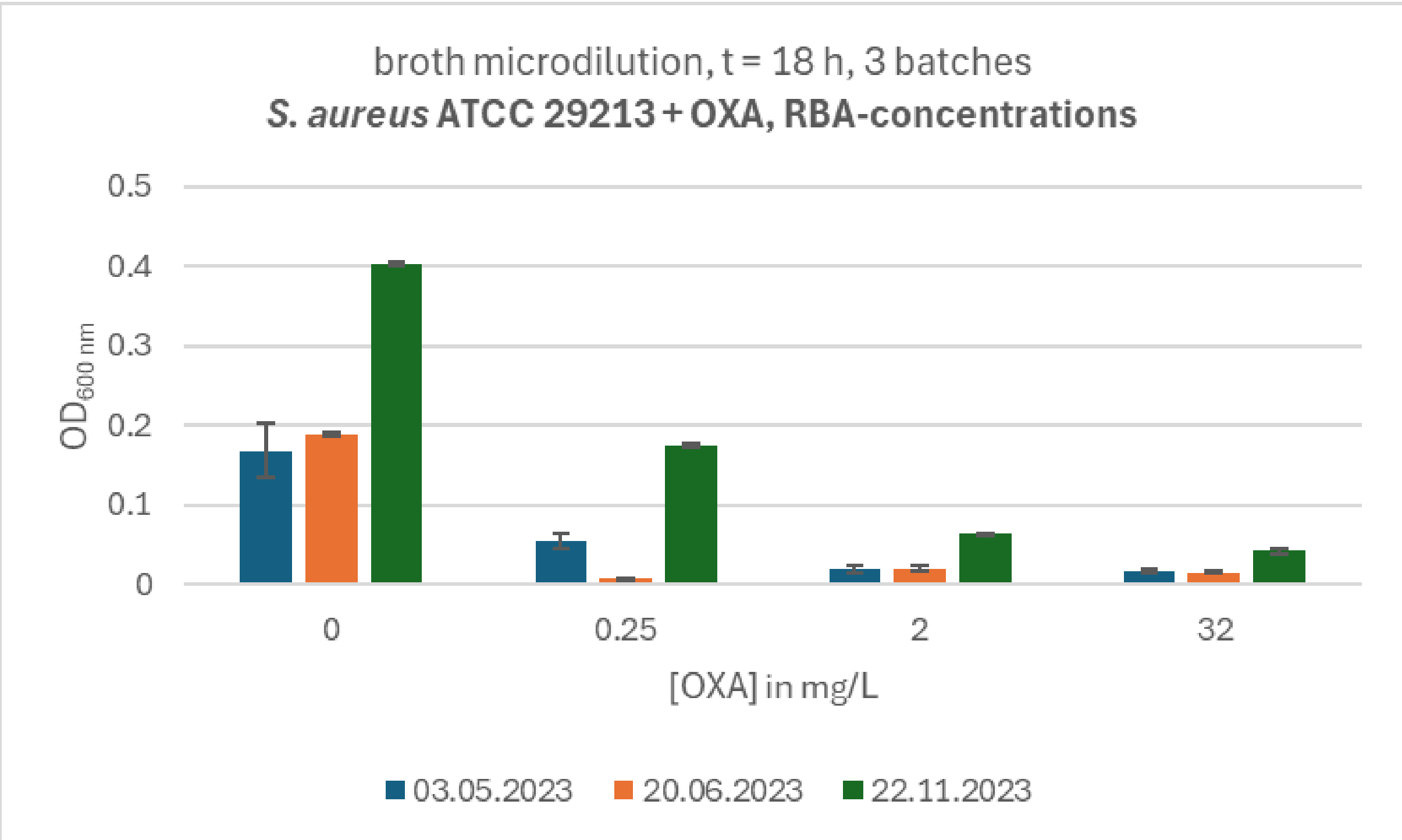

Infecto_Xplore_CV

```
## Culture: real blood culture
```

```
## Laser: 785
```

```
## Metadata table summary:
```

```
##
##                                                       Enterobacteriaceae
##   2023-01-11_MP_Ec+CIP_spectra_RG.zip                                64
##   2023-01-23_MP_Ec+CIP_spectra_RG.zip                                64
##   2023-05-03_MP_Sa+OXA_spectra_RG.zip                                 0
##   2023-06-15_MP_Ec+CIP_spectra_RG.zip                                64
##   2023-06-20_MP_Sa+OXA_532 BK missing_spectra_RG.zip                  0
##   2023-11-12_MP_Sa+OXA_spectra_RG.zip                                 0
##   e.c. 1.zip                                                         16
##   e.c. 4.zip                                                         16
##   e.c. 6.zip                                                         16
##   s.a. 1.zip                                                          0
##   s.a. 2.zip                                                          0
##   s.a. 3.zip                                                          0
##   <NA>                                                                0
##
##                                                       Staphylococcaceae <NA>
##   2023-01-11_MP_Ec+CIP_spectra_RG.zip                                0    4
##   2023-01-23_MP_Ec+CIP_spectra_RG.zip                                0    3
##   2023-05-03_MP_Sa+OXA_spectra_RG.zip                               64    2
##   2023-06-15_MP_Ec+CIP_spectra_RG.zip                                0    3
##   2023-06-20_MP_Sa+OXA_532 BK missing_spectra_RG.zip                48    4
##   2023-11-12_MP_Sa+OXA_spectra_RG.zip                               64    4
##   e.c. 1.zip                                                         0    0
##   e.c. 4.zip                                                         0    0
##   e.c. 6.zip                                                         0    0
##   s.a. 1.zip                                                        16    0
##   s.a. 2.zip                                                        16    0
##   s.a. 3.zip                                                        16    0
##   <NA>                                                               0   23
```

```
## Merged data summary:
```

```
##
##                Enterobacteriaceae Staphylococcaceae <NA>
##   e.c. 1.zip                   24                 0    0
##   e.c. 4.zip                   24                 0    0
##   e.c. 6.zip                   24                 0    0
##   s.a. 1.zip                    0                24    0
##   s.a. 2.zip                    0                24    0
##   s.a. 3.zip                    0                24    0
##   <NA>                          0                 0    0
```

Cross-validate patient data

```
## real blood culture 785 e.c. 1
```

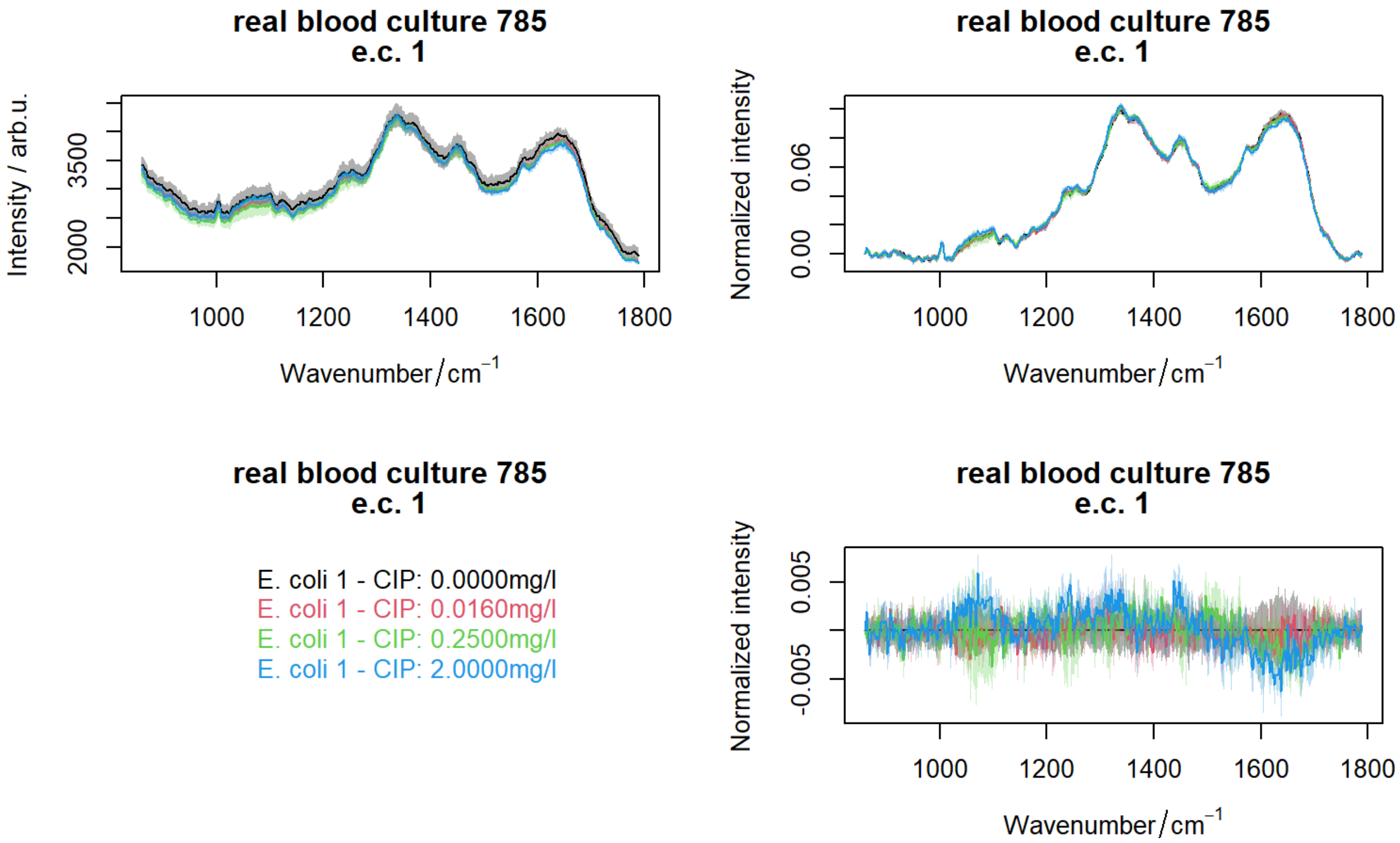


```
## real blood culture 785 e.c. 4
```

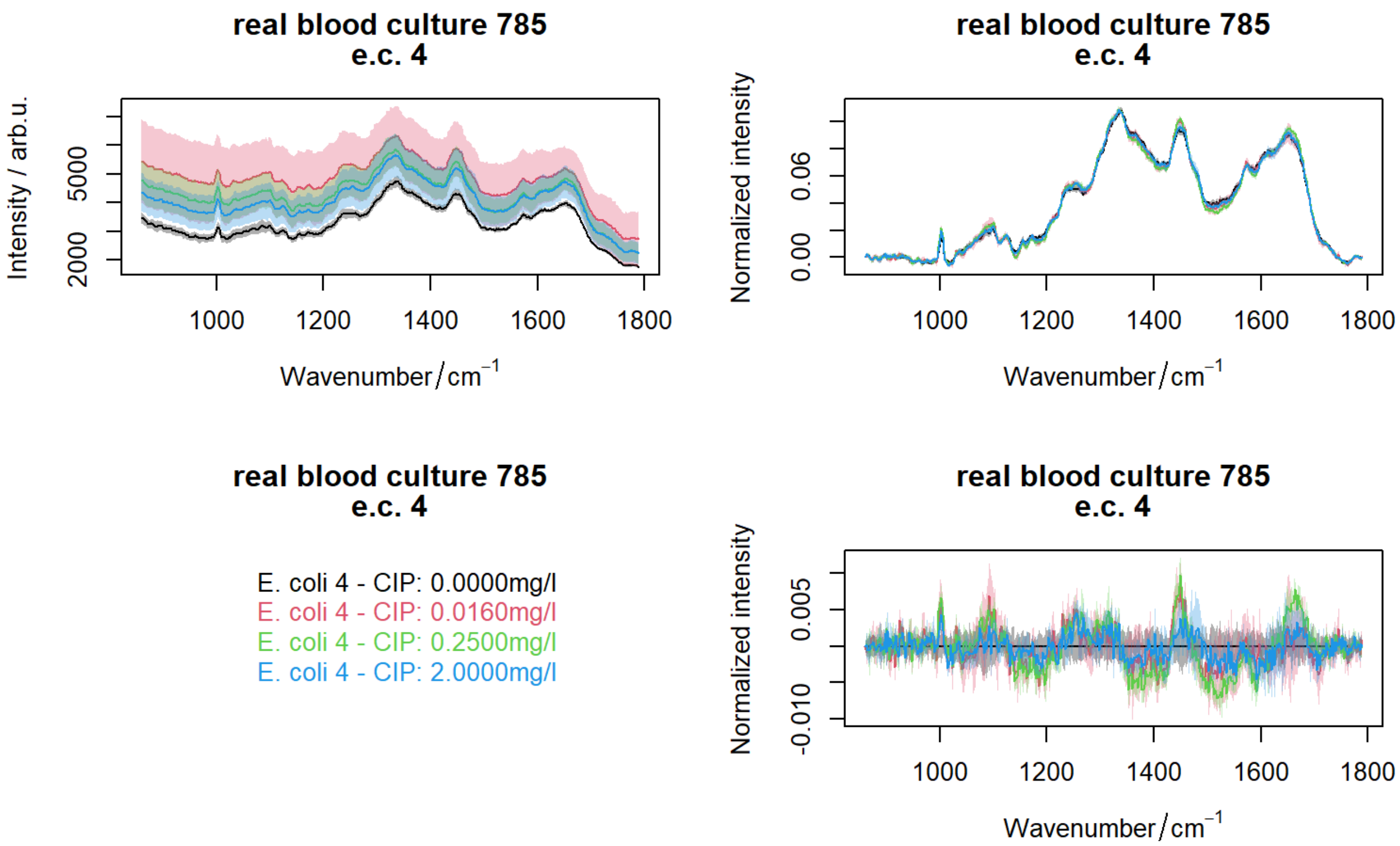

```
## real blood culture 785 e.c. 6
```

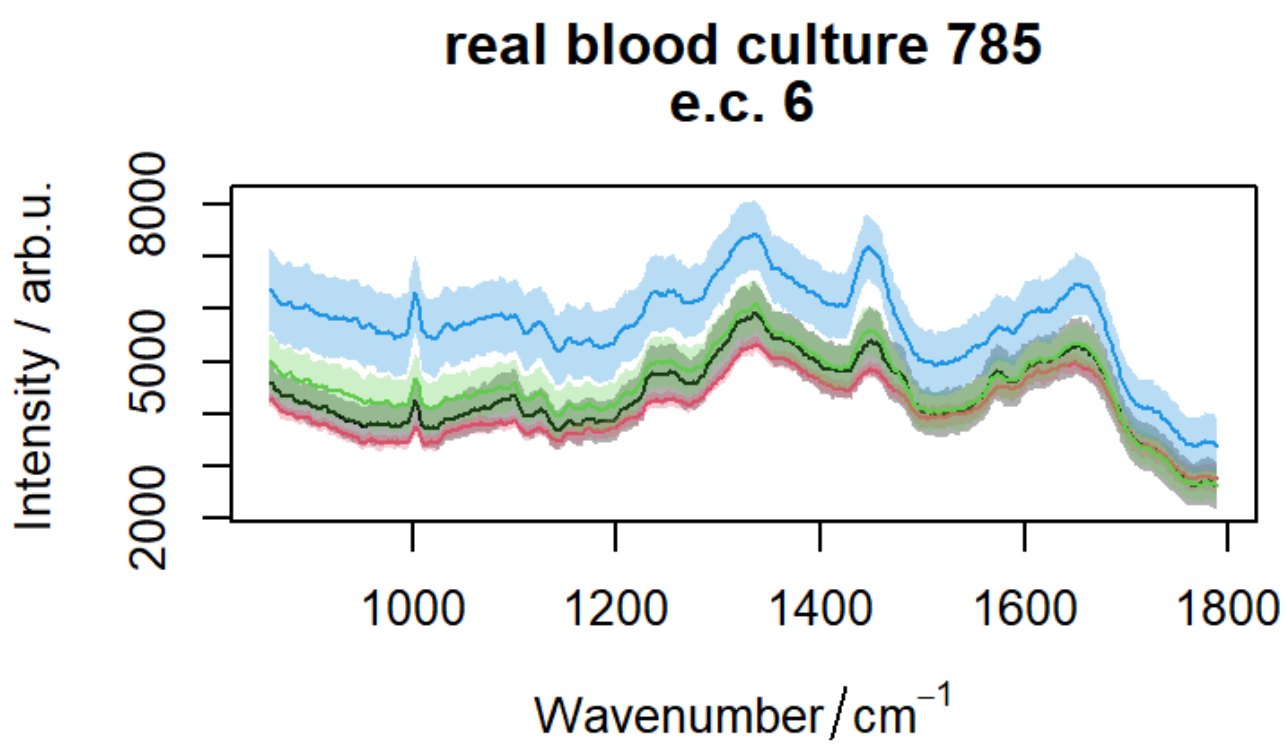

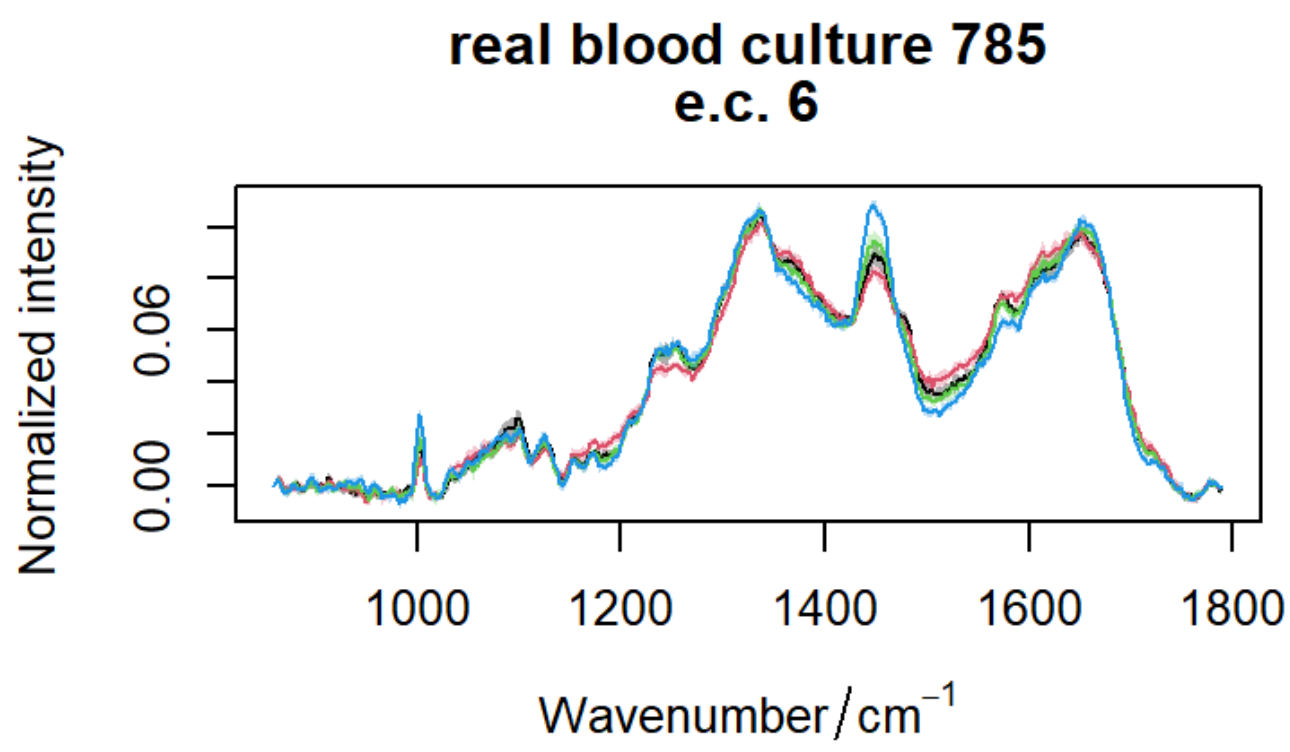

real blood culture 785
e.c. 6

E. coli 6 - CIP: 0.0000mg/l
E. coli 6 - CIP: 0.0160mg/l
E. coli 6 - CIP: 0.2500mg/l
E. coli 6 - CIP: 2.0000mg/l

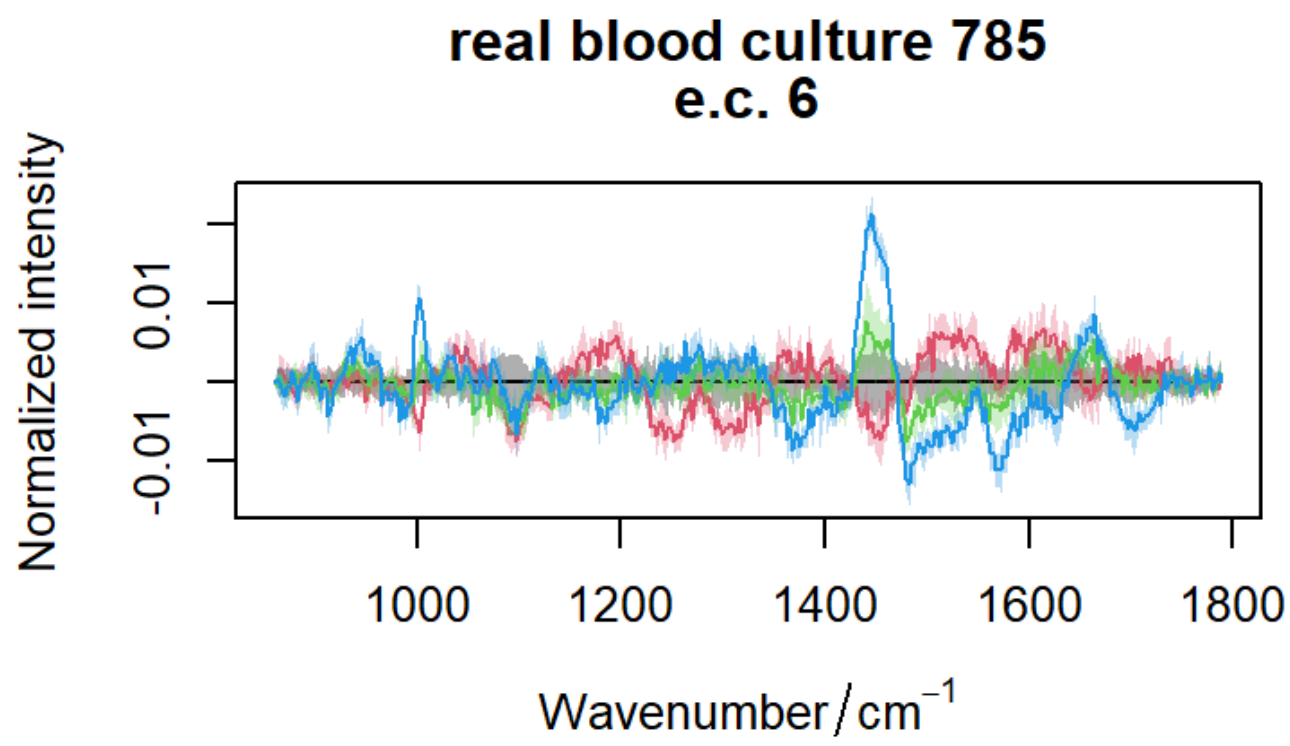


```
## real blood culture 785 s.a. 1
```

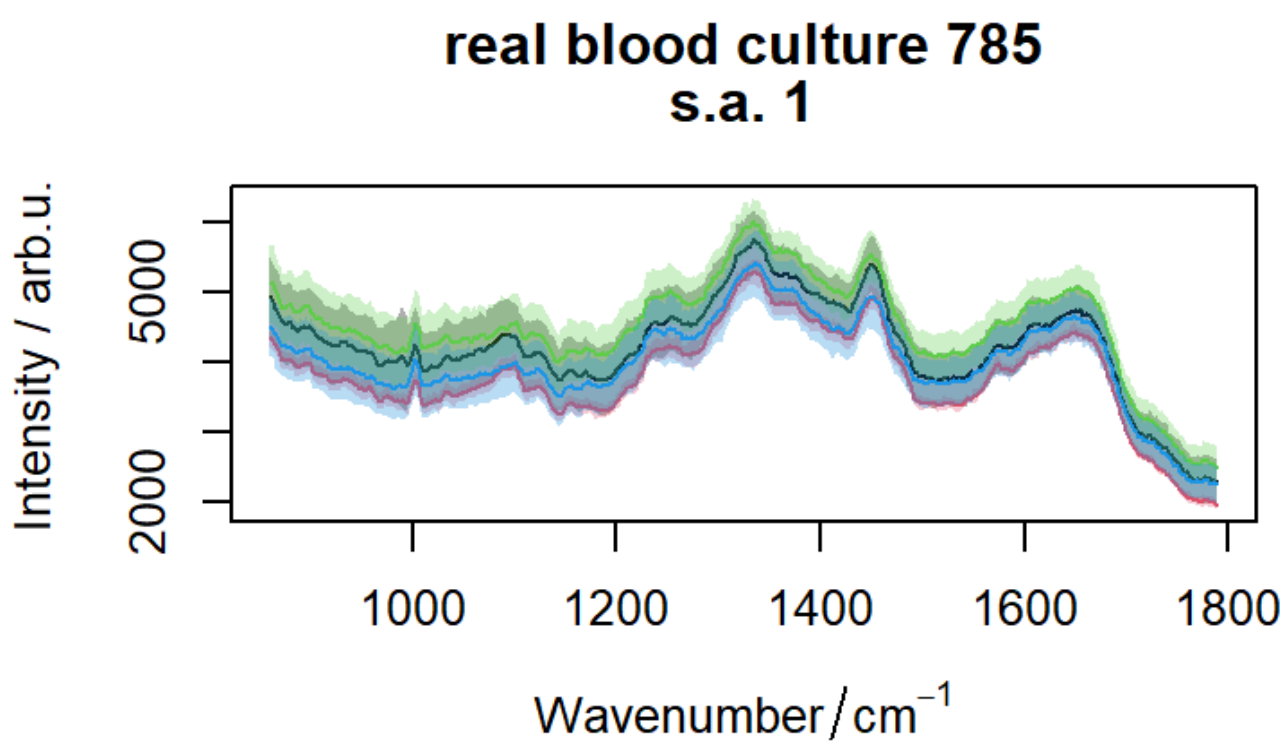

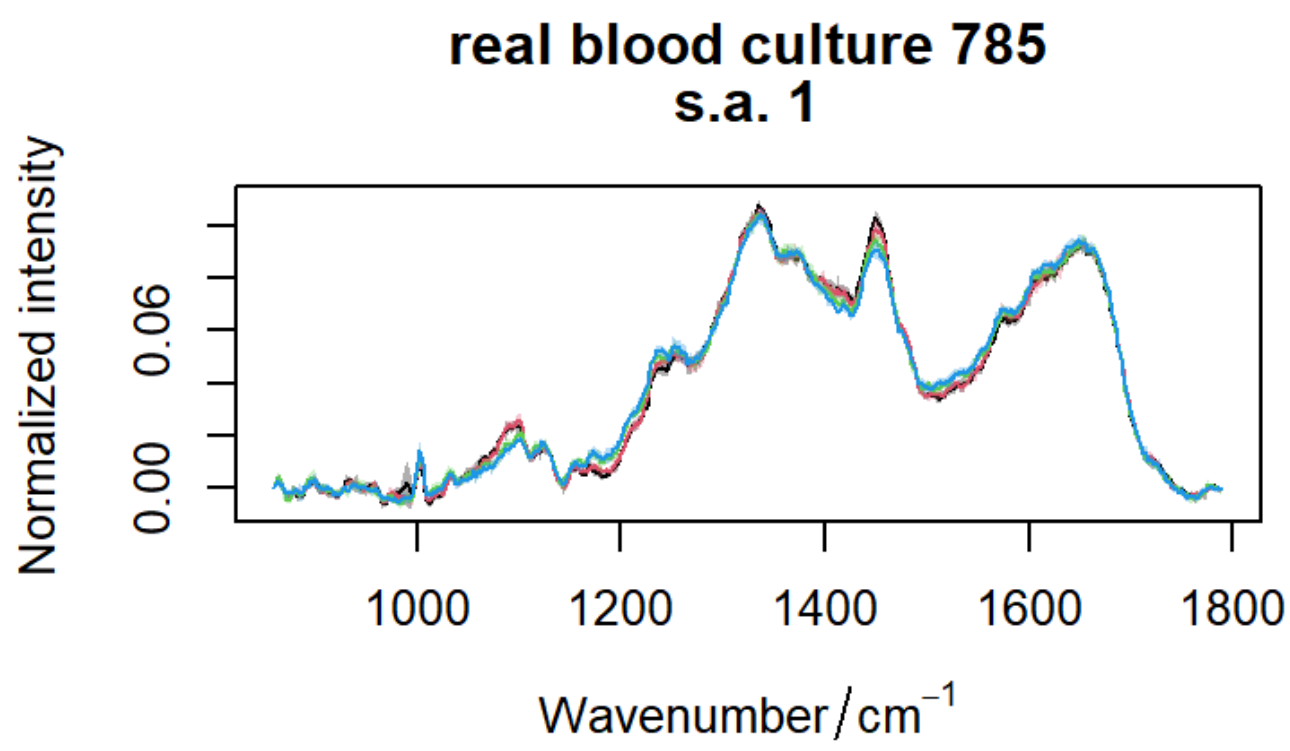

real blood culture 785
s.a. 1

S. aureus 1 - OXA: 0.0000mg/l
S. aureus 1 - OXA: 0.2500mg/l
S. aureus 1 - OXA: 2.0000mg/l
S. aureus 1 - OXA: 32.0000mg/l

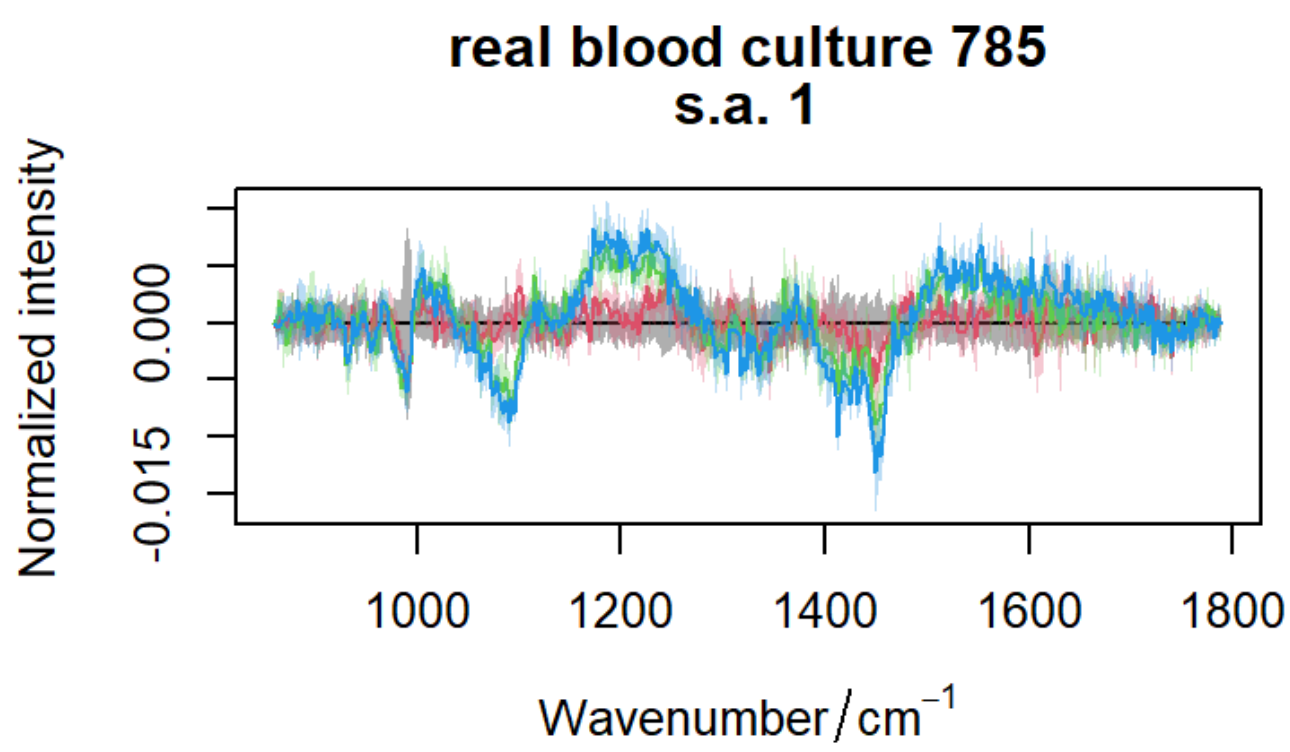

```
## real blood culture 785 s.a. 2
```

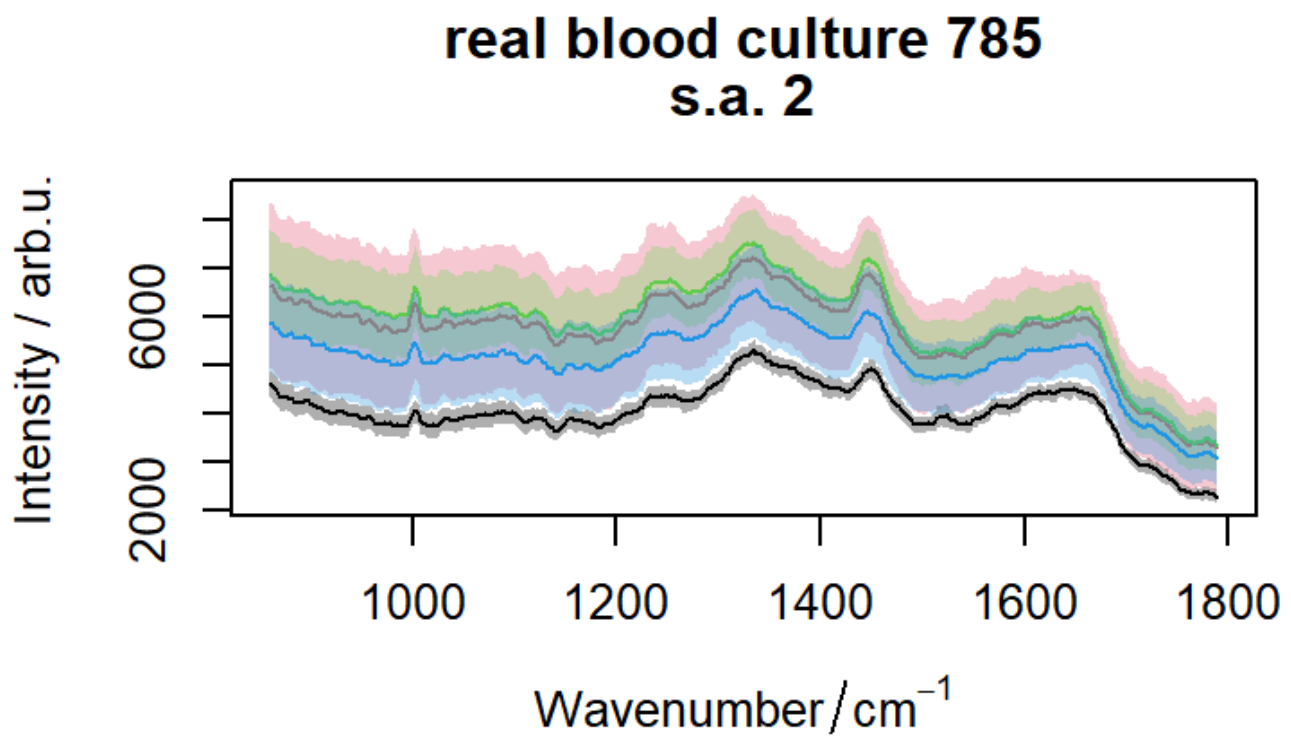

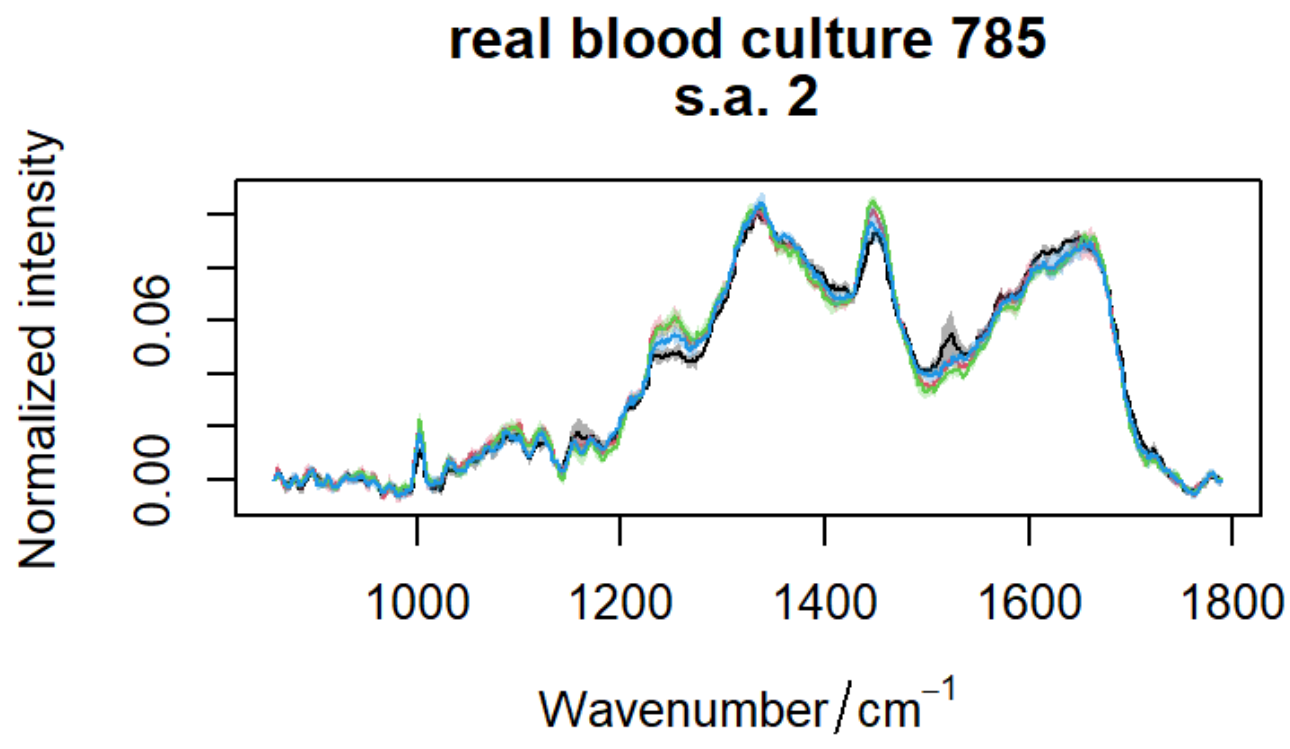

real blood culture 785
s.a. 2

S. aureus 2 - OXA: 0.0000mg/l
S. aureus 2 - OXA: 0.2500mg/l
S. aureus 2 - OXA: 2.0000mg/l
S. aureus 2 - OXA: 32.0000mg/l

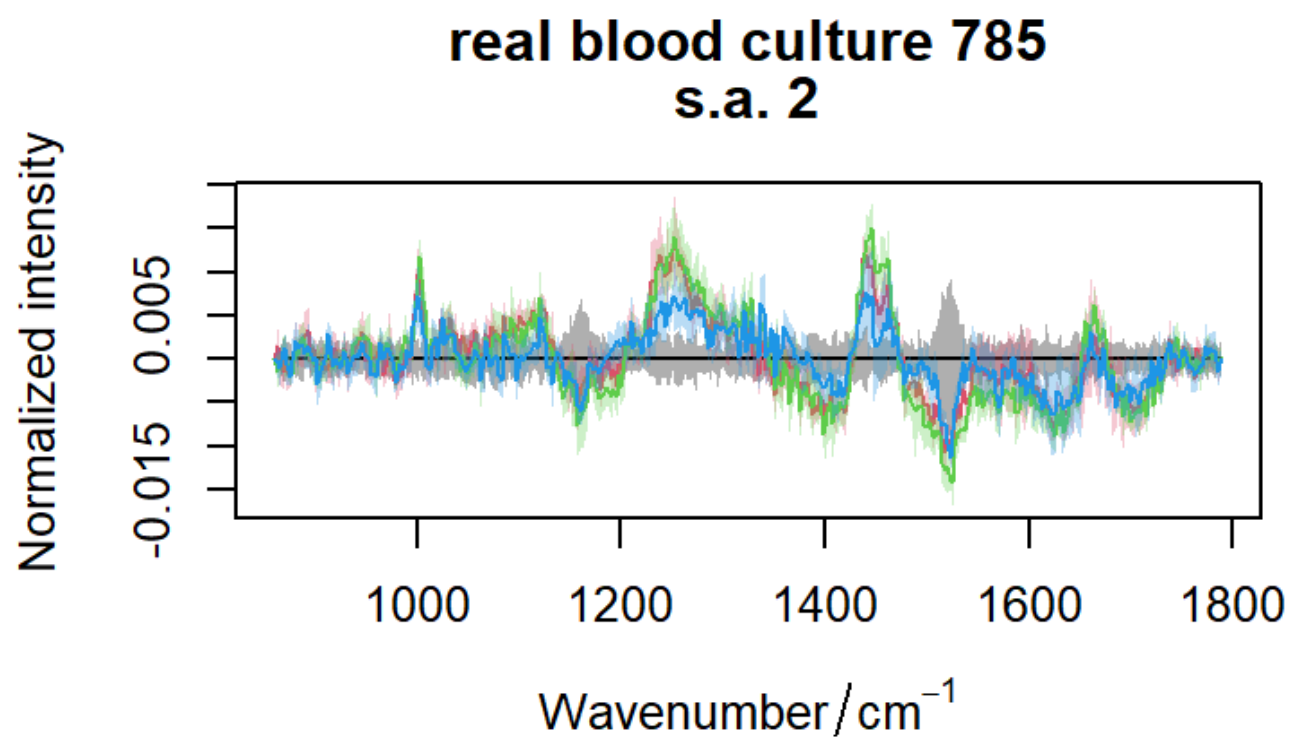


```
## real blood culture 785 s.a. 3
```

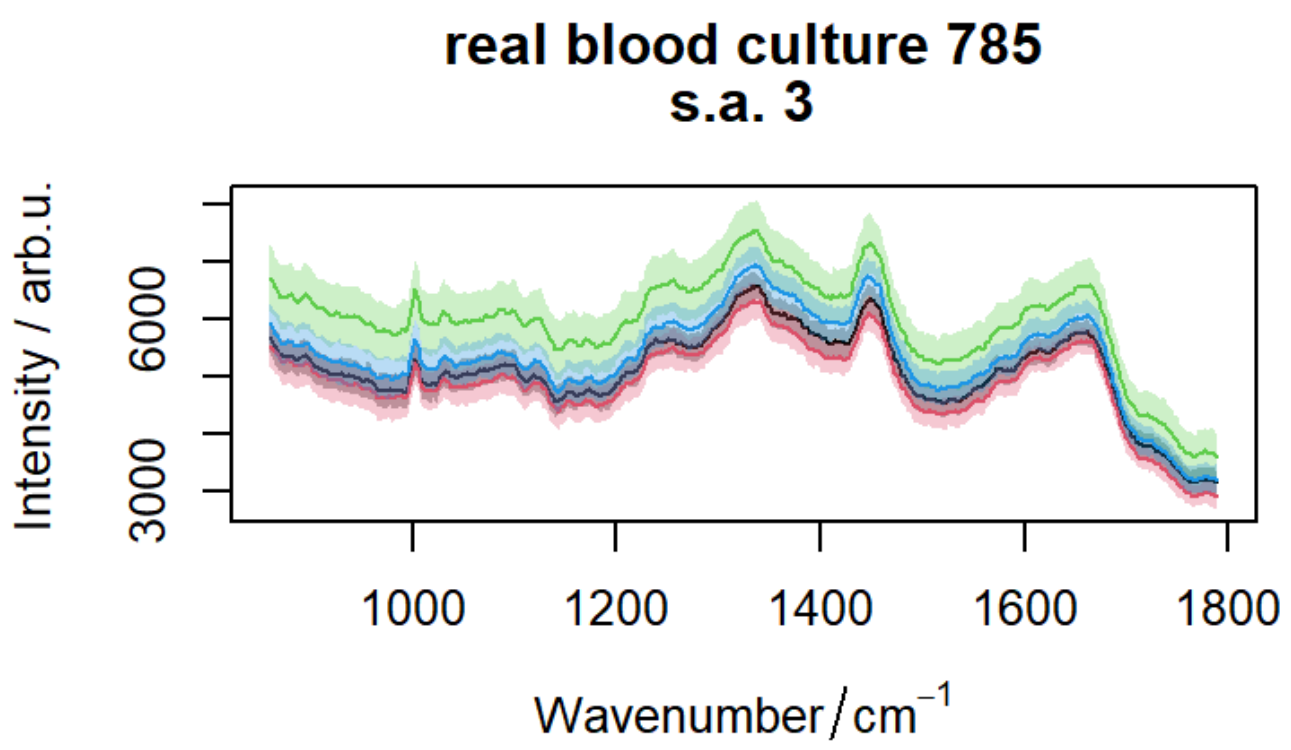

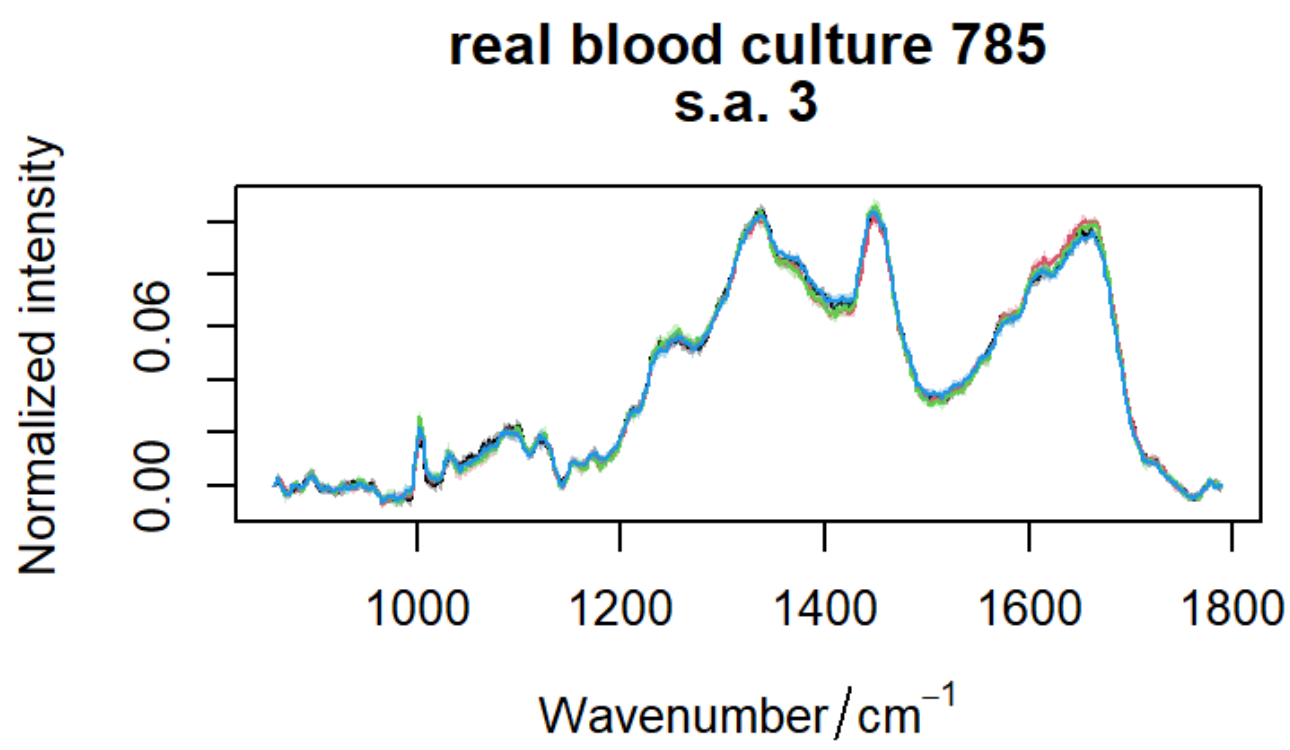

real blood culture 785
s.a. 3

S. aureus 3 - OXA: 0.0000mg/l
S. aureus 3 - OXA: 0.2500mg/l
S. aureus 3 - OXA: 2.0000mg/l
S. aureus 3 - OXA: 32.0000mg/l

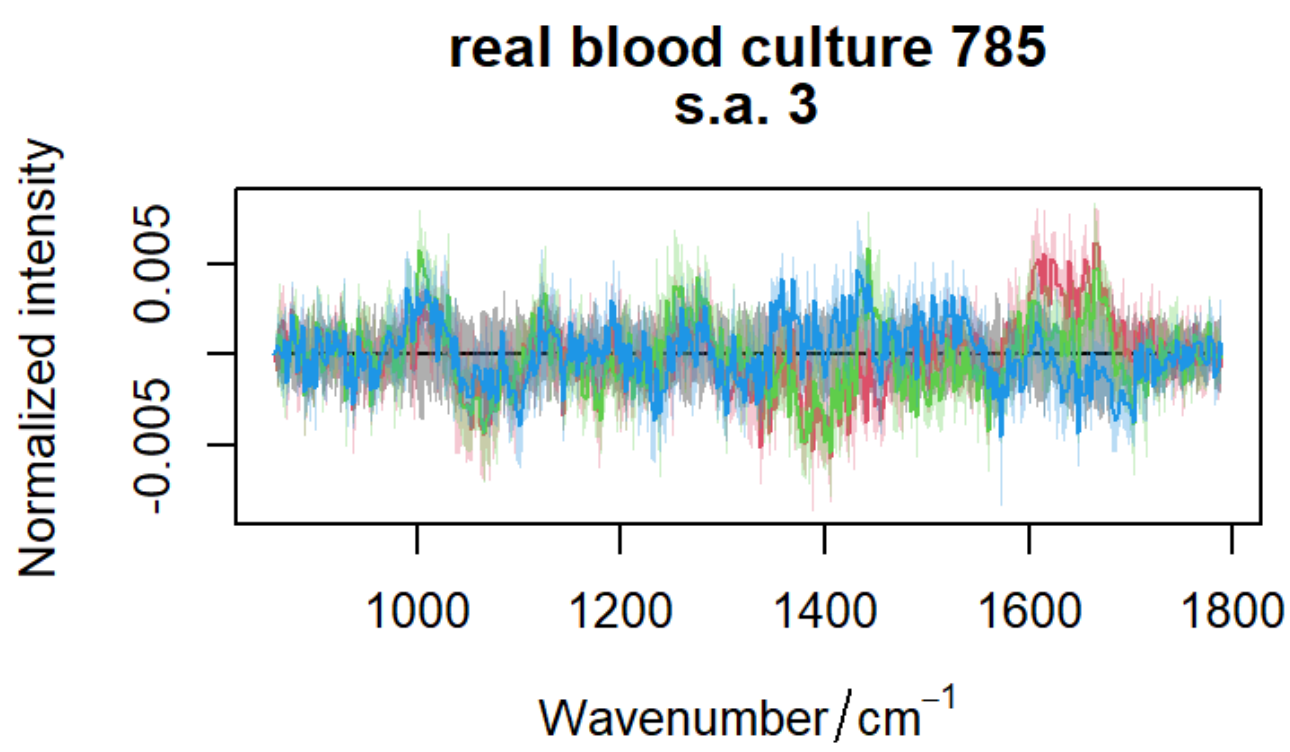

```
##
## growth expected:
```

```
##
##              no yes
##    CIP 0       0  18
##    CIP 0.016   0  18
##    CIP 0.25    6  12
##    CIP 2      12   6
##    OXA 0       0  18
##    OXA 0.25   12   6
##    OXA 2      18   0
##    OXA 32     18   0
```

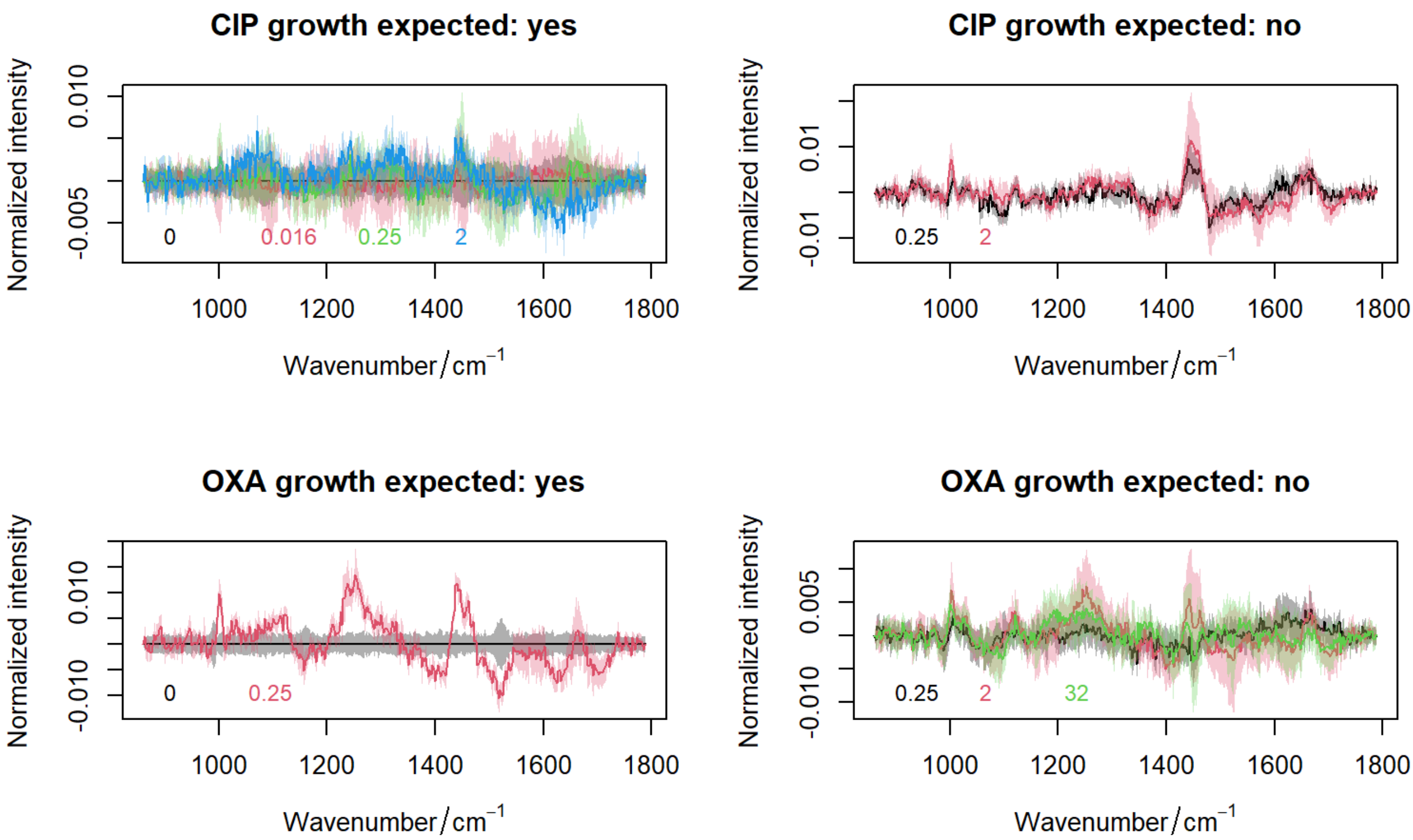

SI 9

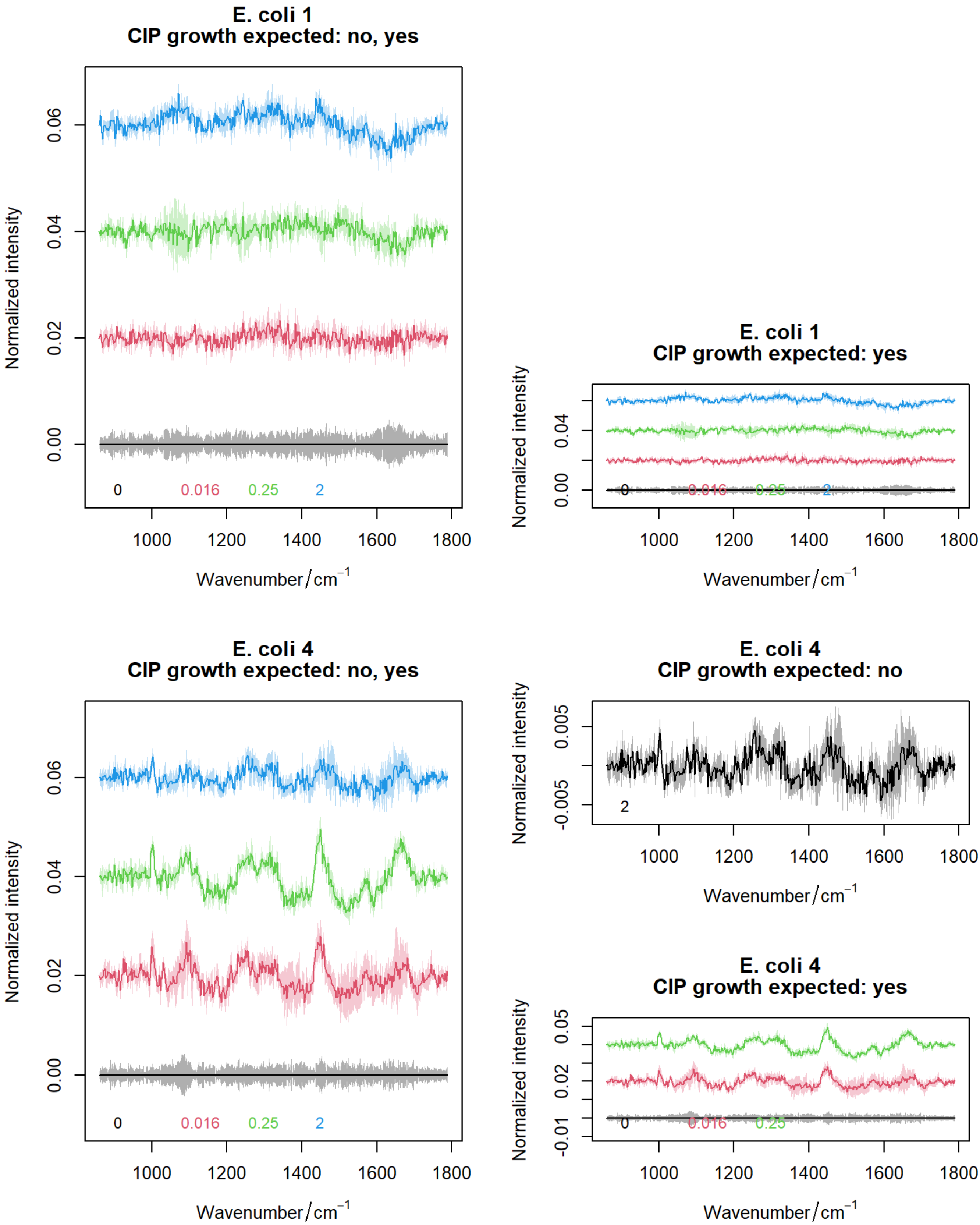

E. coli 1
CIP growth expected: no, yes
Normalized intensity
Wavenumber / cm^{-1}
0
0.016
0.25
2
E. coli 1
CIP growth expected: yes
E. coli 4
CIP growth expected: no, yes
E. coli 4
CIP growth expected: no
E. coli 4
CIP growth expected: yes

SI 9

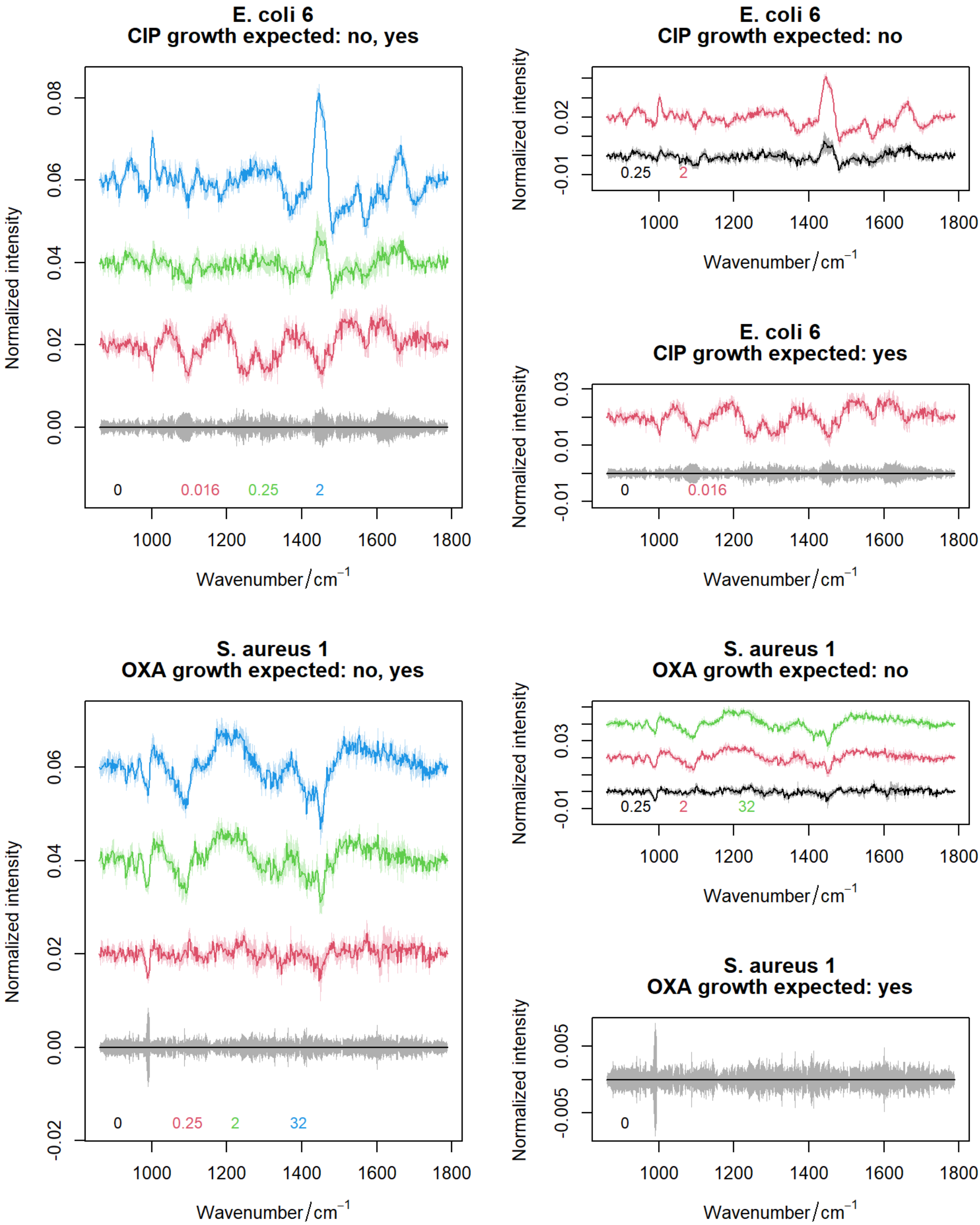

E. coli 6
CIP growth expected: no, yes
Normalized intensity
0.00
0.02
0.04
0.06
0.08
0
0.016
0.25
2
1000
1200
1400
1600
1800
Wavenumber / cm^{-1}
E. coli 6
CIP growth expected: no
-0.01
0.02
0.25
2
E. coli 6
CIP growth expected: yes
-0.01
0.01
0.03
0
0.016
S. aureus 1
OXA growth expected: no, yes
-0.02
0.00
0.02
0.04
0.06
0
0.25
2
32
S. aureus 1
OXA growth expected: no
-0.01
0.03
0.25
2
32
S. aureus 1
OXA growth expected: yes
-0.005
0.005
0

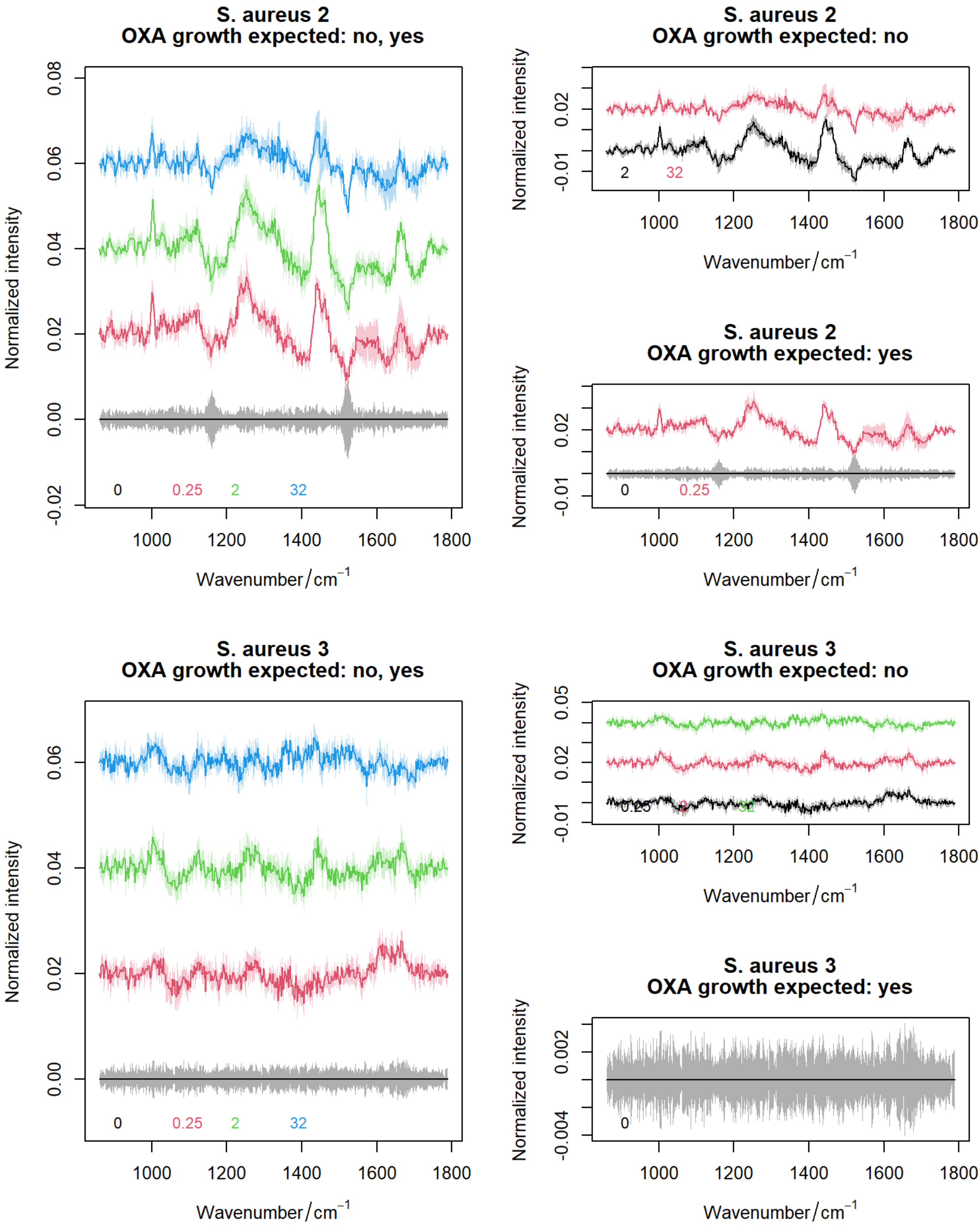

S. aureus 2
OXA growth expected: no, yes
Normalized intensity
Wavenumber / cm^{-1}
0
0.25
2
32
S. aureus 2
OXA growth expected: no
2
32
S. aureus 2
OXA growth expected: yes
0
0.25
S. aureus 3
OXA growth expected: no, yes
0
0.25
2
32
S. aureus 3
OXA growth expected: no
S. aureus 3
OXA growth expected: yes
0

```
##
## ===============
##  CIP E. coli (all)
##
##      pred
## true  no yes       acc sensitivity specificity
##   no  10   8 0.8888889   0.5555556   1.0000000
##   yes  0  54 0.8888889   1.0000000   0.5555556
##
## Balanced accuracy: 77.8 %
##
##
## ===============
##  CIP E. coli 1
##
##      pred
## true  no yes acc sensitivity specificity
##   no   0   0   1         NaN           1
##   yes  0  24   1           1         NaN
##
## Balanced accuracy: NaN %
##
##
## ===============
##  CIP E. coli 4
##
##      pred
## true  no yes  acc sensitivity specificity
##   no   0   6 0.75           0           1
##   yes  0  18 0.75           1           0
##
## Balanced accuracy: 50 %
##
##
## ===============
##  CIP E. coli 6
##
##      pred
## true  no yes       acc sensitivity specificity
##   no  10   2 0.9166667   0.8333333   1.0000000
##   yes  0  12 0.9166667   1.0000000   0.8333333
##
## Balanced accuracy: 91.7 %
```

SI 9

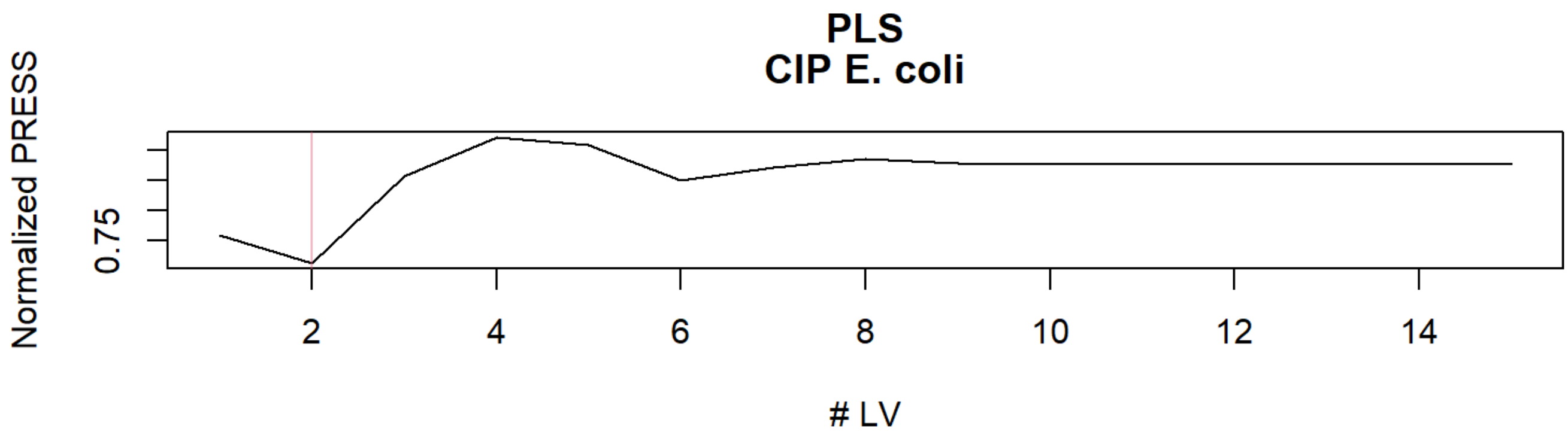
PLS
CIP E. coli
Normalized PRESS
0.75
2
4
6
8
10
12
14
LV

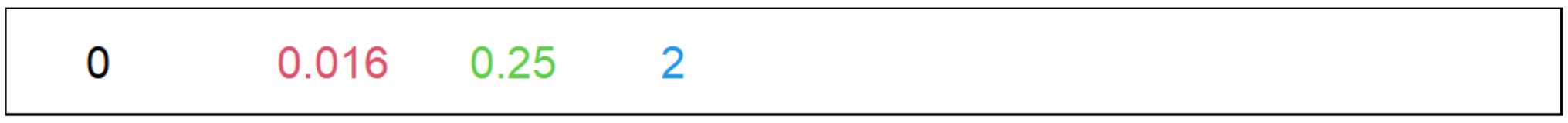
0
0.016
0.25
2

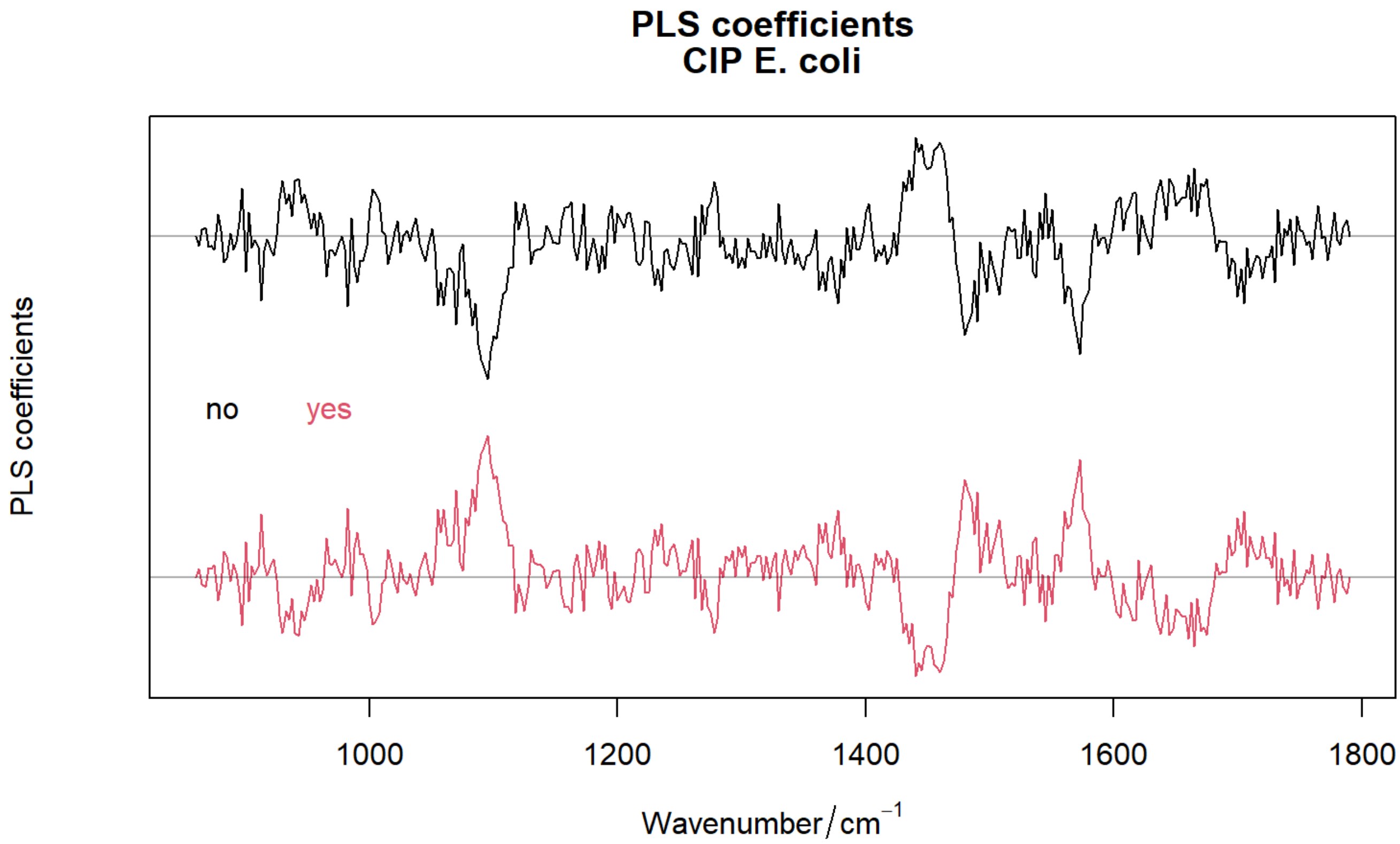
PLS coefficients
CIP E. coli
PLS coefficients
no
yes
1000
1200
1400
1600
1800
Wavenumber / cm⁻¹

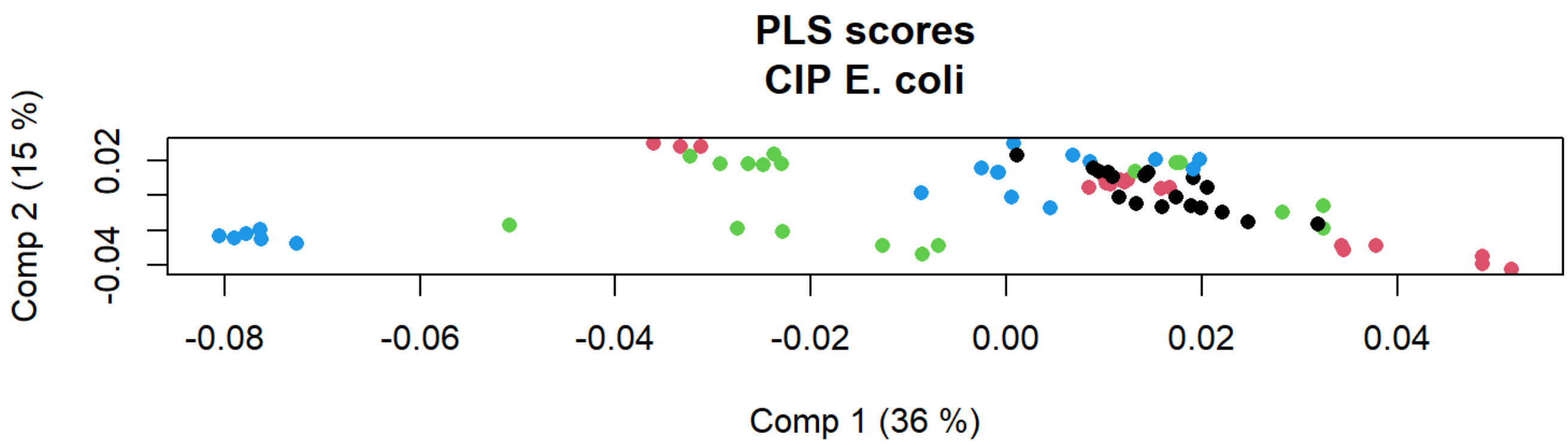
PLS scores
CIP E. coli
Comp 2 (15 %)
0.02
-0.04
-0.08
-0.06
-0.04
-0.02
0.00
0.02
0.04
Comp 1 (36 %)

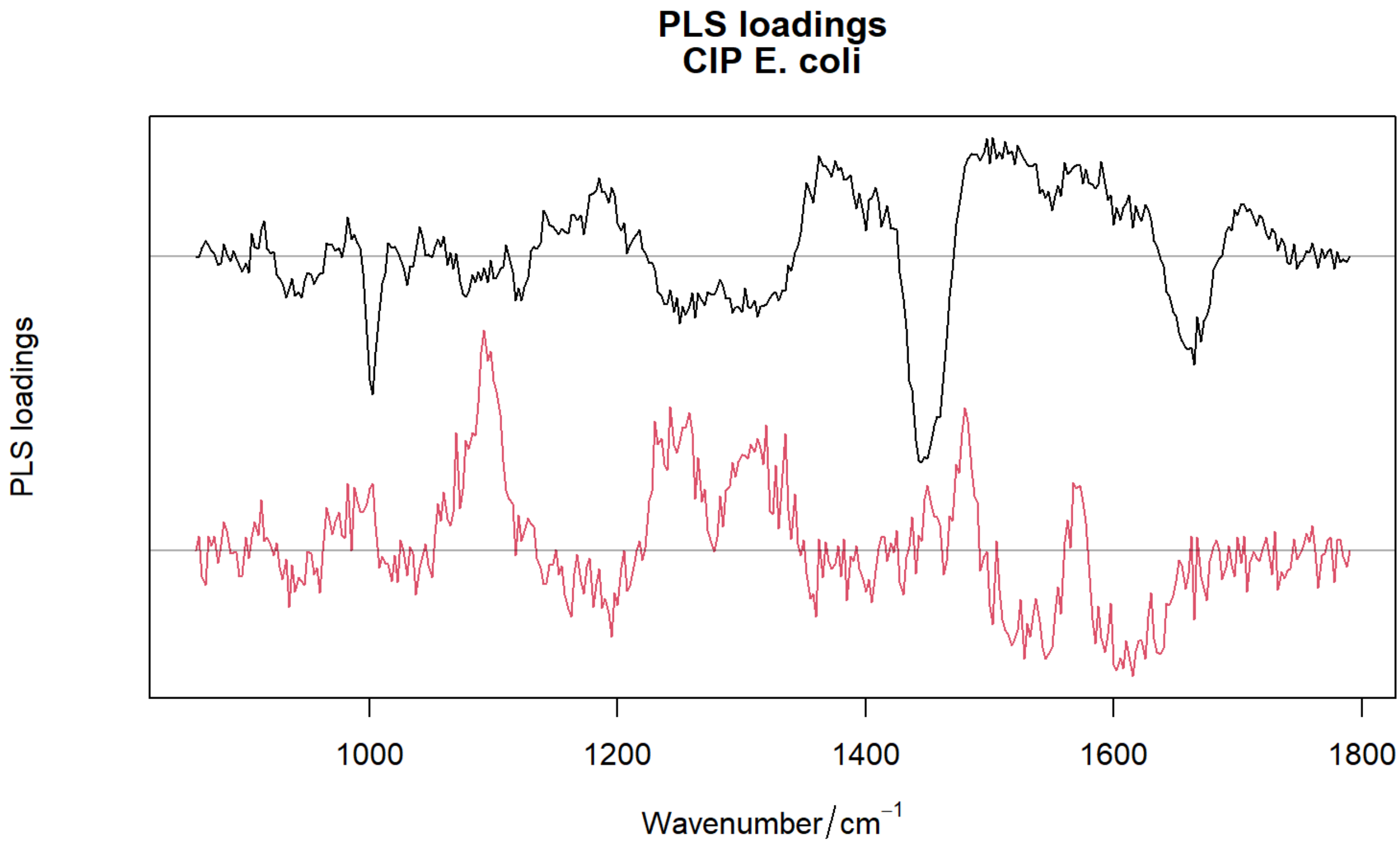
PLS loadings
CIP E. coli
PLS loadings
1000
1200
1400
1600
1800
Wavenumber / cm^{-1}

```
##
## ===============
##  OXA S. aureus (all)
##
##      pred
## true  no yes  acc sensitivity specificity
##   no  36  12 0.75        0.75        0.75
##   yes  6  18 0.75        0.75        0.75
##
## Balanced accuracy: 75 %
##
##
## ===============
##  OXA S. aureus 1
##
##      pred
## true  no yes  acc sensitivity specificity
##   no  12   6 0.75   0.6666667   1.0000000
##   yes  0   6 0.75   1.0000000   0.6666667
##
## Balanced accuracy: 83.3 %
##
##
## ===============
##  OXA S. aureus 2
##
##      pred
## true  no yes acc sensitivity specificity
##   no   6   6 0.5         0.5         0.5
##   yes  6   6 0.5         0.5         0.5
##
## Balanced accuracy: 50 %
##
##
## ===============
##  OXA S. aureus 3
##
##      pred
## true  no yes acc sensitivity specificity
##   no  18   0   1           1           1
##   yes  0   6   1           1           1
##
## Balanced accuracy: 100 %
```

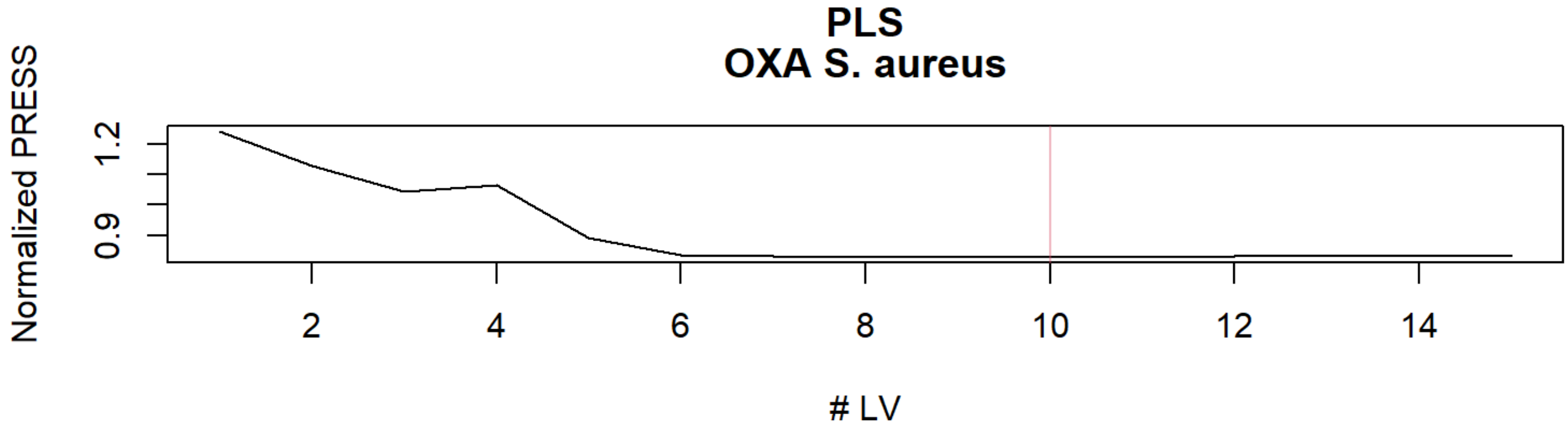
PLS
OXA S. aureus
Normalized PRESS
0.9
1.2
2
4
6
8
10
12
14
LV

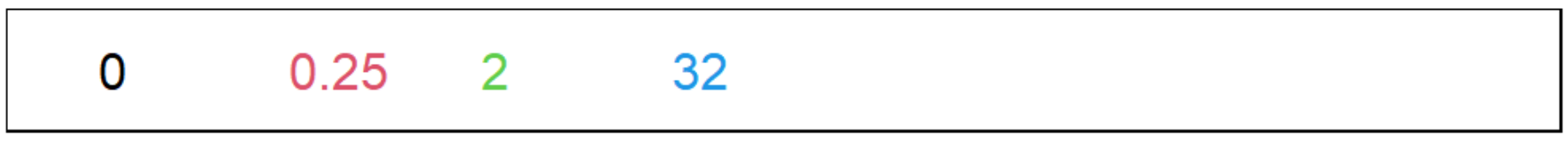
0
0.25
2
32

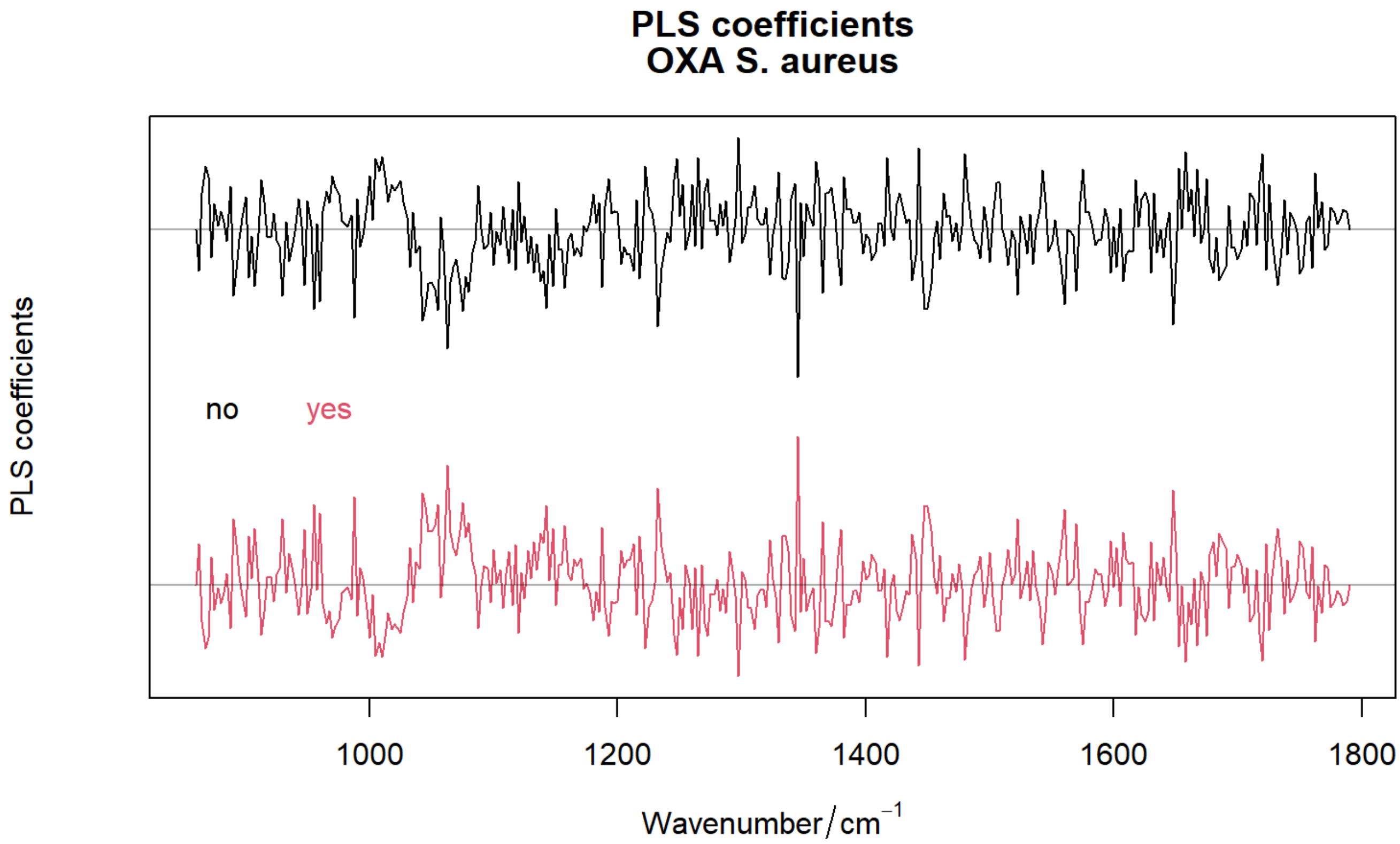
PLS coefficients
OXA S. aureus
PLS coefficients
no
yes
1000
1200
1400
1600
1800
Wavenumber / cm^{-1}

PLS scores
OXA S. aureus

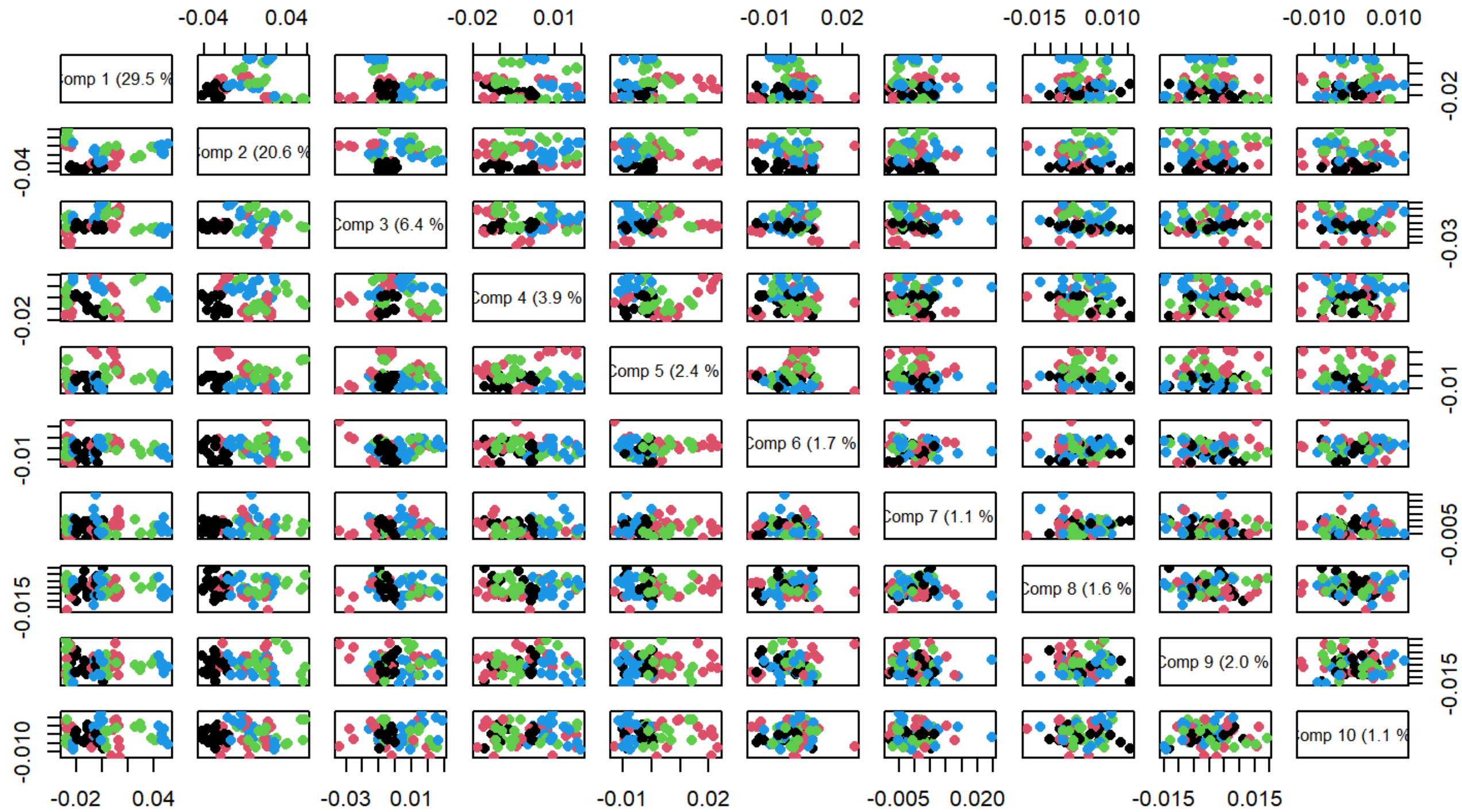

PLS loadings
OXA S. aureus

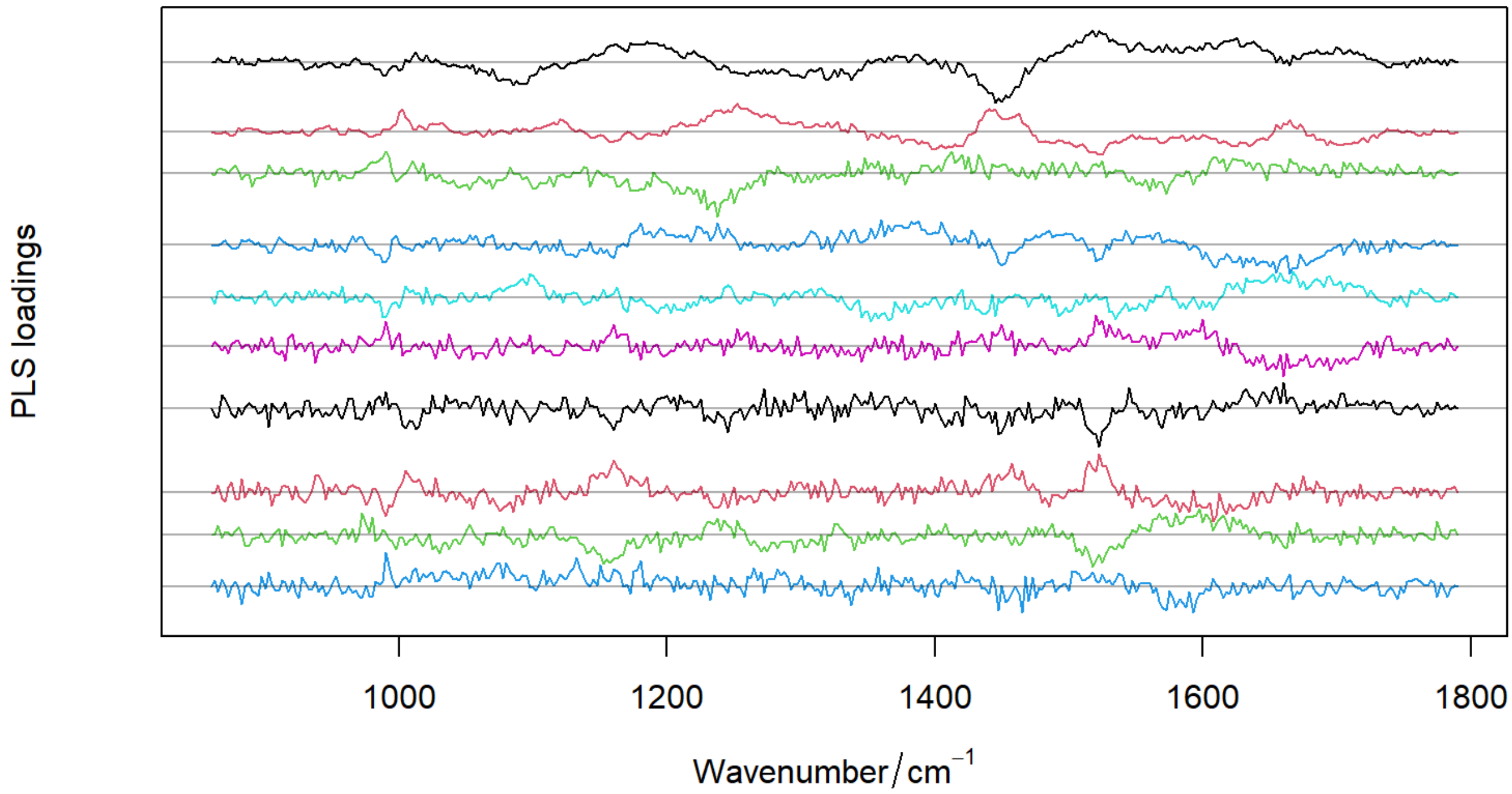

SI 9: Broth microdilution of bacteria isolated from patient blood cultures

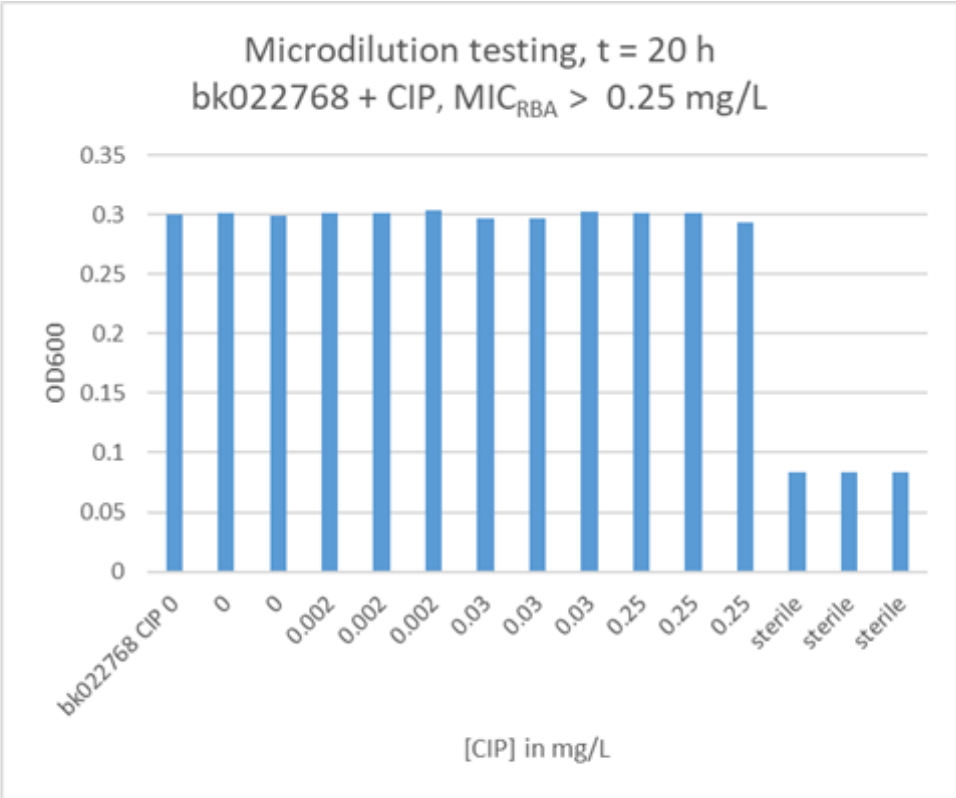

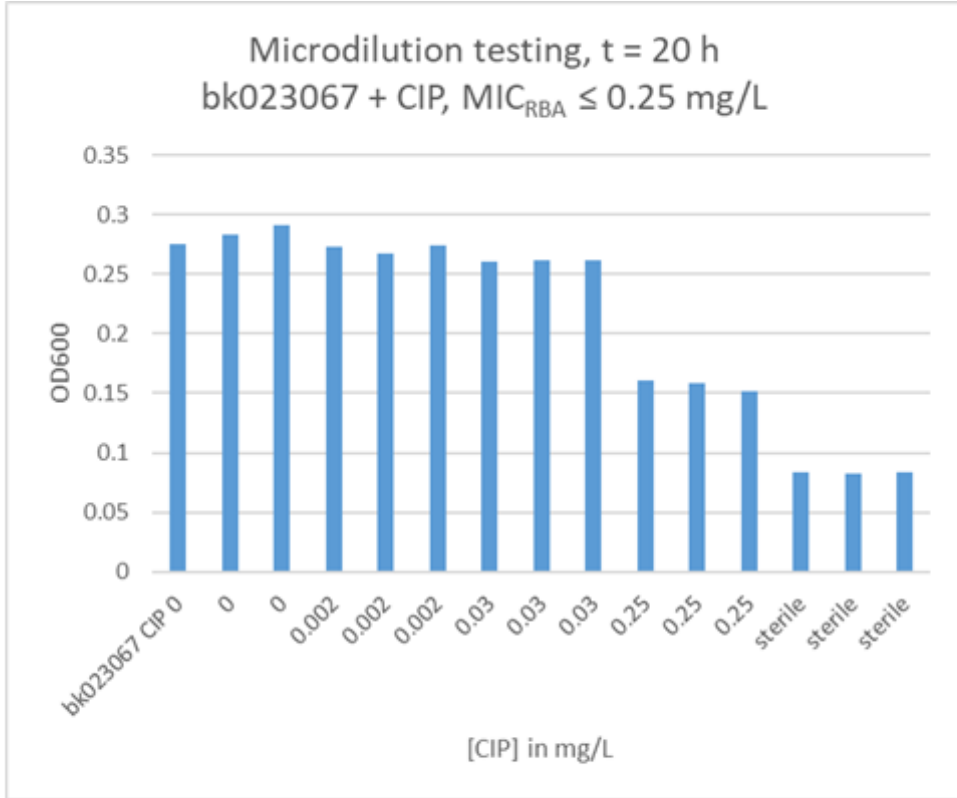

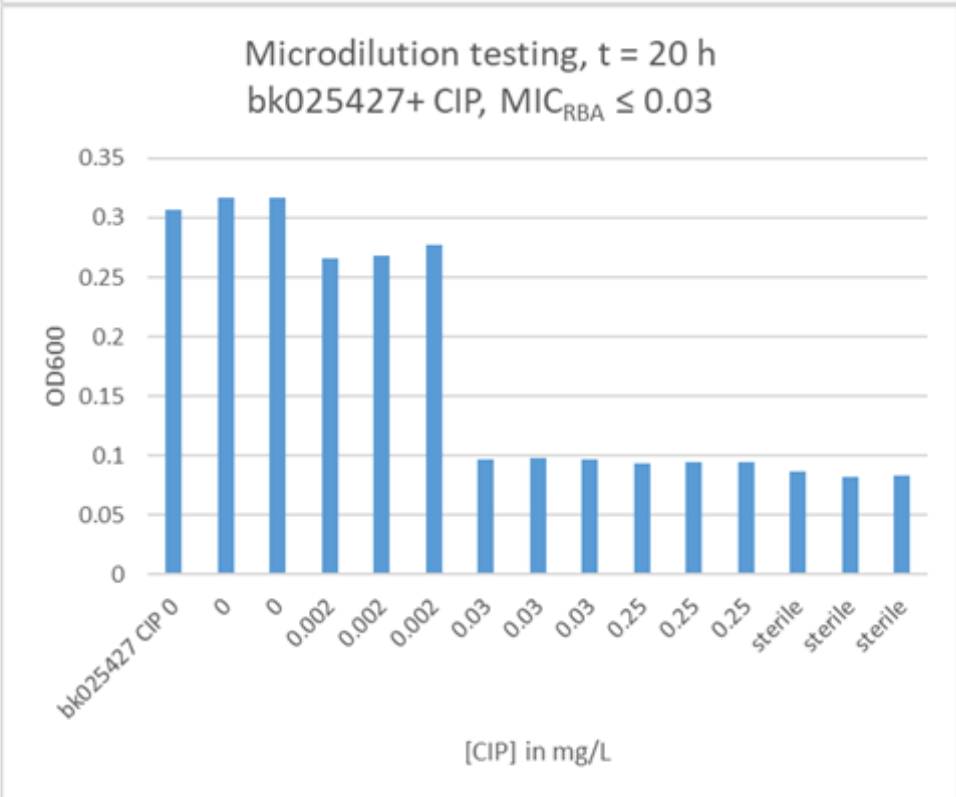

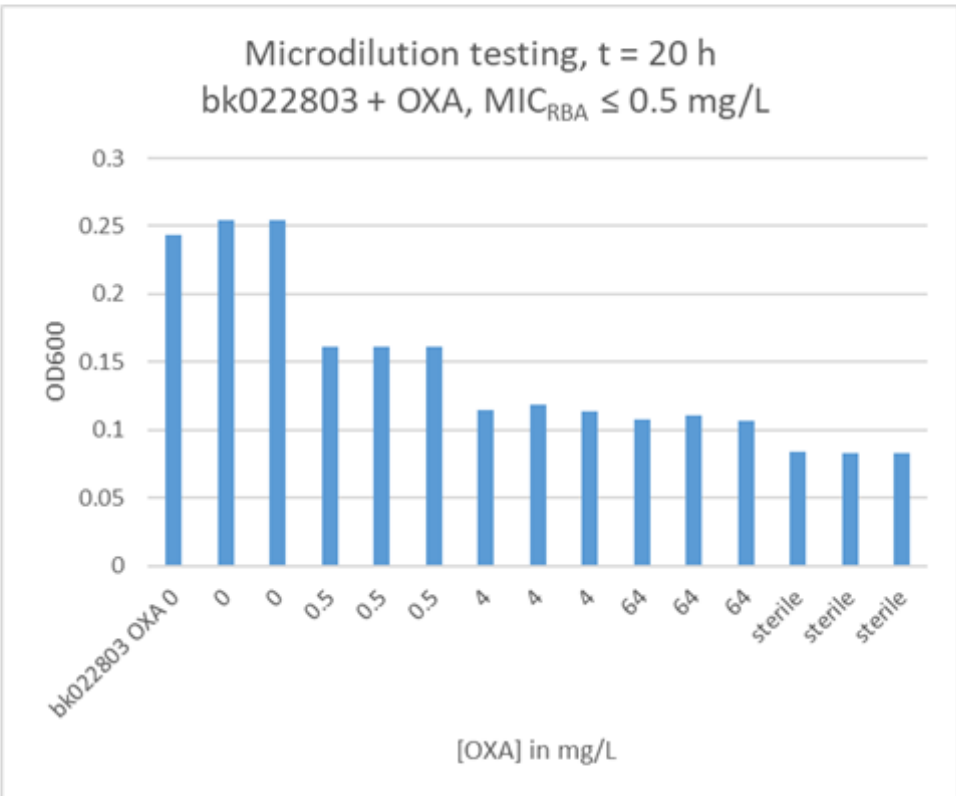

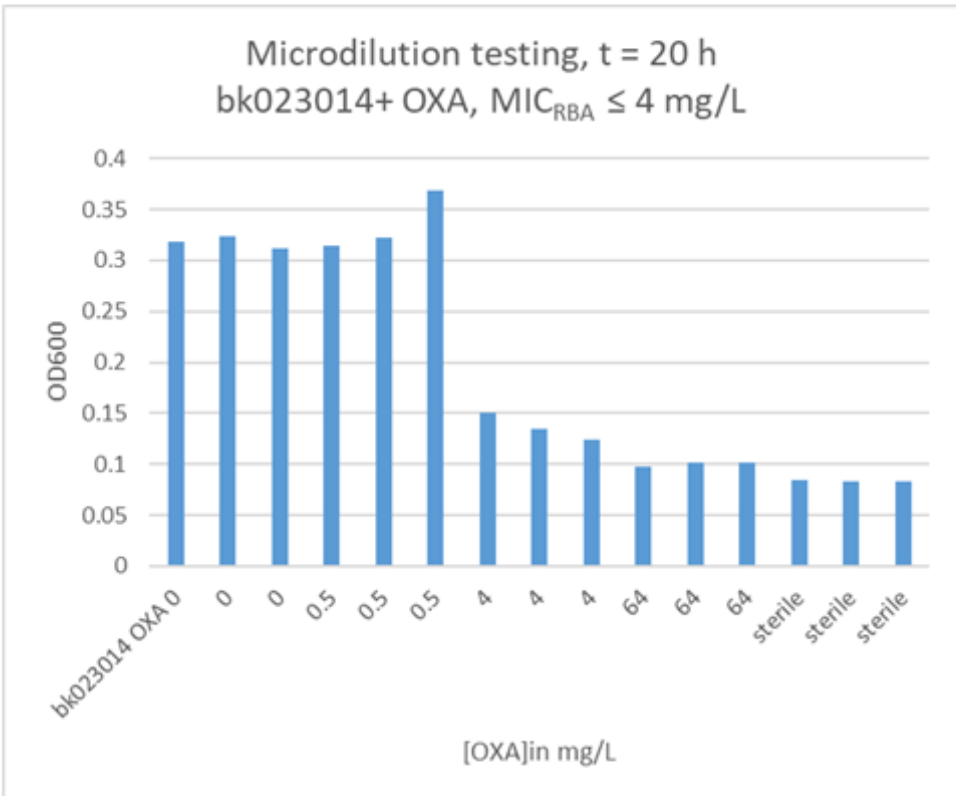

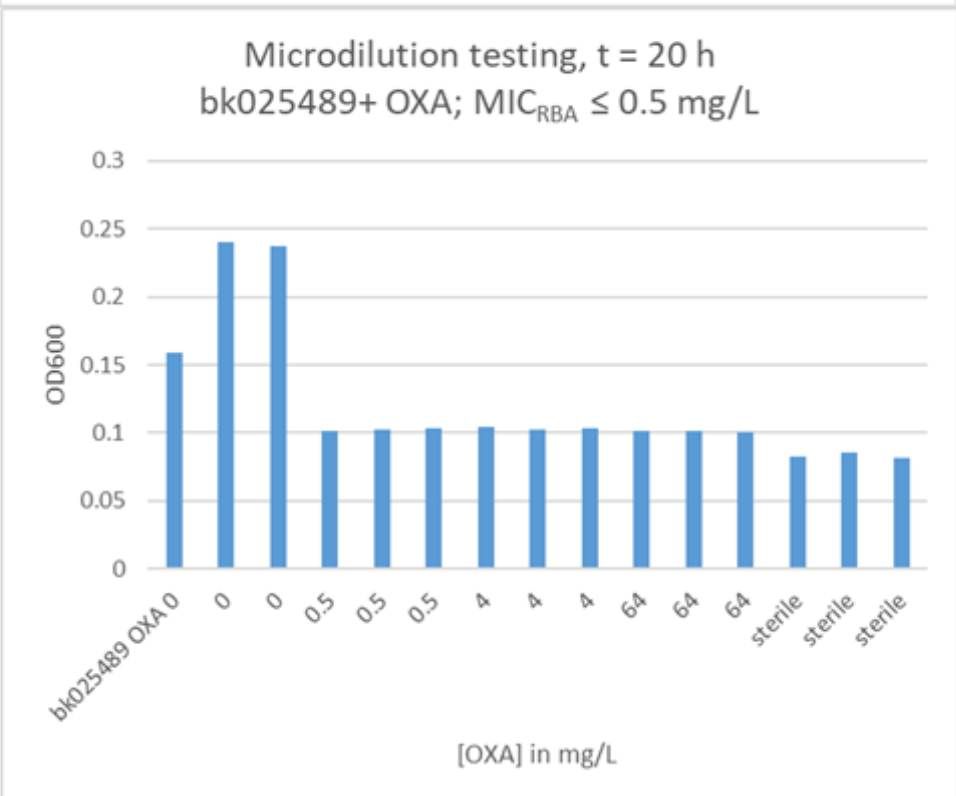

SI 9: patient blood culture results (clinical routine)

				Vitek Coli												Vitek Aureus															
#No	Date	Strain	Date Raman	ESBL	Amoxicillin	Ampi/Sulb	Pip/Taz	Cefotaxim	Ceftazidim	Meropenem	Cipro	Trim/Sulf	Gentamicin	Tigecyclin	Nitrofurantoin	Makrolide	Clinda	Vanco	Pen	Flucloxacillin	Linezolid	Rifampicin	Dapto	Fosfomycin	Cipro	Moxifloxacin	Doxycxclin	Trim/Sulf	Gentamicin	Tigecyclin	ttp
bk022768	09.10.2023	E.Coli	Raman 10.10.23	neg	S	S	S	S	S	S	R	R	S	S	S																9.3
bk022803	09.10.2023	S. aureus	Raman 10.10.23													S	S	S	S	S	S	S	S	S	I	S	S	S	S	S	19.1
bk022991	11.10.2023	E. Coli	Raman 12.10.23	neg	S	S	S	S	S	S	S	S	S	S	S																8.7
bk023014	11.10.2023	S. aureus	Raman 12.10.23													S	S	S	S	S	S	S	S	S	I	S	S	S	S	S	3.9
bk023053	12.10.2023	E. Coli	Raman 13.10.23	neg	S	S	S	S	S	S	S	S	S	S	S																1.8
bk023067	12.10.2023	E. Coli	Raman 13.10.23	neg	S	S	S	S	S	S	S	S	S	S	S																5.5
bk025257	08.11.2023	E. Coli	Raman 09.11.2023	neg	S	S	S	S	S	S	S	S	S	S	S																4.6
bk025427	09.11.2023	E. Coli	Raman 10.11.2023	neg	R	R	S	S	S	S	S	R	S	S	S																4.8
bk025489	09.11.2023	S. aureus	Raman 10.11.2023													S	S	S	S	S	S	S	S	S	I	S	S	S	S	S	6.4

↓

Susceptibility to isoxazolyl penicillins (including flucloxacillin) are inferred from the oxacillin/cefoxitin screen.
Therefore, S/I/R assigments of S. aureus treated with Flocloxacillin can be transferred to S. aureus treated with Oxacillin.

reference: The European Committee on Antimicrobial Susceptibility Testing. Breakpoint tables for interpretation of MICs and zone diameters. Version 14.0, 2024. http://www.eucast.org.

"MRSA is based on AST results for cefoxitin or, if unavailable, oxacillin. AST results reported for cloxacillin, dicloxacillin, flucloxacillin or meticillin are accepted as a marker for oxacillin resistance, if oxacillin is not reported. If no phenotypic results are available, data from molecular confirmation tests (detection of mecA gene PCR or a positive PBP2A-agglutination test) are accepted as a marker for MRSA."

reference: European Centre for Disease Prevention and Control. Antimicrobial resistance in the EU/EEA (EARS-Net) -Annual Epidemiological Report 2023. Stockholm: ECDC; 2024.

SI 9: patient blood culture turnaround times (RBA)

zip_file	file	date	device	calibration	origin	strain	batch	antibiotic	Antibiotic (Abbr.)	time-to-positivity (blood culture)	isolation time [min]	Duration of incubation (min)	end of incubati	end of measurme	∑ time blood culture till end of measureme			tTAT	treatment concentration [mg	growth expected	Position in chip/ picture na	Patient	experimentator	Raman operator	comment	Objective	Raman measurement mode	Laser wavelength [nm]	Spectral center [1/cm]	grating [l/mm]	wavenumber region [1/cm]	Laser power [mW]	integration time [s]	number spectra (theoretically)	Medium
	si cailb 785-2-1	10.10.2023	Renishaw-Inverse-G2-40-Well Chip 200 µm	1	calibration material		1																Richard	Richard	Silicon after internal calibration	50x/0.50 (Leica)	Static	785	520	600		300	0.5	1	Silicon
	dark calib	10.10.2023	Renishaw-Inverse-G2-40-Well Chip 200 µm	1	calibration material		1																Richard	Richard	dark current	50x/0.50 (Leica)	Static	785	520	600		0	0.5	1	Silicon
	4app calib	10.10.2023	Renishaw-Inverse-G2-40-Well Chip 200 µm	1	calibration material		1																Richard	Richard	Paracetamol tablet	50x/0.50 (Leica)	Static	785	1250	600		300	10	1	Paracetamol
s.a. 1.zip	s.a. reale BK, 785, pos a1.txt	10.10.2023	Renishaw-Inverse-G2-40-Well Chip 200 µm	1	real blood culture	S. aureus 1	1	Oxacillin	OXA		20	90							0	yes	a1	bk022803	Richard	Richard	90 min incubation with OXA in flasks at 160 rpm, sample isolated from patient blood culture, application into chip	63x/1.20 W (Leica)	Static	785	1405	600	500-2400	300	20	3	AT2+0.5PBS
s.a. 1.zip	s.a. reale BK, 785, pos a3.txt	10.10.2023	Renishaw-Inverse-G2-40-Well Chip 200 µm	1	real blood culture	S. aureus 1	1	Oxacillin	OXA		20	90							0	yes	a3	bk022803	Richard	Richard	90 min incubation with OXA in flasks at 160 rpm, sample isolated from patient blood culture, application into chip	63x/1.20 W (Leica)	Static	785	1405	600	500-2400	300	20	3	AT2+0.5PBS
s.a. 1.zip	s.a. reale BK, 785, pos a4 ausserhalb.txt	10.10.2023	Renishaw-Inverse-G2-40-Well Chip 200 µm	1	real blood culture	S. aureus 1	1	Oxacillin	OXA		20	90							0.25	no	a4 ausserhalb	bk022803	Richard	Richard	90 min incubation with OXA in flasks at 160 rpm, sample isolated from patient blood culture, application into chip	63x/1.20 W (Leica)	Static	785	1405	600	500-2400	300	20	3	AT2+0.5PBS
s.a. 1.zip	s.a. reale BK, 785, pos a5 ausserhalb.txt	10.10.2023	Renishaw-Inverse-G2-40-Well Chip 200 µm	1	real blood culture	S. aureus 1	1	Oxacillin	OXA		20	90							0.25	no	a5 ausserhalb	bk022803	Richard	Richard	90 min incubation with OXA in flasks at 160 rpm, sample isolated from patient blood culture, application into chip	63x/1.20 W (Leica)	Static	785	1405	600	500-2400	300	20	3	AT2+0.5PBS
s.a. 1.zip	s.a. reale BK, 785, pos b1 ausserhalb-1.txt	10.10.2023	Renishaw-Inverse-G2-40-Well Chip 200 µm	1	real blood culture	S. aureus 1	1	Oxacillin	OXA		20	90							2	no	b1 ausserhalb	bk022803	Richard	Richard	90 min incubation with OXA in flasks at 160 rpm, sample isolated from patient blood culture, application into chip	63x/1.20 W (Leica)	Static	785	1405	600	500-2400	300	20	3	AT2+0.5PBS
s.a. 1.zip	s.a. reale BK, 785, pos b3 ausserhalb-1.txt	10.10.2023	Renishaw-Inverse-G2-40-Well Chip 200 µm	1	real blood culture	S. aureus 1	1	Oxacillin	OXA		20	90							2	no	b3 ausserhalb	bk022803	Richard	Richard	90 min incubation with OXA in flasks at 160 rpm, sample isolated from patient blood culture, application into chip	63x/1.20 W (Leica)	Static	785	1405	600	500-2400	300	20	3	AT2+0.5PBS
s.a. 1.zip	s.a. reale BK, 785, pos b4 ausserhalb.txt	10.10.2023	Renishaw-Inverse-G2-40-Well Chip 200 µm	1	real blood culture	S. aureus 1	1	Oxacillin	OXA		20	90							32	no	b4 ausserhalb	bk022803	Richard	Richard	90 min incubation with OXA in flasks at 160 rpm, sample isolated from patient blood culture, application into chip	63x/1.20 W (Leica)	Static	785	1405	600	500-2400	300	20	3	AT2+0.5PBS
s.a. 1.zip	s.a. reale BK, 785, pos b5 ausserhalb-1.txt	10.10.2023	Renishaw-Inverse-G2-40-Well Chip 200 µm	1	real blood culture	S. aureus 1	1	Oxacillin	OXA	19:23:00	20	90	12:40	13:54	184			1347	32	no	b5 ausserhalb	bk022803	Richard	Richard	90 min incubation with OXA in flasks at 160 rpm, sample isolated from patient blood culture, application into chip	63x/1.20 W (Leica)	Static	785	1405	600	500-2400	300	20	3	AT2+0.5PBS
	si calib 785-4	12.10.2023	Renishaw-Inverse-G2-40-Well Chip 200 µm	1	calibration material		2																Richard	Richard	Silicon after internal calibration	50x/0.50 (Leica)	Static	785	520	600		300	0.5	1	Silicon
	dark calib	12.10.2023	Renishaw-Inverse-G2-40-Well Chip 200 µm	1	calibration material		2																Richard	Richard	dark current	50x/0.50 (Leica)	Static	785	520	600		0	0.5	1	Silicon
	4aap 785 calib-2	12.10.2023	Renishaw-Inverse-G2-40-Well Chip 200 µm	1	calibration material		2																Richard	Richard	Paracetamol tablet	50x/0.50 (Leica)	Static	785	1250	600		300	10	1	Paracetamol
s.a. 2.zip	s.a. 2, 785, pos c2 ausserhalb.txt	12.10.2023	Renishaw-Inverse-G2-40-Well Chip 200 µm	1	real blood culture	S.aureus 2	2	Oxacillin	OXA		20	90							0	yes	c2	bk023014	Richard	Richard	90 min incubation with OXA in flasks at 160 rpm, sample isolated from patient blood culture, application into chip	63x/1.20 W (Leica)	Static	785	1405	600	500-2400	300	20	3	AT2+0.5PBS
s.a. 2.zip	s.a. 2, 785, pos c3 ausserhalb..txt	12.10.2023	Renishaw-Inverse-G2-40-Well Chip 200 µm	1	real blood culture	S.aureus 2	2	Oxacillin	OXA		20	90							0	yes	c3	bk023014	Richard	Richard	90 min incubation with OXA in flasks at 160 rpm, sample isolated from patient blood culture, application into chip	63x/1.20 W (Leica)	Static	785	1405	600	500-2400	300	20	3	AT2+0.5PBS
s.a. 2.zip	s.a. 2, 785, pos c4 ausserhalb..txt	12.10.2023	Renishaw-Inverse-G2-40-Well Chip 200 µm	1	real blood culture	S.aureus 2	2	Oxacillin	OXA		20	90							0.25	yes	c4	bk023014	Richard	Richard	90 min incubation with OXA in flasks at 160 rpm, sample isolated from patient blood culture, application into chip	63x/1.20 W (Leica)	Static	785	1405	600	500-2400	300	20	3	AT2+0.5PBS
s.a. 2.zip	s.a. 2, 785, pos c5 ausserhalb..txt	12.10.2023	Renishaw-Inverse-G2-40-Well Chip 200 µm	1	real blood culture	S.aureus 2	2	Oxacillin	OXA		20	90							0.25	yes	c5	bk023014	Richard	Richard	90 min incubation with OXA in flasks at 160 rpm, sample isolated from patient blood culture, application into chip	63x/1.20 W (Leica)	Static	785	1405	600	500-2400	300	20	3	AT2+0.5PBS
s.a. 2.zip	s.a. 2, 785, pos d1 ausserhalb.txt	12.10.2023	Renishaw-Inverse-G2-40-Well Chip 200 µm	1	real blood culture	S.aureus 2	2	Oxacillin	OXA		20	90							2	no	d1	bk023014	Richard	Richard	90 min incubation with OXA in flasks at 160 rpm, sample isolated from patient blood culture, application into chip	63x/1.20 W (Leica)	Static	785	1405	600	500-2400	300	20	3	AT2+0.5PBS
s.a. 2.zip	s.a. 2, 785, pos d2.txt	12.10.2023	Renishaw-Inverse-G2-40-Well Chip 200 µm	1	real blood culture	S.aureus 2	2	Oxacillin	OXA		20	90							2	no	d2	bk023014	Richard	Richard	90 min incubation with OXA in flasks at 160 rpm, sample isolated from patient blood culture, application into chip	63x/1.20 W (Leica)	Static	785	1405	600	500-2400	300	20	3	AT2+0.5PBS
s.a. 2.zip	s.a. 2, 785, pos d4.txt	12.10.2023	Renishaw-Inverse-G2-40-Well Chip 200 µm	1	real blood culture	S.aureus 2	2	Oxacillin	OXA		20	90							32	no	d4	bk023014	Richard	Richard	90 min incubation with OXA in flasks at 160 rpm, sample isolated from patient blood culture, application into chip	63x/1.20 W (Leica)	Static	785	1405	600	500-2400	300	20	3	AT2+0.5PBS
s.a. 2.zip	s.a. 2, 785, pos d5 ausserhalb-1.txt	12.10.2023	Renishaw-Inverse-G2-40-Well Chip 200 µm	1	real blood culture	S.aureus 2	2	Oxacillin	OXA	04:54:00	20	90	12:05	13:13	178			472	32	no	d5	bk023014	Richard	Richard	90 min incubation with OXA in flasks at 160 rpm, sample isolated from patient blood culture, application into chip	63x/1.20 W (Leica)	Static	785	1405	600	500-2400	300	20	3	AT2+0.5PBS
	si calib 785-4	10.11.2023	Renishaw-Inverse-G2-40-Well Chip 200 µm	1	calibration material		3																Richard	Richard	Silicon after internal calibration	50x/0.50 (Leica)	Static	785	520	600		300	0.5	1	Silicon
	dark	10.11.2023	Renishaw-Inverse-G2-40-Well Chip 200 µm	1	calibration material		3																Richard	Richard	dark current	50x/0.50 (Leica)	Static	785	520	600		0	0.5	1	Silicon
	4aap calib	10.11.2023	Renishaw-Inverse-G2-40-Well Chip 200 µm	1	calibration material		3																Richard	Richard	Paracetamol tablet	50x/0.50 (Leica)	Static	785	1250	600		300	10	1	Paracetamol
s.a. 3.zip	s. aureus 3, 785, pos c7.txt	10.11.2023	Renishaw-Inverse-G2-40-Well Chip 200 µm	1	real blood culture	S. aureus 3	3	Oxacillin	OXA		20	90							0	yes	c7	bk025489	Richard	Richard	90 min incubation with OXA in flasks at 160 rpm, sample isolated from patient blood culture, application into chip	63x/1.20 W (Leica)	Static	785	1405	600	500-2400	300	20	3	AT2+0.5PBS
s.a. 3.zip	s. aureus 3, 785, pos c8.txt	10.11.2023	Renishaw-Inverse-G2-40-Well Chip 200 µm	1	real blood culture	S. aureus 3	3	Oxacillin	OXA		20	90							0	yes	c8	bk025489	Richard	Richard	90 min incubation with OXA in flasks at 160 rpm, sample isolated from patient blood culture, application into chip	63x/1.20 W (Leica)	Static	785	1405	600	500-2400	300	20	3	AT2+0.5PBS
s.a. 3.zip	s. aureus 3, 785, pos c9-1.txt	10.11.2023	Renishaw-Inverse-G2-40-Well Chip 200 µm	1	real blood culture	S. aureus 3	3	Oxacillin	OXA		20	90							0.25	no	c9	bk025489	Richard	Richard	90 min incubation with OXA in flasks at 160 rpm, sample isolated from patient blood culture, application into chip	63x/1.20 W (Leica)	Static	785	1405	600	500-2400	300	20	3	AT2+0.5PBS
s.a. 3.zip	s. aureus 3, 785, pos c10.txt	10.11.2023	Renishaw-Inverse-G2-40-Well Chip 200 µm	1	real blood culture	S. aureus 3	3	Oxacillin	OXA		20	90							0.25	no	c10	bk025489	Richard	Richard	90 min incubation with OXA in flasks at 160 rpm, sample isolated from patient blood culture, application into chip	63x/1.20 W (Leica)	Static	785	1405	600	500-2400	300	20	3	AT2+0.5PBS
s.a. 3.zip	s. aureus 3, 785, pos d7.txt	10.11.2023	Renishaw-Inverse-G2-40-Well Chip 200 µm	1	real blood culture	S. aureus 3	3	Oxacillin	OXA		20	90							2	no	d7	bk025489	Richard	Richard	90 min incubation with OXA in flasks at 160 rpm, sample isolated from patient blood culture, application into chip	63x/1.20 W (Leica)	Static	785	1405	600	500-2400	300	20	3	AT2+0.5PBS
s.a. 3.zip	s. aureus 3, 785, pos d8.txt	10.11.2023	Renishaw-Inverse-G2-40-Well Chip 200 µm	1	real blood culture	S. aureus 3	3	Oxacillin	OXA		20	90							2	no	d8	bk025489	Richard	Richard	90 min incubation with OXA in flasks at 160 rpm, sample isolated from patient blood culture, application into chip	63x/1.20 W (Leica)	Static	785	1405	600	500-2400	300	20	3	AT2+0.5PBS
s.a. 3.zip	s. aureus 3, 785, pos d9.txt	10.11.2023	Renishaw-Inverse-G2-40-Well Chip 200 µm	1	real blood culture	S. aureus 3	3	Oxacillin	OXA		20	90							32	no	d9	bk025489	Richard	Richard	90 min incubation with OXA in flasks at 160 rpm, sample isolated from patient blood culture, application into chip	63x/1.20 W (Leica)	Static	785	1405	600	500-2400	300	20	3	AT2+0.5PBS
s.a. 3.zip	s. aureus 3, 785, pos d10.txt	10.11.2023	Renishaw-Inverse-G2-40-Well Chip 200 µm	1	real blood culture	S. aureus 3	3	Oxacillin	OXA	05:27:00	20	90	13:10	14:19	179			506	32	no	d10	bk025489	Richard	Richard	90 min incubation with OXA in flasks at 160 rpm, sample isolated from patient blood culture, application into chip	63x/1.20 W (Leica)	Static	785	1405	600	500-2400	300	20	3	AT2+0.5PBS
[redacted]																																			
	si cailb 785-2-1	10.10.2023	Renishaw-Inverse-G2-40-Well Chip 200 µm	1	calibration material		1																Richard	Richard	Silicon after internal calibration	50x/0.50 (Leica)	Static	785	520	600		300	0.5	1	Silicon
	dark calib	10.10.2023	Renishaw-Inverse-G2-40-Well Chip 200 µm	1	calibration material		1																Richard	Richard	dark current	50x/0.50 (Leica)	Static	785	520	600		0	0.5	1	Silicon
	4app calib	10.10.2023	Renishaw-Inverse-G2-40-Well Chip 200 µm	1	calibration material		1																Richard	Richard	Paracetamol tablet	50x/0.50 (Leica)	Static	785	1250	600		300	10	1	Paracetamol
e.c. 1.zip	e.c. 1, 532, pos a6.txt	10.10.2023	Renishaw-Inverse-G2-40-Well Chip 200 µm	1	real blood culture	E. coli 1	1	Ciprofloxacin	CIP		20	90							0	yes	a6	bk022768	Richard	Richard	90 min incubation with CIP in flasks at 160 rpm, sample isolated from patient blood culture, application into chip	63x/1.20 W (Leica)	Static	785	1405	600	500-2400	300	20	3	AT2+0.5PBS
e.c. 1.zip	e.c. 1, 532, pos a7.txt	10.10.2023	Renishaw-Inverse-G2-40-Well Chip 200 µm	1	real blood culture	E. coli 1	1	Ciprofloxacin	CIP		20	90							0	yes	a7	bk022768	Richard	Richard	90 min incubation with CIP in flasks at 160 rpm, sample isolated from patient blood culture, application into chip	63x/1.20 W (Leica)	Static	785	1405	600	500-2400	300	20	3	AT2+0.5PBS
e.c. 1.zip	e.c. 1, 532, pos a9.txt	10.10.2023	Renishaw-Inverse-G2-40-Well Chip 200 µm	1	real blood culture	E. coli 1	1	Ciprofloxacin	CIP		20	90							0.016	yes	a9	bk022768	Richard	Richard	90 min incubation with CIP in flasks at 160 rpm, sample isolated from patient blood culture, application into chip	63x/1.20 W (Leica)	Static	785	1405	600	500-2400	300	20	3	AT2+0.5PBS
e.c. 1.zip	e.c. 1, 785, pos a10.txt	10.10.2023	Renishaw-Inverse-G2-40-Well Chip 200 µm	1	real blood culture	E. coli 1	1	Ciprofloxacin	CIP		20	90							0.016	yes	a10	bk022768	Richard	Richard	90 min incubation with CIP in flasks at 160 rpm, sample isolated from patient blood culture, application into chip	63x/1.20 W (Leica)	Static	785	1405	600	500-2400	300	20	3	AT2+0.5PBS
e.c. 1.zip	e.c. 1, 785, pos b6.txt	10.10.2023	Renishaw-Inverse-G2-40-Well Chip 200 µm	1	real blood culture	E. coli 1	1	Ciprofloxacin	CIP		20	90							0.25	yes	b6	bk022768	Richard	Richard	90 min incubation with CIP in flasks at 160 rpm, sample isolated from patient blood culture, application into chip	63x/1.20 W (Leica)	Static	785	1405	600	500-2400	300	20	3	AT2+0.5PBS
e.c. 1.zip	e.c. 1, 785, pos b8.txt	10.10.2023	Renishaw-Inverse-G2-40-Well Chip 200 µm	1	real blood culture	E. coli 1	1	Ciprofloxacin	CIP		20	90							0.25	yes	b8	bk022768	Richard	Richard	90 min incubation with CIP in flasks at 160 rpm, sample isolated from patient blood culture, application into chip	63x/1.20 W (Leica)	Static	785	1405	600	500-2400	300	20	3	AT2+0.5PBS
e.c. 1.zip	e.c. 1, 785, pos b9.txt	10.10.2023	Renishaw-Inverse-G2-40-Well Chip 200 µm	1	real blood culture	E. coli 1	1	Ciprofloxacin	CIP		20	90							2	yes	b9	bk022768	Richard	Richard	90 min incubation with CIP in flasks at 160 rpm, sample isolated from patient blood culture, application into chip	63x/1.20 W (Leica)	Static	785	1405	600	500-2400	300	20	3	AT2+0.5PBS
e.c. 1.zip	e.c. 1, 785, pos b10.txt	10.10.2023	Renishaw-Inverse-G2-40-Well Chip 200 µm	1	real blood culture	E. coli 1	1	Ciprofloxacin	CIP	09:21:00	20	90	14:22	15:26	174			735	2	yes	b10	bk022768	Richard	Richard	90 min incubation with CIP in flasks at 160 rpm, sample isolated from patient blood culture, application into chip	63x/1.20 W (Leica)	Static	785	1405	600	500-2400	300	20	3	AT2+0.5PBS
	dark	13.10.2023	Renishaw-Inverse-G2-40-Well Chip 200 µm	1	calibration material		2																Richard	Richard	dark current	50x/0.50 (Leica)	Static	785	520	600		0	0.5	1	Silicon
	4aap 785 calib-2	13.10.2023	Renishaw-Inverse-G2-40-Well Chip 200 µm	1	calibration material		2																Richard	Richard	Paracetamol tablet	50x/0.50 (Leica)	Static	785	1250	600		300	10	1	Paracetamol
e.c. 4.zip	e.c. 4, 785, pos a8.txt	13.10.2023	Renishaw-Inverse-G2-40-Well Chip 200 µm	0	real blood culture	E.coli 4	2	Ciprofloxacin	CIP		20	90							0	yes	a6	bk023067	Richard	Richard	90 min incubation with CIP in flasks at 160 rpm, sample isolated from patient blood culture, application into chip	63x/1.20 W (Leica)	Static	785	1405	600	500-2400	300	20	3	AT2+0.5PBS
e.c. 4.zip	e.c. 4, 785, pos a8-1.txt	13.10.2023	Renishaw-Inverse-G2-40-Well Chip 200 µm	0	real blood culture	E.coli 4	2	Ciprofloxacin	CIP	18:53:00	20	90	11:55	14:04	239			1372	0	yes	a8	bk023067	Richard	Richard	90 min incubation with CIP in flasks at 160 rpm, sample isolated from patient blood culture, application into chip	63x/1.20 W (Leica)	Static	785	1405	600	500-2400	300	20	3	AT2+0.5PBS
e.c. 4.zip	e.c. 4, 785, pos a9-1.txt	13.10.2023	Renishaw-Inverse-G2-40-Well Chip 200 µm	0	real blood culture	E.coli 4	2	Ciprofloxacin	CIP		20	90							0.016	yes	a9	bk023067	Richard	Richard	90 min incubation with CIP in flasks at 160 rpm, sample isolated from patient blood culture, application into chip	63x/1.20 W (Leica)	Static	785	1405	600	500-2400	300	20	3	AT2+0.5PBS
e.c. 4.zip	e.c. 4, 785, pos a10.txt	13.10.2023	Renishaw-Inverse-G2-40-Well Chip 200 µm	0	real blood culture	E.coli 4	2	Ciprofloxacin	CIP		20	90							0.016	yes	a10	bk023067	Richard	Richard	90 min incubation with CIP in flasks at 160 rpm, sample isolated from patient blood culture, application into chip	63x/1.20 W (Leica)	Static	785	1405	600	500-2400	300	20	3	AT2+0.5PBS
e.c. 4.zip	e.c. 4, 785, pos b6.txt	13.10.2023	Renishaw-Inverse-G2-40-Well Chip 200 µm	0	real blood culture	E.coli 4	2	Ciprofloxacin	CIP		20	90							0.25	yes	b6	bk023067	Richard	Richard	90 min incubation with CIP in flasks at 160 rpm, sample isolated from patient blood culture, application into chip	63x/1.20 W (Leica)	Static	785	1405	600	500-2400	300	20	3	AT2+0.5PBS
e.c. 4.zip	e.c. 4, 785, pos b8.txt	13.10.2023	Renishaw-Inverse-G2-40-Well Chip 200 µm	0	real blood culture	E.coli 4	2	Ciprofloxacin	CIP		20	90							0.25	yes	b8	bk023067	Richard	Richard	90 min incubation with CIP in flasks at 160 rpm, sample isolated from patient blood culture, application into chip	63x/1.20 W (Leica)	Static	785	1405	600	500-2400	300	20	3	AT2+0.5PBS
e.c. 4.zip	e.c. 4, 785, pos b9.txt	13.10.2023	Renishaw-Inverse-G2-40-Well Chip 200 µm	0	real blood culture	E.coli 4	2	Ciprofloxacin	CIP		20	90							2	no	b9	bk023067	Richard	Richard	90 min incubation with CIP in flasks at 160 rpm, sample isolated from patient blood culture, application into chip	63x/1.20 W (Leica)	Static	785	1405	600	500-2400	300	20	3	AT2+0.5PBS
e.c. 4.zip	e.c. 4, 785, pos b10.txt	13.10.2023	Renishaw-Inverse-G2-40-Well Chip 200 µm	0	real blood culture	E.coli 4	2	Ciprofloxacin	CIP		20	90							2	no	b10	bk023067	Richard	Richard	90 min incubation with CIP in flasks at 160 rpm, sample isolated from patient blood culture, application into chip	63x/1.20 W (Leica)	Static	785	1405	600	500-2400	300	20	3	AT2+0.5PBS
	si calib 785-4	10.11.2023	Renishaw-Inverse-G2-40-Well Chip 200 µm	1	calibration material		3																Richard	Richard	Silicon after internal calibration	50x/0.50 (Leica)	Static	785	520	600		300	0.5	1	Silicon
	4aap calib	10.11.2023	Renishaw-Inverse-G2-40-Well Chip 200 µm	1	calibration material		3																Richard	Richard	dark current	50x/0.50 (Leica)	Static	785	520	600		0	0.5	1	Silicon
	dark	10.11.2023	Renishaw-Inverse-G2-40-Well Chip 200 µm	1	calibration material		3																Richard	Richard	Paracetamol tablet	50x/0.50 (Leica)	Static	785	1250	600		300	10	1	Paracetamol
e.c. 6.zip	e. coli 6, 785, pos c1.txt	10.11.2023	Renishaw-Inverse-G2-40-Well Chip 200 µm	1	real blood culture	E. coli 6	3	Ciprofloxacin	CIP		20	90							0	yes	c1	bk025427	Richard	Richard	90 min incubation with CIP in flasks at 160 rpm, sample isolated from patient blood culture, application into chip	63x/1.20 W (Leica)	Static	785	1405	600	500-2400	300	20	3	AT2+0.5PBS
e.c. 6.zip	e. coli 6, 785, pos c3.txt	10.11.2023	Renishaw-Inverse-G2-40-Well Chip 200 µm	1	real blood culture	E. coli 6	3	Ciprofloxacin	CIP		20	90							0	yes	c3	bk025427	Richard	Richard	90 min incubation with CIP in flasks at 160 rpm, sample isolated from patient blood culture, application into chip	63x/1.20 W (Leica)	Static	785	1405	600	500-2400	300	20	3	AT2+0.5PBS
e.c. 6.zip	e. coli 6, 785, pos c4.txt	10.11.2023	Renishaw-Inverse-G2-40-Well Chip 200 µm	1	real blood culture	E. coli 6	3	Ciprofloxacin	CIP		20	90							0.016	yes	c4	bk025427	Richard	Richard	90 min incubation with CIP in flasks at 160 rpm, sample isolated from patient blood culture, application into chip	63x/1.20 W (Leica)	Static	785	1405	600	500-2400	300	20	3	AT2+0.5PBS
e.c. 6.zip	e. coli 6, 785, pos c5.txt	10.11.2023	Renishaw-Inverse-G2-40-Well Chip 200 µm	1	real blood culture	E. coli 6	3	Ciprofloxacin	CIP		20	90							0.016	yes	c5	bk025427	Richard	Richard	90 min incubation with CIP in flasks at 160 rpm, sample isolated from patient blood culture, application into chip	63x/1.20 W (Leica)	Static	785	1405	600	500-2400	300	20	3	AT2+0.5PBS
e.c. 6.zip	e. coli 6, 785, pos d1.txt	10.11.2023	Renishaw-Inverse-G2-40-Well Chip 200 µm	1	real blood culture	E. coli 6	3	Ciprofloxacin	CIP		20	90							0.25	no	d1	bk025427	Richard	Richard	90 min incubation with CIP in flasks at 160 rpm, sample isolated from patient blood culture, application into chip	63x/1.20 W (Leica)	Static	785	1405	600	500-2400	300	20	3	AT2+0.5PBS
e.c. 6.zip	e. coli 6, 785, pos d3.txt	10.11.2023	Renishaw-Inverse-G2-40-Well Chip 200 µm	1	real blood culture	E. coli 6	3	Ciprofloxacin	CIP		20	90							0.25	no	d3	bk025427	Richard	Richard	90 min incubation with CIP in flasks at 160 rpm, sample isolated from patient blood culture, application into chip	63x/1.20 W (Leica)	Static	785	1405	600	500-2400	300	20	3	AT2+0.5PBS
e.c. 6.zip	e. coli 6, 785, pos d4.txt	10.11.2023	Renishaw-Inverse-G2-40-Well Chip 200 µm	1	real blood culture	E. coli 6	3	Ciprofloxacin	CIP		20	90							2	no	d4	bk025427	Richard	Richard	90 min incubation with CIP in flasks at 160 rpm, sample isolated from patient blood culture, application into chip	63x/1.20 W (Leica)	Static	785	1405	600	500-2400	300	20	3	AT2+0.5PBS
e.c. 6.zip	e. coli 6, 785, pos d5.txt	10.11.2023	Renishaw-Inverse-G2-40-Well Chip 200 µm	1	real blood culture	E. coli 6	3	Ciprofloxacin	CIP	10:51:00	20	90	11:40	12:33	163			814	2	no	d5	bk025427	Richard	Richard	90 min incubation with CIP in flasks at 160 rpm, sample isolated from patient blood culture, application into chip	63x/1.20 W (Leica)	Static	785	1405	600	500-2400	300	20	3	AT2+0.5PBS
														**mean turnaround time [min]**	**186**	**03:06:10**	**mean total turnaround time [min]**	**874**	**14:34:20**																
														**standard deviation [min]**	**24**	**00:24:29**	**standard deviation [min]**	**398**	**06:37:55**																

SI 9: patient blood culture turnaround times (clinical routine)

internal name	bk sample	laboratory entrance	positive flag	time-to-positivity	ID	ID method	start Vitek	Resistance from Vitek in the system	turnaround time Vitek [hh:mm]	final resistance results released	total turnaround time
E. coli 1	bk022768	6.10.23 10:28	6.10.23 19:49	09:21:00	7.10.23 13:31	LAMP	8.10.23 10:57	8.10.23 20:19	09:22:00	9.10.23 8:02	57:51:00
S. aureus 1	bk022803	6.10.23 15:27	7.10.23 10:50	19:23:00	7.10.23 13:31	LAMP	8.10.23 10:58	9.10.23 0:28	13:30:00	9.10.23 8:03	57:01:00
S. aureus 2	bk023014	9.10.23 10:43	9.10.23 15:37	04:54:00	10.10.23 10:12	MS	10.10.23 8:32	10.10.23 22:28	13:56:00	11.10.23 8:09	35:45:00
E.coli 4	bk023067	9.10.23 18:27	10.10.23 13:20	18:53:00	11.10.23 14:12	MS	11.10.23 8:36	12.10.23 6:56	22:20:00	12.10.23 8:30	60:29:00
E. coli 6	bk025427	7.11.23 1:01	7.11.23 11:52	10:51:00	7.11.23 15:11	LAMP	8.11.23 8:05	8.11.23 18:52	10:47:00	9.11.23 8:11	41:51:00
s. aureus 3	bk025489	7.11.23 14:09	7.11.23 19:36	05:27:00	8.11.23 13:05	LAMP	9.11.23 8:20	9.11.23 21:47	13:27:00	10.11.23 9:00	55:38:00
			mean →	11:28:10				mean →	**13:53:40**	mean →	**51:25:50**
			SD →	06:21:14				SD →	**04:30:40**	SD →	**10:05:47**

SI 10: MIC-shifts due to discrepancies of EUCAST and RamanBioAssay™ (RBA) conditions.

Due to variations from EUCAST standard it was necessary to adapt the RamanBioAssay (RBA) minimal inhibitory concentrations (MIC). For this purpose, gold standard broth microdilution testing was performed with deviating RBA conditions (= adapted broth microdilution test). MIC-shifts for each bacterium-antibiotic combination were determined by the number of 2-fold dilution steps.

Condition discrepancies

	**EUCAST standard** (ISO standard 20776-1) [1]	**RBA conditions**
**medium**	cation-adjusted Mueller-Hinton broth	AT2 50 % (proprietary medium → Raman compatible)
**CFU** in ml^{-1}	$2 * 10^5$	$5 * 10^8$
**interpretation** (MIC readout)	*"[…] lowest concentration of the agent that completely inhibits visible growth."*	$\frac{OD_{600nm}\ (tested\ concentration)}{OD_{600nm}\ (growth\ control)} \leq 0{,}5$

I. MIC-shift: E. coli ATCC 25922 + Ciprofloxacin

EUCAST standard [2]	MIC shift			RBA conditions *
MIC_{range} = 0.004-0.016	→ +	3 dilutions steps (2-fold)	- ←	MIC_{range} = ≤0,03 – 0,125
MIC = 0.008	→ +		- ←	**MIC = 0.06**

*

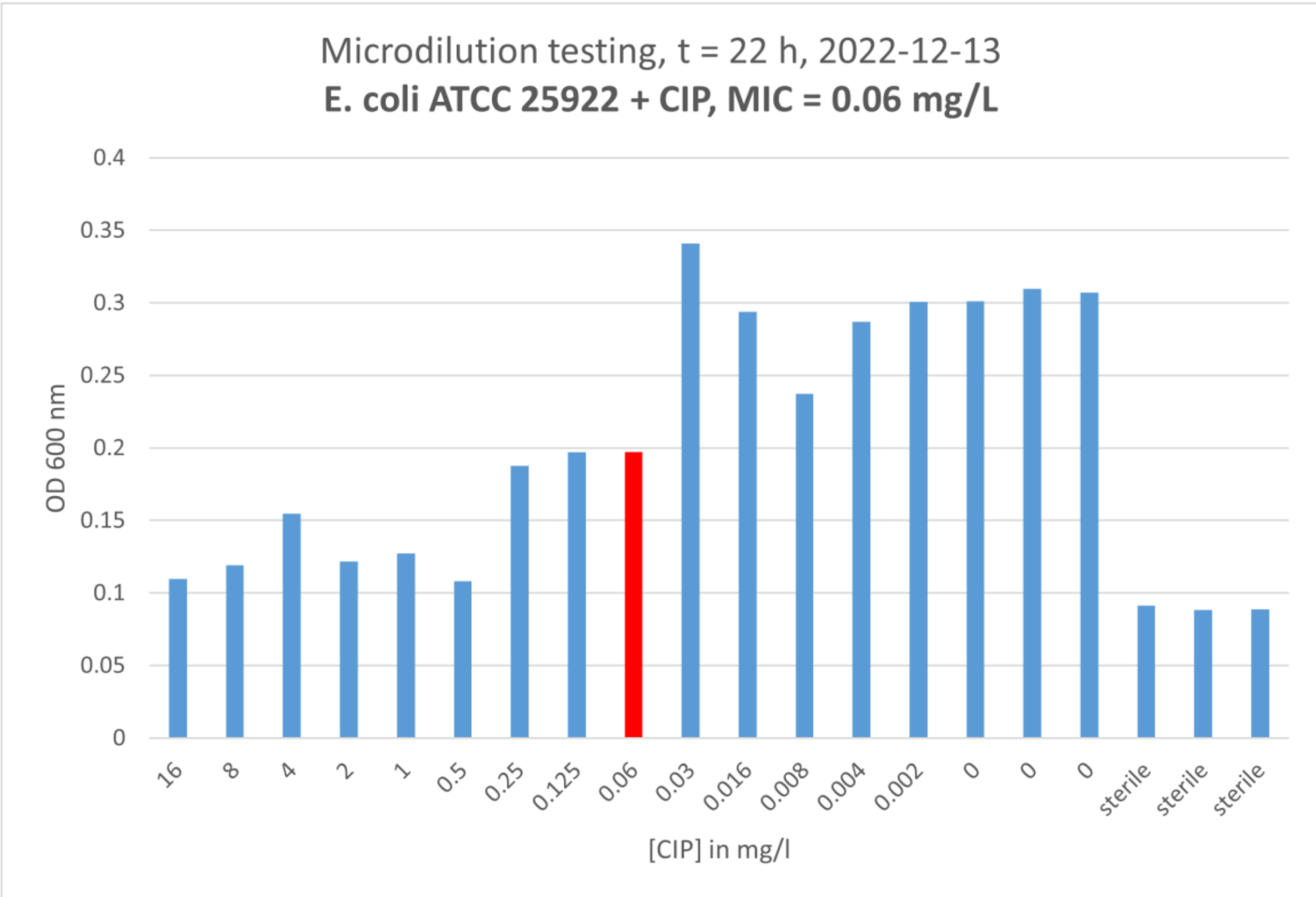

MIC was definded as first the concentration with < 50 % of the growth control's value (sterile subtracted).

II. RBA-concentrations for AST: E. coli (medium control, blood culture: spiked and patient)

antibiotic \ sensitivity	ATCC-reference					
	ATCC 25922					
	EUCAST-standard MIC in $\frac{mg}{L}$	RBA-MIC in $\frac{mg}{L}$	RBA-concentrations in $\frac{mg}{L}$			
			GC	$\mathrm{MIC}_{range} - 1$	$\mathrm{MIC}_{range} + 1$	$\mathrm{MIC} + 5$
Ciprofloxacin<br>EUCAST: S ≤ 0.25/R > 0.5	≤ 0.004 – 0.016	+ 3 dilution steps (2-fold)<br>≤ 0.03 – 0.125	0	0.016	0.25	2

growth: yes	growth: no

GC … growth control

MIC_{range} -1 … subinhibitory

MIC_{range} +1 … inhibitory

MIC + 5 … inhibition control

dilution steps (2-fold) according to EUCAST: 0.002/0.004/0.008/0.015/0.03/0.06/0.125/0.25/0.5/1/2/4/8/16/32/64/128/256/512 $\frac{mg}{L}$

III. MIC-shift: S. aureus ATCC 29213 + Oxacillin (medium control)

**EUCAST standard [2,] ** **		**MIC shift**		**RBA conditions *** **
MIC_{range} = 0.25 – 0.5	→ **+**	1 dilution step	**-** ←	MIC_{range} = 0.5 – 1
MIC = 0.25	→ **+**		**-** ←	MIC = 0.5

IV. S. aureus ATCC 29213 + Oxacillin (artifical and patient blood culture)

**EUCAST standard [2,] ** **		**MIC shift**		**RBA conditions *** **
MIC_{range} = 0.25 – 0.5	→ **-**	1 dilution steps	**+** ←	MIC_{range} = ≤0.125 – 0,25
MIC = 0.25	→ **-**		**+** ←	MIC = 0.125

** Due to an unspecified EUCAST MIC for S. aureus ATCC 29213 [2](experpt see below) a clinical routine test with Vitek 2 (bioMérieux) was additionally performed to determine the MIC_{range} and MIC.

Routine QC EUCAST QC Tables v. 14.0, valid from 2024-01-01

Staphylococcus aureus **ATCC 29213**

(NCTC 12973, CIP 103429, DSM 2569, CCUG 15915, CECT 794)

β-lactamase-producing strain (weak)

Test according to EUCAST methodology for non-fastidious organisms (MH broth and agar). See EUCAST Breakpoint Tables for short descriptions of MIC and disk diffusion methodology.

**Antimicrobial agent**	**MIC (mg/L)**		**Disk content (µg)**	**Inhibition zone diameter (mm)**	
	**Target[1]**	**Range[2]**		**Target[1]**	**Range[3]**
Oxacillin	Note[10]	Note[10]	1	***22***	***19-25***

[10] There is currently no EUCAST MIC range for *S. aureus* ATCC 29213 and oxacillin. The range in CLSI M100-S34, 2024, is 0.125-0.5 mg/L.

Kartentyp: AST-P611 Barcode: 4912098103088910 Analysegerät: 000019241232 (Bolte)
Anwender: ??? (Labsuper1)
QK-Referenznr.: ATCC29213

Kommentare	

Infos zur AST-Karte	Karte: AST-P611	Chargenbez.: 4912098103	Verfallsdat.: 25.08.2023
	Beendet: 28.06.2022 02:55 MESZ		Status: Fertig, 13,80 Std
Keim-ID:	Staphylococcus aureus ATCC 29213		
Keim eingegeben von:	System Eingegeben am: 27.06.2022 12:52 MESZ		
AST Meldungen	Folgende Antibiotika werden nicht angegeben: Gentamicin, High-Level (Synergie), Induzierbare Clindamycin Resistenz.		

Antibiotika-Details

Antibiotikum	Gefunden	Erwartet	Antibiotikum	Gefunden	Erwartet
Ampicillin	0,5	0.5 - 2	Imipenem	<=1	<=1
Benzylpenicillin	>=0,5	0.25 - >=0.5	Induzierbare Clindamycin Resistenz		
Cefoxitin-Screen	Neg	-	Linezolid	1	1 - 4
Ciprofloxacin	<=0,5	<=0.5	Moxifloxacin	<=0,25	<=0.25
Clindamycin	<=0,25	<=0.25	Oxacillin	<=0,25	<=0.25 - 0.5
Daptomycin	0,25	0.25 - 1	Rifampicin	<=0,5	<=0.5
Erythromycin	<=0,25	<=0.25 - 1	Tetracyclin	<=1	<=1
Fosfomycin	<=8	<=8	Tigecycline	<=0,12	<=0.12 - 0.25
Fusidinsäure	<=0,5	<=0.5	Trimethoprim/Sulfamethoxazol	<=10	<=10
Gentamicin	<=0,5	<=0.5 - 1	Vancomycin	<=0,5	<=0.5 - 2
Gentamicin, High-Level (Synergie)					

* Abweichendes Ergebnis

***

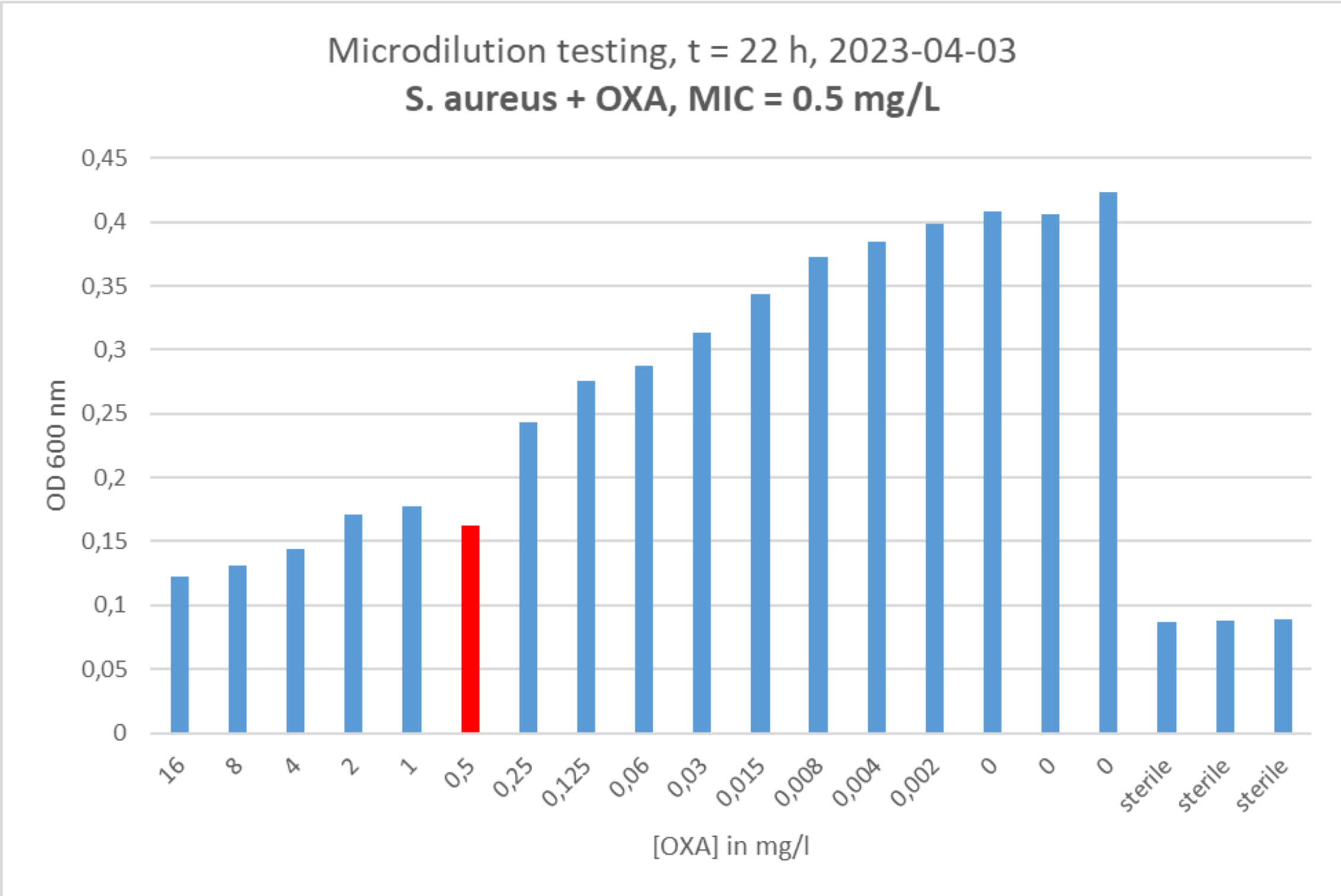

MIC was definded as first the concentration with < 50 % of the growth control's value (sterile subtracted).

V. RBA-concentrations for AST: S. aureus (medium control)

antibiotic \ sensitivity	ATCC-reference					
	ATCC 29213					
	EUCAST-standard MIC in $\frac{mg}{L}$	RBA-MIC in $\frac{mg}{L}$	RBA-concentrations in $\frac{mg}{L}$			
			GC	$\text{MIC}_{range} - 1$	$\text{MIC}_{range} + 1$	$\text{MIC} + 5$
oxacillin<br>EUCAST: S ≤ 1/R > 2	≤ 0.25 – 0.5	+ 1 dilution step (2-fold)<br>≤ 0.5 – 1	0	0.25	2	32

growth: yes	growth: no

GC … growth control

MIC_{range} -1 … subinhibitory

MIC_{range} +1 … inhibitory

MIC + 5 … inhibition control

dilution steps (2-fold) according to EUCAST: 0.002/0.004/0.008/0.015/0.03/0.06/0.125/0.25/0.5/1/2/4/8/16/32/64/128/256/512 $\frac{mg}{L}$

RBA-concentrations for AST: S. aureus (artificial and patient blood culture)

**sensitivity** / **antibiotic**	ATCC-reference					
	**ATCC 29213**					
	EUCAST-standard MIC in $\frac{mg}{L}$	RBA-MIC in $\frac{mg}{L}$	RBA-concentrations in $\frac{mg}{L}$			
			GC	$\mathrm{MIC}_{range} - 1$	$\mathrm{MIC}_{range} + 1$	$\mathrm{MIC} + 5$
oxacillin<br><br>EUCAST: S ≤ 1/R > 2	≤ 0.25 – 0.5	- 1 dilution step (2-fold)<br>*(+1: EUCAST to medium control, see above),*<br>*-2: medium control to blood culture)*<br>≤ 0.125 – 0.25	0	0.25	2	32

growth: yes	growth: no

GC … growth control

MIC_{range} -1 … subinhibitory

MIC_{range} +1 … inhibitory

MIC + 5 … inhibition control

dilution steps (2-fold) according to EUCAST: 0.002/0.004/0.008/0.015/0.03/0.06/0.125/0.25/0.5/1/2/4/8/16/32/64/128/256/512 $\frac{mg}{L}$

For isolation of *S. aureus* form blood cultures, a shift in RBA-MIC for oxacillin occurred, most likely due to induced bacterial stress. The MIC shifted by two 2-fold dilution steps resulting in a MIC_{range} of ≤ 0.125 – 0.25. Since the same RBA-concentrations were used for medium control and blood culture experiments the former subinhibitory $\mathrm{MIC}_{range} - 1$ was now inhibitory, too (see table above). This blood culture isolation-dependent MIC shift effect did not occur for *E.coli* + ciprofloxacin.

VI. Adaptation of RBA-concentrations for AST of patient blood cultures for direct comparison to standard EUCAST concentrations

**Bacteria + antibiotics, MIC_{range} and MIC under RBA-conditions**	**RBA-concentrations** as determined by BMD	**MIC-shift taken into consideration**	**Adapted RBA-concentrations** → directly comparable to standard EUCAST concentrations
E. coli + CIP<br><br>MIC_{range} ≤ 0.03 – 0.125<br>MIC = 0.06		- 3 (difference due to RBA conditions not fully compatible with EUCAST standard conditions)<br><br>0 (isolation from blood culture, no MIC shift	
GC	0	**- 3 dilution steps** (2-fold)	0
MIC_{range}-1	0.016	→	0.002
MIC_{range}+1	0.25	→	0.03
MIC+5	2	→	0.25
S. aureus + OXA<br><br>MIC_{range} ≤ 0.5 – 1<br>MIC = 0.5		- 1 (difference due to RBA conditions not fully compatible with EUCAST standard conditions)<br><br>+ 2 (isolation from blood culture)	
GC	0	**+ 1 dilution step** (2-fold)	0
MIC_{range}-1	0.25	→	0.5
MIC_{range}+1	2	→	4

MIC+5	32	→	64

References

1. EUCAST. Breakpoint tables for interpretation of MICs and zone diameters. Accessed November 11, 2024. https://www.eucast.org/fileadmin/src/media/PDFs/EUCAST_files/Breakpoint_tables/v_14.0_Breakpoint_Tables.pdf
2. EUCAST. Routine and extended internal quality control for MIC determination and disk diffusion as recommended by EUCAST. Accessed November 11, 2024. https://www.eucast.org/fileadmin/src/media/PDFs/EUCAST_files/QC/v_14.0_EUCAST_QC_tables_routine_and_extended_QC.pdf

SI 11: Graphical representation of workflow and beampath in DEP-Chip

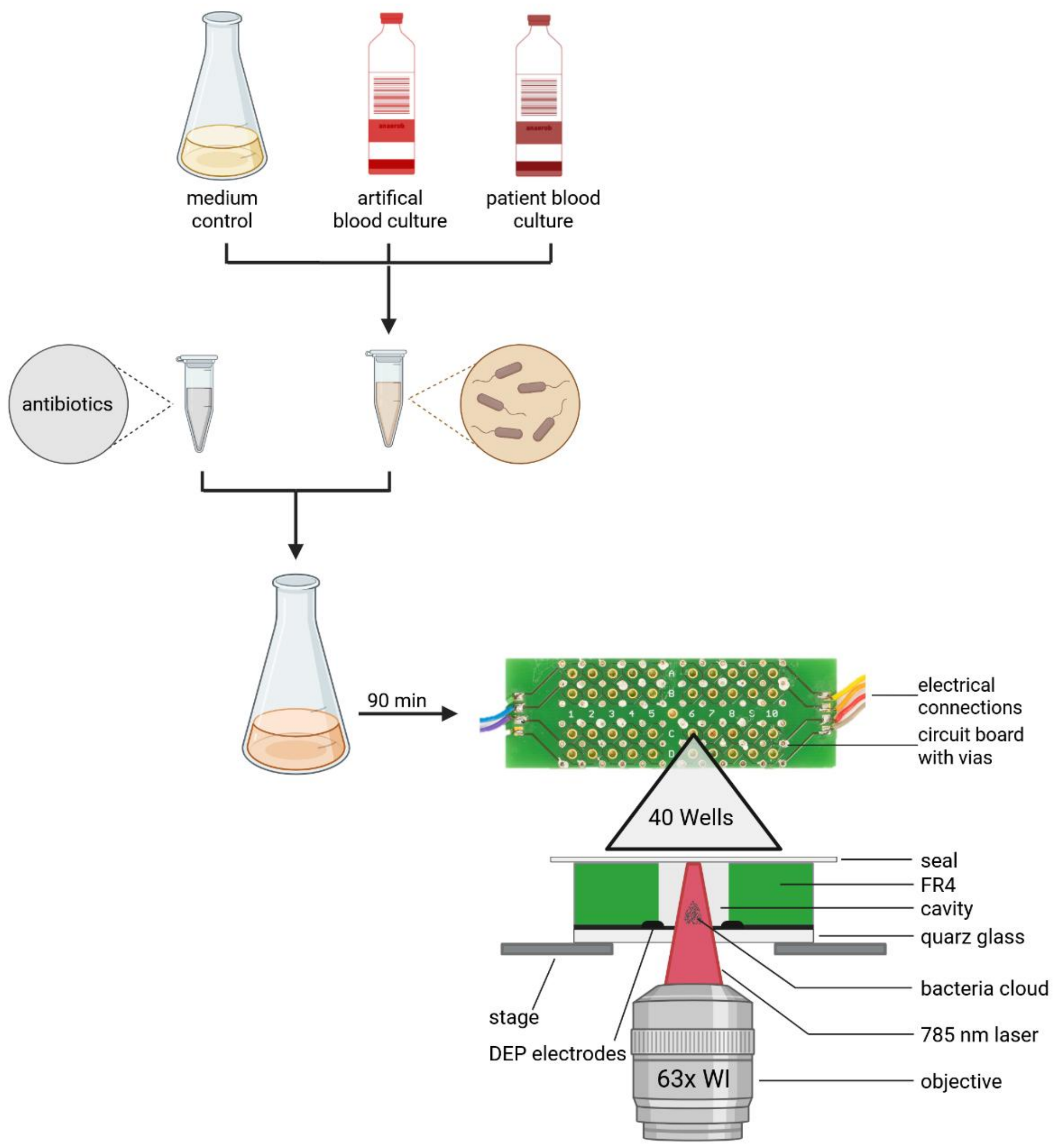

Created in BioRender. Grohs, R. (2026) https://BioRender.com/xrzfz81
